# Genome‐Wide Association Analyses Identify Hydrogen Peroxide–Responsive Loci in Wheat Diversity

**DOI:** 10.1002/pld3.70067

**Published:** 2025-04-17

**Authors:** Mohammad Kamruzzaman, Md. Nurealam Siddiqui, Samira Rustamova, Agim Ballvora, Jens Léon, Ali Ahmad Naz

**Affiliations:** ^1^ Institute of Crop Science and Resource Conservation (INRES)‐Plant Breeding and Biotechnology University of Bonn Bonn Germany; ^2^ Plant Breeding Division, Bangladesh Institute of Nuclear Agriculture (BINA) Bangladesh Agricultural University Campus Mymensingh Bangladesh; ^3^ Department of Biochemistry and Molecular Biology Gazipur Agricultural University Gazipur Bangladesh; ^4^ Institute of Molecular Biology and Biotechnologies, Ministry of Science and Education Republic of Azerbaijan Baku Azerbaijan; ^5^ Field Lab Campus Klein‐Altendorf University of Bonn Rheinbach Germany; ^6^ Department of Plant Breeding University of Applied Sciences Osnabrueck Germany

**Keywords:** genetic diversity, GWAS, H_2_O_2_, wheat

## Abstract

Hydrogen peroxide (H_2_O_2_) is a signaling molecule that plays a crucial role in plant growth and development against different abiotic stresses. Identifying genetic factors associated with H_2_O_2_ regulation and homeostasis can provide valuable insights for improving stress tolerance. Here, we explored genetic diversity of root and shoot traits mediated by H_2_O_2_ using a global diversity panel of 150 bread wheat cultivars. The H_2_O_2_ treatment significantly reduced root and shoot growth. We calculated relative values and stress tolerance index (STI) of root and shoot traits and performed genome‐wide association studies (GWAS). This led to identification of 108 marker–trait associations including the topmost associations on chromosomes 3B, 2A, 5A, 3B, 5D, 5A, 6B, 4B, and 3B for relative root length, STI root length, relative shoot length, STI shoot length, relative root fresh weight, relative shoot fresh weight, STI shoot fresh weight, and relative and STI root–shoot ratio, respectively. Linkage disequilibrium analysis revealed that major alleles of significant markers were linked with high relative values and STIs for all traits except for relative root length and relative root–shoot ratio. The selected candidate genes were involved mostly in metal ion binding, transmembrane transport, oxidation–reduction process, protein phosphorylation, DNA, and ADP binding processes. These findings provide a fundamental basis for functional analysis of putative candidate genes linked to H_2_O_2_‐mediated root–shoot growth of wheat. The result will also help to construct genetic map for H_2_O_2_‐mediated root–shoot growth variation.

## 1 Introduction

The life cycle of plants frequently encounters various biotic and abiotic stresses that impact growth and productivity. During stress, the plant's adjustment mechanisms can recognize and respond to external stimuli accordingly (Lamers et al. 2020). Hydrogen peroxide (H_2_O_2_) is a common reactive oxygen species produced under stresses. It plays an integral role in physiology necessary for the normal growth and development of plants (Deng et al. 2012; Liu et al. 2021). Additionally, H_2_O_2_ acts as a signaling molecule and mediates systemic signaling networks against different stress conditions (Mittler et al. 2022). Excessive formation of H_2_O_2_ is a common occurrence in several stresses, including mechanical injury, pathogen attack, extreme temperature, and drought (Bollag et al. 2020; Khedia et al. 2019; Dufková et al. 2019; Si et al. 2018). Different metabolic pathways within plant organelles are modulated by various stress conditions and could generate H_2_O_2_ during oxidative stress (Bhardwaj et al. 2021). These oxidative stresses have been documented to exhibit cross tolerance to the same and other different stresses in wheat, rice, tomatoes, and maize (Nazir et al. 2020; Khedia et al. 2019). Root and shoot play crucial roles in the growth and development of plants (Chen et al. 2022). The shoot, during the later stages of growth, is directly involved in photosynthesis and provides energy to the grain. Conversely, the root serves as the primary organ anchoring the plant to the soil for water and nutrient absorption. In cereals, quantitative trait loci (QTLs) for root traits have been mapped in maize ( *Zea mays* L.) (Kailiang et al. 2023), rice ( *Oryza sativa* L.) (Dinh et al. 2023), and sorghum (Bernardino et al. 2019). Barnaby et al. (2022) identified QTLs for root–shoot biomass at the tillering stage in rice, whereas Pace et al. (2015) detected loci for total root length in maize. Studies on loci associated with root and shoot traits in wheat are limited, especially in terms of loci for root–shoot traits under stress conditions. Recently, Fatima et al. (2021) identified osmotic stress‐mediated root‐linked loci and genes in wheat seedlings. Yang et al. (2021) reported root loci in the same crop mediated by low levels of phosphorus. Toxic levels of H_2_O_2_ reduce cell growth in roots and shoots compared to non‐treated controls (Voothuluru et al. 2020). Therefore, understanding H_2_O_2_‐mediated root–shoot growth could aid in mapping architectural traits associated with H_2_O_2_ regulation. Further, studying root–shoot growth under toxic levels of H_2_O_2_ facilitates identifying key genetic factors associated with various abiotic stresses.

Genome‐wide association studies (GWAS) are powerful genetic tools that have been utilized in recent years to dissect complex traits underlying development, metabolism, and stress tolerance (Horton et al. 2012; Yu et al. 2023; Kamruzzaman et al. 2023). GWAS followed by candidate gene search and gene ontology (GO) analyses pinpoint some promising candidate loci and genes (Daba et al. 2018). A recent GWAS pinpointed 36 candidate loci and a few promising candidate genes for H_2_O_2_ regulation in wheat diversity (Kamruzzaman et al. 2022). Sadhukhan et al. (2017) conducted a GWAS to identify loci and genes involved in tolerance pathways for root length of *Arabidopsis* seedlings mediated by toxic levels of H_2_O_2_. Another study demonstrated 1%–2% of genes being upregulated in *Arabidopsis* in response to H_2_O_2_ (Desikan et al. 2000). Vanderauwera et al. (2005) reported transcriptional upregulation of heat shock proteins through H_2_O_2_ imposition.

The assessment of relative growth derived from root–shoot traits under control and toxic doses of H_2_O_2_ treatments holds great significance. Relative value and stress tolerance index (STI) are popular approaches to evaluate the relative performance of a genotype under stress conditions for a specific trait, allowing for the control of background effects among different genotypes (Kan et al. 2015; Sukumaran et al. 2018). Relative value and STI have been reported under drought conditions to assess diversity in wheat populations, recently (Kamruzzaman et al. 2022, 2023). Utilizing GWAS with STI and relative growth values, followed by GO analyses, can help to uncover the genetic factors involved in H_2_O_2_‐mediated root–shoot growth in wheat seedlings.

Wheat is a leading economic crop worldwide, but due to the complexity of the genomic structure, the mechanisms of genetic regulation of adaptation processes are not well understood. Genome complexity has been derived from A, B, and D sub‐genomes that collectively possess vast genetic diversity (Walkowiak et al. 2020). Therefore, it would be interesting to combine the power of GWAS and wheat diversity to unveil genetic factors associated with H_2_O_2_. Genetic factors of H_2_O_2_ networks are potentially applicable to understand in‐depth H_2_O_2_ signaling pathways in crops. This could be implemented in stress breeding programs for other cereals, including rice and maize. The present work aimed to explore genetic variation and stress‐adaptive components in wheat diversity under H_2_O_2_ stress. The specific objectives were (a) to assess the phenotypic variations of root and shoot traits under H_2_O_2_ treatment, (b) to identify marker and haplotype alleles associated with root and shoot growth variations, and (c) to underpin the candidate loci and genes involved in H_2_O_2_‐mediated tolerance pathways.

## 2 Materials and Methods

### 2.1 Plant Materials, Growth Conditions, and Treatments

The study material consisted of a diversity panel of 150 winter bread wheat cultivars. The cultivars possess a broad genetic background, and the selection criteria followed economic and agronomic importance in Germany during their respective period of release from 1966 to 2016. Ninety‐two cultivars originated from Germany, while the others were from the United States, the United Kingdom, Mexico, France, Denmark, Serbian, Chile, Australia, and Ukraine. The cultivars are listed in Table S1, and a brief description of this population has also been provided in previous publications (Voss‐Fels et al. 2019; Begum et al. 2020). The experiment was conducted in a growth chamber (Bronson Climate, LW Zaltbommel, Netherlands) with a setup of a 16/8 h light/dark cycle, 22°C–23°C temperature, 55%–60% relative humidity, and a light intensity of 600 μmol m^−2^ s^−1^. Fifteen seeds per cultivar were surface‐sterilized with 5% sodium hypochlorite for 10 min, followed by washing five times with distilled water. The seeds were then placed into 9‐mm petri plates containing blotting paper (Whatman no. 2) and kept at room temperature for germination for 3 days. Uniformly germinated seeds were then placed into new petri plates with treatments either distilled water (control) or 20 mM of H_2_O_2_ (Sigma, USA). The 3‐day‐old seedlings were allowed to grow for the next 7 days. The treatment solutions were refreshed every 2 days. To determine the proper range of treatment for wheat, seeds were preliminarily treated with different doses of H_2_O_2_ (100, 200, and 500 μM and 20, 100 and 150 mM), along with a control (without H_2_O_2_) according to Sadhukhan et al. (2017). Based on these preliminary observations, 20 mM H_2_O_2_ was selected for the final experimental setup (Figure S1).

### 2.2 Root and Shoot Phenotyping

After exposure to 7 days, root length, shoot length, total seedling length, root–shoot ratio, root fresh weight, root dry weight, shoot fresh weight, and shoot dry weight were recorded from both treatments. A measuring tape was used to measure the root, shoot, and total length from *n* ≥ 9 individual seedlings in unit of millimeters (mm). All weights were recorded using a digital balance in milligram (mg). The weights of the dry samples were recorded by oven‐drying the fresh samples at 70°C for 72 h.

### 2.3 Phenotypic Data Analysis

Descriptive statistics, including the mean, maximum, minimum, and coefficient of variation (%), were performed through R statistical computing software (Version 3.6) (Table 1). For the analysis of variance (ANOVA), packages such as “nlme” and “emmeans” were utilized. A mixed linear model (MLM), two‐way ANOVA were applied for comparing the means of the traits, considering the effect of cultivar and replication as random effects and treatments as fixed effects (Kadam et al. 2017). The variance components were used for calculating broad‐sense heritability (H^2^). The following formula was used for estimating H^2^:

$$\text{Heritability}\;\left(H^{2}\right)=\frac{\mathrm{Vg}}{\mathrm{Vg}+\frac{\text{Verr}}{r}}$$

where Vg is the genotypic variance and Verr is the error variance and r is the number of replications.

**TABLE 1:** Descriptive statistics and analysis of variance based on average phenotypic values of root and shoot traits under control and H_2_O_2_ stress conditions.

Traits	Control					H_2_O_2_ stress					%R	Two‐way ANOVA		
	Max	Min	Mean	CV (%)	**H** ^2^	Max	Min	Mean	CV (%)	**H** ^2^		G	T	G × T
RL	155.92	82.33	113.69	11.52	0.96	134.50	42.78	79.86	18.29	0.93	29.8	***	***	***
SL	150.42	74.00	101.80	14.10	0.92	133.33	43.33	87.31	16.61	0.89	14.2	***	***	***
RSRatio	1.66	0.82	1.13	14.59	0.61	1.75	0.56	0.93	17.54	0.60	18.2	***	***	***
TL	294.33	169.91	215.49	10.55	0.68	260.33	112.26	167.17	15.46	0.68	22.4	***	***	NS
SFW	135.57	57.50	88.96	18.53	0.63	118.00	23.60	72.41	21.70	0.61	18.6	*	***	*
SDW	17.40	4.63	7.94	22.58	0.62	10.43	0.55	5.90	26.53	0.68	25.6	***	***	***
RFW	254.00	87.70	130.41	13.06	0.74	148.70	65.00	113.24	12.71	0.55	13.2	***	***	*
RDW	46.50	9.45	21.58	33.20	0.59	32.71	5.82	15.04	30.99	0.59	30.3	***	***	NS

Abbreviations: %R, % reduction compared to the control; CV, **H** ^**2**^ = broad‐sense heritability, coefficient of variation; G × T, genotype × treatment interaction; G, genotype; NS, nonsignificant; RDW, root dry weight; RFW, root fresh weight; RL, root length; SFW, shoot fresh weight; shoot dry weight; SL, shoot length; T, treatment; TL, total length.

* *p* < 0.05.

*** *p* < 0.0001.

We estimated relative value and STI. To calculate the relative value, mean values of root and shoot traits were used in the following formula:

$$\text{Relative value}=\frac{\text{value under stress conditions}}{\text{value under control conditions}}\;\left[\%\right]$$

Thus, relative root length, relative shoot length, relative shoot fresh weight, relative root fresh weight, and relative root–shoot ratio were recorded. Similarly, mean value of traits was used to calculate STI by using following formula:

$$\mathrm{STI}=\frac{\left(\mathrm{Yww}\right)\times \left(\mathrm{Ytd}\right)\;}{\left(\mathrm{Xww}\right)2}$$

where Y_ww_ = value of control treatment of a particular cultivar, Y_td_ = value of H_2_O_2_ treatment of a particular cultivar, and X_ww_ = mean value over all cultivars under control treatment (Fernandez 1992).

Thus, STI root length, STI shoot length, STI shoot fresh weight, and STI root–shoot ratio were recorded.

To estimate the relationship among the traits, Pearson's correlation coefficients (*r*) were calculated using the mean values of cultivars in the R program. A correlation table was created using “xtable” package with the “corstars” function. Additionally, the studied panel was categorized into two subgroups: cultivars registered before the year 2000 as “traditional” and cultivars registered on and after the year 2000 as “modern.” Mean comparisons between modern and traditional cultivars were performed using Student's *t*‐test in MS Office Excel 2013, assuming unequal variance for the traits relative shoot length, STI shoot length, relative root‐shoot ratio, and STI root–shoot ratio. Linkage disequilibrium (LD) decay patterns of all genomes (A, B, and D) were plotted in R (package, ggplot2).

### 2.4 GWAS

A 135K SNP chip representing 24,216 single nucleotide polymorphic (SNP) markers across 21 chromosomes was employed for GWAS. Details of these markers were described in Dadshani et al. (2021) and Voss‐Fels et al. (2019). Missing SNPs were excluded, and minor allele frequency (MAF) < 5% was filtered using Trait Analysis by Association, Evolution and Linkage (TASSEL) Version 5.2 (Money et al. 2015). GWAS was conducted by using TASSEL 5.2 with a compressed MLM that incorporated the population structures and five principal components. Kinship matrix (K) was used as an additive genetic effect, and the first five principal components were used as a fixed effect to correct for false positive associations due to population structure (Zhang et al. 2010). After the Bonferroni correction, only STI shoot length and STI root–shoot ratio passed the significant threshold. Therefore, a threshold *p* value of 0.001 [−log_10_(p) = 3] was set as a significant according to previous studies (Tadesse et al. 2015; Gao et al. 2016). This threshold level was employed for candidate gene search. Manhattan plots were visualized using the R package “CMplot.” The phenotypic variation (PV) explained by a marker (*r* ^2^) was calculated using TASSEL 5.2 (Bradbury et al. 2007).

### 2.5 Linkage Disequilibrium Analysis and Putative Candidate Gene Search

Linkage Disequilibrium (LD) block analysis was performed using Haploview 4.2 according to Barrett et al. (2005). It estimated linkage equilibrium by pairwise D′ values multiplied by 100 of each SNP combinations. The analyses included significant and nonsignificant adjacent SNPs on the same chromosomes. LD block was formed when the D′ values between SNPs displayed in a heat map with upper confidence bounds exceeded 0.98 and the lower bound exceeded 0.7 (Gabriel et al. 2002). LD blocks harboring significant SNPs were considered as loci, and genes in these loci were assembled. For significant SNPs not present in any LD block, a 1.0 Mbp window on either sides of chromosomes was scanned to search putative candidate genes following the approach of Begum et al. (2020). Student's *t*‐test assuming two‐sample unequal variances was conducted to compare the means of the traits for contrasting haplotypes. Annotation and GO were obtained from the WHEAT URGI database (https://wheat‐urgi.versailles.inra.fr) (Alaux et al. 2018). Significant SNPs associated with different relative and STI traits with chromosomal positions, *p* values, phenotypic variance (%), minor–major, and favorable alleles are listed in Tables 2a and 2b. The genes located within significant SNPs or in haplotype block regions were listed in Tables S2 and S3. Pleotropic markers or haplotype blocks were listed in Table 3. Further, candidate genes linked to top five significant SNPs (based on *p* value) were short‐listed and presented in Table 4. The decay rates of LD were determined for each genome separately by plotting LD (*r* ^2^) values obtained from TASSEL 5.2 against the distance (megabase pairs) between SNPs. A nonlinear regression function was used to fit the trend of LD decay according to Koua et al. (2022). The distance value for the regression line at *r* ^2^ = 0.1 gives the critical distance between markers up to which a QTL could extend.

**TABLE 3:** Pleiotropic markers located in different chromosomes and the corresponding traits.

Marker	Haplotype	Chr	Position (bp)	Traits	No. of genes
AX‐108852904	—	3B	822,891,332	STI_SL and STI_RSRatio	32
BS00071183_51			823,762,843		13
AX‐158598301			826,091,387		12
BS00073411_51			829,197,896		19
AX‐111015220	—		829,203,418		19
AX‐158578652			829,293,411		37
BS00065603_51		3D	611,497,215	STI_SL and STI_RSRatio	40
BS00068415_51		3D	612,903,461	STI_SL and STI_RSRatio	31
AX‐158582574	Com_Hap1	4B	456,188,724	R_RL and R_RSRatio	157
AX‐158600273	—	6A	520,581,021	R_RL and STI_SFW	9
AX‐158600281			520,712,811		2
AX‐111016876		2A	717,417,914	STI_RL and STI_RSRatio	40
AX‐108742509		2A	718,354,502		7

**TABLE 4:** Short‐list of putative candidate genes, their gene ontology, molecular functions, and biological processes linked with top five significant markers or marker‐established haplotype block for relative and STI of H_2_O_2_.

Trait	Marker/Haplotype block	Chr	Candidate gene	Short‐Description	Biological process	Molecular function
R_RL	AX‐109990240	3B	*TraesCS3B01G134400*	Zinc finger family protein	NA	Nucleic acid binding (GO:0003676)
	wsnp_JD_c2623_3541255	3B	*TraesCS3B01G132100*	bZIP transcription factor, putative (DUF1664)	NA	NA
	AX‐158600273	6A	*TraesCS6A01G287700*	Dof zinc finger protein	Regulation of transcription (GO:0006355)	DNA binding (GO:0003677)
	wsnp_Ku_c8497_14429303	7B	*TraesCS7B01G061900*	Receptor‐like kinase	Protein phosphorylation (GO:0006468)	Protein kinase activity (GO:0004672)
	AX‐158600281	6A	*TraesCS6A01G288300*	2‐Oxoglutarate (2OG) and Fe (II)‐dependent oxygenase superfamily protein	Oxidation–reduction process (GO:0055114)	Oxidoreductase activity (GO:0016491)
R_RL, R_RSRatio	Com_Hap1	4B	*TraesCS4B01G208800*	Pentatricopeptide repeat‐containing protein	NA	Protein binding (GO:0005515)
STI_RL	AX‐158596313	2A	*TraesCS2A01G265500*	Disease resistance protein (TIR‐NBS‐LRR class) family	NA	Nucleic acid binding (GO:0003676)
	AX‐158581925	4A	*TraesCS4A01G051600*	Protein transport protein GOT1	Vesicle‐mediated transport (GO:0016192)	NA
	wsnp_JD_c52_87219	2B	*TraesCS2B01G448300*	Receptor‐like kinase	Protein phosphorylation (GO:0006468)	Protein kinase activity (GO:0004672)
	sti_RL_2B_Hap1	2B	*TraesCS4B01G201600*	Receptor‐like kinase, putative	Protein phosphorylation (GO:0006468)	Protein kinase activity (GO:0004672)
STI_RL, STI_RSRatio	AX‐111016876	2A	*TraesCS2A01G477200*	NBS‐LRR disease resistance protein	NA	ADP binding (GO:0043531)
R_SL	AX‐109884177	5A	*TraesCS5A01G043000*	Heavy metal transport/detoxification superfamily protein, putative	Metal ion transport (GO:0030001)	Metal ion binding (GO:0046872)
	Rel_SL_1B_Hap1	1B	*TraesCS1B01G456400*	S‐type anion channel	NA	Transmembrane transport (GO:0055085)
STI_SL, STI_RSRatio	AX‐111015220	3B	*TraesCS3B01G611100*	Receptor‐like protein kinase	Protein phosphorylation (GO:0006468)	Protein kinase activity (GO:0004672)
STI_SL	BS00068415_51	3D	*TraesCS3D01G541600*	Disease resistance protein (NBS‐LRR class) family	NA	ADP binding (GO:0043531)
	AX‐158598301	3B	*TraesCS3B01G608500*	Aquaporin	Transport (GO:0006810)	Transporter activity (GO:0005215)
STI_SL, STI_RSRatio	BS00071183_51	3B	*TraesCS3B01G604800*	NBS‐LRR‐like resistance protein	NA	ADP binding (GO:0043531)
	BS00065603_51	3D	*TraesCS3D01G540900*	Aquaporin	Transporter activity (GO:0005215)	Transport (GO:0006810)
R_RSRatio	IAAV4343	3A	*TraesCS3A01G237800*	Protein phosphatase 2c, putative		catalytic activity (GO:0003824)
	AX‐158523668	3A	*TraesCS3A01G238300*	Ethylene‐responsive transcription factor, putative	Regulation of transcription, DNA‐templated (GO:0006355)	DNA binding (GO:0003677)
	AX‐158548980	3D	*TraesCS3D01G421900*	Disease resistance protein RPM1	NA	ADP binding (GO:0043531)
STI_RSRatio, STI_SL	AX‐158578652	3B	*TraesCS3B01G609400*	Cytochrome P450	Oxidation–reduction process (GO:0055114)	Oxidoreductase activity (GO:0016705)
STI_RSRatio	AX‐158598301	3B	*TraesCS3B01G608500*	Aquaporin	Transport (GO:0006810)	Transporter activity (GO:0005215)
	AX‐108742509	2A	*TraesCS2A01G480300*	Thioredoxin	Cell redox homeostasis (GO:0045454)	NA
R_RFW	BS00065783_51	5D	*TraesCS5D01G071300*	FAD/NAD(P) binding oxidoreductase family protein	Oxidation–reduction process (GO:0055114)	Oxidoreductase activity (GO:0016491)
	AX‐158528874	6B	*TraesCS6B01G473100*	F‐box protein family	NA	NA
	Tdurum_contig82473_67	5B	*TraesCS5B01G449200*	Receptor‐like protein kinase	Protein phosphorylation (GO:0006468)	ATP binding (GO:0005524)
	RAC875_c63067_283	1B	*TraesCS1B01G093500*	Pentatricopeptide repeat‐containing protein	NA	protein binding (GO:0005515)
R_SFW	BS00074299_51	5A	*TraesCS5A01G380800*	DNA repair protein XRCC4	DNA recombination (GO:0006310)	DNA binding (GO:0003677)
	BS00076246_51	5A	*TraesCS5A01G380800*	DNA repair protein XRCC4	DNA recombination (GO:0006310)	DNA binding (GO:0003677)
	Tdurum_contig86202_175	5A	*TraesCS5A01G380700*	Disease resistance protein (NBS‐LRR class) family		ADP binding (GO:0043531)
	AX‐108742709	5A	*TraesCS5A01G381100*	Cytochrome P450 family protein, expressed	Oxidation–reduction process (GO:0055114)	Oxidoreductase activity, acting on paired donors, with incorporation or reduction of molecular oxygen (GO:0016705)
R_SFW	AX‐89769139	5A	*TraesCS5A01G380700*	Disease resistance protein (NBS‐LRR class) family	NA	ADP binding (GO:0043531)
STI_SFW	AX‐158535753	6B	*TraesCS6B01G074100*	Cytochrome P450 family protein	Oxidation–reduction process (GO:0055114)	Oxidoreductase activity, acting on paired donors, with incorporation or reduction of molecular oxygen (GO:0016705)
	Excalibur_c54055_694	1D	*TraesCS1D01G219800*	bZIP transcription factor (DUF630 and DUF632)	NA	NA
	AX‐158586104	5B	*TraesCS5B01G382000*	F‐box/LRR‐repeat protein 17	NA	Protein binding (GO:0005515)
	wsnp_Ku_c4296_7807837	6A	*TraesCS6A01G288900*	BZIP transcription factor	Transcription factor activity, sequence‐specific DNA binding (GO:0003700)	Regulation of transcription, DNA‐templated (GO:0006355)

Abbreviations: R_RL, relative root length; R_RSRatio, relative root‐shoot ratio; R_SFW, relative shoot fresh weight; STI_RL, stress tolerance index root length; STI_RSRatio, stress tolerance index root–shoot ratio; STI_SFW, stress tolerance index shoot fresh weight; STI_SL, stress tolerance index shoot length.

## 3 Results

### 3.1 H_2_O_2_ Application Significantly Reduced Root and Shoot Growth in Wheat

To assess the impact of H_2_O_2_ treatment on root and shoot growth, eight traits (root length, shoot length, root–shoot ratio, total length, root fresh weight, shoot fresh weight, root dry weight, and shoot dry weight) were measured. It was observed that all traits were affected by the H_2_O_2_ treatment (Table 1). Significant effect of genotype (cultivar), treatment, and genotype–treatment interactions were observed for most traits, except for total length and root dry weight, where the interaction effect was nonsignificant (Table 1). H_2_O_2_ stress reduced root length by 29.8%, shoot length by 14.2%, root shoot ratio by 18.2%, total length by 22.4%, shoot fresh weight by 18.6%, shoot dry weight by 25.6%, root fresh weight by 13.2%, and root dry weight by 30.3% (Table 1). These results indicate that H_2_O_2_ stress had a strong negative impact on root and shoot growth. Importantly, a wider genetic diversity of cultivars was observed under H_2_O_2_ treatment, except for root fresh weight and root dry weight.

Pearson's correlation revealed varying degrees of correlations among the traits under H_2_O_2_ treatment (Table S4). High positive associations (*r* = 0.34–0.89; *p* < 0.05) were observed among root length, shoot length, total length, shoot fresh weight, shoot dry weight, and root fresh weight under this treatment. However, shoot length, shoot fresh weight, and shoot dry weight showed a negative correlations with root‐shoot ratio (*r* = −0.07 to −0.35). Root dry weight exhibited weak but negative associations with other traits, including root length, shoot length, total length, shoot fresh weight, and shoot dry weight (*r* = −0.13 to −0.24). Root length displayed high positive correlations with shoot length, root–shoot ratio, total length, shoot fresh weight, shoot dry weight, and root fresh weight under H_2_O_2_ treatment (*r* = 0.27–0.89) (Table S4).

### 3.2 High STI and Relative Root and Shoot Growth Were Linked With Traditional Subgroup

**FIGURE 1:**
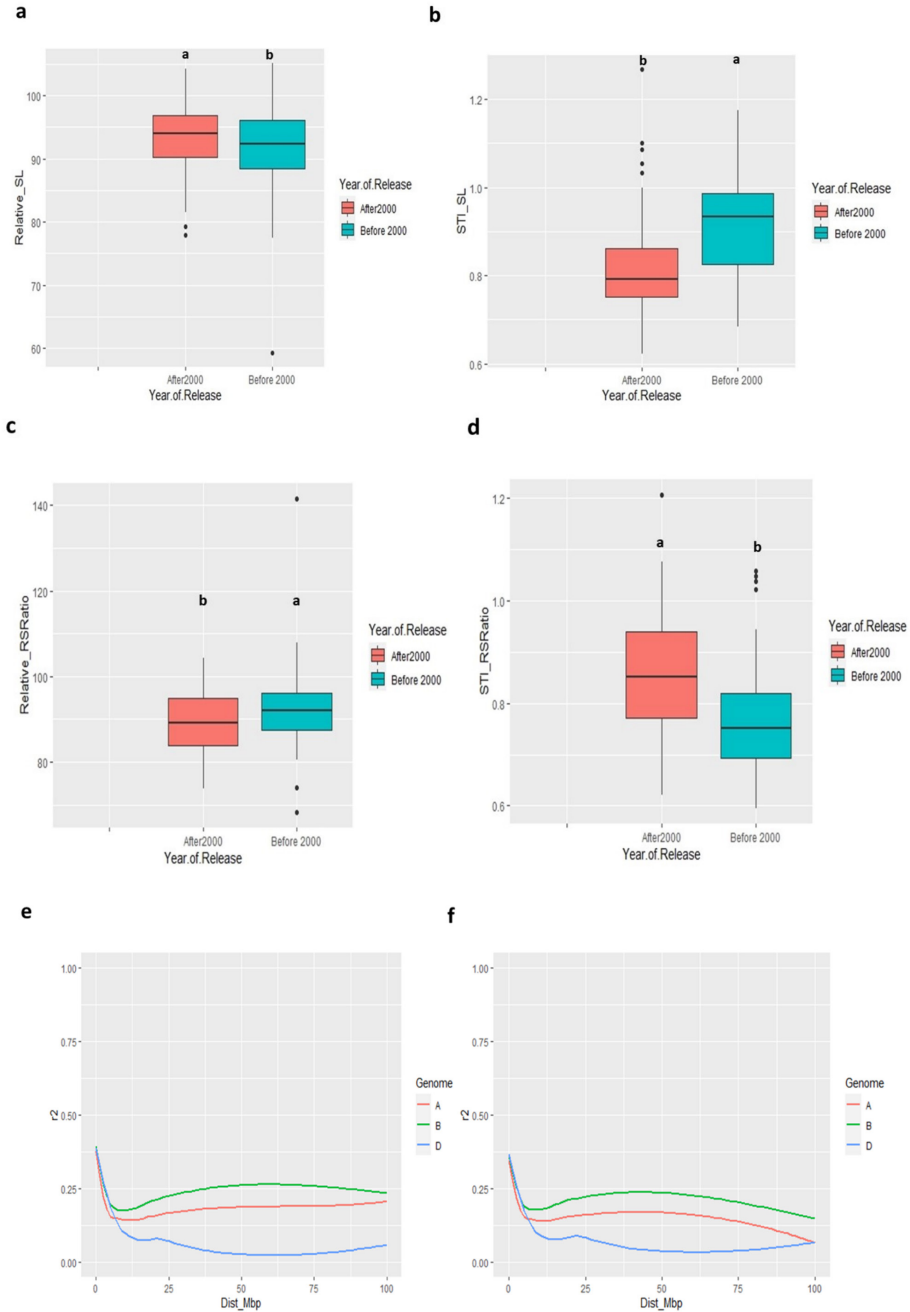
Performance of wheat cultivars registered on and after the year 2000 (modern) and before (traditional). (a) Relative shoot length. (b) STI shoot length. (c) Relative root–shoot ratio. (d) STI root–shoot ratio. (e,f) The LD decay pattern of studied cultivars of bread wheat across three genomes A, B, and D. (e) The LD decay pattern of modern cultivars. (f) The LD decay pattern of traditional cultivars. In (a–d), different letters indicate statistical difference at *p* < 0.05.

We assessed H_2_O_2_‐mediated root and shoot growth of modern and traditional cultivar subgroups to observe if there is any year effect on cultivars. The analyses included relative shoot length (Figure 1a), STI shoot length (Figure 1b), relative root–shoot ratio (Figure 1c), and STI root–shoot ratio (Figure 1d). The results showed distinct relative and STI of traits between those two subgroups. It was observed that traditional cultivars had significantly higher (*p* < 0.05) STI shoot length, relative root–shoot ratio, and STI root–shoot ratio values than the modern group, except for relative shoot length, which showed no significant difference between these two subgroups. LD decay plots exhibited a distinguished pattern of LD decay between modern and traditional cultivars (Figure 1e,f). The analyses found that the modern cultivar group with the preferable allele exhibited longer shoot and root length in response to H_2_O_2_ treatment compared to the traditional cultivar group (Tables S5a and S5b).

### 3.3 GWAS Uncovered the Candidate QTLs for H_2_O_2_

GWAS were conducted to identify QTL regions of root and shoot trait diversity mediated by H_2_O_2_. GWAS utilized the STI and relative values representing the relative reduction percentage of root and shoot traits due to exogenous H_2_O_2_ treatment. The compressed mixed linear model (cMLM) was employed, incorporating the population structure and kinship matrix as per Larsson et al. (2013). Marker–trait associations (MTAs) that met the threshold level of *p* = 0.001 were considered significant for investigating candidate genes to relative and STI root–shoot traits. A total of 108 significant SNPs were identified across the wheat genomes (Tables 2a, 2b, and S2). The LD analysis using Haploview 4.2 identified LD blocks containing 17 SNPs those encompassed a total of 277 protein‐coding genes (Table S2). Thirty‐seven of these genes are short‐listed in the Table 4.

**TABLE 2a:** Significant SNPs associated with different STI root–shoot traits in bread wheat and corresponding marker position, *p* value (*p* ≤ 0.001), phenotypic variance (%), major, minor (Ma: Mi), and favorable (Fav.) alleles.

Traits	Marker	Chr	Position (bp)	*p*	Amount of variance (%)	Allele (Ma: Mi)	Fav. allele
STI_RL	AX‐158596313	2A	421,064,707	1.66E‐04	10.25	C:T	T
	AX‐111016876	2A	717,417,914	4.14E‐04	9.07	T:C	T
	AX‐108742509	2A	718,354,502	9.03E‐04	8.05	A:G	A
	AX‐158581925	4A	43,031,174	3.56E‐04	9.26	C:T	C
	AX‐110016919	5B	591,062,022	8.12E‐04	8.18	C:A	C
	D_contig78519_72	7D	10,724,734	9.87E‐04	7.81	C:A	C
STI_RSRatio	RAC875_c21906_247	2A	691,478,630	2.60E‐04	8.77	G:A	A
	AX‐158555653	2A	691,479,513	2.17E‐04	8.91	G:A	A
	AX‐158555652	2A	691,845,720	2.43E‐04	9.08	C:T	T
	RAC875_c52458_454	2A	692,755,001	9.38E‐04	7.13	C:T	T
	BobWhite_c17403_635	2A	693,241,897	9.38E‐04	7.13	T:C	C
	AX‐158572604	2A	706,575,588	7.03E‐04	7.53	G:A	G
	AX‐158572603	2A	706,757,509	7.45E‐04	7.49	A:G	A
	AX‐111016876	2A	717,417,914	8.86E‐05	10.58	T:C	T
	AX‐158557238	2A	717,418,634	4.86E‐04	8.28	T:C	T
	AX‐109854150	2A	717,857,707	3.50E‐04	8.77	G:T	G
	AX‐108742509	2A	718,354,502	1.05E‐04	10	A:G	A
	AX‐158608713	2A	718,358,893	3.40E‐04	8.46	C:T	C
	BS00093201_51	2A	718,571,577	9.76E‐04	7.28	A:G	A
	AX‐110966497	2A	718,721,301	1.31E‐04	9.7	C:T	C
	BS00081506_51	2A	718,730,480	4.28E‐04	8.08	C:T	C
	AX‐109340451	2A	718,934,808	3.05E‐04	8.68	A:G	A
	Kukri_c51247_322	3A	140,043,493	5.21E‐04	7.84	C:T	C
	AX‐108852904	3B	822,891,332	2.64E‐04	8.82	G:A	G
	BS00071183_51	3B	823,762,843	6.00E‐04	7.82	A:G	A
	AX‐158598301	3B	826,091,387	7.38E‐05	10.31	G:A	A
	BS00073411_51	3B	829,197,896	7.51E‐04	7.54	T:G	T
	AX‐111015220	3B	829,203,418	1.25E‐05	12.92	T:C	T
	AX‐158578652	3B	829,293,411	4.76E‐05	11.05	T:G	T
	BS00065603_51	3D	611,497,215	6.00E‐04	7.82	C:T	C
	BS00068415_51	3D	612,903,461	6.63E‐04	7.69	G:A	G
	AX‐158617434	4A	18,119,033	7.38E‐04	7.53	G:A	G
	Kukri_rep_c68594_530	4D	12,773,159	7.77E‐04	7.37	A:G	G
STI_SFW	AX‐158600273	6A	520,581,021	8.94E‐04	7.98	C:T	C
	AX‐158600281	6A	520,712,811	8.68E‐04	8.02	T:G	T
	wsnp_Ku_c4296_7807837	6A	520,717,537	8.12E‐04	8.12	T:C	T
	Excalibur_c54055_694	1D	308,455,842	5.33E‐04	8.78	C:T	C
	AX‐158535753	6B	51,225,226	1.83E‐04	10.43	A:G	G
STI_SL	AX‐108852904	3B	822,891,332	3.77E‐05	12.87	G:A	G
	BS00071183_51	3B	823,762,843	1.94E‐05	13.81	A:G	A
	IAAV8659	3B	826,081,626	1.15E‐04	11.07	C:A	C
	wsnp_Ra_rep_c75740_73183118	3B	826,081,626	1.15E‐04	11.07	G:T	G
	AX‐158598301	3B	826,091,387	1.68E‐05	13.86	G:A	A
	BS00073411_51	3B	829,197,896	8.64E‐05	11.58	T:G	T
	AX‐111015220	3B	829,203,418	7.40E‐06	15.49	T:C	T
	AX‐158578652	3B	829,293,411	4.15E‐05	12.66	T:G	T
	BS00065603_51	3D	611,497,215	1.94E‐05	13.81	C:T	C
	BS00068415_51	3D	612,903,461	1.57E‐05	14.13	G:A	G

Abbreviations: STI_RL, stress tolerance index root length; STI_RSRatio, stress tolerance index root–shoot ratio; STI_SFW, stress tolerance index shoot fresh weight; STI_SL, stress tolerance index shoot length.

**TABLE 2b:** Significant SNPs associated with different relative root‐shoot traits in bread wheat and marker position, *p* value (*p* ≤ 0.001), phenotypic variance (%), major, minor (Ma: Mi), and favorable alleles.

Traits	Marker	Chr	Position (bp)	*p*	Amount of variance (%)	Allele (Ma: Mi)	Fav. allele
R_RFW	Kukri_c67721_184	1A	21,282,106	7.42E‐04	9.04	G:A	G
	BS00087787_51	1B	50,778,549	5.23E‐04	9.85	C:T	C
	BS00084305_51	1B	90,992,706	6.26E‐04	9.89	G:T	G
	RAC875_c63067_283	1B	95,654,954	2.92E‐04	10.55	T:C	T
	AX‐158607168	1B	107,000,000	2.98E‐04	10.71	G:A	G
	AX‐158570694	1B	116,000,000	5.59E‐04	9.66	T:C	T
	Kukri_c29582_126	1B	119,000,000	2.92E‐04	10.55	C:T	C
	AX‐158606938	1B	223,000,000	3.98E‐04	10.02	G:T	G
	Kukri_rep_c105316_262	1B	231,000,000	3.46E‐04	10.28	T:G	T
	IAAV902	3A	574,000,000	5.29E‐04	9.66	G:A	G
	AX‐158603951	4A	535,000,000	7.05E‐04	9.93	G:A	G
	Tdurum_contig82473_67	5B	621,000,000	2.66E‐04	10.65	G:A	A
	BS00065783_51	5D	69,456,300	8.02E‐05	12.56	T:C	T
	Excalibur_c28759_914	6B	716,000,000	2.96E‐04	10.97	A:G	A
	AX‐158626906	7B	705,000,000	6.37E‐04	9.25	T:C	T
R_RL	AX‐89555340	1A	592,000,000	9.70E‐04	7.65	T:C	C
	wsnp_JD_c2623_3541255	3B	115,000,000	4.89E‐04	8.53	G:T	T
	AX‐109990240	3B	116,000,000	4.38E‐04	8.88	C:T	C
	AX‐158600273	6A	521,000,000	7.47E‐04	7.93	C:T	T
	AX‐158600281	6A	521,000,000	8.19E‐04	7.81	T:G	G
	wsnp_Ku_c8497_14429303	7B	64,728,963	8.13E‐04	7.89	G:A	A
R_RSRatio	Kukri_c29170_680	2A	693,000,000	6.94E‐04	8.08	A:G	G
	AX‐158533093	3A	444,000,000	7.36E‐04	8.55	A:G	A
	AX‐158523668	3A	445,000,000	2.37E‐04	9.91	C:T	T
	AX‐158523313	3A	462,000,000	5.05E‐04	8.52	C:T	C
	AX‐158533132	3A	478,000,000	6.31E‐04	8.26	G:A	A
	AX‐158548980	3D	534,000,000	3.85E‐04	8.95	T:G	G
	AX‐158582575	4B	510,000,000	1.66E‐04	10.24	A:G	G
R_SFW	AX‐110382510	5A	578,000,000	5.81E‐04	10.57	A:G	A
	AX‐108744896	5A	578,000,000	5.81E‐04	10.57	A:C	A
	Ku_c19858_2078	5A	578,000,000	5.81E‐04	10.57	T:C	T
	AX‐89769139	5A	578,000,000	3.86E‐04	11.35	A:G	A
	BS00076246_51	5A	578,000,000	1.01E‐04	13.78	T:C	T
	BS00074299_51	5A	578,000,000	1.01E‐04	13.78	T:C	T
	Tdurum_contig86202_175	5A	578,000,000	1.01E‐04	13.78	G:A	G
	AX‐108742709	5A	578,000,000	1.01E‐04	13.78	A:C	A
	AX‐158589824	6D	3,075,685	9.60E‐04	9.64	G:A	G
R_SL	AX‐158521438	1B	6,867,170	6.38E‐04	8.27	C:G	C
	AX‐158532334	2D	535,000,000	5.85E‐04	8.4	A:G	A
	AX‐158521912	2D	542,000,000	8.63E‐04	7.85	C:T	C
	AX‐158582483	4B	661,000,000	9.65E‐04	7.77	G:A	G
	AX‐109884177	5A	37,843,077	3.16E‐04	9.24	T:G	G
	AX‐158544315	7D	87,479,166	7.72E‐04	8.04	G:A	G

Abbreviations: R_RFW, relative root fresh weight; R_RL, relative root length; R_RSRatio, relative root and shoot ratio; R_SFW, relative shoot fresh weight; R_SL, relative shoot length.

### 3.4 GWAS Identified Candidate Loci for Root Length Variations

**FIGURE 2:**
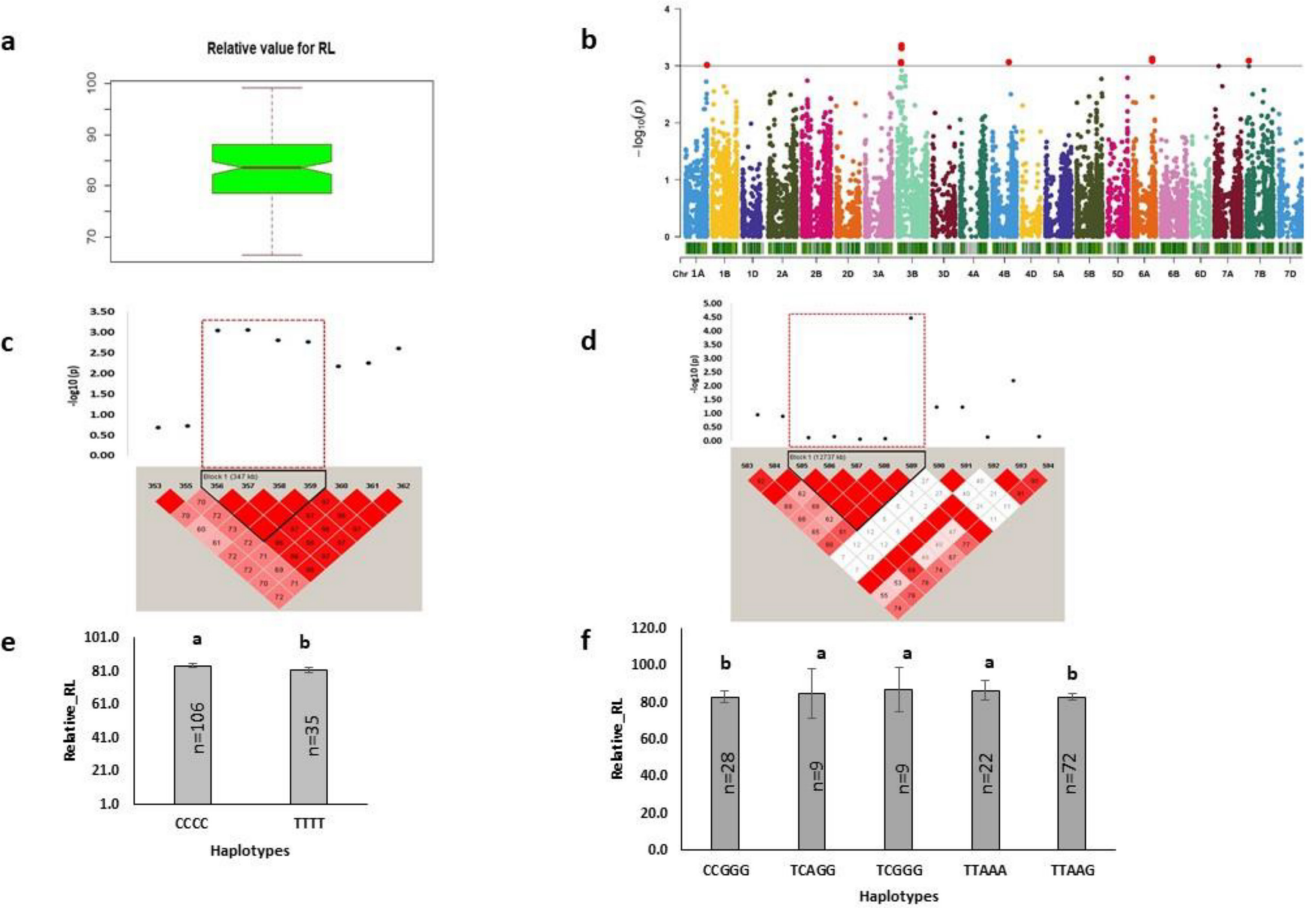
Marker–trait associations for relative root length. (a) The box plot shows the distribution of relative values of root length. (b) The Manhattan plot displays the marker–trait associations; the horizontal red line indicates threshold level (*p* < 0.001); the dots above this line indicate significant markers. (c) The linkage disequilibrium (LD) heat map illuminates the peak region on chromosome 3B (Rel_RL_3B_Hap1). (d) The LD heat map illuminates the peak region on chromosome 4B (Com_Hap1). In (c,d), the pairwise LD map between SNP markers is marked by D′ values, dark red represents 1, whereas white is for 0. The region surrounded by the red dotted line indicates LD block that contains significant SNPs. (e,f) Phenotypic comparison of the haplotype groups established for the significant SNPs, as detected by LD block. Different letters indicate statistical difference at *p* < 0.05; *n* indicates the number of genotypes representing each specific haplotype.

**FIGURE 3:**
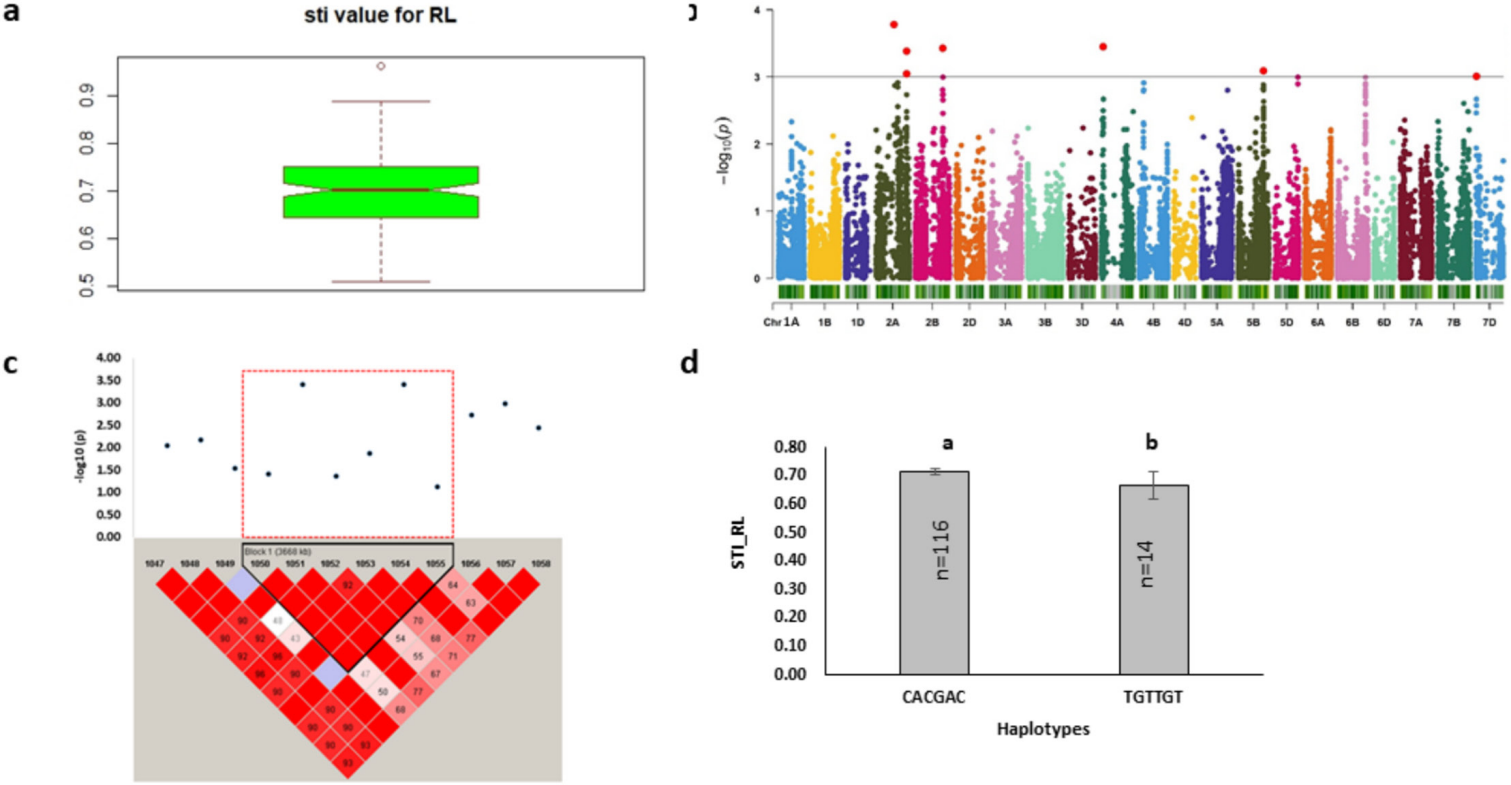
Marker–trait associations for STI root length. (a) The box plot shows the distribution of STI root length; (b) The Manhattan plot displays the marker–trait associations; the horizontal red line indicates threshold level (*p* < 0.001); the dots above this line indicate significant markers. (c) The linkage disequilibrium (LD) heat map illuminates the peak region on chromosome 2B (sti_RL_2B_Hap1). In (c), the pairwise LD map between SNP markers is marked by D′ values, dark red represents 1, whereas white is for 0. The region surrounded by the red dotted line indicates an LD block that contains significant SNPs. (d) Phenotypic comparison of the haplotype groups established for the significant SNPs, as detected by LD block. Different letters indicate statistical difference at *p* < 0.05; *n* indicates the number of genotypes representing each specific haplotype.

Root length mediated by H_2_O_2_ treatment exhibited broad phenotypic variation, as represented by relative and STI root length (Figures 2a and 3a). Significant MTAs were identified on 1A, 2A, 2B, 3B, 4A, 4B, 5B, 6A, 7B, and 7D, encompassing a total of 17 SNP markers (Figures 2b and 3b). Among them, STI and relative root length were associated with 8 and 9 markers, respectively. These markers showed a phenotypic variation ranging from 7.65% to 8.88% for relative root length and 7.81% to 10.25% for STI root length (Tables 2a and 2b). Regarding the 9 markers for relative root length, two markers were found to establish the haplotype block, Rel_RL_3B_Hap1 on 3B chromosome (Figure 2c,e), and another marker formed Com_Hap1on 4B (Figure 2d,f). The major allele of significant marker for Rel_RL_3B_Hap1 and the minor allele for Com_Hap1 exhibited higher relative root length (Figure 1e,f). Single‐marker analysis revealed that 83% of minor alleles were linked to significantly higher relative root length. In total, 255 protein‐coding genes were associated with relative root length (Table S2). Chromosome 3B emerged as the hotspot region for MTA. Top five MTAs and promising candidate genes are listed in Table 4. The top most marker, AX‐109990240 (*p* = 4.38E‐04), was situated on chromosome 3B (Figure 2b and Table 2b). The linked gene encoded a zinc finger protein with nucleic acid binding activity (GO: 0003676). Notably, within the top 5 MTAs, the haplotype Com_Hap1 was identified as pleiotropic, overlapped with the trait relative root‐shoot ratio (Table 3). The genes associated with the remaining four candidate SNPs included *TraesCS3B01G132100* (bZIP transcription factor), *TraesCS7B01G061900* (receptor‐like kinase [RLK]), *TraesCS4B01G208800* (pentatricopeptide repeat‐containing protein) were involved in oxidation–reduction, protein phosphorylation, and regulation of transcription functions (Table 4).

Candidate loci for STI root length were located on chromosomes 2A, 2B, 4A, 5B, and 7D, with AX‐158596313 as the top marker situated on 2A. The gene associated with the topmost marker encoded a disease resistance protein involved in nucleic acid binding function (GO: 0003676). The major allele of single markers (> 80%) was associated with higher STI root length (Table 2a). Similarly, the major allele of the haplotype sti_RL_2B_Hap1 demonstrated significantly higher STI root length compared to the minor allele (Figure 3c,d). The haplotype block region coincided with the candidate gene, *TraesCS4B01G201600*, encoding a RLK protein (Table 4). The candidate loci comprised a total of 172 protein‐coding genes for STI root length (Table S3). The top 5 significant MTAs were associated with stress‐related pathways, including protein phosphorylation, protein kinase, and ADP binding functions. Notably, other significant markers located on 2A, 7D, and 5B chromosomes contained BS‐LRR disease resistance protein‐coding genes involved in ADP binding function (Table S3).

### 3.5 GWAS Identified Candidate Loci for Shoot Length Variation

**FIGURE 4:**
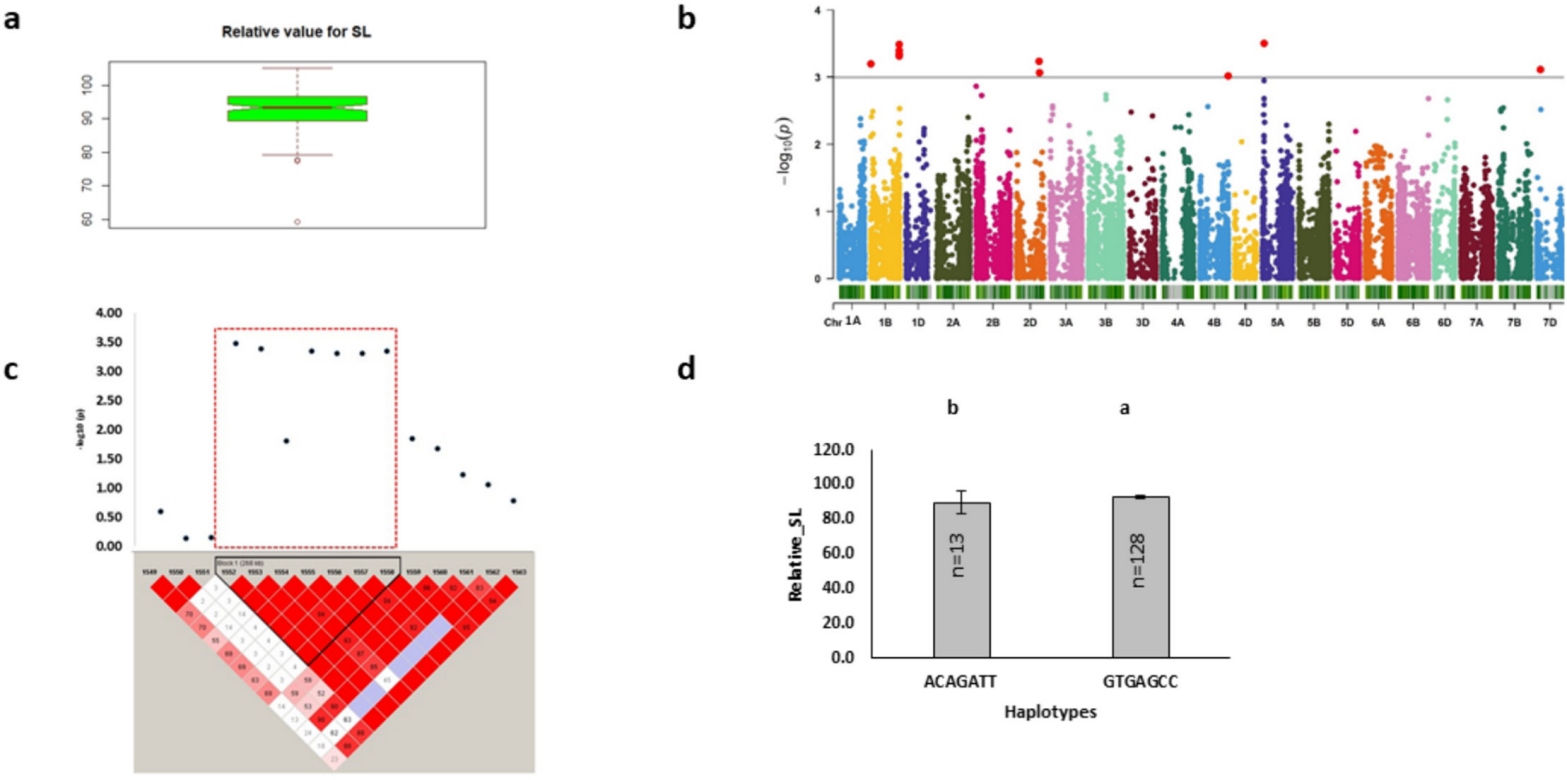
Marker–trait associations for relative shoot length. (a) The box plot shows the distribution of relative shoot length. (b) The Manhattan plot displaying the marker‐trait associations; the horizontal red line indicates threshold level (*p* < 0.001); the dots above this line indicate significant markers. (c) The linkage disequilibrium (LD) heat map illuminates the peak region on chromosome 1B (Rel_SL_1B_Hap1). In (c), the pairwise LD map between SNP markers is marked by D′ values, dark red represents 1, whereas white is for 0. The region surrounded by the red dotted line indicates an LD block that contains significant SNPs. (d) Phenotypic comparison of the haplotype groups established for the significant SNPs, as detected by LD block. Different letters indicate statistical difference at *p* < 0.05; *n* indicates the number of genotypes representing each specific haplotype.

**FIGURE 5:**
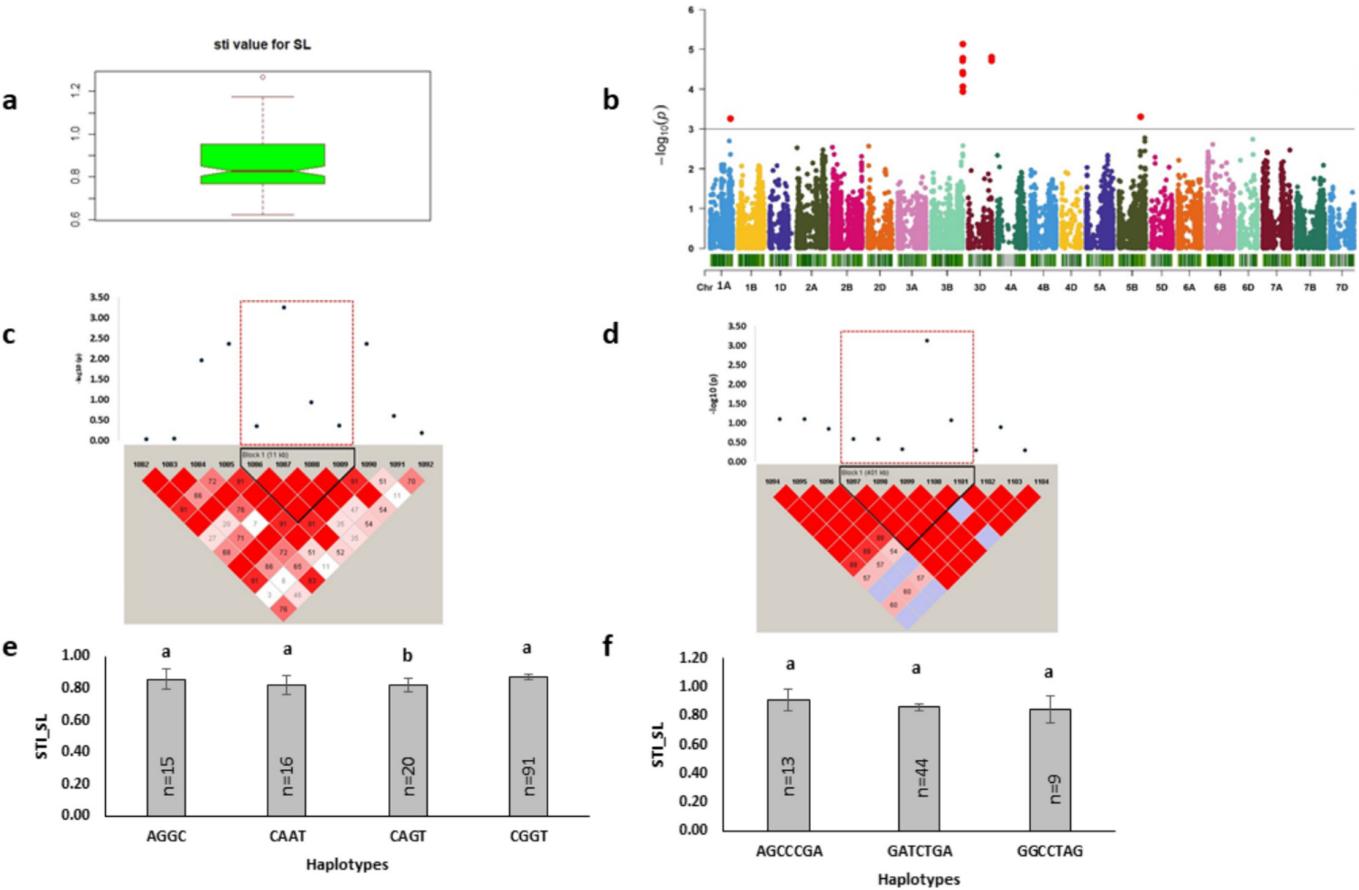
Marker–trait associations for STI shoot length. (a) The box plot shows the distribution of STI shoot length; (b) The Manhattan plot displaying the marker–trait associations; the horizontal red line indicates threshold level (*p* < 0.001); the dots above this line indicate significant markers. (c) The linkage disequilibrium (LD) heat map illuminates the peak region on chromosome 1A (sti_SL_1A_Hap1). (d) The LD heat map illuminates the peak region on chromosome 5B (sti_SL_5B_Hap1). In (c,d), the pairwise LD map between SNP markers is marked by D′ values, dark red represents 1, whereas white is for 0. The region surrounded by the red dotted line indicates an LD block that contains significant SNPs. (e,f) Phenotypic comparison of the haplotype groups established for the significant SNPs, as detected by LD block. Different letters indicate statistical difference at *p* < 0.05; *n* indicates the number of genotypes representing each specific haplotype.

Relative and STI shoot length, representing shoot length, showed a greater phenotypic variation (Figures 4a and 5a). This variation was associated with a total of 23 significant markers distributed across chromosomes 1A, 1B, 2D, 3B, 3D, 4B, 5A, 5B, and 7D, and was controlled by 352 protein‐coding genes (Figures 4b and 5b; Tables 3a and 3b). Among these, 9 and 14 markers showing phenotypic variation ranging between 7.77% to 9.24% and 11.7% to 15.49% were associated with relative shoot length and STI shoot length, respectively (Tables 2a and 2b). Chromosome 1B was identified as the hotspot region accumulating the highest number of significant markers for relative shoot length. The haplotype on it was linked to 3 protein‐coding genes. The major allele of this haplotype and 83% major alleles of single markers showed higher relative shoot length (Figure 4c,d). The top significant marker, AX‐109884177 [*p* = 3.16E‐04] located on chromosome 5A, was associated with the gene *TraesCS5A01G043000*, which is involved in metal ion binding function. Another haplotype, Rel_SL_1B_Hap1, was linked with the gene *TraesCS1B01G456400*, which functioned in transmembrane transport (Table 4).

The topmost significant marker for STI shoot length was identified as AX‐111015220 (*p* = 7.40E‐06) on chromosome 3B, where the associated gene exhibited protein phosphorylation activity (GO: 0006468) (Table 4). Two haplotype blocks, sti_SL_1A_Hap1 and sti_SL_5B_Hap1, located on chromosomes 1A and 5B, encompassed 2 and 15 protein‐coding genes, respectively (Figure 5c,d). Additionally, pleotropic markers BS00065603_51 and BS00068415_51 located on chromosome 3D overlapped both STI shoot length and STI root–shoot ratio (Table 3). The single‐marker analysis indicated over 90% of major alleles contributed to higher STI shoot length (Table 2a). Top 5 candidate SNP loci included receptor‐like protein kinase, disease resistance protein, aquaporin, and NBS‐LRR‐like resistance protein‐coding genes (Table 4). These genes were involved in stress‐related biological processes such as protein phosphorylation, transport, and transporter activity.

### 3.6 GWAS Identified Candidate Loci for Root Fresh Weight Variations

**FIGURE 6:**
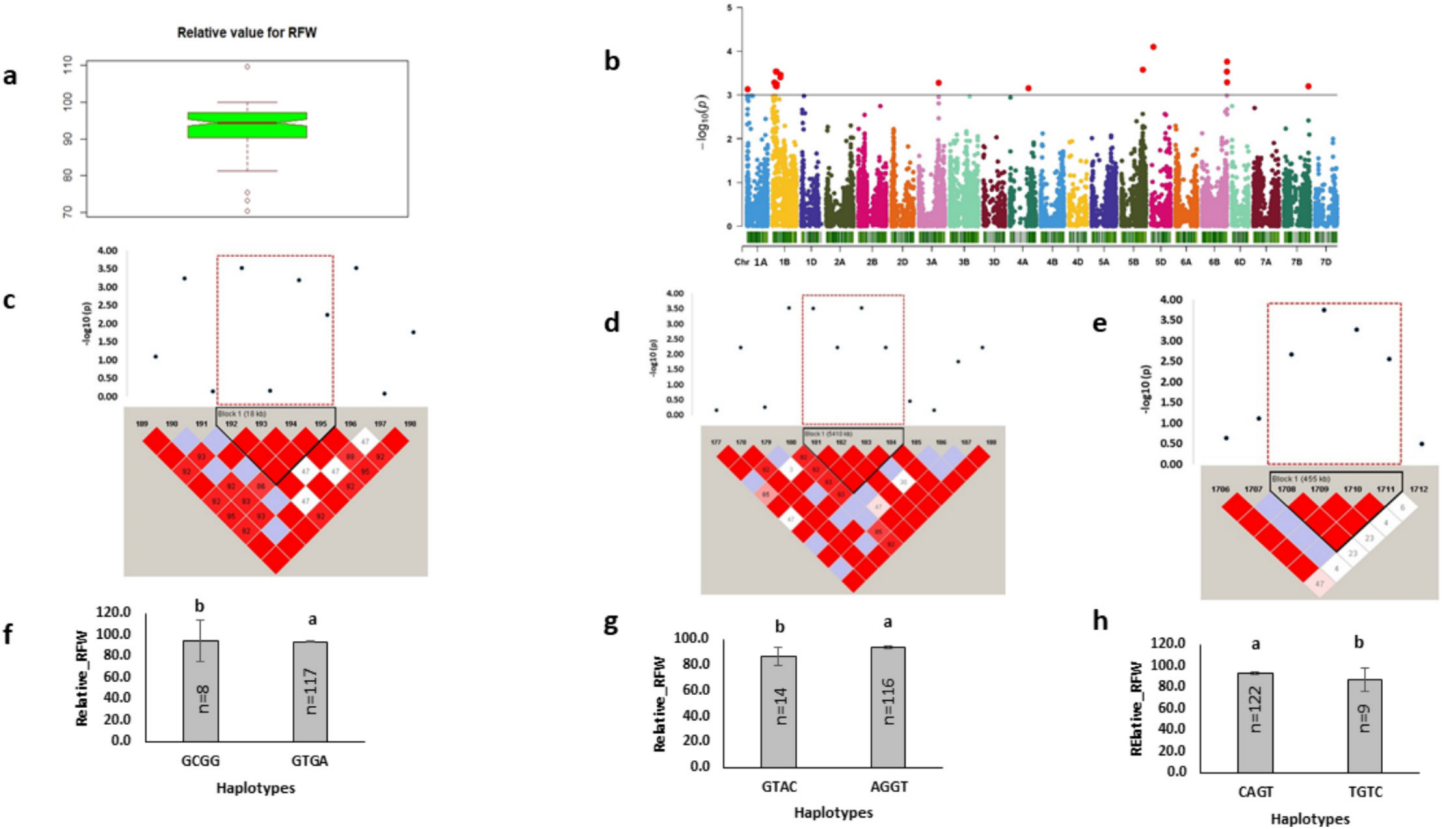
Marker–trait associations for relative root fresh weight. (a) The box plot shows the distribution of relative root fresh weight. (b) The Manhattan plot displays the marker‐trait associations; the horizontal red line indicates threshold level (*p* < 0.001); the dots above this line indicate significant markers. (c,d) The linkage disequilibrium (LD) heat maps illuminate the peak region on chromosome 1B (Rel_RFW_1B_Hap1 and Rel_RFW_1B_Hap2). (e) The LD heat map illuminates the peak region on chromosome 6B (Rel_RFW_6B_Hap1). In (c,e), the pairwise LD map between SNP markers is marked by D′ values, dark red represents 1, whereas white is for 0. The region surrounded by the red dotted line indicates an LD block that contains significant SNPs. (f–h) Phenotypic comparison of the haplotype groups established for the significant SNPs, as detected by LD block. Different letters indicate statistical difference at *p* < 0.05; *n* indicates the number of genotypes representing each specific haplotype.

Root fresh weight mediated by H_2_O_2_ treatment was represented by relative root fresh weight (Figure 6a). Association mapping identified a total of 18 markers for relative root fresh weight distributed across chromosomes 1A, 1B, 3A, 4A, 5B, 5D, 6B, and 7B, and linked to 339 protein‐coding genes (Figure 6b). These markers exhibited a phenotypic variation ranging from 9.25 to 10.71 (Tables 2a and 2b). Over 90% of major alleles of single markers were linked with high relative root fresh weight (Table 2b). The topmost (*p* = 8.02E‐05) marker BS00065783_51, located on chromosome 5D, explained 12.56% phenotypic variation. This marker was linked with a FAD/NAD(P) binding oxidoreductase family protein‐coding gene involved in oxidation–reduction process (GO: 0055114) (Table 4).

The significant marker AX‐158607168, located on 1B chromosome, was associated with the gene *TraesCS1B01G096200* (*peroxidase*), which is involved in oxidative stress responsive biological process (Table S2). Another significant marker, AX‐158570694, on chromosome 1A was associated with a mitogen‐activated protein kinase (MAPK) gene with MAP kinase activity (Table S2). The major allele of haplotypes located on chromosomes 1B and 6B were linked with higher relative root fresh weight (Figure 6c–h). These haplotypes were associated with the genes encoding F‐box protein family, receptor‐like protein kinase, and pentatricopeptide repeat‐containing protein involved in biological process like oxidation–reduction and protein phosphorylation (Table 4).

### 3.7 GWAS Identified Candidate Loci for Shoot Fresh Weight Variations

**FIGURE 7:**
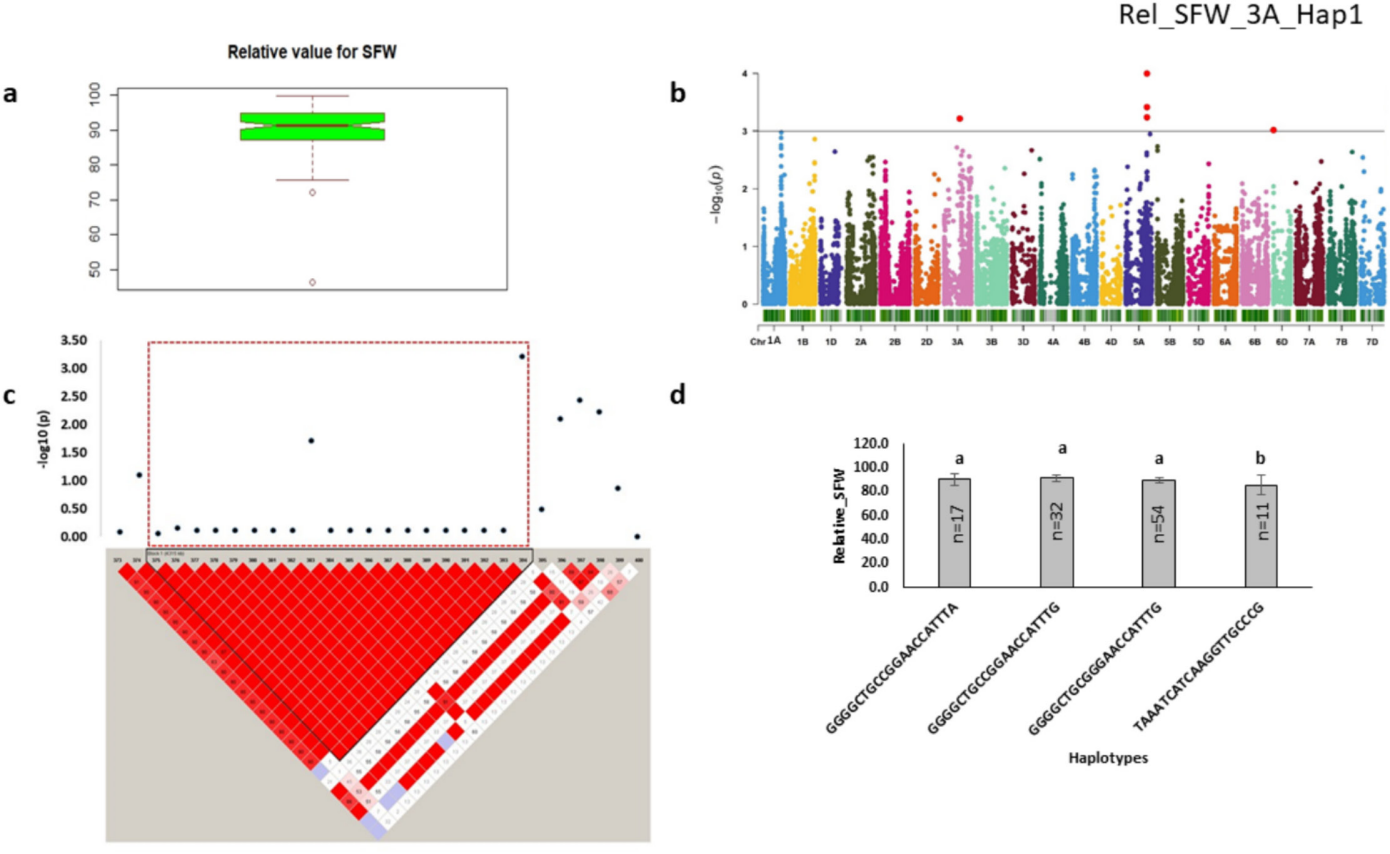
Marker–trait associations for relative shoot fresh weight. (a) The Box plot shows the distribution of relative shoot fresh weight. (b) The Manhattan plot displays the marker‐trait associations; the horizontal red line indicates threshold level (*p* < 0.001); the dots above this line indicate significant markers. (c) The linkage disequilibrium (LD) heat map illuminates the peak region on chromosome 3A (Rel_SFW_3A_Hap1). In (c), the pairwise LD map between SNP markers is marked by D′ values, dark red represents 1, whereas white is for 0. The region surrounded by the red dotted line indicates an LD block that contains significant SNPs. (d) Phenotypic comparison of the haplotype groups established for the significant SNPs, as detected by LD block. Different letters indicate statistical difference at *p* < 0.05; n indicates the number of genotypes representing each specific haplotype.

**FIGURE 8:**
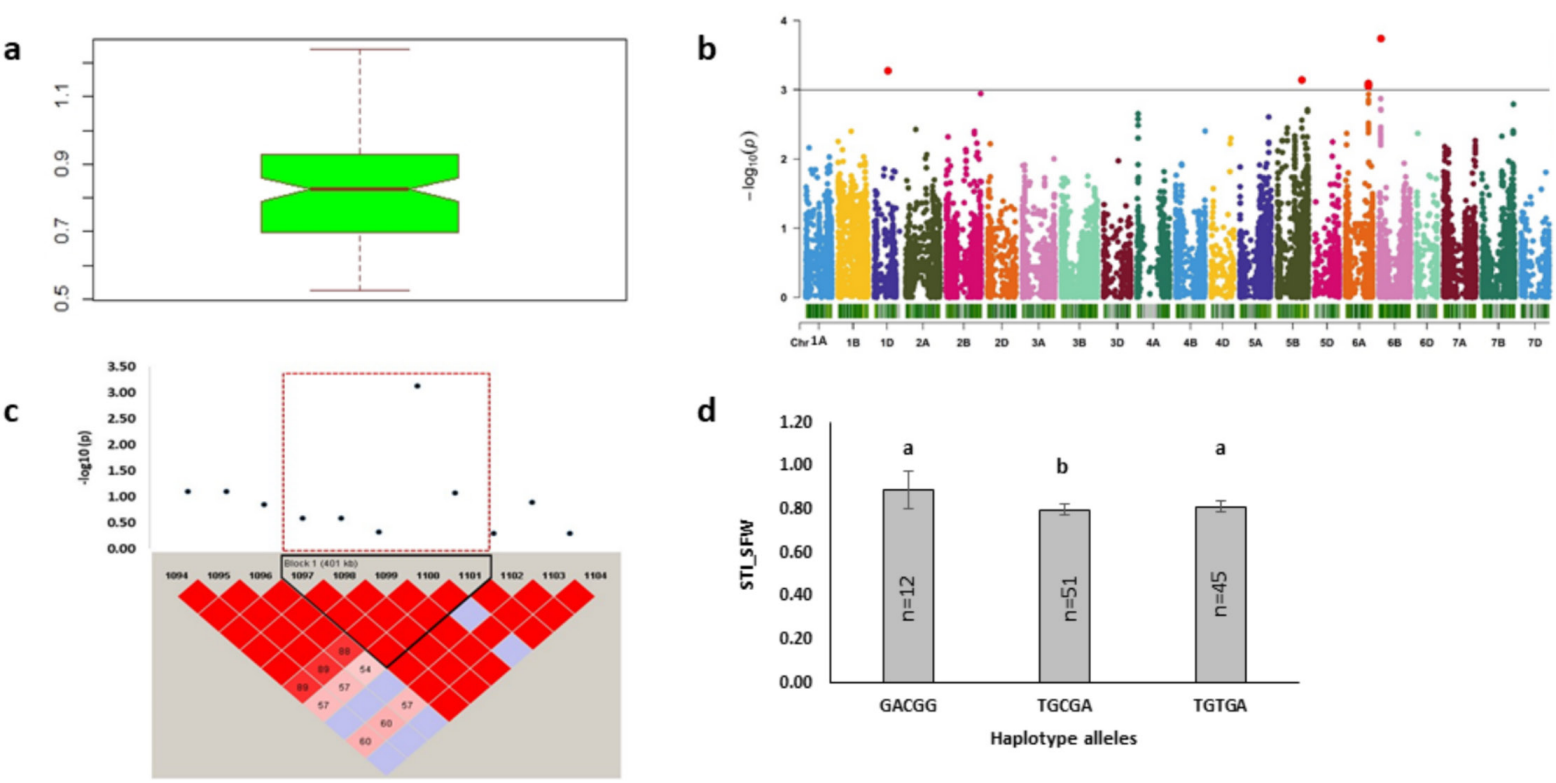
Marker–trait associations for STI shoot fresh weight. (a) Box plot showing the distribution of STI shoot fresh weight. (b) Manhattan plot displays the marker–trait associations; the horizontal red line indicates threshold level (*p* < 0.001); the dots above this line indicate significant markers. (c) The linkage disequilibrium (LD) heat map illuminates the peak region on chromosome 5B (sti_SFW_5B_Hap1). In (c), the pairwise LD map between SNP markers is marked by D′ values, dark red represents 1, whereas white is for 0. The region surrounded by the red dotted line indicates an LD block that contains significant SNPs. (d) Phenotypic comparison of the haplotype groups established for the significant SNPs, as detected by LD block. Different letters indicate statistical difference at *p* < 0.05; *n* indicates the number of genotypes representing each specific haplotype.

H_2_O_2_‐mediated phenotypic variation was represented by relative shoot fresh weight and STI shoot fresh weight (Figures 7a and 8a). Shoot fresh weight was associated with a total of 18 markers located on 1D, 3A, 5A, 5B, 6A,6B, and 6D chromosomes (Figures 7b and 8b). Among identified markers, 6 and 12 explained phenotypic variation ranging from 7.98 to 10.43 and 9.64 to 13.78 for relative shoot fresh weight and STI shoot fresh weight, respectively (Tables 2a and 2b). The topmost marker (*p* = 1.01E‐04), BS00076246_51, was identified for relative shoot fresh weight, linked with the gene *TraesCS5A01G380800*, demonstrating DNA binding function. Major alleles of single markers exhibited significantly higher relative shoot fresh weight. A haplotype formed on 3A chromosome was linked with 26 protein‐coding genes (Figure 7c,d). However, minor allele of STI haplotype showed higher STI shoot fresh weight (Figure 8c,d).

The significant markers were mostly associated with putative candidate genes encoding DNA repair protein, disease resistance protein, and cytochrome P450 family protein. Genes are involved in biological processes including DNA binding, ADP binding, and oxidoreductase activity, acting on paired donors, with incorporation or reduction of molecular oxygen (Tables 4 and S2). The topmost marker (*p* = 1.83E‐04), AX‐158535753, was identified for STI shoot fresh weight. This marker was linked with a putative candidate gene encoding a cytochrome P450 family protein involved in the oxidation–reduction process (GO: 0055114). Single‐marker analysis identified that 80% of major alleles contributed significantly to higher STI shoot fresh weight. However, the major allele of the haplotype was associated with significantly lower STI shoot fresh weight. Top markers were associated with bZIP transcription factor and F‐box/LRR‐repeat protein, involved in oxidation–reduction process, transcription factor activity, and sequence‐specific DNA binding (Table 4).

### 3.8 GWAS Identified Candidate Loci for Root–Shoot Ratio

Larger phenotypic variation was observed in the root–shoot ratio (Figures S2a and S3a). MTA mapping identified a total of 45 markers that satisfied the threshold (*p* ≤ 0.001). These markers were distributed across 2A, 3A, 3B, 3D, 4A, 4B, and 4D chromosomes and were linked to a total of 789 protein‐coding genes (Figures S2b and S3b). Among them, 37 and 8 markers displayed phenotypic variations for the relative root–shoot ratio and STI root–shoot ratio, respectively (Tables 2a and 2b).

The MTA identified the topmost association on 4B chromosome. This marker established the haplotype Com_Hap1, which is also identified as pleiotropic as described above. Single‐marker analysis revealed that 70% of minor alleles showed a lower relative root–shoot ratio compared to the 30% major alleles. Top five markers were associated with the genes encoding protein phosphatase 2C, ethylene‐responsive transcription factor, putative, disease resistance protein involved in molecular catalytic activity, DNA binding, and ADP binding functions. Similarly, for STI root–shoot ratio, more than 70% of major alleles of single markers contributed to a high STI root‐shoot ratio (Tables 2a and 2b). The top markers were linked with NBS‐LRR disease resistance protein, receptor‐like protein kinase, aquaporin protein‐coding genes, involved in protein kinase, ADP binding, and transport activity. Overall, GWAS analyses showed H_2_O_2_‐mediated root and shoot growth variability and identified associated candidate loci and putative candidate genes. The identified genes were involved mostly in metabolic pathways and functions.

### 3.9 Pleiotropy of Candidate Loci Revealed More Than One Effect

The pleiotropy of candidate loci was assessed to identify whether traits share common loci. We identified a sum of 11 SNPs that shared both STI and relative value; therefore, these were designated as pleiotropic markers. We observed that these significant markers are linked to a total of 373 protein‐coding genes (Tables 2a and 2b). Six SNPs out of the 11 were located on 3B chromosome and coincided with 134 protein‐coding genes, encompassing both STI shoot length and STI root–shoot ratio. A haplotype block (Com_Hap1) located on 4A chromosome was associated with 157 protein‐coding genes (Table S6). This haplotype block was pleiotropic for both relative root length and relative root–shoot ratio. Similarly, the markers AX‐158600273 and AX‐158600281 were located on 6A chromosome and linked with 9 and 2 protein‐coding genes, respectively. These markers were pleotropic for relative root length and STI shoot fresh weight (Table 3).

## 4 Discussion

H_2_O_2_ production is a common phenomenon in stress situations; therefore, stress‐related functions are directly linked to H_2_O_2_ signaling and metabolism. Understanding the genetic components of H_2_O_2_ will be helpful in determining how this molecule contributes to stress adaptation and aids in developing stress‐tolerant crop varieties (Mhamdi 2023; Si et al. 2018). The present study assessed H_2_O_2_‐mediated genetic variation of root and shoot traits, and the candidate loci and genes might contribute to tolerance mechanisms in wheat. A significant treatment effect was observed among the root and shoot traits, including root length, shoot length, root–shoot ratio, total length, shoot fresh weight, shoot dry weight, root fresh weight, and root dry weight. The ANOVA revealed a substantial genetic variation among the cultivars under both control and H_2_O_2_ treatment conditions (Table 1). H_2_O_2_ treatment reduced root and shoot growth compared to the control treatment and exhibited a wider phenotypic variation among the cultivars. Our findings are underpinned by previous studies (Jira‐anunkul and Pattanagul 2021; Pasternak et al. 2023). H_2_O_2_ has been shown to modulate root architecture by regulating root length, lateral root formation, and adventitious root formation (Hernández‐Barrera et al. 2015). Furthermore, the relative value and STI represented the relative growth reduction also showed a greater variation among the cultivars. These findings suggest that the studied panel is rich in high genetic diversity, which is advantageous for genetic improvement of traits according to Swarup et al. (2021).

Moreover, high broad‐sense heritability was observed among the traits in both control and H_2_O_2_ treatments, indicating that the phenotypic variation is controlled by genetic factors (Table 1). Similar findings were also observed for yield and photosynthesis‐related traits in this population panel in previous studies (Voss‐Fels et al. 2019; Koua et al. 2021; Siddiqui et al. 2021). In addition, genetic variability of modern and traditional subgroups was depicted for traits such as relative shoot length, STI shoot length, relative root–shoot ratio, and STI root–shoot ratio. The underlying LD decay plot exhibited a contrasting decay pattern across A, B, and D genomes (Figure 1e,f). These results also indicate that the phenotypic variations between traditional and modern cultivars are linked to genetic factors. Recently, Kamruzzaman et al. (2022) found phenotypic differences among the wheat cultivars derived from two distinct genetic subgroups, supporting our findings.

Root length is an important bases for genetic improvement of wheat, as it is associated with nutrient and mineral acquisition. Chen et al. (2022) identified 28 QTLs in wheat that were linked to root and shoot‐related traits at the seedling stage under normal conditions. The present study identified a total of 108 MTAs across different chromosomes. Among them, H_2_O_2_‐induced root length was assessed as relative root length and STI root length. These markers identified on 3B and 2A for relative root length and STI root length are unique and will help to understand how they control root length. Therefore, these markers can be incorporated into marker‐assisted breeding programs to improve H_2_O_2_ stress tolerance wheat varieties. Further observations identified that major alleles of the haplotype and single markers are linked with higher STI root length, STI shoot length, relative shoot length, relative shoot fresh weight, relative root fresh weight, STI shoot fresh weight, and STI root–shoot ratio, respectively, as opposed to the trait relative root length and relative root‐shoot ratio. Single markers and haplotypes of relative root length and relative root–shoot ratio are linked with the higher corresponding values. The alleles linked to high relative and STI values for root and shoot traits indicate possession of a tolerance mechanism. Similar findings have been reported in previous studies, supporting our results (Nayak et al. 2021; Tao et al. 2021).

MTA was identified for shoot length on 1B, 2D, 4B, 5A, and 7D, as well as 1A, 3B, 3D and 5B chromosomes, including the top most markers on 5A and 3B for relative shoot length and STI shoot length, respectively. These loci will provide valuable insight into regulation of shoot length mediated by H_2_O_2_. Similarly, loci associated with relative root fresh weight, STI shoot fresh weight, relative root–shoot ratio, and STI root–shoot ratio were identified on different chromosomes, potentially involved in H_2_O_2_‐mediated root–shoot growth regulation.

A distinct LD decay pattern was observed between modern and traditional cultivar groups. Modern cultivars bearing favorable alleles showed higher relative values in response to H_2_O_2_, indicating greater tolerance to H_2_O_2_ toxicity. The result aligns with Koua et al. (2022), where modern cultivars demonstrated more resilience to drought as compared to the traditional cultivars. Therefore, modern cultivars harboring favorable alleles in our study could be utilized in H_2_O_2_‐mediated marker‐assisted program in wheat.

The present study identified a candidate gene, *TraesCS3B01G134400*, on the 3B chromosome that encodes a zinc finger family protein. This gene has a nucleic acid binding function. Zinc finger family protein has been shown to be involved in H_2_O_2_ regulation in the previous studies (Bouard and Houde 2022; Huang et al. 2022). Therefore, the gene reported in this study has a high likelihood of being involved in H_2_O_2_‐linked root length in wheat. The topmost marker for STI root length is associated with a gene that encodes a disease resistance protein. A previous study reported that a disease resistance protein‐coding gene, N1P1, exhibits high expression in response to H_2_O_2_, supporting our result (Sadhukhan et al. 2017).

The MTA for relative shoot length identified the topmost marker on 5A, and the linked gene encodes heavy metal transport/detoxification protein. Zhang et al. (2023) demonstrated that the toxicity of heavy metals is mediated by the formation of ROS, particularly H_2_O_2_ in roots. However, to reduce root damage, heavy metals are transported to the shoots. For STI shoot length and STI root–shoot ratio, the topmost SNP marker is associated with a receptor‐like protein kinase gene. RLK is one of the largest gene families in plants, and have been the subject of intense research in recent years. RLKs play an important role in plant growth regulation, stress response, and signaling (Zhu et al. 2023). The topmost MTA for relative root fresh weight was identified on 5D, harboring a gene involved in the oxidation–reduction activity. Previous studies suggest that cellular signaling events are mainly based on redox reactions; therefore, it is plausible that the linked gene of oxidoreductase family protein is directly linked to the cellular redox metabolism (Sellés Vidal et al. 2018; Fang et al. 2022). The top marker for relative shoot fresh weight coincided with the gene *TraesCS5A01G380800*, which is involved in DNA binding activities. A previous study identified that H_2_O_2_ was associated with DNA binding activities through expression regulation of the DNA binding proteins (Li et al. 2019).

One significant marker (AX‐158570694) for relative root fresh weight was linked with the MAPK encoding gene *TraesCS1B01G104900* (Table S2). MAPK cascades are important signaling modules in plants, functioning downstream of sensors/receptors to coordinate cellular responses involved in normal growth, development, and stress adaptation (Zhang et al. 2022). Therefore, MAPK gene in this study might be involved in H_2_O_2_ signaling pathways. A few loci were linked with the genes related to DNA and ATP binding activities (Table S2). The significant marker BS00068415_51 was associated with both STI shoot length and STI root–shoot ratio. This SNP was linked with the protein phosphatase family (Table S3), which is largely involved in ROS signaling (Xing et al. 2021). Overall, the molecular functions of identified genes are biologically involved in stress‐related pathways.

In conclusion, wheat genetic diversity revealed significant variation for root and shoot traits under a toxic dose of H_2_O_2_. Genome‐wide association analysis identified the putative candidate loci and genes distributed across different chromosomes. Contrasting alleles that distinguished higher and lower root and shoot growth, along with the putative genes linked to the H_2_O_2_ and ROS, are valuable resources to be incorporated in physiological studies for understanding adaptive mechanisms in wheat and related crop species.

## Author Contributions

M.K., A.A.N., A.B., and J.L. conceptualized the research idea. A.A.N., M.K., and J.L. designed the research. M.K., M.A.B., and M.N.S. performed the experiment. M.K., M.N.S., and S.R. analyzed the data. M.K., A.A.N., S.R., A.B., and J.L. wrote the paper with input from all authors.

## Conflicts of Interest

The authors declare no conflicts of interest.

## Supporting information


**Figure S1.** Seedlings of a bread wheat cultivar represent growth after 7 days under control (without H_2_O_2_), 100, 200, and 500 μM and 20, 100, and 150 mM of H_2_O_2_ treatment, respectively.
**Figure S2.** Marker–trait associations for relative root‐ shoot ratio. (a) The box plot shows the distribution of relative root‐ shoot ratio. (b) The Manhattan plot displays the marker–trait associations; the horizontal red line indicates threshold level (*p* < 0.001); the dots above this line indicate significant markers. (c) The linkage disequilibrium (LD) heat map illuminates the peak region on chromosome 4B (Com_Hap1). In (c), the pairwise LD map between SNP markers is marked by D′ values, dark red represents 1, whereas white is for 0. The region surrounded by the red dotted line indicates an LD block that contains significant SNPs. (d) Phenotypic comparison of the haplotype groups established for the significant SNPs, as detected by LD block. Different letters indicate statistical difference at *p* < 0.05; n indicates the number of genotypes representing each specific haplotype.
**Figure S3.** Marker–trait associations for STI_root shoot ratio. (a) The box plot shows the distribution of STI root‐ shoot ratio. (b) The Manhattan plot displays the marker‐trait associations; the horizontal red line indicates threshold level (*p* < 0.001); the dots above this line indicate significant markers.

**Table S1.** List of cultivars used in the present study, their origin, and year of release.
**Table S2.** List of significant haplotypes/SNPs for relative values and corresponding chromosomal position and their linked candidate genes in 1 Mb span of up‐ and downstream regions.
**Table S3.** List of significant haplotypes/SNPs for STI, and corresponding chromosomal position and their linked candidate genes in 1 Mb span of up‐ and downstream regions.
**Table S4.** Pearson's correlation coefficients among root and shoot traits under H_2_O_2_ treatment in the evaluated wheat association panel.
**Table S5a.** Relative root length of modern and traditional cultivar groups carrying the favorable allele (GTGAGCC) of Rel_SL_1A_Hap1.
**Table S5b.** Stress tolerance index (STI) of modern and traditional cultivar groups carrying the favorable allele (CGGT) of sti_SL_1A_Hap1.
**Table S6.** The haplotype blocks associated with different traits and their chromosomal positions and alleles.

## Data Availability

The data that support the findings of this study are available from Mohammad Kamruzzaman (kamruzzaman_bina2013@yahoo.com) upon request.
