# Supplementary material for: Genome‐Wide Association Analyses Identify Hydrogen Peroxide–Responsive Loci in Wheat Diversity
Source: Plant Direct. 2025 Apr 17;9(4):e70067. doi: 10.1002/pld3.70067 (PMC12004125; doi:10.1002/pld3.70067)
Supplement: Supplementary file 1 — Figure S1. Seedlings of a bread wheat cultivar represent growth after 7 days under control (without H2O2), 100, 200, and 500 μM and 20, 100, and 150 mM of H2O2 treatment, respectively. Figure S2. Marker–trait associations for relative root‐ shoot ratio. (a) The box plot shows the distribution of relative root‐ shoot ratio. (b) The Manhattan plot displays the marker–trait associations; the horizontal red line indicates threshold level (p < 0.001); the dots above this line indicate significant markers. (c) The linkage disequilibrium (LD) heat map illuminates the peak region on chromosome 4B (Com_Hap1). In (c), the pairwise LD map between SNP markers is marked by D′ values, dark red represents 1, whereas white is for 0. The region surrounded by the red dotted line indicates an LD block that contains significant SNPs. (d) Phenotypic comparison of the haplotype groups established for the significant SNPs, as detected by LD block. Different letters indicate statistical difference at p < 0.05; n indicates the number of genotypes representing each specific haplotype. Figure S3. Marker–trait associations for STI_root shoot ratio. (a) The box plot shows the distribution of STI root‐ shoot ratio. (b) The Manhattan plot displays the marker‐trait associations; the horizontal red line indicates threshold level (p < 0.001); the dots above this line indicate significant markers. [file PLD3-9-e70067-s002.docx]

**Supplementary Figures Legends:**

**Fig. S1.** Phenotypic representation of seedling of a bread wheat cultivar after 7 days under control and different doses of H_2_O_2_ treatments

**Fig. S2.** Marker-trait associations for relative root shoot ratio.

**Fig. S3.** Marker-trait associations for STI root shoot ratio.


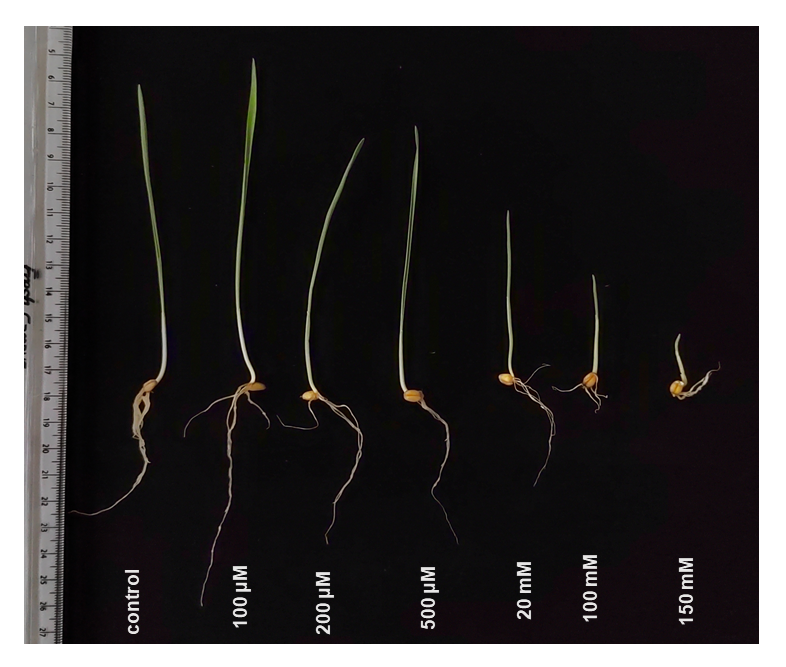


**Figure S1.** Seedlings of a bread wheat cultivar represent growth after 7 days under control (without H_2_O_2_), 100, 200, and 500 μM, and 20 , 100, and 150 mM of H_2_O_2_ treatment, respectively


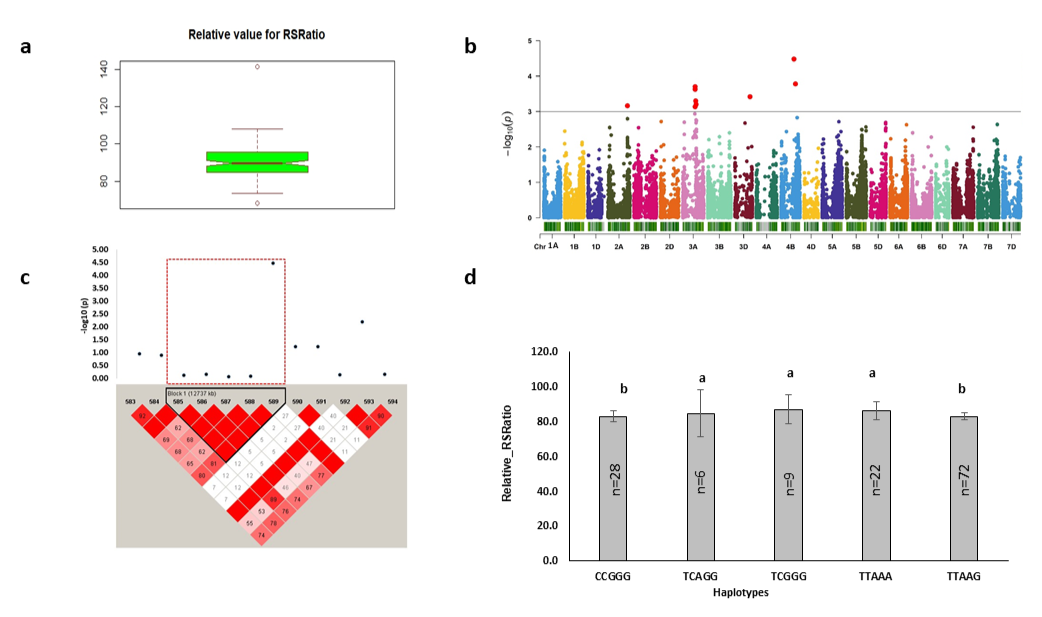


**Figure S2.** Marker-trait associations for relative root- shoot ratio. (a) The box plot shows the distribution of relative root- shoot ratio; (b) The Manhattan plot displays the marker-trait associations; the horizontal red line indicates threshold level (P < 0.001), the dots above this line indicate significant markers. (c) The linkage disequilibrium (LD) heat map illuminates the peak region on chromosome 4B (Com_Hap1). In (c), the pair‐wise LD map between SNP markers is marked by D′ values, dark red represents 1, whereas white is for 0. The region surrounded by the red dotted line indicates an LD block that contains significant SNPs. (d) Phenotypic comparison of the haplotype groups established for the significant SNPs, as detected by LD block. Different letters indicate statistical difference at P < 0.05, n indicates the number of genotypes representing each specific haplotype.


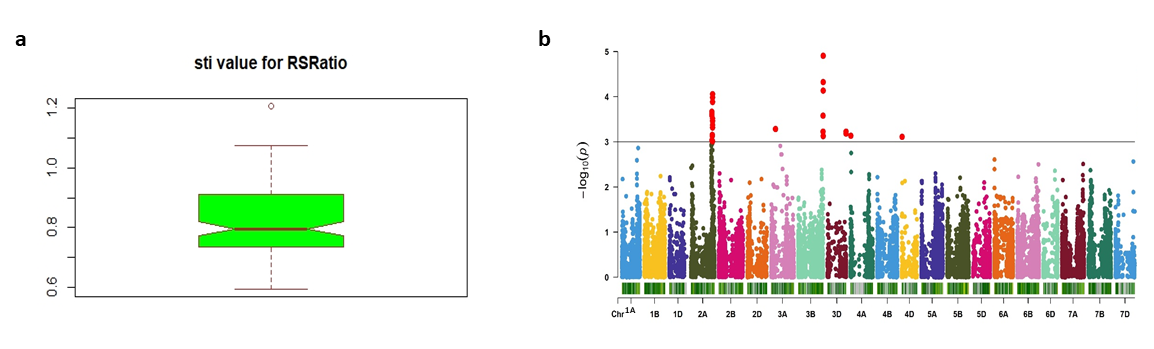


**Figure S3.** Marker-trait associations for STI_root- shoot ratio. (a) The box plot shows the distribution of STI_root shoot ratio; (b) The Manhattan plot displays the marker-trait associations; the horizontal red line indicates threshold level (P < 0.001), the dots above this line indicate significant markers.
