# Supplementary material for: Genome‐Wide Association Analyses Identify Hydrogen Peroxide–Responsive Loci in Wheat Diversity
Source: Plant Direct. 2025 Apr 17;9(4):e70067. doi: 10.1002/pld3.70067 (PMC12004125; doi:10.1002/pld3.70067)
Supplement: Supplementary file 2 — Table S1. List of cultivars used in the present study, their origin, and year of release. Table S2. List of significant haplotypes/SNPs for relative values and corresponding chromosomal position and their linked candidate genes in 1 Mb span of up‐ and downstream regions. Table S3. List of significant haplotypes/SNPs for STI, and corresponding chromosomal position and their linked candidate genes in 1 Mb span of up‐ and downstream regions. Table S4. Pearson's correlation coefficients among root and shoot traits under H2O2 treatment in the evaluated wheat association panel. Table S5a. Relative root length of modern and traditional cultivar groups carrying the favorable allele (GTGAGCC) of Rel_SL_1A_Hap1. Table S5b. Stress tolerance index (STI) of modern and traditional cultivar groups carrying the favorable allele (CGGT) of sti_SL_1A_Hap1. Table S6. The haplotype blocks associated with different traits and their chromosomal positions and alleles. [file PLD3-9-e70067-s001.docx]

**Table S1. List of cultivars used in the present study, their origin and year of release**

| **SL No.** | **Cultivar** | **Origin** | **Year of Release** |
| --- | --- | --- | --- |
| WH1 | Einstein | GB | 2004 |
| WH2 | Oakley | UK/BE | 2008 |
| WH3 | Jafet | Germany | 2008 |
| WH4 | Claire | IE/UK | 1999 |
| WH5 | Rebell | Germany | 2013 |
| WH6 | Memory | Germany | 2013 |
| WH7 | Kurt | Germany | 2013 |
| WH8 | Zappa | Germany | 2009 |
| WH9 | Chevalier | AT, CZ, LT, LU | 2005 |
| WH10 | Gordian | Germany | 2013 |
| WH11 | Mentor | Germany | 2012 |
| WH12 | Meister | Germany | 2010 |
| WH13 | KWS Santiago | England | 2011 |
| WH14 | Brigand | GBR | 1979 |
| WH15 | Profilus | Germany | 2008 |
| WH16 | Durin | Frankreich | Unknown |
| WH17 | KWS Pius | Germany | 2010 |
| WH18 | Paroli | Germany | 2004 |
| WH19 | Estivus | Germany | 2012 |
| WH20 | Desamo | Germany | 2013 |
| WH21 | Carenius | Germany | 2006 |
| WH22 | Mulan | Germany | 2006 |
| WH23 | Nelson | Germany | 2011 |
| WH24 | Patras | Germany | 2012 |
| WH25 | Götz | Germany | 1978 |
| WH26 | Robigus | England | 2004 |
| WH27 | Anapolis | Germany | 2013 |
| WH28 | Solstice | England | 2001 |
| WH29 | Capone | Germany | 2012 |
| WH30 | Tabasco | Germany | 2008 |
| WH31 | Cubus | Germany | 2002 |
| WH32 | Edward | Germany | 2013 |
| WH33 | SW Topper | USA | 2002 |
| WH34 | Jenga | Germany | 2007 |
| WH35 | Linus | Germany | 2010 |
| WH36 | TJB 990-15 | GBR | 1980 |
| WH37 | Forum | DE/EE/PO/SE | 2012 |
| WH38 | Colonia | DE/BE/HU | 2011 |
| WH39 | Transit | DE | 1994 |
| WH40 | Gaucho | USA | 1993 |
| WH41 | Tarso | Germany | 1992 |
| WH42 | Hermann | Germany | 2004 |
| WH43 | Glaucus | Germany | 2011 |
| WH44 | Atomic | Germany | 2012 |
| WH45 | Tobak | Germany | 2011 |
| WH46 | Manager | Germany | 2006 |
| WH47 | Gourmet | Germany | 2013 |
| WH48 | Limes | Germany | 2003 |
| WH49 | Kalahari | DE/BE | 2010 |
| WH50 | Zobel | Germany | 2006 |
| WH51 | Global | DE/AT | 2009 |
| WH52 | Greif | Germany | 1989 |
| WH53 | Skalmeje | Germany | 2006 |
| WH54 | Genius | Germany | 2010 |
| WH55 | Enorm | Germany | 2002 |
| WH56 | Florian | Germany | 2010 |
| WH57 | Skater | Germany | 2000 |
| WH58 | Brilliant | Germany | 2005 |
| WH59 | Maris Huntsman | Germany | 1975 |
| WH60 | Landsknecht | Germany | 2013 |
| WH62 | Impression | Germany | 2005 |
| WH63 | Winnetou | Germany | 2002 |
| WH64 | Toronto | Germany | 1990 |
| WH65 | Torrild | Germany | 2005 |
| WH66 | Contra | Germany | 1990 |
| WH67 | Schamane | Germany | 2005 |
| WH68 | Granada | Germany | 1980 |
| WH69 | Tommi | Germany | 2002 |
| WH71 | JB Asano | Germany | 2008 |
| WH72 | Kerubino | Germany | 2004 |
| WH73 | NaturaSt | Germany | 2002 |
| WH74 | Orestis | Germany | 1988 |
| WH75 | Flair | Germany | 1996 |
| WH76 | Anthus | Germany | 2005 |
| WH77 | Bombus | Germany | 2012 |
| WH78 | Lucius | Germany | 2006 |
| WH79 | Sorbas | Germany | 1985 |
| WH80 | Magister | Germany | 2005 |
| WH81 | Aristos | Germany | 1966 |
| WH82 | Joss | Germany | 1972 |
| WH83 | Sperber | Germany | 1982 |
| WH84 | Helios | USA | 2013 |
| WH85 | Obelisk | NE; DE | 1987 |
| WH86 | Disponent | Germany | 1975 |
| WH87 | Tambor | Germany | 1993 |
| WH88 | Boxer | Germany | 2013 |
| WH89 | Sokrates | Germany | 2001 |
| WH90 | Carisuper | Germany | 1975 |
| WH91 | Rektor | Germany | 1980 |
| WH92 | Alidos | Germany | 1987 |
| WH93 | Cardos | Germany | 1975 |
| WH94 | Akratos | Germany | 2004 |
| WH95 | Knirps | Germany | 1985 |
| WH96 | Oberst | DE | 1980 |
| WH97 | Cappelle Desprez | FR, CHL, GBR, NL | 1946 |
| WH98 | Ibis | Germany | 1991 |
| WH99 | Batis | Germany | 1994 |
| WH100 | Akteur | Germany | 2003 |
| WH101 | Astron | Germany | 1989 |
| WH102 | Basalt | Germany | 1980 |
| WH103 | Aron | Germany | 1992 |
| WH104 | Aszita | Germany | 2005 |
| WH105 | Kobold | Germany | 1978 |
| WH106 | Vuka | Germany | 1975 |
| WH107 | Benno | Germany | 1973 |
| WH108 | Aquila | GRB/IT | 1979 |
| WH109 | Kraka | Germany | 1982 |
| WH110 | Caribo | Germany | 1968 |
| WH111 | Konsul | Germany | 1990 |
| WH112 | Centurk | USA | 1971 |
| WH113 | NS 22/92 | Serbien | Unknown |
| WH114 | Benni multifloret | USA/Indiana | 1980 |
| WH115 | Hope | USA/(S.Dakota) | 1927-1948 |
| WH116 | Vel | USA | Unknown |
| WH117 | Phoenix | AUS New-South-Wales | 1981 |
| WH118 | Mironovska 808 | Ukraine | 1963 |
| WH119 | Caphorn | Frankreich | 2000 |
| WH120 | Cordiale | England | 2003 |
| WH121 | Apache | CZ | 1997 |
| WH122 | Isengrain | FR/SI/ES | 1996 |
| WH123 | Alixan | Frankreich | 2005 |
| WH124 | Boregar | Frankreich | 2007 (BSA)/2008 (EU) |
| WH125 | Renesansa | Serbien | 1995 |
| WH126 | Tremie | ES, FR, IT, | 1991 |
| WH127 | Triple Dirk "S" | Australien | Unknown |
| WH128 | Soissons | BE, Es, FR, IE, IT; SI | 1987 |
| WH129 | BCD 1302/83 | Moldavien | Unknown |
| WH130 | Arlequin | Frankreich | 2007 |
| WH131 | Sonalika | Indien | 1967 |
| WH132 | Camp Remy | Germany | 1980 |
| WH133 | Cajeme 71 | Mexico | 1971 |
| WH134 | Avalon | GBR; | 1980 |
| WH135 | Ivanka | Serbien | 1998 |
| WH136 | Pobeda | Serbien | 1990 |
| WH137 | NS 66/92 | Serbien | Unknown |
| WH138 | Mexico 3 | Mexico | Unknown |
| WH139 | Orcas | Germany | 2010 |
| WH140 | Nimbus | Germany | 1975 |
| WH141 | Florida | USA | 1984 |
| WH142 | Highbury | GBR | 1968 |
| WH143 | Siete Cerros 66 | Mexiko | 1966 |
| WH144 | Kontrast | Germany | 1990 |
| WH145 | WW 4180 | Germany | 2012 |
| WH146 | INTRO 615 | USA | Unknown |
| WH147 | NS 46/90 | Serbien | Unknown |
| WH149 | Lambriego Inia | Chile | 1980 |

**Table S2: List of significant Haplotypes/SNPs for relative values, and corresponding chromosomal position and their linked candidate genes in 1Mb span of up and down stream regions**

| **Trait** | **Marker** | **Haplotype** | **Chromosome** | **SNP position (bp)** | **Genes** | **Description** | **Protein Family** | **Gene Ontology (GO)** |
| --- | --- | --- | --- | --- | --- | --- | --- | --- |
| Relative root fresh weight | Kukri_c67721_184 |  | 1A | 21282106 | TraesCS1A01G036600 | Rotundifolia-like protein | PF08137: DVL family | NA |
|  |  |  |  |  | TraesCS1A01G036700 | Short-chain dehydrogenase/reductase family protein | PF13561: Enoyl-(Acyl carrier protein) reductase | NA |
|  |  |  |  |  | TraesCS1A01G036800 | F-box family protein | PF03478: Protein of unknown function (DUF295) | GO:0005515 MF: protein binding |
|  |  |  |  |  | TraesCS1A01G036900 | Vromindoline VIN2 | NA | NA |
|  |  |  |  |  | TraesCS1A01G037000 | RNA helicase, ATP-dependent, SK12/DOB1 protein | NA | NA |
|  |  |  |  |  | TraesCS1A01G037100 | F-box protein | PF00646: F-box domain | GO:0005515 MF: protein binding |
|  |  |  |  |  | TraesCS1A01G037200 | AWPM-19-like membrane family protein | PF05512: AWPM-19-like family | NA |
|  |  |  |  |  | TraesCS1A01G037300 | Thaumatin | PF00314: Thaumatin family | NA |
|  |  |  |  |  | TraesCS1A01G037400 | Cation/H(+) antiporter | PF00999: Sodium/hydrogen exchanger family | GO:0006812 BP: cation transport;GO:0015299 MF: solute:proton antiporter activity; |
|  |  |  |  |  | TraesCS1A01G037500 | 3-ketoacyl-CoA synthase 5 | NA | NA |
|  |  |  |  |  | TraesCS1A01G037600 | 3-ketoacyl-CoA synthase | PF08392: FAE1/Type III polyketide synthase-like protein; PF08541: 3-Oxoacyl-[acyl-carrier-protein (ACP)] synthase III C terminal | GO:0003824 MF: catalytic activity;GO:0016747 MF: transferase activity, transferring acyl groups other than amino-acyl groups |
|  |  |  |  |  | TraesCS1A01G037700 | Phenylalanine ammonia-lyase | PF00221: Aromatic amino acid lyase | GO:0003824 MF: catalytic activity;GO:0016841 MF: ammonia-lyase activity |
|  |  |  |  |  | TraesCS1A01G037800 | Phenylalanine ammonia-lyase | PF00221: Aromatic amino acid lyase | GO:0003824 MF: catalytic activity;GO:0016841 MF: ammonia-lyase activity |
|  |  |  |  |  | TraesCS1A01G037900 | BRI1-KD interacting protein | NA | NA |
|  |  |  |  |  | TraesCS1A01G038000 | Powder tolerance-related protein | NA | NA |
|  |  |  |  |  | TraesCS1A01G038100 | Histone H2A | PF00125: Core histone H2A/H2B/H3/H4; PF16211: C-terminus of histone H2A | GO:0000786 CC: nucleosome;GO:0003677 MF: DNA binding; |
|  |  |  |  |  | TraesCS1A01G038200 | Histone H2A | PF00125: Core histone H2A/H2B/H3/H4; PF16211: C-terminus of histone H2A | GO:0005634 CC: nucleus;GO:0046982 MF: protein heterodimerization activity |
|  |  |  |  |  | TraesCS1A01G038300 | Inorganic pyrophosphatase family protein | PF00719: Inorganic pyrophosphatase | GO:0005737 CC: cytoplasm;GO:0006796 BP: phosphate-containing compound metabolic process |
|  |  |  |  |  | TraesCS1A01G038400 | Poly [ADP-ribose] polymerase | PF00645: Poly(ADP-ribose) polymerase and DNA-Ligase Zn-finger region; PF08063: PADR1 (NUC008) domain; PF00533: BRCA1 C Terminus (BRCT) domain; PF05406: WGR domain; PF02877: Poly(ADP-ribose) polymerase, regulatory domain; PF00644: Poly(ADP-ribose) polymerase catalytic domain | GO:0003677 MF: DNA binding;GO:0003950 MF: NAD+ ADP-ribosyltransferase activity; |
|  |  |  |  |  | TraesCS1A01G038500 | Unconventional myosin-Va | NA | NA |
|  |  |  |  |  | TraesCS1A01G038600 | Pm3-like disease resistance protein | PF00931: NB-ARC domain | GO:0043531 MF: ADP binding |
|  |  |  |  |  | TraesCS1A01G038700 | NBS-LRR-like resistance protein | PF00931: NB-ARC domain | GO:0043531 MF: ADP binding |
|  |  |  |  |  | TraesCS1A01G038800 | UDP-N-acetylenolpyruvoylglucosamine reductase | NA | NA |
|  |  |  |  |  | TraesCS1A01G039000 | Defensin | NA | NA |
|  |  |  |  |  | TraesCS1A01G039100 | TF-B3 domain-containing protein | NA | GO:0003677 , MF: DNA binding |
|  |  |  |  |  | TraesCS1A01G039200 | MORC family CW-type zinc finger protein 4 | PF13589: Histidine kinase-, DNA gyrase B-, and HSP90-like ATPase | NA |
|  |  |  |  |  | TraesCS1A01G039300 | Type I inositol-1,4,5-trisphosphate 5-phosphatase 1 | PF03372: Endonuclease/Exonuclease/phosphatase family | GO:0046856 BP: phosphatidylinositol dephosphorylation |
|  |  |  |  |  | TraesCS1A01G039400 | NA | NA | NA |
|  |  |  |  |  | TraesCS1A01G039500 | NA | NA | NA |
|  |  |  |  |  | TraesCS1A01G039600 | High molecular weight glutenin subunit | NA | NA |
|  |  |  |  |  | TraesCS1A01G039700 | ABC transporter, putative | PF00664: ABC transporter transmembrane region; PF00005: ABC transporter | GO:0005524 MF: ATP binding;GO:0006810 BP: transport;;GO:0055085 BP: transmembrane transport |
|  |  |  |  |  | TraesCS1A01G039800 | 3-ketoacyl-CoA synthase | PF08392: FAE1/Type III polyketide synthase-like protein; PF08541: 3-Oxoacyl-[acyl-carrier-protein (ACP)] synthase III C terminal | GO:0003824 MF: catalytic activity;GO:0006633 BP: fatty acid biosynthetic process; |
|  |  |  |  |  | TraesCS1A01G039900 | S-adenosyl-L-methionine-dependent methyltransferases superfamily protein | PF13968: Domain of unknown function (DUF4220); PF04578: Protein of unknown function, DUF594 | NA |
|  |  |  |  |  | TraesCS1A01G040000 | Sulfite reductase [NADPH] hemoprotein beta-component | NA | NA |
|  |  |  |  |  | TraesCS1A01G040100 | Zinc finger family protein | PF13912: C2H2-type zinc finger | GO:0003676 MF: nucleic acid binding |
|  |  |  |  |  | TraesCS1A01G040200 | Zinc finger family protein | PF13912: C2H2-type zinc finger | GO:0003676 MF: nucleic acid binding |
|  |  |  |  |  | TraesCS1A01G442200 | Flavonoid 3'-hydroxylase | PF00067: Cytochrome P450 | PF00067: Cytochrome P450 |
| Relative root length | AX-89555340 |  | 1A | 591926658 | TraesCS1A01G442300 | Flavonoid 3'-hydroxylase | PF00067: Cytochrome P450 | PF00067: Cytochrome P450 |
|  |  |  |  |  | TraesCS1A01G442400 | NA | NA | NA |
|  |  |  |  |  | TraesCS1A01G442400 | TATA-binding protein associated factor-like protein | PF12054: Domain of unknown function (DUF3535); PF02985: HEAT repeat; PF00176: SNF2 family N-terminal domain; PF00271: Helicase conserved C-terminal domain | PF12054: Domain of unknown function (DUF3535); PF02985: HEAT repeat; PF00176: SNF2 family N-terminal domain; PF00271: Helicase conserved C-terminal domain |
|  |  |  |  |  | TraesCS1A01G442500 | ATP-dependent zinc metalloprotease FTSH protein | PF00004: ATPase family associated with various cellular activities (AAA); PF01434: Peptidase family M41 | PF00004: ATPase family associated with various cellular activities (AAA); PF01434: Peptidase family M41 |
|  |  |  |  |  | TraesCS1A01G442600 | Protein kinase family protein | PF00069: Protein kinase domain | PF00069: Protein kinase domain |
|  |  |  |  |  | TraesCS1A01G442700 | D111/G-patch domain-containing protein | NA | NA |
|  |  |  |  |  | TraesCS1A01G442800 | Protein kinase family protein | PF00069: Protein kinase domain | PF00069: Protein kinase domain |
|  |  |  |  |  | TraesCS1A01G442900 | F-box family protein | PF03478: Protein of unknown function (DUF295) | PF03478: Protein of unknown function (DUF295) |
|  |  |  |  |  | TraesCS1A01G443000 | Tudor/PWWP/MBT superfamily protein | NA | NA |
|  |  |  |  |  | TraesCS1A01G443100 | Protein kinase | PF00069: Protein kinase domain | PF00069: Protein kinase domain |
|  |  |  |  |  | TraesCS1A01G443200 | Early flowering 3 | NA | NA |
|  |  |  |  |  | TraesCS1A01G443300 | EMBRYO SURROUNDING FACTOR 1.3 | NA | NA |
|  |  |  |  |  | TraesCS1A01G443400 | ATP-dependent RNA helicase | PF00270: DEAD/DEAH box helicase; PF00271: Helicase conserved C-terminal domain | PF00270: DEAD/DEAH box helicase; PF00271: Helicase conserved C-terminal domain |
|  |  |  |  |  | TraesCS1A01G443500 | Carbonic anhydrase | PF00194: Eukaryotic-type carbonic anhydrase | PF00194: Eukaryotic-type carbonic anhydrase |
|  |  |  |  |  | TraesCS1A01G443600 | WD-repeat protein, putative | PF08154: NLE (NUC135) domain; PF00400: WD domain, G-beta repeat | PF08154: NLE (NUC135) domain; PF00400: WD domain, G-beta repeat |
|  |  |  |  |  | TraesCS1A01G443700 | Small nuclear ribonucleoprotein | PF01423: LSM domain | PF01423: LSM domain |
|  |  |  |  |  | TraesCS1A01G443800 | Pathogenesis-related protein 1 | PF00188: Cysteine-rich secretory protein family | PF00188: Cysteine-rich secretory protein family |
|  |  |  |  |  | TraesCS1A01G443900 | UDP-glucose-4-epimerase | PF16363: GDP-mannose 4,6 dehydratase | PF16363: GDP-mannose 4,6 dehydratase |
|  |  |  |  |  | TraesCS1A01G444000 | Pathogenesis-related protein 1 | PF00188: Cysteine-rich secretory protein family | PF00188: Cysteine-rich secretory protein family |
|  |  |  |  |  | TraesCS1A01G444100 | Response regulator | PF00072: Response regulator receiver domain | PF00072: Response regulator receiver domain |
|  |  |  |  |  | TraesCS1A01G444200 | Zinc finger protein CONSTANS | PF06203: CCT motif | PF06203: CCT motif |
|  |  |  |  |  | TraesCS1A01G444300 | Nucleoside diphosphate kinase | PF00334: Nucleoside diphosphate kinase | PF00334: Nucleoside diphosphate kinase |
|  |  |  |  |  | TraesCS1A01G444400 | Zinc finger protein CONSTANS | PF06203: CCT motif | PF06203: CCT motif |
|  |  |  |  |  | TraesCS1A01G444500 | Non-specific serine/threonine protein kinase | PF08263: Leucine rich repeat N-terminal domain; PF12799: Leucine Rich repeats (2 copies); PF00560: Leucine Rich Repeat; PF00069: Protein kinase domain | PF08263: Leucine rich repeat N-terminal domain; PF12799: Leucine Rich repeats (2 copies); PF00560: Leucine Rich Repeat; PF00069: Protein kinase domain |
|  |  |  |  |  | TraesCS1A01G444600 | NA | NA | NA |
|  |  |  |  |  | TraesCS1A01G444700 | Aspartic proteinase nepenthesin-2 | PF14543: Xylanase inhibitor N-terminal; PF14541: Xylanase inhibitor C-terminal | PF14543: Xylanase inhibitor N-terminal; PF14541: Xylanase inhibitor C-terminal |
|  |  |  |  |  | TraesCS1A01G444800 | Translation initiation factor IF-2 | NA | NA |
|  |  |  |  |  | TraesCS1A01G444900 | Ring finger protein, putative | PF13639: Ring finger domain | PF13639: Ring finger domain |
|  |  |  |  |  | TraesCS1A01G445000 | Structural maintenance of chromosomes family protein | PF02463: RecF/RecN/SMC N terminal domain | PF02463: RecF/RecN/SMC N terminal domain |
|  |  |  |  |  | TraesCS1A01G445100 | Response regulator 2, putative | PF00072: Response regulator receiver domain | PF00072: Response regulator receiver domain |
|  |  |  |  |  | TraesCS1A01G445200 | Response regulator | PF00072: Response regulator receiver domain | PF00072: Response regulator receiver domain |
| Relative root fresh weight | AX-158570694 |  | 1B | 115958656 | TraesCS1B01G104900 | Mitogen-activated protein kinase | PF00069: Protein kinase domain | GO:0004672 MF: protein kinase activity;GO:0004707 MF: MAP kinase activity; |
| Relative root fresh weight | AX-158606938 |  | 1B | 223135574 | TraesCS1B01G149300 | plant U-box 26 | NA | NA |
|  |  |  |  |  | TraesCS1B01G149400 | plant U-box 26 | NA | NA |
|  |  |  |  |  | TraesCS1B01G149500 | Ubiquitin carboxyl-terminal hydrolase-like protein | PF06337: DUSP domain; PF00443: Ubiquitin carboxyl-terminal hydrolase | GO:0004843 MF: thiol-dependent ubiquitin-specific protease activity;GO:0006511 BP: ubiquitin-dependent protein catabolic process; |
|  |  |  |  |  | TraesCS1B01G149600 | Prolyl 4-hydroxylase alpha subunit, putative | PF13640: 2OG-Fe(II) oxygenase superfamily | GO:0005506 MF: iron ion binding;GO:0016491 MF: oxidoreductase activity |
|  |  |  |  |  | TraesCS1B01G149700 | Glycerol-3-phosphate acyltransferase | PF12710: haloacid dehalogenase-like hydrolase; PF01553: Acyltransferase | GO:0008152 BP: metabolic process;GO:0016746 MF: transferase activity, transferring acyl groups |
|  |  |  |  |  |  | DNA mismatch repair protein mutS | PF01624: MutS domain I; PF05188: MutS domain II; PF05192: MutS domain III; PF05190: MutS family domain IV; PF00488: MutS domain V | GO:0005524 MF: ATP binding;GO:0006298 BP: mismatch repair;GO:0030983 MF: mismatched DNA binding |
| Relative root fresh weight | AX-158607168 |  | 1B | 106764122 | TraesCS1B01G096200 | Peroxidase | PF00141: Peroxidase | GO:0004601 MF: peroxidase activity;GO:0006979 BP: response to oxidative stress |
|  |  |  |  |  | TraesCS1B01G096300 | Peroxidase | PF00141: Peroxidase | GO:0004601; GO:0006979; GO:0020037; GO:0055114 |
|  |  |  |  |  | TraesCS1B01G096400 | Peroxidase | PF00141: Peroxidase | GO:0004601; GO:0006979; GO:0020037; GO:0055114 |
|  |  |  |  |  | TraesCS1B01G096500 | Zinc finger MYM-type protein 5 | NA | GO:0004601; GO:0006979; GO:0020037; GO:0055114 |
|  |  |  |  |  | TraesCS1B01G096600 | Peroxidase | PF00141: Peroxidase | NA |
|  |  |  |  |  | TraesCS1B01G096700 | phosphoribosylformylglycinamidine synthase | NA | GO:0004601; GO:0006979; GO:0020037; GO:0055114 |
|  |  |  |  |  | TraesCS1B01G096800 | Peroxidase | PF00141: Peroxidase | NA |
|  |  |  |  |  | TraesCS1B01G096900 | Peroxidase | PF00141: Peroxidase | GO:0004601; GO:0006979; GO:0020037; GO:0055114 |
|  |  |  |  |  | TraesCS1B01G097000 | basic helix-loop-helix (bHLH) DNA-binding superfamily protein | PF00010: Helix-loop-helix DNA-binding domain | GO:0004601; GO:0006979; GO:0020037; GO:0055114 |
|  |  |  |  |  | TraesCS1B01G097100 | Lecithin-cholesterol acyltransferase-like 1 | PF02450: Lecithin:cholesterol acyltransferase | GO:0046983 |
|  |  |  |  |  | TraesCS1B01G097200 | Glutathione S-transferase | PF02798: Glutathione S-transferase, N-terminal domain; PF00043: Glutathione S-transferase, C-terminal domain | GO:0006629; GO:0008374 |
|  |  |  |  |  | TraesCS1B01G097300 | Lecithin-cholesterol acyltransferase-like 1 | PF02450: Lecithin:cholesterol acyltransferase | GO:0005515 |
|  |  |  |  |  | TraesCS1B01G097400 | Glutathione S-transferase | PF02798: Glutathione S-transferase, N-terminal domain; PF00043: Glutathione S-transferase, C-terminal domain | GO:0006629; GO:0008374 |
|  |  |  |  |  | TraesCS1B01G097500 | Myb-like transcription factor | PF00249: Myb-like DNA-binding domain | GO:0005515 |
|  |  |  |  |  | TraesCS1B01G097600 | Ubiquitin carboxyl-terminal hydrolase family protein | PF11955: Plant organelle RNA recognition domain | GO:0003677 |
|  |  |  |  |  | TraesCS1B01G097700 | Kinase family protein | PF07714: Protein tyrosine kinase | NA |
|  |  |  |  |  | TraesCS1B01G097800 | myosin heavy chain, cardiac protein | NA | GO:0004672; GO:0005524; GO:0006468 |
|  |  |  |  |  | TraesCS1B01G097900 | Histone H4 | PF15511: Centromere kinetochore component CENP-T histone fold | NA |
|  |  |  |  |  | TraesCS1B01G098000 | Germin-like protein 1 | PF00190: Cupin | GO:0000786; GO:0003677; GO:0005634; GO:0006334; GO:0046982 |
|  |  |  |  |  | TraesCS1B01G098100 | DNA binding protein, putative | NA | GO:0030145; GO:0045735 |
|  |  |  |  |  | TraesCS1B01G098200 | Zinc ion binding protein | NA | NA |
|  |  |  |  |  | TraesCS1B01G098300 | Peptide chain release factor 1 | PF03462: PCRF domain; PF00472: RF-1 domain | NA |
|  |  |  |  |  | TraesCS1B01G098400 | BTB/POZ domain containing protein | PF00651: BTB/POZ domain | GO:0003747; GO:0005737; GO:0006415; GO:0016149 |
|  |  |  |  |  | TraesCS1B01G098500 | ATP-dependent kinase YFH7 | NA | GO:0005515 |
|  |  |  |  |  | TraesCS1B01G098600 | Non-specific serine/threonine protein kinase | PF00069: Protein kinase domain; PF03822: NAF domain | NA |
|  |  |  |  |  | TraesCS1B01G098700 | Non-specific serine/threonine protein kinase | PF00069: Protein kinase domain; PF03822: NAF domain | GO:0004672; GO:0005524; GO:0006468; GO:0007165 |
|  |  |  |  |  | TraesCS1B01G098800 | 3-N-debenzoyl-2-deoxytaxol N-benzoyltransferase | PF02458: Transferase family | GO:0004672; GO:0005524; GO:0006468; GO:0007165 |
|  |  |  |  |  | TraesCS1B01G098900 | BTB/POZ and MATH domain-containing protein 2 | NA | GO:0016747 |
|  |  |  |  |  | TraesCS1B01G099000 | ABC transporter B family protein | PF00664: ABC transporter transmembrane region; PF00005: ABC transporter | GO:0005515 |
|  |  |  |  |  | TraesCS1B01G099100 | Diacylglycerol acyltransferase | NA | GO:0004601; GO:0006979; GO:0020037; GO:0055114 |
|  |  |  |  |  | TraesCS1B01G099200 | Peroxidase | PF00141: Peroxidase | GO:0004601; GO:0006979; GO:0020037; GO:0055114 |
|  |  |  |  |  | TraesCS1B01G099300 | Peroxidase | PF00141: Peroxidase | NA |
| Relative_RFW | BS00084305_51 |  | 1B | 90992706 | TraesCS1B01G089500 | BTB/POZ domain containing protein | PF00651: BTB/POZ domain | GO:0005515 MF: protein binding |
|  |  |  |  |  | TraesCS1B01G089600 | electron protein, putative (Protein of unknown function, DUF547) | PF14389: Leucine-zipper of ternary complex factor MIP1; PF04784: Protein of unknown function, DUF547 | NA |
|  |  |  |  |  | TraesCS1B01G089700 | ATP-dependent zinc metalloprotease FtsH | PF00004: ATPase family associated with various cellular activities (AAA) | GO:0005524 MF: ATP binding |
|  |  |  |  |  | TraesCS1B01G089800 | Mitochondrial pyruvate carrier | PF03650: Uncharacterised protein family (UPF0041) | GO:0005743 CC: mitochondrial inner membrane;GO:0006850 BP: mitochondrial pyruvate transport |
|  |  |  |  |  | TraesCS1B01G089900 | phosphoglycolate phosphatase | PF12452: Protein of unknown function (DUF3685) | NA |
|  |  |  |  |  | TraesCS1B01G090000 | Mitochondrial pyruvate carrier | PF03650: Uncharacterised protein family (UPF0041) | GO:0005743 CC: mitochondrial inner membrane;GO:0006850 BP: mitochondrial pyruvate transport |
|  |  |  |  |  | TraesCS1B01G090100 | Receptor-like protein kinase | PF07714: Protein tyrosine kinase | GO:0004672 MF: protein kinase activity;GO:0005524 MF: ATP binding; |
|  |  |  |  |  | TraesCS1B01G090200 | Wall-associated receptor kinase 2 | PF13947: Wall-associated receptor kinase galacturonan-binding | GO:0030247 MF: polysaccharide binding |
|  |  |  |  |  | TraesCS1B01G090300 | Carotenoid isomerase, putative, expressed | PF01593: Flavin containing amine oxidoreductase | GO:0016117 BP: carotenoid biosynthetic process;GO:0016491 MF: oxidoreductase activity; |
|  |  |  |  |  | TraesCS1B01G090400 | Metal tolerance protein | PF01545: Cation efflux family | GO:0006812 BP: cation transport;GO:0008324 MF: cation transmembrane transporter activity; |
|  |  |  |  |  | TraesCS1B01G090500 | Glutamate--cysteine ligase, chloroplastic | PF04107: Glutamate-cysteine ligase family 2(GCS2) | GO:0004357 MF: glutamate-cysteine ligase activity;GO:0006750 BP: glutathione biosynthetic process; |
|  |  |  |  |  | TraesCS1B01G090600 | Aminotransferase like protein | PF00202: Aminotransferase class-III | GO:0003824 MF: catalytic activity;GO:0006525 BP: arginine metabolic process; |
|  |  |  |  |  | TraesCS1B01G090700 | Endoglucanase | PF00759: Glycosyl hydrolase family 9 | GO:0003824 MF: catalytic activity;GO:0004553 MF: hydrolase activity, hydrolyzing O-glycosyl compounds; |
|  |  |  |  |  | TraesCS1B01G090800 | Ribonucleoside-diphosphate reductase | NA | NA |
| Relative root fresh weight | BS00087787_51 |  | 1B | 50778549 | TraesCS1B01G065200 | NADH-ubiquinone oxidoreductase chain 1 | PF00146: NADH dehydrogenase | GO:0016020 CC: membrane;GO:0055114 BP: oxidation-reduction process |
|  |  |  |  |  | TraesCS1B01G065300 | ATP synthase subunit a | PF00119: ATP synthase A chain | GO:0015078 MF: hydrogen ion transmembrane transporter activity;GO:0015986 BP: ATP synthesis coupled proton transport; |
|  |  |  |  |  | TraesCS1B01G065400 | 26S proteasome non-ATPase regulatory subunit-like protein | NA | NA |
|  |  |  |  |  | TraesCS1B01G065500 | Cytochrome c biogenesis Fc | NA | NA |
|  |  |  |  |  | TraesCS1B01G065600 | NADH-ubiquinone oxidoreductase chain 1 | PF00146: NADH dehydrogenase | GO:0016020 CC: membrane;GO:0055114 BP: oxidation-reduction process |
|  |  |  |  |  | TraesCS1B01G065700 | Nuclear pore complex protein Nup107 | PF04121: Nuclear pore protein 84 / 107 | GO:0005643 CC: nuclear pore;GO:0006810 BP: transport |
|  |  |  |  |  | TraesCS1B01G065800 | E3 ubiquitin-protein ligase RNF14 | PF05773: RWD domain; PF01485: IBR domain, a half RING-finger domain | GO:0003676 MF: nucleic acid binding;GO:0005515 MF: protein binding; |
|  |  |  |  |  | TraesCS1B01G065900 | Arginine/serine-rich splicing factor, putative | PF00076: RNA recognition motif. (a.k.a. RRM, RBD, or RNP domain); PF00098: Zinc knuckle | GO:0003676 MF: nucleic acid binding;GO:0008270 MF: zinc ion binding |
|  |  |  |  |  | TraesCS1B01G066000 | ABC transporter G family member | PF00005: ABC transporter; PF01061: ABC-2 type transporter | GO:0005524 MF: ATP binding;GO:0016020 CC: membrane; |
|  |  |  |  |  | TraesCS1B01G066100 | DNA ligase | PF03017: TNP1/EN/SPM transposase | NA |
|  |  |  |  |  | TraesCS1B01G066200 | Protein FATTY ACID EXPORT 4, chloroplastic | NA | NA |
|  |  |  |  |  | TraesCS1B01G066300 | Peptidyl-prolyl cis-trans isomerase | PF04979: Protein phosphatase inhibitor 2 (IPP-2) | GO:0004864 MF: protein phosphatase inhibitor activity;GO:0009966 BP: regulation of signal transduction; |
|  |  |  |  |  | TraesCS1B01G066400 | Alcohol dehydrogenase, putative | PF08240: Alcohol dehydrogenase GroES-like domain; PF00107: Zinc-binding dehydrogenase | GO:0008270 MF: zinc ion binding;GO:0016491 MF: oxidoreductase activity; |
|  |  |  |  |  | TraesCS1B01G066500 | Tryptophan decarboxylase | PF00282: Pyridoxal-dependent decarboxylase conserved domain | GO:0003824 MF: catalytic activity;GO:0006520 BP: cellular amino acid metabolic process; |
|  |  |  |  |  | TraesCS1B01G066600 | Tryptophan decarboxylase | PF00282: Pyridoxal-dependent decarboxylase conserved domain | GO:0019752 BP: carboxylic acid metabolic process;GO:0030170 MF: pyridoxal phosphate binding |
|  |  |  |  |  | TraesCS1B01G066700 | Pentatricopeptide repeat-containing protein | PF13812: Pentatricopeptide repeat domain; PF01535: PPR repeat | NA |
|  |  |  |  |  | TraesCS1B01G066800 | 2-phytyl-1,4-naphtoquinone methyltransferase | NA | NA |
|  |  |  |  |  | TraesCS1B01G066900 | Bidirectional sugar transporter SWEET | PF03083: Sugar efflux transporter for intercellular exchange | GO:0016021 CC: integral component of membrane |
|  |  |  |  |  | TraesCS1B01G067000 | Defensin-like protein | PF00304: Gamma-thionin family | GO:0006952 BP: defense response |
|  |  |  |  |  | TraesCS1B01G067100 | Defensin | PF00304: Gamma-thionin family | GO:0006952 BP: defense response |
|  |  |  |  |  | TraesCS1B01G067200 | NA | NA | NA |
|  |  |  |  |  | TraesCS1B01G067200 | LysM domain receptor-like kinase 3 | NA | NA |
|  |  |  |  |  | TraesCS1B01G067300 | Defensin | PF00304: Gamma-thionin family | GO:0006952 BP: defense response |
|  |  |  |  |  | TraesCS1B01G067400 | F-box protein | NA | GO:0005515 MF: protein binding |
| Relative root fresh weight | Excalibur_c7954_672 and | Rel_RFW_1B_Hap1 | 1B | 117170305 | TraesCS1B01G105300 | O-acyltransferase | PF03062: MBOAT, membrane-bound O-acyltransferase family | NA |
|  | Ku_c3695_1696 | Rel_RFW_1B_Hap1 | 1B | 117188717 |  |  |  |  |
| Relative root fresh weight | Kukri_c29582_126 |  | 1B | 118510327 | TraesCS1B01G106700 | ATP-dependent RNA helicase, putative | PF00271: Helicase conserved C-terminal domain; PF00642: Zinc finger C-x8-C-x5-C-x3-H type (and similar) | GO:0046872 MF: metal ion binding |
|  |  |  |  |  | TraesCS1B01G106800 | Zinc finger homeodomain protein | PF04770: ZF-HD protein dimerisation region | GO:0003677 MF: DNA binding |
|  |  |  |  |  | TraesCS1B01G106900 | NA | NA | NA |
|  |  |  |  |  | TraesCS1B01G107000 | Serine/threonine-protein phosphatase | PF13415: Galactose oxidase, central domain; PF00149: Calcineurin-like phosphoesterase | GO:0004721 MF: phosphoprotein phosphatase activity;GO:0005515 MF: protein binding; |
|  |  |  |  |  | TraesCS1B01G107100 | Ankyrin repeat domain-containing protein 2 | PF00023: Ankyrin repeat; PF13637: Ankyrin repeats (many copies); PF13920: Zinc finger, C3HC4 type (RING finger) | GO:0005515 MF: protein binding;GO:0008270 MF: zinc ion binding |
|  |  |  |  |  | TraesCS1B01G107200 | DNA repair helicase | PF06733: DEAD_2; PF06777: Helical and beta-bridge domain; PF13307: Helicase C-terminal domain | GO:0003676 MF: nucleic acid binding;GO:0003677 MF: DNA binding; |
|  |  |  |  |  | TraesCS1B01G107300 | Agmatine coumaroyltransferase-1 | PF02458: Transferase family | GO:0016747 MF: transferase activity, transferring acyl groups other than amino-acyl groups |
|  |  |  |  |  | TraesCS1B01G107400 | Agmatine coumaroyltransferase-1 | PF02458: Transferase family | GO:0016747 MF: transferase activity, transferring acyl groups other than amino-acyl groups |
| Relative root fresh weight | Kukri_rep_c105316_262 |  | 1B |  | 230943735 | TraesCS1B01G150400 | NA | NA |
|  |  |  |  |  | TraesCS1B01G150500 | NA | NA | NA |
|  |  |  |  |  | TraesCS1B01G150600 | Zinc finger CCCH domain protein | PF00642: Zinc finger C-x8-C-x5-C-x3-H type (and similar) | GO:0046872 MF: metal ion binding |
|  |  |  |  |  | TraesCS1B01G150700 | NA | NA | NA |
|  |  |  |  |  | TraesCS1B01G150800 | Ion channel pollux-like protein | PF06241: Castor and Pollux, part of voltage-gated ion channel | NA |
| Relative root fresh weight | RAC875_c28894_526 and | Rel_RFW_1B_Hap2 | 1B | 106792444 | TraesCS1B01G099300 | polyadenylate-binding protein 1-B-binding protein | NA | NA |
|  | wsnp_Ex_c11976_19193550 | Rel_RFW_1B_Hap2 | 1B | 109729521 | TraesCS1B01G099400 | Polyadenylate-binding protein 1-B-binding protein | NA | NA |
|  |  |  |  |  | TraesCS1B01G099500 | polyadenylate-binding protein 1-B-binding protein | NA | NA |
|  |  |  |  |  | TraesCS1B01G099600 | Aspartic proteinase | PF00026: Eukaryotic aspartyl protease; PF03489: Saposin-like type B, region 2; PF05184: Saposin-like type B, region 1 | GO:0004190; GO:0006508; GO:0006629 |
|  |  |  |  |  | TraesCS1B01G099700 | Transmembrane protein, putative | NA | NA |
|  |  |  |  |  | TraesCS1B01G099800 | Thioesterase family protein | PF03061: Thioesterase superfamily | NA |
|  |  |  |  |  | TraesCS1B01G099900 | SsrA-binding | NA | NA |
|  |  |  |  |  | TraesCS1B01G100000 | Chitinase | PF00182: Chitinase class I | GO:0004568; GO:0005975; GO:0006032; GO:0016998 |
|  |  |  |  |  | TraesCS1B01G100100 | Low temperature and salt responsive protein | PF01679: Proteolipid membrane potential modulator | GO:0016021 |
|  |  |  |  |  | TraesCS1B01G100200 | Bushy growth protein | PF04855: SNF5 / SMARCB1 / INI1 | GO:0000228; GO:0006338 |
|  |  |  |  |  | TraesCS1B01G100300 | Auxin-responsive protein | PF02309: AUX/IAA family | GO:0005515 |
|  |  |  |  |  | TraesCS1B01G100400 | Basic helix-loop-helix transcription factor | PF00010: Helix-loop-helix DNA-binding domain | GO:0046983 |
|  |  |  |  |  | TraesCS1B01G100500 | RNA-binding (RRM/RBD/RNP motifs) family protein | NA | NA |
|  |  |  |  |  | TraesCS1B01G100600 | MYB transcription factor | PF00249: Myb-like DNA-binding domain | GO:0003677 |
|  |  |  |  |  | TraesCS1B01G100700 | Peptide chain release factor 1 | NA | NA |
|  |  |  |  |  | TraesCS1B01G100800 | RNA-binding family protein | PF00076: RNA recognition motif. (a.k.a. RRM, RBD, or RNP domain) | GO:0003676; GO:0003723; GO:0005634; GO:0005737; GO:0006396 |
|  |  |  |  |  | TraesCS1B01G100900 | 60S acidic ribosomal protein P0 | NA | NA |
|  |  |  |  |  | TraesCS1B01G101000 | Agenet domain containing protein | NA | NA |
|  |  |  |  |  | TraesCS1B01G101100 | Dehydrogenase | PF13561: Enoyl-(Acyl carrier protein) reductase | GO:0016491 |
|  |  |  |  |  | TraesCS1B01G101200 | F-box/RNI-like/FBD-like domains-containing protein | PF08387: FBD | NA |
|  |  |  |  |  | TraesCS1B01G101300 | Benzyl alcohol O-benzoyltransferase | PF02458: Transferase family | GO:0016747 |
|  |  |  |  |  | TraesCS1B01G101400 | Ubiquitinyl hydrolase 1 | NA | NA |
|  |  |  |  |  | TraesCS1B01G101500 | Benzyl alcohol O-benzoyltransferase | PF02458: Transferase family | GO:0016747 |
|  |  |  |  |  | TraesCS1B01G101600 | tRNA/rRNA methyltransferase family protein | PF00588: SpoU rRNA Methylase family | GO:0003723; GO:0006396; GO:0008173 |
|  |  |  |  |  | TraesCS1B01G101700 | RING/U-box superfamily protein, putative | PF13920: Zinc finger, C3HC4 type (RING finger) | GO:0005515; GO:0008270 |
|  |  |  |  |  | TraesCS1B01G101800 | 2,3-bisphosphoglycerate-dependent phosphoglycerate mutase | PF00300: Histidine phosphatase superfamily (branch 1) | GO:0003824; GO:0008152 |
|  |  |  |  |  | TraesCS1B01G101900 | embryo defective 2423 | NA | NA |
|  |  |  |  |  | TraesCS1B01G102000 | Heat-inducible transcription repressor HrcA | NA | NA |
|  |  |  |  |  | TraesCS1B01G102100 | F-box/RNI-like/FBD-like domains-containing protein | NA | NA |
| Relative root fresh weight | RAC875_c63067_283 |  | 1B | 95654954 | TraesCS1B01G093300 | G-patch domain containing protein | PF01424: R3H domain; PF01585: G-patch domain | GO:0003676 MF: nucleic acid binding |
|  |  |  |  |  | TraesCS1B01G093400 | Transmembrane protein, putative | NA | NA |
|  |  |  |  |  | TraesCS1B01G093500 | Pentatricopeptide repeat-containing protein | PF13041: PPR repeat family; PF01535: PPR repeat; PF12854: PPR repeat | GO:0005515 MF: protein binding |
|  |  |  |  |  | TraesCS1B01G093600 | Long-Chain Acyl-CoA Synthetase | PF00501: AMP-binding enzyme | GO:0003824 MF: catalytic activity;GO:0008152 BP: metabolic process |
|  |  |  |  |  | TraesCS1B01G093700 | Agenet domain-containing protein | PF05641: Agenet domain | NA |
|  |  |  |  |  | TraesCS1B01G093800 | Receptor-like protein kinase | PF00069: Protein kinase domain | GO:0004672 MF: protein kinase activity;GO:0005524 MF: ATP binding; |
|  |  |  |  |  | TraesCS1B01G093900 | DNA-directed RNA polymerase III subunit RPC3 | PF08221: RNA polymerase III subunit RPC82 helix-turn-helix domain; PF05645: RNA polymerase III subunit RPC82 | GO:0003677 MF: DNA binding;GO:0003899 MF: DNA-directed 5'-3' RNA polymerase activity; |
|  |  |  |  |  | TraesCS1B01G094000 | FBD-associated F-box protein | NA | GO:0005515 MF: protein binding |
|  |  |  |  |  | TraesCS1B01G094100 | Myb/SANT-like DNA-binding domain protein | NA | NA |
|  |  |  |  |  | TraesCS1B01G094200 | Formin-like protein | PF10409: C2 domain of PTEN tumour-suppressor protein; PF02181: Formin Homology 2 Domain | GO:0005515 MF: protein binding |
|  |  |  |  |  | TraesCS1B01G094300 | Phosphate translocator | PF03151: Triose-phosphate Transporter family | GO:0005215 MF: transporter activity;GO:0006810 BP: transport; |
|  |  |  |  |  | TraesCS1B01G094400 | Transmembrane protein 234 like | PF10639: Putative transmembrane family 234 | NA |
|  |  |  |  |  | TraesCS1B01G094500 | MYB transcription factor | PF00249: Myb-like DNA-binding domain | GO:0003677 MF: DNA binding |
|  |  |  |  |  | TraesCS1B01G094600 | Nitrogen regulatory protein P-II-like protein | PF00543: Nitrogen regulatory protein P-II | GO:0006808 BP: regulation of nitrogen utilization;GO:0030234 MF: enzyme regulator activity |
|  |  |  |  |  | TraesCS1B01G094700 | O-fucosyltransferase family protein | PF10250: GDP-fucose protein O-fucosyltransferase | NA |
| Relative shoot length | AX-158521438 |  | 1B | 6867170 | TraesCS1B01G012100 | Pm3-like disease resistance protein | PF00931: NB-ARC domain | GO:0043531 MF: ADP binding |
|  |  |  |  |  | TraesCS1B01G012200 | Pm3-like disease resistance protein | PF00931: NB-ARC domain | GO:0043531 MF: ADP binding |
|  |  |  |  |  | TraesCS1B01G012300 | Ankyrin repeat protein family-like protein | PF12796: Ankyrin repeats (3 copies); PF13857: Ankyrin repeats (many copies); PF13962: Domain of unknown function | GO:0005515 MF: protein binding |
|  |  |  |  |  | TraesCS1B01G012400 | Pm3-like disease resistance protein | PF00931: NB-ARC domain | GO:0043531 MF: ADP binding |
|  |  |  |  |  | TraesCS1B01G012500 | Disease resistance protein (TIR-NBS-LRR class) family | PF00931: NB-ARC domain | GO:0043531 MF: ADP binding |
|  |  |  |  |  | TraesCS1B01G012600 | NBS-LRR class disease resistance protein | PF00931: NB-ARC domain | GO:0043531 MF: ADP binding |
|  |  |  |  |  | TraesCS1B01G012700 | Disease resistance protein (TIR-NBS-LRR class) family | PF00931: NB-ARC domain | GO:0043531 MF: ADP binding |
|  |  |  |  |  | TraesCS1B01G012800 | Pm3-like disease resistance protein | PF00931: NB-ARC domain | GO:0043531 MF: ADP binding |
|  |  |  |  |  | TraesCS1B01G012900 | Pm3-like disease resistance protein | PF00931: NB-ARC domain | GO:0043531 MF: ADP binding |
|  |  |  |  |  | TraesCS1B01G013000 | Disease resistance protein | NA | NA |
|  |  |  |  |  | TraesCS1B01G013100 | Dirigent protein | PF03018: Dirigent-like protein | NA |
|  |  |  |  |  | TraesCS1B01G013200 | Dirigent protein | PF01419: Jacalin-like lectin domain | NA |
|  |  |  |  |  | TraesCS1B01G013300 | Dirigent protein | PF03018: Dirigent-like protein; PF01419: Jacalin-like lectin domain | NA |
|  |  |  |  |  | TraesCS1B01G013500 | Low molecular weight glutenin subunit | PF13016: Cys-rich Gliadin N-terminal | GO:0045735 MF: nutrient reservoir activity |
|  |  |  |  |  | TraesCS1B01G013600 | Dirigent protein | PF03018: Dirigent-like protein | NA |
|  |  |  |  |  | TraesCS1B01G013700 | disease resistance protein (TIR-NBS-LRR class) | NA | NA |
|  |  |  |  |  | TraesCS1B01G013800 | RING-H2 group F2A | NA | NA |
|  |  |  |  |  | TraesCS1B01G013900 | Ankyrin repeat family protein | PF12796: Ankyrin repeats (3 copies); PF13606: Ankyrin repeat | GO:0005515 MF: protein binding |
|  |  |  |  |  | TraesCS1B01G014000 | MICOS complex subunit Mic25 | NA | NA |
|  |  |  |  |  | TraesCS1B01G014100 | Pm3-like disease resistance protein | PF00931: NB-ARC domain | GO:0005515 MF: protein binding;GO:0043531 MF: ADP binding |
|  |  |  |  |  | TraesCS1B01G014200 | Dirigent protein | PF03018: Dirigent-like protein; PF01419: Jacalin-like lectin domain | NA |
|  |  |  |  |  | TraesCS1B01G014300 | HXXXD-type acyl-transferase family protein | PF02458: Transferase family | GO:0016747 MF: transferase activity, transferring acyl groups other than amino-acyl groups |
|  |  |  |  |  | TraesCS1B01G014400 | disease resistance family protein / LRR family protein | PF00931: NB-ARC domain | GO:0043531 MF: ADP binding |
|  |  |  |  |  | TraesCS1B01G014500 | disease resistance family protein / LRR family protein | PF00931: NB-ARC domain | GO:0043531 MF: ADP binding |
|  |  |  |  |  | TraesCS1B01G014600 | Dirigent protein | PF03018: Dirigent-like protein; PF01419: Jacalin-like lectin domain | NA |
|  |  |  |  |  | TraesCS1B01G014700 | Dirigent protein | PF03018: Dirigent-like protein | NA |
|  |  |  |  |  | TraesCS1B01G014800 | Pm3-like disease resistance protein | PF00931: NB-ARC domain | GO:0043531 MF: ADP binding |
|  |  |  |  |  | TraesCS1B01G014900 | Pm3-like disease resistance protein | PF00931: NB-ARC domain | GO:0043531 MF: ADP binding |
|  |  |  |  |  | TraesCS1B01G015000 | Disease resistance protein | NA | NA |
|  |  |  |  |  | TraesCS1B01G015100 | S-locus lectin protein kinase family protein | NA | NA |
|  |  |  |  |  | TraesCS1B01G015200 | Myb/SANT-like DNA-binding domain protein | PF12776: Myb/SANT-like DNA-binding domain | NA |
|  |  |  |  |  | TraesCS1B01G015300 | Pm3-like disease resistance protein | PF00931: NB-ARC domain | GO:0043531 MF: ADP binding |
|  |  |  |  |  | TraesCS1B01G015400 | SNF2 domain-containing protein / helicase domain-containing protein / zinc finger protein-like protein | NA | NA |
|  |  |  |  |  | TraesCS1B01G015500 | cysteine-rich RLK (RECEPTOR-like protein kinase) 37 | NA | NA |
|  |  |  |  |  | TraesCS1B01G015600 | S-locus lectin protein kinase family protein | NA | NA |
|  |  |  |  |  | TraesCS1B01G015700 | S-locus lectin protein kinase family protein | NA | NA |
|  |  |  |  |  | TraesCS1B01G015800 | Dirigent protein | PF03018: Dirigent-like protein | NA |
|  |  |  |  |  | TraesCS1B01G015900 | Cytochrome P450 | PF00067: Cytochrome P450 | GO:0005506 MF: iron ion binding;GO:0016705 MF: oxidoreductase activity, acting on paired donors, with incorporation or reduction of molecular oxygen; |
|  |  |  |  |  | TraesCS1B01G016000 | Pm3-like disease resistance protein | PF00931: NB-ARC domain | GO:0043531 MF: ADP binding |
|  |  |  |  |  | TraesCS1B01G016100 | Pm3-like disease resistance protein | PF00931: NB-ARC domain | GO:0043531 MF: ADP binding |
| Relative shoot length | AX-158540096 | Rel_SL_1B_Hap1 | 1B | 671198412 | TraesCS1B01G456400 | S-type anion channel | PF03595: Voltage-dependent anion channel | GO:0016021 CC: integral component of membrane;GO:0055085 BP: transmembrane transport |
| Relative shoot length | AX-158560878 | Rel_SL_1B_Hap1 | 1B | 670931058 | TraesCS1B01G456500 | S-type anion channel | PF03595: Voltage-dependent anion channel | GO:0016021 CC: integral component of membrane;GO:0055085 BP: transmembrane transport |
| Relative root fresh weight | Tdurum_contig94450_255 | Rel_SL_1B_Hap1 | 1B | 671199253 | TraesCS1B01G456600 | Protein ABIL1 | NA | NA |
| Relative root shoot weight | Kukri_c29170_680 |  | 2A | 693293427 | TraesCS2A01G442300 | p-loop containing nucleoside triphosphate hydrolases superfamily protein, putative | PF13086: AAA domain; PF13087: AAA domain; PF00580: UvrD/REP helicase N-terminal domain | GO:0005515 MF: protein binding;GO:0005524 MF: ATP binding |
|  |  |  |  |  | TraesCS2A01G442400 | Phospholipid scramblase | PF03803: Scramblase | NA |
|  |  |  |  |  | TraesCS2A01G442500 | Basic helix loop helix (BHLH) family transcription factor | PF00010: Helix-loop-helix DNA-binding domain | GO:0046983 MF: protein dimerization activity |
|  |  |  |  |  | TraesCS2A01G442600 | Glycosyltransferase | PF04577: Protein of unknown function (DUF563) | GO:0016757 MF: transferase activity, transferring glycosyl groups |
|  |  |  |  |  | TraesCS2A01G442700 | Basic helix-loop-helix (BHLH) Transcription Factor | PF00010: Helix-loop-helix DNA-binding domain | GO:0046983 MF: protein dimerization activity |
|  |  |  |  |  | TraesCS2A01G442800 | Tudor/PWWP/MBT superfamily protein | PF00855: PWWP domain | NA |
|  |  |  |  |  | TraesCS2A01G442900 | Receptor-like protein kinase | PF13947: Wall-associated receptor kinase galacturonan-binding; PF08488: Wall-associated kinase; PF07714: Protein tyrosine kinase | GO:0004672 MF: protein kinase activity;GO:0004674 MF: protein serine/threonine kinase activity; |
|  |  |  |  |  | TraesCS2A01G443000 | Kinase, putative | PF00069: Protein kinase domain | GO:0004672 MF: protein kinase activity;GO:0005524 MF: ATP binding; |
|  |  |  |  |  | TraesCS2A01G443100 | LEAFY-like protein | PF01698: Floricaula / Leafy protein | GO:0003677 MF: DNA binding;GO:0006355 BP: regulation of transcription, DNA-templated |
|  |  |  |  |  | TraesCS2A01G443200 | 50S ribosomal protein L11 | PF03946: Ribosomal protein L11, N-terminal domain; PF00298: Ribosomal protein L11, RNA binding domain | GO:0003735 MF: structural constituent of ribosome;GO:0005840 CC: ribosome; |
|  |  |  |  |  | TraesCS2A01G443300 | Seed specific protein Bn15D1B | NA | NA |
|  |  |  |  |  | TraesCS2A01G443400 | Translation initiation factor IF-2 | NA | NA |
|  |  |  |  |  | TraesCS2A01G443500 | F-box domain containing protein, expressed | NA | NA |
|  |  |  |  |  | TraesCS2A01G443600 | Peptide transporter | PF00854: POT family | GO:0005215 MF: transporter activity;GO:0006810 BP: transport; |
|  |  |  |  |  | TraesCS2A01G443700 | Peptide transporter | PF00854: POT family | GO:0005215 MF: transporter activity;GO:0006810 BP: transport; |
|  |  |  |  |  | TraesCS2A01G443800 | WRKY transcription factor | PF03106: WRKY DNA -binding domain | GO:0003700 MF: transcription factor activity, sequence-specific DNA binding;GO:0006355 BP: regulation of transcription, DNA-templated; |
| Relative_SL | AX-158532334 |  | 2D | 534958538 | TraesCS2D01G419900 | GRF zinc finger protein | PF06839: GRF zinc finger | GO:0008270 MF: zinc ion binding |
|  |  |  |  |  | TraesCS2D01G420000 | GRF zinc finger-containing protein-like protein | PF06839: GRF zinc finger | GO:0008270 MF: zinc ion binding |
|  |  |  |  |  | TraesCS2D01G420100 | SKP1-like protein | PF03931: Skp1 family, tetramerisation domain; PF01466: Skp1 family, dimerisation domain | GO:0006511 BP: ubiquitin-dependent protein catabolic process |
|  |  |  |  |  | TraesCS2D01G420200 | BURP domain protein RD22 | PF03181: BURP domain | NA |
|  |  |  |  |  | TraesCS2D01G420300 | B3 domain-containing protein LOC_Os12g40080 | NA | NA |
|  |  |  |  |  | TraesCS2D01G420400 | ABC subfamily C transporter | PF00664: ABC transporter transmembrane region; PF00005: ABC transporter | GO:0006810 BP: transport;GO:0016021 CC: integral component of membrane; |
|  |  |  |  |  | TraesCS2D01G420500 | Armadillo/beta-catenin-like repeat family protein, expressed | NA | GO:0005488 MF: binding;GO:0005515 MF: protein binding |
|  |  |  |  |  | TraesCS2D01G420600 | NA | NA | NA |
|  |  |  |  |  | TraesCS2D01G420700 | Cysteine proteinases superfamily protein | NA | NA |
|  |  |  |  |  | TraesCS2D01G420800 | F-box family protein | PF12937: F-box-like | GO:0005515 MF: protein binding |
|  |  |  |  |  | TraesCS2D01G420900 | Pathogenesis-related protein 1 | PF00407: Pathogenesis-related protein Bet v I family | GO:0006952 BP: defense response;GO:0009607 BP: response to biotic stimulus |
|  |  |  |  |  | TraesCS2D01G421000 | Werner Syndrome-like exonuclease | PF01612: 3'-5' exonuclease | GO:0003676 MF: nucleic acid binding;GO:0006139 BP: nucleobase-containing compound metabolic process; |
|  |  |  |  |  | TraesCS2D01G421100 | Methyl-CpG-binding domain protein | PF01429: Methyl-CpG binding domain | GO:0003677 MF: DNA binding;GO:0005634 CC: nucleus |
|  |  |  |  |  | TraesCS2D01G421200 | UDP-N-acetylglucosamine pyrophosphorylase | PF01704: UTP--glucose-1-phosphate uridylyltransferase | GO:0008152 BP: metabolic process;GO:0070569 MF: uridylyltransferase activity |
|  |  |  |  |  | TraesCS2D01G421300 | 30S ribosomal protein S17 | PF16205: Ribosomal_S17 N-terminal; PF00366: Ribosomal protein S17 | GO:0003735 MF: structural constituent of ribosome;GO:0005622 CC: intracellular; |
|  |  |  |  |  | TraesCS2D01G421400 | Cell differentiation protein rcd1, putative, expressed | PF04078: Cell differentiation family, Rcd1-like | GO:0005488 MF: binding |
|  |  |  |  |  | TraesCS2D01G421500 | L-aspartate oxidase | PF10184: Uncharacterized conserved protein (DUF2358) | NA |
|  |  |  |  |  | TraesCS2D01G421600 | Leucine-rich repeat receptor-like protein kinase family protein | PF08263: Leucine rich repeat N-terminal domain; PF00560: Leucine Rich Repeat | GO:0005515 MF: protein binding |
|  |  |  |  |  | TraesCS2D01G421700 | Polygalacturonase QRT3 | PF12708: Pectate lyase superfamily protein | NA |
|  |  |  |  |  | TraesCS2D01G421800 | DNA-directed RNA polymerase subunit beta | NA | NA |
|  |  |  |  |  | TraesCS2D01G421900 | F-box family protein | NA | GO:0005515 MF: protein binding |
|  |  |  |  |  | TraesCS2D01G422000 | Zinc transporter | PF02535: ZIP Zinc transporter | GO:0005385 MF: zinc ion transmembrane transporter activity;GO:0016020 CC: membrane; |
|  |  |  |  |  | TraesCS2D01G422100 | Vacuolar ATPase assembly integral membrane protein VMA21 homolog | PF09446: VMA21-like domain | GO:0070072 BP: vacuolar proton-transporting V-type ATPase complex assembly |
|  |  |  |  |  | TraesCS2D01G422200 | Pentatricopeptide repeat-containing protein | PF01535: PPR repeat; PF17177: Pentacotripeptide-repeat region of PROPR | GO:0005515 MF: protein binding |
|  |  |  |  |  | TraesCS2D01G422300 | Cytochrome P450 | PF00067: Cytochrome P450 | GO:0005506 MF: iron ion binding;GO:0016705 MF: oxidoreductase activity, acting on paired donors, with incorporation or reduction of molecular oxygen; |
|  |  |  |  |  | TraesCS2D01G422400 | Alcohol dehydrogenase, putative | PF08240: Alcohol dehydrogenase GroES-like domain; PF00107: Zinc-binding dehydrogenase | GO:0008270 MF: zinc ion binding;GO:0016491 MF: oxidoreductase activity; |
|  |  |  |  |  | TraesCS2D01G422500 | Ribosome-recycling factor | NA | NA |
|  |  |  |  |  | TraesCS2D01G422600 | Coatomer subunit epsilon | PF04733: Coatomer epsilon subunit | GO:0005198 MF: structural molecule activity;GO:0005515 MF: protein binding; |
|  |  |  |  |  | TraesCS2D01G422700 | Bifunctional inhibitor/lipid-transfer protein/seed storage 2Salbumin superfamily protein | PF14547: Hydrophobic seed protein | NA |
| Relative shoot length | AX-158521912 |  | 2D | 542057320 | TraesCS2D01G429400 | Serine/arginine repetitive matrix protein 2, putative isoform 1 | NA | NA |
|  |  |  |  |  | TraesCS2D01G429500 | Ubiquitin-specific protease family C19-related protein | NA | NA |
|  |  |  |  |  | TraesCS2D01G429600 | Ubiquitin-specific protease family C19 protein | NA | NA |
|  |  |  |  |  | TraesCS2D01G429700 | Cytochrome P450 | PF00067: Cytochrome P450 | GO:0005506 MF: iron ion binding;GO:0016705 MF: oxidoreductase activity, acting on paired donors, with incorporation or reduction of molecular oxygen |
|  |  |  |  |  | TraesCS2D01G429800 | DNA ligase | PF04675: DNA ligase N terminus; PF01068: ATP dependent DNA ligase domain; PF04679: ATP dependent DNA ligase C terminal region; PF16589: BRCT domain, a BRCA1 C-terminus domain; PF11411: DNA ligase IV | GO:0003677 MF: DNA binding;GO:0003909 MF: DNA ligase activity; |
|  |  |  |  |  | TraesCS2D01G429900 | ELKS/Rab6-interacting/CAST family protein | NA | GO:0005515 MF: protein binding |
|  |  |  |  |  | TraesCS2D01G430000 | alpha-1,2-Mannosidase | PF01532: Glycosyl hydrolase family 47 | GO:0004571 MF: mannosyl-oligosaccharide 1,2-alpha-mannosidase activity;GO:0005509 MF: calcium ion binding; |
|  |  |  |  |  | TraesCS2D01G430100 | DNA ligase | NA | NA |
|  |  |  |  |  | TraesCS2D01G430200 | DNA ligase | NA | NA |
|  |  |  |  |  | TraesCS2D01G430300 | 30S ribosomal protein S10 | NA | NA |
|  |  |  |  |  | TraesCS2D01G430400 | 30S ribosomal protein S10 | NA | NA |
|  |  |  |  |  | TraesCS2D01G430500 | 60S ribosomal protein L7 | PF08079: Ribosomal L30 N-terminal domain; PF00327: Ribosomal protein L30p/L7e | NA |
| Relative root shoot ratio | AX-158523313 |  | 3A | 462336775 | TraesCS3A01G246300 | Serine/threonine-protein kinase | PF01453: D-mannose binding lectin; PF00954: S-locus glycoprotein domain; PF00069: Protein kinase domain | GO:0004672 MF: protein kinase activity;GO:0004674 MF: protein serine/threonine kinase activity;GO:0005524 MF: ATP binding;GO:0006468 BP: protein phosphorylation;GO:0048544 BP: recognition of pollen |
| Relative root shoot ratio | AX-158523668 |  | 3A | 444726186 | TraesCS3A01G238100 | NAD-dependent deacetylase sirtuin-6 | NA | NA |
|  |  |  |  |  | TraesCS3A01G238200 | Arabinogalactan peptide-like protein | PF06376: Arabinogalactan peptide | NA |
|  |  |  |  |  | TraesCS3A01G238300 | Ethylene-responsive transcription factor, putative | PF00847: AP2 domain | GO:0003677 MF: DNA binding;GO:0003700 MF: transcription factor activity, sequence-specific DNA binding; |
| Relative root shoot ratio | AX-158533093 |  | 3A | 444292176 | TraesCS3A01G232800 | Pentatricopeptide repeat-containing protein | PF13041: PPR repeat family; PF01535: PPR repeat | GO:0005515 MF: protein binding |
|  |  |  |  |  | TraesCS3A01G232900 | NA | NA | NA |
|  |  |  |  |  | TraesCS3A01G233000 | DELLA protein GAI | PF03514: GRAS domain family | NA |
|  |  |  |  |  | TraesCS3A01G233100 | F-box protein | PF00646: F-box domain | GO:0005515 MF: protein binding |
|  |  |  |  |  | TraesCS3A01G233200 | NA | NA | NA |
|  |  |  |  |  | TraesCS3A01G233300 | ADP,ATP carrier protein | PF03219: TLC ATP/ADP transporter | GO:0005471 MF: ATP:ADP antiporter activity;GO:0005524 MF: ATP binding; |
|  |  |  |  |  | TraesCS3A01G233400 | NA | NA | NA |
|  |  |  |  |  | TraesCS3A01G233500 | NA | NA | NA |
|  |  |  |  |  | TraesCS3A01G233600 | zinc ion binding protein | NA | NA |
|  |  |  |  |  | TraesCS3A01G233700 | Alpha/beta hydrolase-like | PF12697: Alpha/beta hydrolase family | NA |
|  |  |  |  |  | TraesCS3A01G233800 | Potassium channel | PF00520: Ion transport protein; PF00027: Cyclic nucleotide-binding domain; PF12796: Ankyrin repeats (3 copies); PF11834: KHA, dimerisation domain of potassium ion channel | GO:0005216 MF: ion channel activity;GO:0005249 MF: voltage-gated potassium channel activity; |
|  |  |  |  |  | TraesCS3A01G233900 | Transcription initiation factor TFIID subunit 7 | PF04658: TAFII55 protein conserved region | GO:0005669 CC: transcription factor TFIID complex;GO:0006367 BP: transcription initiation from RNA polymerase II promoter |
|  |  |  |  |  | TraesCS3A01G234000 | Transcription factor GTE4-like protein | PF00439: Bromodomain | GO:0005515 MF: protein binding |
|  |  |  |  |  | TraesCS3A01G234100 | Transcription factor GTE4-like protein | PF00439: Bromodomain | GO:0005515 MF: protein binding |
|  |  |  |  |  | TraesCS3A01G234200 | PGL/p-HBAD biosynthesis glycosyltransferase MT3031 | NA | NA |
|  |  |  |  |  | TraesCS3A01G234300 | Elongation factor G | NA | NA |
|  |  |  |  |  | TraesCS3A01G234400 | Transcription initiation factor TFIID subunit 7 | PF04658: TAFII55 protein conserved region | GO:0005669 CC: transcription factor TFIID complex;GO:0006367 BP: transcription initiation from RNA polymerase II promoter |
|  |  |  |  |  | TraesCS3A01G234500 | Transcription factor GTE4 | PF00439: Bromodomain | GO:0005044 MF: scavenger receptor activity;GO:0005515 MF: protein binding; |
|  |  |  |  |  | TraesCS3A01G234600 | Transcription factor GTE4-like protein | PF00439: Bromodomain | GO:0005515 MF: protein binding |
|  |  |  |  |  | TraesCS3A01G234700 | WD repeat protein | PF04003: Dip2/Utp12 Family | GO:0005515 MF: protein binding |
|  |  |  |  |  | TraesCS3A01G234800 | Malate dehydrogenase | PF00056: lactate/malate dehydrogenase, NAD binding domain; PF02866: lactate/malate dehydrogenase, alpha/beta C-terminal domain | GO:0003824 MF: catalytic activity;GO:0005975 BP: carbohydrate metabolic process; |
|  |  |  |  |  | TraesCS3A01G234900 | GDSL esterase/lipase | PF00657: GDSL-like Lipase/Acylhydrolase | GO:0016788 MF: hydrolase activity, acting on ester bonds |
|  |  |  |  |  | TraesCS3A01G235000 | GDSL esterase/lipase | PF00657: GDSL-like Lipase/Acylhydrolase | GO:0016788 MF: hydrolase activity, acting on ester bonds |
|  |  |  |  |  | TraesCS3A01G235100 | GDSL esterase/lipase | PF00657: GDSL-like Lipase/Acylhydrolase | GO:0006629 BP: lipid metabolic process;GO:0016298 MF: lipase activity; |
|  |  |  |  |  | TraesCS3A01G235200 | Pentatricopeptide repeat-containing protein | PF01535: PPR repeat; PF13812: Pentatricopeptide repeat domain; PF14432: DYW family of nucleic acid deaminases | GO:0005515 MF: protein binding;GO:0008270 MF: zinc ion binding |
|  |  |  |  |  | TraesCS3A01G235300 | Phospholipase A1 | PF01764: Lipase (class 3) | GO:0006629 BP: lipid metabolic process |
|  |  |  |  |  | TraesCS3A01G235400 | Phospholipase A1 | PF01764: Lipase (class 3) | GO:0006629 BP: lipid metabolic process |
|  |  |  |  |  | TraesCS3A01G235500 | GDSL esterase/lipase | PF00657: GDSL-like Lipase/Acylhydrolase | GO:0016788 MF: hydrolase activity, acting on ester bonds |
|  |  |  |  |  | TraesCS3A01G235600 | MBOAT (membrane bound O-acyl transferase) family protein | PF13813: Membrane bound O-acyl transferase family | NA |
|  |  |  |  |  | TraesCS3A01G235700 | Phospholipase A1 | PF01764: Lipase (class 3) | GO:0006629 BP: lipid metabolic process |
|  |  |  |  |  | TraesCS3A01G235800 | Protein CHUP1, chloroplastic | NA | NA |
|  |  |  |  |  | TraesCS3A01G235900 | Trichome birefringence-like protein | PF14416: PMR5 N terminal Domain; PF13839: GDSL/SGNH-like Acyl-Esterase family found in Pmr5 and Cas1p | NA |
|  |  |  |  |  | TraesCS3A01G236000 | DUF538 family protein (Protein of unknown function, DUF538) | PF04398: Protein of unknown function, DUF538 | NA |
|  |  |  |  |  | TraesCS3A01G236100 | zinc finger CCCH domain protein | NA | NA |
|  |  |  |  |  | TraesCS3A01G236200 | Trichome birefringence-like protein | PF14416: PMR5 N terminal Domain; PF13839: GDSL/SGNH-like Acyl-Esterase family found in Pmr5 and Cas1p | NA |
|  |  |  |  |  | TraesCS3A01G236300 | Trichome birefringence-like protein | PF14416: PMR5 N terminal Domain; PF13839: GDSL/SGNH-like Acyl-Esterase family found in Pmr5 and Cas1p | NA |
|  |  |  |  |  | TraesCS3A01G236400 | Glycogen synthase | PF13439: Glycosyltransferase Family 4; PF00534: Glycosyl transferases group 1 | NA |
|  |  |  |  |  | TraesCS3A01G236500 | Transducin/WD40 repeat-like superfamily protein | NA | GO:0005515 MF: protein binding |
|  |  |  |  |  | TraesCS3A01G236600 | Glutathione S-transferase T3 | NA | NA |
|  |  |  |  |  | TraesCS3A01G236700 | CTP synthase | PF06418: CTP synthase N-terminus; PF00117: Glutamine amidotransferase class-I | GO:0003883 MF: CTP synthase activity;GO:0006221 BP: pyrimidine nucleotide biosynthetic process |
|  |  |  |  |  | TraesCS3A01G236800 | Protease m50 membrane-bound transcription factor site 2 protease, putative | PF02163: Peptidase family M50 | GO:0004222 MF: metalloendopeptidase activity;GO:0006508 BP: proteolysis; |
|  |  |  |  |  | TraesCS3A01G236900 | Actin-related protein 2/3 complex subunit 2 | PF04045: Arp2/3 complex, 34 kD subunit p34-Arc | GO:0005885 CC: Arp2/3 protein complex;GO:0015629 CC: actin cytoskeleton; |
|  |  |  |  |  | TraesCS3A01G237000 | Seed maturation protein PM41 | NA | NA |
|  |  |  |  |  | TraesCS3A01G237100 | Isocitrate dehydrogenase [NADP] | PF00180: Isocitrate/isopropylmalate dehydrogenase | GO:0000287 MF: magnesium ion binding;GO:0004450 MF: isocitrate dehydrogenase (NADP+) activity; |
|  |  |  |  |  | TraesCS3A01G237200 | Histone-lysine N-methyltransferase | PF00855: PWWP domain; PF13831: PHD-finger; PF13832: PHD-zinc-finger like domain; PF00856: SET domain | GO:0005515 MF: protein binding;GO:0008270 MF: zinc ion binding |
|  |  |  |  |  | TraesCS3A01G237300 | Eukaryotic translation initiation factor 3 subunit 10-like | NA | NA |
|  |  |  |  |  | TraesCS3A01G237400 | Kinase family protein | PF00069: Protein kinase domain | GO:0004672 MF: protein kinase activity;GO:0005524 MF: ATP binding;GO:0006468 BP: protein phosphorylation |
|  |  |  |  |  | TraesCS3A01G237500 | NA | NA | NA |
|  |  |  |  |  | TraesCS3A01G237600 | Mediator complex, subunit Med7 | NA | NA |
|  |  |  |  |  | TraesCS3A01G237700 | Long-Chain Acyl-CoA Synthetase | PF00501: AMP-binding enzyme | GO:0003824 MF: catalytic activity;GO:0008152 BP: metabolic process |
| Relative_Rsratio | AX-158533132 |  | 3A | 477712324 | TraesCS3A01G254900 | Subtilisin-like protease | PF00082: Subtilase family; PF02225: PA domain | GO:0004252 MF: serine-type endopeptidase activity;GO:0006508 BP: proteolysis |
|  |  |  |  |  | TraesCS3A01G255000.6 | Sec14p-like phosphatidylinositol transfer family protein | PF03765: CRAL/TRIO, N-terminal domain; PF00650: CRAL/TRIO domain | NA |
|  |  |  |  |  | TraesCS3A01G255100 | Carboxyl methyltransferase | PF03492: SAM dependent carboxyl methyltransferase | GO:0008168 MF: methyltransferase activity |
|  |  |  |  |  | TraesCS3A01G255200 | Epoxide hydrolase 2 | PF00561: alpha/beta hydrolase fold | GO:0003824 MF: catalytic activity |
|  |  |  |  |  | TraesCS3A01G255300 | Bidirectional sugar transporter SWEET | PF03083: Sugar efflux transporter for intercellular exchange | GO:0016021 CC: integral component of membrane |
|  |  |  |  |  | TraesCS3A01G255400 | Sodium channel protein type 5 subunit alpha | NA | NA |
|  |  |  |  |  | TraesCS3A01G255500 | AWPM-19-like membrane family protein | PF05512: AWPM-19-like family | NA |
|  |  |  |  |  | TraesCS3A01G255600 | Protein kinase-like | PF00069: Protein kinase domain | GO:0004672 MF: protein kinase activity;GO:0005524 MF: ATP binding; |
|  |  |  |  |  | TraesCS3A01G255700 | Protein kinase-like | PF00069: Protein kinase domain | GO:0004672 MF: protein kinase activity;GO:0005524 MF: ATP binding; |
|  |  |  |  |  | TraesCS3A01G255800 | GMP synthase [glutamine-hydrolyzing] | PF02540: NAD synthase; PF00958: GMP synthase C terminal domain | GO:0003922 MF: GMP synthase (glutamine-hydrolyzing) activity;GO:0005524 MF: ATP binding; |
|  |  |  |  |  | TraesCS3A01G255900 | ABC transporter ATP-binding protein | PF00005: ABC transporter; PF06472: ABC transporter transmembrane region 2 | GO:0005524 MF: ATP binding;GO:0006810 BP: transport; |
|  |  |  |  |  | TraesCS3A01G256000 | RNA polymerase II transcriptional coactivator KELP | PF08766: DEK C terminal domain; PF02229: Transcriptional Coactivator p15 (PC4) | GO:0003677 MF: DNA binding;GO:0003713 MF: transcription coactivator activity; |
|  |  |  |  |  | TraesCS3A01G256100 | Proliferating cell nuclear antigen | PF00705: Proliferating cell nuclear antigen, N-terminal domain; PF02747: Proliferating cell nuclear antigen, C-terminal domain | GO:0003677 MF: DNA binding;GO:0006275 BP: regulation of DNA replication; |
|  |  |  |  |  | TraesCS3A01G256200 | Protein kinase-like | PF00069: Protein kinase domain | GO:0004672 MF: protein kinase activity;GO:0005524 MF: ATP binding; |
|  |  |  |  |  | TraesCS3A01G256300 | Protein kinase-like | PF00069: Protein kinase domain | GO:0004672 MF: protein kinase activity;GO:0005524 MF: ATP binding; |
|  |  |  |  |  | TraesCS3A01G256400 | Protein TIC 62, chloroplastic | PF13460: NAD(P)H-binding | NA |
|  |  |  |  |  | TraesCS3A01G256500 | NA | NA | NA |
|  |  |  |  |  | TraesCS3A01G256600 | Strictosidine synthase family protein | PF03088: Strictosidine synthase | GO:0009058 BP: biosynthetic process;GO:0016844 MF: strictosidine synthase activity |
|  |  |  |  |  | TraesCS3A01G256700 | FBD-associated F-box protein | PF08387: FBD | GO:0005515 MF: protein binding |
|  |  |  |  |  | TraesCS3A01G256800 | Flavin-containing monooxygenase | PF13434: L-lysine 6-monooxygenase (NADPH-requiring) | GO:0016491 MF: oxidoreductase activity;GO:0055114 BP: oxidation-reduction process |
|  |  |  |  |  | TraesCS3A01G256900 | SWAP (Suppressor-of-White-APricot)/surp domain-containing protein | PF09750: Alternative splicing regulator; PF01805: Surp module | GO:0003723 MF: RNA binding;GO:0006396 BP: RNA processing |
| Relative root shoot ratio | IAAV4343 |  | 3A | 444721312 | TraesCS3A01G237800 | Protein phosphatase 2c, putative | PF00481: Protein phosphatase 2C | GO:0003824 MF: catalytic activity;GO:0043169 MF: cation binding |
|  |  |  |  |  | TraesCS3A01G237900 | DNA repair protein RAD23 | NA | NA |
| Relative root shoot ratio | AX-158532834 | Rel_SFW_3A_Hap1 | 3A | 434839779 | TraesCS3A01G230200 | Exostosin family protein | PF03016: Exostosin family | NA |
|  |  |  |  |  | TraesCS3A01G230300 | CASP-like protein | PF04535: Domain of unknown function (DUF588) | NA |
|  |  |  |  |  | TraesCS3A01G230400 | Dual-specificity RNA methyltransferase RlmN | PF04055: Radical SAM superfamily; PF13394: 4Fe-4S single cluster domain | GO:0003824 MF: catalytic activity;GO:0005737 CC: cytoplasm; |
|  |  |  |  |  | TraesCS3A01G230500 | Kinase family protein | PF07714: Protein tyrosine kinase | GO:0004672 MF: protein kinase activity;GO:0005524 MF: ATP binding; |
|  |  |  |  |  | TraesCS3A01G230600 | HD domain-containing protein 2 | PF13023: HD domain | NA |
|  |  |  |  |  | TraesCS3A01G230700 | Bifunctional dihydroflavonol 4-reductase/flavanone 4-reductase isoform 2 | PF03661: Uncharacterised protein family (UPF0121) | GO:0016021 CC: integral component of membrane |
|  |  |  |  |  | TraesCS3A01G230800 | Spc97/Spc98 family of spindle pole body (SBP) component | NA | NA |
|  |  |  |  |  | TraesCS3A01G230900 | ATP-dependent zinc metalloprotease FtsH | PF14363: Domain associated at C-terminal with AAA; PF00004: ATPase family associated with various cellular activities (AAA) | GO:0005524 MF: ATP binding |
|  |  |  |  |  | TraesCS3A01G231000 | Isoprenylcysteine alpha-carbonyl methylesterase ICME | PF00135: Carboxylesterase family; PF00326: Prolyl oligopeptidase family | GO:0006508 BP: proteolysis;GO:0008236 MF: serine-type peptidase activity |
|  |  |  |  |  | TraesCS3A01G231100 | SWI/SNF-related matrix-associated actin-dependent regulator of chromatin subfamily A-like protein 1 | NA | NA |
|  |  |  |  |  | TraesCS3A01G231200 | F-box/kelch-repeat protein | NA | GO:0005515 MF: protein binding |
|  |  |  |  |  | TraesCS3A01G231300 | Protein MIZU-KUSSEI 1 | PF04759: Protein of unknown function, DUF617 | NA |
|  |  |  |  |  | TraesCS3A01G231400 | Single-stranded DNA-binding protein | PF00436: Single-strand binding protein family | GO:0003697 MF: single-stranded DNA binding |
|  |  |  |  |  | TraesCS3A01G231500 | Auxin efflux carrier component | PF03547: Membrane transport protein | GO:0016021 CC: integral component of membrane;GO:0055085 BP: transmembrane transport |
|  |  |  |  |  | TraesCS3A01G231600 | Homeobox associated leucine zipper protein | PF00046: Homeobox domain; PF02183: Homeobox associated leucine zipper | GO:0003677 MF: DNA binding;GO:0003700 MF: transcription factor activity, sequence-specific DNA binding; |
|  |  |  |  |  | TraesCS3A01G231700 | Mitogen-activated protein kinase | PF00069: Protein kinase domain | GO:0004672 MF: protein kinase activity;GO:0004707 MF: MAP kinase activity; |
|  |  |  |  |  | TraesCS3A01G231800 | ATP-dependent protease ATPase subunit HslU | NA | NA |
|  |  |  |  |  | TraesCS3A01G231900 | Tat pathway signal sequence family protein | NA | NA |
|  |  |  |  |  | TraesCS3A01G232000 | Cold-regulated protein | NA | NA |
|  |  |  |  |  | TraesCS3A01G232100 | Zinc finger protein ZPR1 | PF03367: ZPR1 zinc-finger domain | GO:0008270 MF: zinc ion binding |
|  |  |  |  |  | TraesCS3A01G232200 | CheY-like two-component responsive regulator family protein | NA | NA |
|  |  |  |  |  | TraesCS3A01G232300 | Elongation factor | PF00009: Elongation factor Tu GTP binding domain; PF03144: Elongation factor Tu domain 2; PF14492: Elongation Factor G, domain II; PF03764: Elongation factor G, domain IV; PF00679: Elongation factor G C-terminus | GO:0003924 MF: GTPase activity;GO:0005525 MF: GTP binding |
|  |  |  |  |  | TraesCS3A01G232400 | Metabotropic glutamate receptor 1 | NA | NA |
|  |  |  |  |  | TraesCS3A01G232500 | Zinc finger CCCH domain protein | PF00642: Zinc finger C-x8-C-x5-C-x3-H type (and similar) | GO:0046872 MF: metal ion binding |
|  |  |  |  |  | TraesCS3A01G232600 | Flavin-containing monooxygenase | PF00743: Flavin-binding monooxygenase-like | GO:0004499 MF: N,N-dimethylaniline monooxygenase activity;GO:0016491 MF: oxidoreductase activity; |
|  |  |  |  |  | TraesCS3A01G232700 | Sodium Bile acid symporter family | PF01758: Sodium Bile acid symporter family | GO:0016020 CC: membrane |
| Relative root fresh weight | IAAV902 |  | 3A | 574256873 | TraesCS3A01G328100 | Ethylene-responsive transcription factor, putative | PF00847: AP2 domain | GO:0003677 MF: DNA binding;GO:0003700 MF: transcription factor activity, sequence-specific DNA binding; |
|  |  |  |  |  | TraesCS3A01G328200 | Serine/Threonine-kinase WNK (WNK)-like protein | PF14215: bHLH-MYC and R2R3-MYB transcription factors N-terminal | NA |
|  |  |  |  |  | TraesCS3A01G328300 | Protein CHLOROPLAST ENHANCING STRESS TOLERANCE, chloroplastic | NA | NA |
|  |  |  |  |  | TraesCS3A01G328400 | Protein CHLOROPLAST ENHANCING STRESS TOLERANCE, chloroplastic | NA | NA |
|  |  |  |  |  | TraesCS3A01G328500 | Protein root hair specific 4 | NA | NA |
|  |  |  |  |  | TraesCS3A01G328600 | transmembrane protein, putative (DUF594) | PF13968: Domain of unknown function (DUF4220) | NA |
|  |  |  |  |  | TraesCS3A01G328700 | DNA-3-methyladenine glycosylase | PF03352: Methyladenine glycosylase | GO:0003824 MF: catalytic activity;GO:0006281 BP: DNA repair; |
|  |  |  |  |  | TraesCS3A01G328800 | Metacaspase | PF00656: Caspase domain | NA |
|  |  |  |  |  | TraesCS3A01G328900 | S-adenosylmethionine synthase | NA | NA |
|  |  |  |  |  | TraesCS3A01G329000 | S-adenosylmethionine synthase | NA | NA |
|  |  |  |  |  | TraesCS3A01G329100 | Transportin-1 | PF00161: Ribosome inactivating protein; PF03810: Importin-beta N-terminal domain | GO:0005488 MF: binding;GO:0006886 BP: intracellular protein transport; |
|  |  |  |  |  | TraesCS3A01G329200 | Serine carboxypeptidase family protein, expressed | PF00450: Serine carboxypeptidase | GO:0004185 MF: serine-type carboxypeptidase activity;GO:0006508 BP: proteolysis |
|  |  |  |  |  | TraesCS3A01G329300 | Polyadenylate-binding protein 1-B-binding protein | NA | NA |
|  |  |  |  |  | TraesCS3A01G329400 | MORC family CW-type zinc finger protein 4 | PF13589: Histidine kinase-, DNA gyrase B-, and HSP90-like ATPase | NA |
|  |  |  |  |  | TraesCS3A01G329500 | NADH-ubiquinone oxidoreductase chain 2 | NA | NA |
|  |  |  |  |  | TraesCS3A01G329600 | Pentatricopeptide repeat-containing protein | PF01535: PPR repeat; PF13041: PPR repeat family | GO:0005515 MF: protein binding |
|  |  |  |  |  | TraesCS3A01G329700 | Purple acid phosphatase | PF16656: Purple acid Phosphatase, N-terminal domain; PF00149: Calcineurin-like phosphoesterase; PF14008: Iron/zinc purple acid phosphatase-like protein C | GO:0003993 MF: acid phosphatase activity;GO:0016787 MF: hydrolase activity; |
|  |  |  |  |  | TraesCS3A01G329800 | Protease inhibitor/seed storage/lipid transfer protein family protein | NA | NA |
|  |  |  |  |  | TraesCS3A01G329900 | Protease inhibitor/seed storage/lipid transfer protein family protein | NA | NA |
|  |  |  |  |  | TraesCS3A01G330000 | F-box family protein | NA | NA |
|  |  |  |  |  | TraesCS3A01G330100 | 5-hydroxyisourate hydrolase 1 | NA | NA |
|  |  |  |  |  | TraesCS3A01G330200 | DNA-(apurinic or apyrimidinic site) lyase | PF02037: SAP domain; PF03372: Endonuclease/Exonuclease/phosphatase family | GO:0003677 MF: DNA binding;GO:0004518 MF: nuclease activity; |
|  |  |  |  |  | TraesCS3A01G330300 | DNA-(apurinic or apyrimidinic site) lyase | NA | GO:0003677 MF: DNA binding;GO:0004518 MF: nuclease activity; |
|  |  |  |  |  | TraesCS3A01G330400 | Oxidoreductase/ transition metal ion binding protein | NA | NA |
|  |  |  |  |  | TraesCS3A01G330500 | Beta-1,3-glucanase | PF00332: Glycosyl hydrolases family 17 | GO:0004553 MF: hydrolase activity, hydrolyzing O-glycosyl compounds;GO:0005975 BP: carbohydrate metabolic process |
| Relative root lengt | AX-109990240 |  | 3B | 115785002 | TraesCS3B01G132400 | 3'-N-debenzoyl-2'-deoxytaxol N-benzoyltransferase | PF02458: Transferase family | GO:0016747 MF: transferase activity, transferring acyl groups other than amino-acyl groups |
|  |  |  |  |  | TraesCS3B01G132500 | Extracellular ribonuclease | PF04231: Endonuclease I | GO:0004518 MF: nuclease activity |
|  |  |  |  |  | TraesCS3B01G132600 | regulatory particle non-ATPase subunit 5B | NA | NA |
|  |  |  |  |  | TraesCS3B01G132700 | Threonine--tRNA ligase | NA | NA |
|  |  |  |  |  | TraesCS3B01G132800 | Cysteine/Histidine-rich C1 domain family protein | PF07762: Protein of unknown function (DUF1618) | NA |
|  |  |  |  |  | TraesCS3B01G132900 | cDNA clone:001-038-D11, full insert sequence | NA | NA |
|  |  |  |  |  | TraesCS3B01G133000 | F-box protein | PF00646: F-box domain; PF03478: Protein of unknown function (DUF295) | GO:0005515 MF: protein binding |
|  |  |  |  |  | TraesCS3B01G133100 | Histone-lysine N-methyltransferase | PF00628: PHD-finger; PF13832: PHD-zinc-finger like domain | GO:0005515 MF: protein binding;GO:0008270 MF: zinc ion binding |
|  |  |  |  |  | TraesCS3B01G133200 | 60S ribosomal protein L18a-like protein | NA | NA |
|  |  |  |  |  | TraesCS3B01G133300 | Anthocyanidin 5,3-O-glucosyltransferase | NA | NA |
|  |  |  |  |  | TraesCS3B01G133400 | Alkaline alpha-galactosidase seed imbibition protein | PF05691: Raffinose synthase or seed imbibition protein Sip1 | GO:0003824 MF: catalytic activity |
|  |  |  |  |  | TraesCS3B01G133500 | gamma-irradiation and mitomycin c induced 1 | NA | NA |
|  |  |  |  |  | TraesCS3B01G133600 | Cytochrome P450 family protein, expressed | PF00067: Cytochrome P450 | GO:0005506 MF: iron ion binding;GO:0016705 MF: oxidoreductase activity, acting on paired donors, with incorporation or reduction of molecular oxygen;GO:0020037 MF: heme binding;GO:0055114 BP: oxidation-reduction process |
|  |  |  |  |  | TraesCS3B01G133700 | Cytochrome P450 family protein, expressed | PF00067: Cytochrome P450 | GO:0005506 MF: iron ion binding;GO:0016705 MF: oxidoreductase activity, acting on paired donors, with incorporation or reduction of molecular oxygen;GO:0020037 MF: heme binding;GO:0055114 BP: oxidation-reduction process |
|  |  |  |  |  | TraesCS3B01G133800 | P53/DNA damage-regulated protein | NA | NA |
|  |  |  |  |  | TraesCS3B01G133900 | Metacaspase-1 | PF00656: Caspase domain | NA |
|  |  |  |  |  | TraesCS3B01G134000 | Coatomer, beta subunit | PF10187: N-terminal domain of NEFA-interacting nuclear protein NIP30 | NA |
|  |  |  |  |  | TraesCS3B01G134100 | Cytochrome P450 family protein | PF00067: Cytochrome P450 | GO:0005506 MF: iron ion binding;GO:0016705 MF: oxidoreductase activity, acting on paired donors, with incorporation or reduction of molecular oxygen; |
|  |  |  |  |  | TraesCS3B01G134200 | Nuclear factor Y subunit C | PF00808: Histone-like transcription factor (CBF/NF-Y) and archaeal histone | GO:0046982 MF: protein heterodimerization activity |
|  |  |  |  |  | TraesCS3B01G134300 | Type I inositol-1,4,5-trisphosphate 5-phosphatase 1 | NA | GO:0046856 BP: phosphatidylinositol dephosphorylation |
|  |  |  |  |  | TraesCS3B01G134400 | Zinc finger family protein | PF13912: C2H2-type zinc finger | GO:0003676 MF: nucleic acid binding |
|  |  |  |  |  | TraesCS3B01G134500 | Zinc finger family protein | PF13912: C2H2-type zinc finger | GO:0003676 MF: nucleic acid binding |
|  |  |  |  |  | TraesCS3B01G134600 | Transducin/WD40 repeat-like superfamily protein | NA | NA |
|  |  |  |  |  | TraesCS3B01G134700 | Ankyrin repeat protein-like | PF12796: Ankyrin repeats (3 copies) | GO:0005515 MF: protein binding |
| Relative_root length | BobWhite_c10402_140 | Rel_RL_3B_Hap1 | 3B | 100968184 | TraesCS3B01G125400 | Pollen-specific protein SF21 | PF03096: Ndr family | NA |
| Relative root length | wsnp_JD_c30422_23944042 | Rel_RL_3B_Hap1 | 3B | 100965754 |  |  |  |  |
| Relative root length | wsnp_JD_c2623_3541255 |  | 3B | 114728184 | TraesCS3B01G131500 | Ubiquitin-conjugating enzyme E2-like protein | PF00179: Ubiquitin-conjugating enzyme | NA |
|  |  |  |  |  | TraesCS3B01G131600 | Double Clp-N motif-containing P-loop nucleoside triphosphate hydrolases superfamily protein | NA | NA |
|  |  |  |  |  | TraesCS3B01G131700.9 | Homeobox protein, putative | PF00046: Homeobox domain; PF02791: DDT domain; PF15612: WSTF, HB1, Itc1p, MBD9 motif 1; PF15613: Williams-Beuren syndrome DDT (WSD), D-TOX E motif | GO:0003677 MF: DNA binding |
|  |  |  |  |  | TraesCS3B01G131800 | Eukaryotic translation initiation factor 3 subunit C | NA | NA |
|  |  |  |  |  | TraesCS3B01G131900 | Phosducin, thioredoxin-like domain-containing protein | PF02114: Phosducin | NA |
|  |  |  |  |  | TraesCS3B01G132000 | FACT complex subunit SSRP1 | PF03531: Structure-specific recognition protein (SSRP1); PF08512: Histone chaperone Rttp106-like; PF00505: HMG (high mobility group) box | GO:0003677 MF: DNA binding;GO:0005634 CC: nucleus |
|  |  |  |  |  | TraesCS3B01G132100 | bZIP transcription factor, putative (DUF1664) | PF07889: Protein of unknown function (DUF1664) | NA |
|  |  |  |  |  | TraesCS3B01G132200 | FAD-binding Berberine family protein | PF01565: FAD binding domain; PF08031: Berberine and berberine like | GO:0003824 MF: catalytic activity;GO:0016491 MF: oxidoreductase activity; |
|  |  |  |  |  | TraesCS3B01G132300 | Glutamine--tRNA ligase | PF04558: Glutaminyl-tRNA synthetase, non-specific RNA binding region part 1; PF04557: Glutaminyl-tRNA synthetase, non-specific RNA binding region part 2; PF00749: tRNA synthetases class I (E and Q), catalytic domain; PF03950: tRNA synthetases class I (E and Q), anti-codon binding domain | GO:0000166 MF: nucleotide binding;GO:0004812 MF: aminoacyl-tRNA ligase activity; |
|  |  |  |  |  | TraesCS3B01G132400 | 3'-N-debenzoyl-2'-deoxytaxol N-benzoyltransferase | PF02458: Transferase family | GO:0016747 MF: transferase activity, transferring acyl groups other than amino-acyl groups |
|  |  |  |  |  | TraesCS3B01G132500 | Extracellular ribonuclease | PF04231: Endonuclease I | GO:0004518 MF: nuclease activity |
|  |  |  |  |  | TraesCS3B01G132600 | regulatory particle non-ATPase subunit 5B | NA | NA |
|  |  |  |  |  | TraesCS3B01G132700 | Threonine--tRNA ligase | NA | NA |
|  |  |  |  |  | TraesCS3B01G132800 | Cysteine/Histidine-rich C1 domain family protein | PF07762: Protein of unknown function (DUF1618) | NA |
|  |  |  |  |  | TraesCS3B01G132900 | cDNA clone:001-038-D11, full insert sequence | NA | NA |
|  |  |  |  |  | TraesCS3B01G133000 | F-box protein | PF00646: F-box domain; PF03478: Protein of unknown function (DUF295) | GO:0005515 MF: protein binding |
|  |  |  |  |  | TraesCS3B01G133100 | Histone-lysine N-methyltransferase | PF00628: PHD-finger; PF13832: PHD-zinc-finger like domain | GO:0005515 MF: protein binding;GO:0008270 MF: zinc ion binding |
|  |  |  |  |  | TraesCS3B01G133200 | 60S ribosomal protein L18a-like protein | NA | NA |
|  |  |  |  |  | TraesCS3B01G133300 | Anthocyanidin 5,3-O-glucosyltransferase | NA | NA |
| Relative root shoot ratio | AX-158548980 |  | 3D | 533939883 | TraesCS3D01G420700 | RING/U-box superfamily protein | PF13923: Zinc finger, C3HC4 type (RING finger) | GO:0005515 MF: protein binding;GO:0008270 MF: zinc ion binding |
|  |  |  |  |  | TraesCS3D01G420800 | RING/U-box superfamily protein | PF13923: Zinc finger, C3HC4 type (RING finger) | GO:0005515 MF: protein binding;GO:0008270 MF: zinc ion binding |
|  |  |  |  |  | TraesCS3D01G420900 | RING/U-box superfamily protein | PF13923: Zinc finger, C3HC4 type (RING finger) | GO:0005515 MF: protein binding;GO:0008270 MF: zinc ion binding |
|  |  |  |  |  | TraesCS3D01G421000 | RING/U-box superfamily protein | PF13923: Zinc finger, C3HC4 type (RING finger) | GO:0005515 MF: protein binding;GO:0008270 MF: zinc ion binding |
|  |  |  |  |  | TraesCS3D01G421100 | RING/U-box superfamily protein | PF13923: Zinc finger, C3HC4 type (RING finger) | GO:0005515 MF: protein binding;GO:0008270 MF: zinc ion binding |
|  |  |  |  |  | TraesCS3D01G421200 | RING/U-box superfamily protein | PF13923: Zinc finger, C3HC4 type (RING finger) | GO:0005515 MF: protein binding;GO:0008270 MF: zinc ion binding |
|  |  |  |  |  | TraesCS3D01G421300 | lysine ketoglutarate reductase trans-splicing-like protein, putative (DUF707) | PF05212: Protein of unknown function (DUF707) | NA |
|  |  |  |  |  | TraesCS3D01G421400 | ArfGap/RecO-like zinc finger domain-containing protein | NA | NA |
|  |  |  |  |  | TraesCS3D01G421500 | Alpha/beta hydrolase | PF00561: alpha/beta hydrolase fold | NA |
|  |  |  |  |  | TraesCS3D01G421600 | Auxin efflux carrier component | PF03547: Membrane transport protein | GO:0016021 CC: integral component of membrane;GO:0055085 BP: transmembrane transport |
|  |  |  |  |  | TraesCS3D01G421700 | Acyl-[acyl-carrier-protein] desaturase | PF03405: Fatty acid desaturase | GO:0006631 BP: fatty acid metabolic process;GO:0045300 MF: acyl-[acyl-carrier-protein] desaturase activity; |
|  |  |  |  |  | TraesCS3D01G421800 | F-box domain containing protein | NA | GO:0005515 MF: protein binding |
|  |  |  |  |  | TraesCS3D01G421900 | Disease resistance protein RPM1 | PF00931: NB-ARC domain | GO:0043531 MF: ADP binding |
|  |  |  |  |  | TraesCS3D01G422000 | F-box family protein | PF00646: F-box domain; PF08387: FBD | GO:0005515 MF: protein binding |
|  |  |  |  |  | TraesCS3D01G422100 | F-box family protein | PF00646: F-box domain; PF08387: FBD | GO:0005515 MF: protein binding |
|  |  |  |  |  | TraesCS3D01G422200 | Extracellular matrix-binding protein ebh | NA | NA |
|  |  |  |  |  | TraesCS3D01G422300 | Histone deacetylase | PF00850: Histone deacetylase domain | NA |
|  |  |  |  |  | TraesCS3D01G422400 | F-box family protein | NA | NA |
| Relative root fresh weight | AX-158603951 |  | 4A | 534834602 | TraesCS4A01G225700 | Gibberellin receptor GID1a | PF07859: alpha/beta hydrolase fold | GO:0008152 BP: metabolic process;GO:0016787 MF: hydrolase activity |
|  |  |  |  |  | TraesCS4A01G225800 | CCR4-NOT transcription complex subunit 11 | PF10155: Uncharacterized conserved protein (DUF2363) | GO:0030014 CC: CCR4-NOT complex |
|  |  |  |  |  | TraesCS4A01G225900 | BPS1-like protein (DUF793) | PF03087: Arabidopsis protein of unknown function | NA |
|  |  |  |  |  | TraesCS4A01G226000 | ATP-dependent Clp protease ATP-binding subunit | PF00004: ATPase family associated with various cellular activities (AAA); PF07724: AAA domain (Cdc48 subfamily); PF10431: C-terminal, D2-small domain, of ClpB protein | GO:0005524 MF: ATP binding |
|  |  |  |  |  | TraesCS4A01G226100 | Serine carboxypeptidase family protein, expressed | PF00450: Serine carboxypeptidase | GO:0004185 MF: serine-type carboxypeptidase activity;GO:0006508 BP: proteolysis |
|  |  |  |  |  | TraesCS4A01G226200 | DUF538 family protein, putative (Protein of unknown function, DUF538) | PF04398: Protein of unknown function, DUF538 | NA |
|  |  |  |  |  | TraesCS4A01G226300 | Protein phosphatase 2c, putative | PF00481: Protein phosphatase 2C | GO:0003824 MF: catalytic activity;GO:0043169 MF: cation binding |
|  |  |  |  |  | TraesCS4A01G226400 | Non-lysosomal glucosylceramidase | PF12215: beta-glucosidase 2, glycosyl-hydrolase family 116 N-term; PF04685: Glycosyl-hydrolase family 116, catalytic region | GO:0003824 MF: catalytic activity;GO:0004348 MF: glucosylceramidase activity; |
|  |  |  |  |  | TraesCS4A01G226500 | DNA polymerase III subunit | PF13177: DNA polymerase III, delta subunit; PF12169: DNA polymerase III subunits gamma and tau domain III | GO:0003677 MF: DNA binding;GO:0003887 MF: DNA-directed DNA polymerase activity; |
|  |  |  |  |  | TraesCS4A01G226600 | lysine ketoglutarate reductase trans-splicing-like protein, putative (DUF707) | PF05212: Protein of unknown function (DUF707) | NA |
|  |  |  |  |  | TraesCS4A01G226700 | Heat-shock protein, putative | PF00011: Hsp20/alpha crystallin family | NA |
|  |  |  |  |  | TraesCS4A01G226800 | Zinc finger-homeodomain protein 1 | PF04770: ZF-HD protein dimerisation region | GO:0003677 MF: DNA binding |
|  |  |  |  |  | TraesCS4A01G226900 | Chlorophyll a-b binding protein, chloroplastic | PF00504: Chlorophyll A-B binding protein | NA |
|  |  |  |  |  | TraesCS4A01G227000 | Glycine-rich protein | NA | NA |
|  |  |  |  |  | TraesCS4A01G227100 | Glucuronoxylan 4-O-methyltransferase | PF04669: Polysaccharide biosynthesis | GO:0045492 BP: xylan biosynthetic process |
|  |  |  |  |  | TraesCS4A01G227200 | Protein kinase-like protein | PF00069: Protein kinase domain | GO:0004672 MF: protein kinase activity;GO:0005524 MF: ATP binding; |
|  |  |  |  |  | TraesCS4A01G227300 | Chlorophyll a/b binding protein domain-containing protein | PF13806: Rieske-like [2Fe-2S] domain | GO:0008942 MF: nitrite reductase [NAD(P)H] activity;GO:0016491 MF: oxidoreductase activity; |
|  |  |  |  |  | TraesCS4A01G227400 | ethylene-responsive transcription factor | PF00847: AP2 domain | GO:0003677 MF: DNA binding;GO:0003700 MF: transcription factor activity, sequence-specific DNA binding; |
| Relative_RL | AX-158582574 | Com_Hap1 | 4B | 456188724 | TraesCS4B01G200500 | Serine/threonine-protein kinase | NA | NA |
| Relative root ratio | AX-158582574 | Com_Hap1 | 4B | 456188724 | TraesCS4B01G200600 | ABC transporter G family member | PF00005: ABC transporter; PF01061: ABC-2 type transporter | GO:0005524 MF: ATP binding;GO:0016020 CC: membrane; |
|  |  |  |  |  | TraesCS4B01G200700 | tRNA-2-methylthio-N(6)-dimethylallyladenosine synthase | PF13456: Reverse transcriptase-like | GO:0003676 MF: nucleic acid binding |
|  |  |  |  |  | TraesCS4B01G200800 | FKBP-type peptidyl-prolyl cis-trans isomerase | NA | NA |
|  |  |  |  |  | TraesCS4B01G200900 | Metal-dependent protein hydrolase | NA | NA |
|  |  |  |  |  | TraesCS4B01G201100 | Integrator complex subunit 4 | NA | GO:0005488 MF: binding |
|  |  |  |  |  | TraesCS4B01G201200 | Phosphoglycerate kinase | NA | NA |
|  |  |  |  |  | TraesCS4B01G201300 | F-box domain containing protein-like | NA | NA |
|  |  |  |  |  | TraesCS4B01G201500 | Calcium-transporting ATPase | PF00690: Cation transporter/ATPase, N-terminus; PF00122: E1-E2 ATPase; PF00702: haloacid dehalogenase-like hydrolase; PF00689: Cation transporting ATPase, C-terminus | GO:0000166 MF: nucleotide binding;GO:0016021 CC: integral component of membrane; |
|  |  |  |  |  | TraesCS4B01G201600 | Receptor-like kinase, putative | PF12819: Carbohydrate-binding protein of the ER; PF07714: Protein tyrosine kinase | GO:0004672 MF: protein kinase activity;GO:0005524 MF: ATP binding; |
|  |  |  |  |  | TraesCS4B01G201700 | F-box domain containing protein, expressed | NA | NA |
|  |  |  |  |  | TraesCS4B01G201800 | Cytosine-specific methyltransferase | PF01426: BAH domain; PF00385: Chromo (CHRromatin Organisation MOdifier) domain; PF00145: C-5 cytosine-specific DNA methylase | GO:0003682 MF: chromatin binding;GO:0008168 MF: methyltransferase activity |
|  |  |  |  |  | TraesCS4B01G201900 | Werner Syndrome-like exonuclease | NA | GO:0003676 MF: nucleic acid binding |
|  |  |  |  |  | TraesCS4B01G202000 | Cytochrome P450 | PF00067: Cytochrome P450 | GO:0005506 MF: iron ion binding;GO:0016705 MF: oxidoreductase activity, acting on paired donors, with incorporation or reduction of molecular oxygen; |
|  |  |  |  |  | TraesCS4B01G202100 | centrosomal protein of 135 kDa-like protein | NA | NA |
|  |  |  |  |  | TraesCS4B01G202200 | Membrane-associated kinase regulator-like protein, putative | NA | NA |
|  |  |  |  |  | TraesCS4B01G202300 | Ankyrin repeat-containing protein | PF12796: Ankyrin repeats (3 copies); PF00023: Ankyrin repeat; PF13962: Domain of unknown function | GO:0005515 MF: protein binding |
|  |  |  |  |  | TraesCS4B01G202400 | Ankyrin repeat family protein, putative, expressed | PF12796: Ankyrin repeats (3 copies); PF13962: Domain of unknown function | GO:0005515 MF: protein binding |
|  |  |  |  |  | TraesCS4B01G202500 | UDP-glucuronate decarboxylase protein 1 | PF16363: GDP-mannose 4,6 dehydratase | NA |
|  |  |  |  |  | TraesCS4B01G202600 | Dirigent protein | PF03018: Dirigent-like protein | NA |
|  |  |  |  |  | TraesCS4B01G202700 | methyl-coenzyme M reductase II subunit gamma, putative (DUF3741) | PF14383: DUF761-associated sequence motif; PF12552: Protein of unknown function (DUF3741); PF14309: Domain of unknown function (DUF4378) | NA |
|  |  |  |  |  | TraesCS4B01G202800 | peptidase M50B-like protein | PF13398: Peptidase M50B-like | NA |
|  |  |  |  |  | TraesCS4B01G202900 | Plant protein 1589 of Uncharacterized protein function | PF09713: Plant protein 1589 of unknown function (A_thal_3526) | NA |
|  |  |  |  |  | TraesCS4B01G203000 | ABC transporter ATP-binding protein | PF00664: ABC transporter transmembrane region; PF00005: ABC transporter | GO:0005524 MF: ATP binding;GO:0006810 BP: transport; |
|  |  |  |  |  | TraesCS4B01G203100 | PsbP family protein, expressed | PF01789: PsbP | GO:0005509 MF: calcium ion binding;GO:0009523 CC: photosystem II; |
|  |  |  |  |  | TraesCS4B01G203200 | Nitric oxide synthase-interacting protein homolog | PF15906: Zinc-finger of nitric oxide synthase-interacting protein; PF04641: Rtf2 RING-finger | GO:0005515 MF: protein binding;GO:0008270 MF: zinc ion binding; |
|  |  |  |  |  | TraesCS4B01G203300 | Kinesin-like protein | PF00225: Kinesin motor domain | GO:0003777 MF: microtubule motor activity;GO:0005524 MF: ATP binding; |
|  |  |  |  |  | TraesCS4B01G203400 | Zinc finger family protein | PF13912: C2H2-type zinc finger | GO:0003676 MF: nucleic acid binding |
|  |  |  |  |  | TraesCS4B01G203500 | 3'-5' exonuclease domain-containing protein | NA | GO:0003676 MF: nucleic acid binding |
|  |  |  |  |  | TraesCS4B01G203600 | Peptide chain release factor 3 | NA | NA |
|  |  |  |  |  | TraesCS4B01G203700 | basic helix-loop-helix (bHLH) DNA-binding superfamily protein | NA | GO:0046983 MF: protein dimerization activity |
|  |  |  |  |  | TraesCS4B01G203800 | ATP-binding cassette sub-family D member 2 | NA | NA |
|  |  |  |  |  | TraesCS4B01G203900 | Histone-lysine N-methyltransferase | PF02182: SAD/SRA domain; PF05033: Pre-SET motif; PF00856: SET domain | GO:0005515 MF: protein binding;GO:0005634 CC: nucleus;GO:0008270 MF: zinc ion binding; |
|  |  |  |  |  | TraesCS4B01G204000 | Arginine biosynthesis bifunctional protein ArgJ | PF01960: ArgJ family | GO:0004358 MF: glutamate N-acetyltransferase activity;GO:0006526 BP: arginine biosynthetic process |
|  |  |  |  |  | TraesCS4B01G204100 | Histone H2A | PF00125: Core histone H2A/H2B/H3/H4; PF16211: C-terminus of histone H2A | GO:0000786 CC: nucleosome;GO:0003677 MF: DNA binding; |
|  |  |  |  |  | TraesCS4B01G204200 | Histone H2A | PF00125: Core histone H2A/H2B/H3/H4; PF16211: C-terminus of histone H2A | GO:0000786 CC: nucleosome;GO:0003677 MF: DNA binding; |
|  |  |  |  |  | TraesCS4B01G204300 | RNA-binding family protein | PF00076: RNA recognition motif. (a.k.a. RRM, RBD, or RNP domain) | GO:0003676 MF: nucleic acid binding |
|  |  |  |  |  | TraesCS4B01G204400 | Histone H2B | PF00125: Core histone H2A/H2B/H3/H4 | GO:0000786 CC: nucleosome;GO:0003677 MF: DNA binding; |
|  |  |  |  |  | TraesCS4B01G204500 | ATP synthase subunit b | PF00430: ATP synthase B/B' CF(0) | GO:0015078 MF: hydrogen ion transmembrane transporter activity;GO:0015986 BP: ATP synthesis coupled proton transport; |
|  |  |  |  |  | TraesCS4B01G204600 | RNA binding protein | PF00076: RNA recognition motif. (a.k.a. RRM, RBD, or RNP domain) | GO:0003676 MF: nucleic acid binding |
|  |  |  |  |  | TraesCS4B01G204700 | Acyl-CoA N-acyltransferase with RING/FYVE/PHD-type zinc finger protein | PF16135: TPL-binding domain in jasmonate signalling | GO:0008080 MF: N-acetyltransferase activity |
|  |  |  |  |  | TraesCS4B01G204800 | Polyadenylate-binding protein | PF00076: RNA recognition motif. (a.k.a. RRM, RBD, or RNP domain); PF00658: Poly-adenylate binding protein, unique domain | GO:0003676 MF: nucleic acid binding;GO:0003723 MF: RNA binding |
|  |  |  |  |  | TraesCS4B01G204900 | ADP-ribosylation factor GTPase-activating protein | PF01412: Putative GTPase activating protein for Arf | GO:0005096 MF: GTPase activator activity |
|  |  |  |  |  | TraesCS4B01G205000 | RNA-binding family protein | PF00076: RNA recognition motif. (a.k.a. RRM, RBD, or RNP domain); PF13865: C-terminal duplication domain of Friend of PRMT1 | GO:0003676 MF: nucleic acid binding |
|  |  |  |  |  | TraesCS4B01G205100 | Udp-glucose 4,6-dehydratase | PF16363: GDP-mannose 4,6 dehydratase; PF04321: RmlD substrate binding domain | NA |
|  |  |  |  |  | TraesCS4B01G205200 | UDP-glucuronate decarboxylase protein 6 | PF16363: GDP-mannose 4,6 dehydratase | NA |
|  |  |  |  |  | TraesCS4B01G205300 | Cysteine-rich receptor-kinase-like protein | PF01657: Salt stress response/antifungal | NA |
|  |  |  |  |  | TraesCS4B01G205400 | NAD(P)H dehydrogenase (Quinone) | PF03358: NADPH-dependent FMN reductase | GO:0003955 MF: NAD(P)H dehydrogenase (quinone) activity;GO:0010181 MF: FMN binding; |
|  |  |  |  |  | TraesCS4B01G205500 | Seed maturation protein PM41 | NA | NA |
|  |  |  |  |  | TraesCS4B01G205600 | Dessication-induced 1VOC superfamily protein | PF00903: Glyoxalase/Bleomycin resistance protein/Dioxygenase superfamily | NA |
|  |  |  |  |  | TraesCS4B01G205700 | 70 kDa heat shock protein | PF00012: Hsp70 protein | NA |
|  |  |  |  |  | TraesCS4B01G205800 | HSP20-like chaperones superfamily protein | NA | NA |
|  |  |  |  |  | TraesCS4B01G205900 | Guanosine nucleotide diphosphate dissociation inhibitor | PF00996: GDP dissociation inhibitor | GO:0005092 MF: GDP-dissociation inhibitor activity;GO:0005093 MF: Rab GDP-dissociation inhibitor activity; |
|  |  |  |  |  | TraesCS4B01G206000 | Serine/threonine-protein phosphatase 7 long form-like protein | PF10536: Plant mobile domain | NA |
|  |  |  |  |  | TraesCS4B01G206100 | NA | NA | NA |
|  |  |  |  |  | TraesCS4B01G206200 | Xylosyltransferase 1 | PF02485: Core-2/I-Branching enzyme | GO:0008375 MF: acetylglucosaminyltransferase activity;GO:0016020 CC: membrane |
|  |  |  |  |  | TraesCS4B01G206300 | 70 kDa heat shock protein | PF00012: Hsp70 protein | NA |
|  |  |  |  |  | TraesCS4B01G206400 | 70 kDa heat shock protein | PF00012: Hsp70 protein | NA |
|  |  |  |  |  | TraesCS4B01G206500 | NA | NA | NA |
|  |  |  |  |  | TraesCS4B01G206600 | FMN-binding split barrel | PF13883: Pyridoxamine 5'-phosphate oxidase | GO:0010181 MF: FMN binding;GO:0016491 MF: oxidoreductase activity; |
|  |  |  |  |  | TraesCS4B01G206700 | 70 kDa heat shock protein | PF00012: Hsp70 protein | NA |
|  |  |  |  |  | TraesCS4B01G206800 | Dof zinc finger protein | PF02701: Dof domain, zinc finger | GO:0003677 MF: DNA binding;GO:0006355 BP: regulation of transcription, DNA-templated |
|  |  |  |  |  | TraesCS4B01G206900 | U-box domain-containing protein | PF04564: U-box domain; PF00514: Armadillo/beta-catenin-like repeat; PF05536: Neurochondrin | GO:0004842 MF: ubiquitin-protein transferase activity;GO:0005488 MF: binding; |
|  |  |  |  |  | TraesCS4B01G207000 | Cytochrome P450 | PF00067: Cytochrome P450 | GO:0005506 MF: iron ion binding;GO:0016705 MF: oxidoreductase activity, acting on paired donors, with incorporation or reduction of molecular oxygen; |
|  |  |  |  |  | TraesCS4B01G207100 | Tryptophan synthase alpha chain | PF00290: Tryptophan synthase alpha chain | GO:0003824 MF: catalytic activity;GO:0004834 MF: tryptophan synthase activity; |
|  |  |  |  |  | TraesCS4B01G207200 | Clathrin assembly protein, putative, expressed | PF07651: ANTH domain | GO:0005543 MF: phospholipid binding;GO:0005545 MF: 1-phosphatidylinositol binding; |
|  |  |  |  |  | TraesCS4B01G207300 | S-acyltransferase | PF01529: DHHC palmitoyltransferase | NA |
|  |  |  |  |  | TraesCS4B01G207400 | Ankyrin repeat family protein, putative, expressed | PF12796: Ankyrin repeats (3 copies); PF13857: Ankyrin repeats (many copies) | GO:0005515 MF: protein binding;GO:0008270 MF: zinc ion binding |
|  |  |  |  |  | TraesCS4B01G207500 | Protein kinase | PF00069: Protein kinase domain | GO:0004672 MF: protein kinase activity;GO:0005524 MF: ATP binding; |
|  |  |  |  |  | TraesCS4B01G207600 | ubiquitin carboxyl-terminal hydrolase | NA | NA |
|  |  |  |  |  | TraesCS4B01G207700 | Telomere repeat-binding protein 5 | NA | GO:0003677 MF: DNA binding |
|  |  |  |  |  | TraesCS4B01G207800 | Oxysterol-binding protein | PF01237: Oxysterol-binding protein | NA |
|  |  |  |  |  | TraesCS4B01G207900 | Haloacid dehalogenase-like hydrolase family protein, putative, expressed | NA | GO:0008152 BP: metabolic process;GO:0016787 MF: hydrolase activity |
|  |  |  |  |  | TraesCS4B01G208000 | Laccase | PF07732: Multicopper oxidase; PF00394: Multicopper oxidase; PF07731: Multicopper oxidase | GO:0005507 MF: copper ion binding;GO:0016491 MF: oxidoreductase activity;GO:0046274 BP: lignin catabolic process;GO:0048046 CC: apoplast;GO:0052716 MF: hydroquinone:oxygen oxidoreductase activity;GO:0055114 BP: oxidation-reduction process |
|  |  |  |  |  | TraesCS4B01G208100 | Rotundifolia-like protein | PF08137: DVL family | NA |
|  |  |  |  |  | TraesCS4B01G208200 | tRNA (Guanine(26)-N(2))-dimethyltransferase | PF02005: N2,N2-dimethylguanosine tRNA methyltransferase | GO:0003723 MF: RNA binding;GO:0004809 MF: tRNA (guanine-N2-)-methyltransferase activity; |
|  |  |  |  |  | TraesCS4B01G208300 | NA | NA | NA |
|  |  |  |  |  | TraesCS4B01G208400 | NA | NA | NA |
|  |  |  |  |  | TraesCS4B01G208500 | far-red elongated hypocotyl 1 | NA | NA |
|  |  |  |  |  | TraesCS4B01G208600 | NA | NA | NA |
|  |  |  |  |  | TraesCS4B01G208600 | RING/U-box superfamily protein | PF13639: Ring finger domain | GO:0005515 MF: protein binding;GO:0008270 MF: zinc ion binding |
|  |  |  |  |  | TraesCS4B01G208700 | Eukaryotic aspartyl protease family protein, expressed | PF14543: Xylanase inhibitor N-terminal; PF14541: Xylanase inhibitor C-terminal | GO:0004190 MF: aspartic-type endopeptidase activity;GO:0006508 BP: proteolysis |
|  |  |  |  |  | TraesCS4B01G208800 | Pentatricopeptide repeat-containing protein | PF01535: PPR repeat; PF13041: PPR repeat family | GO:0005515 MF: protein binding |
|  |  |  |  |  | TraesCS4B01G208900 | Hsp70 nucleotide exchange factor fes1 | NA | GO:0005488 MF: binding |
|  |  |  |  |  | TraesCS4B01G209000 | Helicase/SANT-associated, DNA binding protein | NA | NA |
|  |  |  |  |  | TraesCS4B01G209100 | RING/U-box superfamily protein | PF14369: zinc-ribbon; PF13639: Ring finger domain; PF06547: Protein of unknown function (DUF1117) | GO:0005515 MF: protein binding;GO:0008270 MF: zinc ion binding |
|  |  |  |  |  | TraesCS4B01G209200 | Protein TOC75-3, chloroplastic | PF01103: Surface antigen | GO:0019867 CC: outer membrane |
|  |  |  |  |  | TraesCS4B01G209300 | RNA polymerase sigma factor | PF04542: Sigma-70 region 2; PF04539: Sigma-70 region 3; PF04545: Sigma-70, region 4 | GO:0003677 MF: DNA binding;GO:0003700 MF: transcription factor activity, sequence-specific DNA binding; |
|  |  |  |  |  | TraesCS4B01G209400 | Cleavage and polyadenylation specificity factor subunit 2 | NA | NA |
|  |  |  |  |  | TraesCS4B01G209500 | Hydroxyproline-rich glycoprotein-like | NA | NA |
|  |  |  |  |  | TraesCS4B01G209600 | Avr9/Cf-9 rapidly elicited protein | PF11961: Domain of unknown function (DUF3475); PF05003: Protein of unknown function (DUF668) | NA |
|  |  |  |  |  | TraesCS4B01G209700 | Serine/arginine repetitive matrix protein 1 | PF01480: PWI domain | GO:0006397 BP: mRNA processing |
|  |  |  |  |  | TraesCS4B01G209800 | AT hook motif DNA-binding family protein | PF03479: Domain of unknown function (DUF296) | NA |
|  |  |  |  |  | TraesCS4B01G209900 | Tropinone reductase-like protein | PF13561: Enoyl-(Acyl carrier protein) reductase | GO:0016491 MF: oxidoreductase activity |
|  |  |  |  |  | TraesCS4B01G210000 | Tropinone reductase, putative, expressed | PF13561: Enoyl-(Acyl carrier protein) reductase | GO:0016491 MF: oxidoreductase activity |
|  |  |  |  |  | TraesCS4B01G210100 | Protein phosphatase 2C | PF00481: Protein phosphatase 2C | GO:0003824 MF: catalytic activity;GO:0043169 MF: cation binding |
|  |  |  |  |  | TraesCS4B01G210200 | RNA-binding protein | PF00076: RNA recognition motif. (a.k.a. RRM, RBD, or RNP domain) | GO:0003676 MF: nucleic acid binding |
|  |  |  |  |  | TraesCS4B01G210300 | Mannose-1-phosphate guanyltransferase, putative | PF00483: Nucleotidyl transferase; PF00132: Bacterial transferase hexapeptide (six repeats) | GO:0009058 BP: biosynthetic process;GO:0016779 MF: nucleotidyltransferase activity |
|  |  |  |  |  | TraesCS4B01G210400 | Digalactosyldiacylglycerol synthase (Chloroplastic) | PF00534: Glycosyl transferases group 1 | NA |
|  |  |  |  |  | TraesCS4B01G210600 | Kinase family protein | PF00069: Protein kinase domain | GO:0004672 MF: protein kinase activity;GO:0005524 MF: ATP binding; |
|  |  |  |  |  | TraesCS4B01G210700 | Myosin heavy chain-related protein | NA | NA |
|  |  |  |  |  | TraesCS4B01G210800 | Serine/threonine-protein phosphatase | PF16891: Serine-threonine protein phosphatase N-terminal domain; PF00149: Calcineurin-like phosphoesterase | GO:0016787 MF: hydrolase activity |
|  |  |  |  |  | TraesCS4B01G210900 | LIM domain-containing protein 1 | PF00412: LIM domain; PF12315: Protein DA1 | GO:0008270 MF: zinc ion binding |
|  |  |  |  |  | TraesCS4B01G211000 | Mitochondrial carrier protein, expressed | PF00153: Mitochondrial carrier protein | GO:0055085 BP: transmembrane transport |
|  |  |  |  |  | TraesCS4B01G211100 | Myb/SANT-like DNA-binding domain protein | PF12776: Myb/SANT-like DNA-binding domain | NA |
|  |  |  |  |  | TraesCS4B01G211200 | DUF3527 domain protein | PF12043: Domain of unknown function (DUF3527) | NA |
|  |  |  |  |  | TraesCS4B01G211300 | lipase, putative (DUF620) | PF04788: Protein of unknown function (DUF620) | NA |
|  |  |  |  |  | TraesCS4B01G211400 | Fructose-1,6-bisphosphatase class 1 | PF00316: Fructose-1-6-bisphosphatase, N-terminal domain | GO:0005975 BP: carbohydrate metabolic process;GO:0016791 MF: phosphatase activity; |
|  |  |  |  |  | TraesCS4B01G211500 | NA | NA | NA |
|  |  |  |  |  | TraesCS4B01G211500 | Transducin/WD40 repeat protein | PF00400: WD domain, G-beta repeat | GO:0005515 MF: protein binding |
|  |  |  |  |  | TraesCS4B01G211600 | Heat-shock protein | PF00011: Hsp20/alpha crystallin family | NA |
|  |  |  |  |  | TraesCS4B01G211700 | Heat-shock protein | PF00011: Hsp20/alpha crystallin family | NA |
|  |  |  |  |  | TraesCS4B01G211800 | Receptor-like protein kinase | PF08263: Leucine rich repeat N-terminal domain; PF13855: Leucine rich repeat; PF00069: Protein kinase domain | GO:0004672 MF: protein kinase activity;GO:0005515 MF: protein binding; |
|  |  |  |  |  | TraesCS4B01G211900 | SH3 domain-containing protein 1 | PF14604: Variant SH3 domain | GO:0005515 MF: protein binding |
|  |  |  |  |  | TraesCS4B01G212000 | PHD finger-containing protein | NA | GO:0005515 MF: protein binding;GO:0008270 MF: zinc ion binding |
|  |  |  |  |  | TraesCS4B01G212100 | NA | NA | NA |
|  |  |  |  |  | TraesCS4B01G212200 | Heat-shock protein, putative | PF00011: Hsp20/alpha crystallin family | NA |
|  |  |  |  |  | TraesCS4B01G212300 | Heat-shock protein, putative | PF00011: Hsp20/alpha crystallin family | NA |
|  |  |  |  |  | TraesCS4B01G212400 | neuronal PAS domain protein | NA | NA |
|  |  |  |  |  | TraesCS4B01G212500 | Enolase-like protein | PF03952: Enolase, N-terminal domain; PF00113: Enolase, C-terminal TIM barrel domain | GO:0000015 CC: phosphopyruvate hydratase complex;GO:0000287 MF: magnesium ion binding; |
|  |  |  |  |  | TraesCS4B01G212600 | LIM domain-containing protein | PF00412: LIM domain | GO:0008270 MF: zinc ion binding |
|  |  |  |  |  | TraesCS4B01G212700 | OTU domain-containing protein | PF02338: OTU-like cysteine protease | NA |
|  |  |  |  |  | TraesCS4B01G212800 | Pentatricopeptide repeat (PPR) superfamily protein | NA | NA |
|  |  |  |  |  | TraesCS4B01G212900 | Ubiquitin-like-specific protease ESD4 | PF02902: Ulp1 protease family, C-terminal catalytic domain | GO:0006508 BP: proteolysis;GO:0008234 MF: cysteine-type peptidase activity |
|  |  |  |  |  | TraesCS4B01G213000 | Maternal effect embryo arrest 59 | NA | NA |
|  |  |  |  |  | TraesCS4B01G213100 | Transmembrane protein | PF06127: Protein of unknown function (DUF962) | NA |
|  |  |  |  |  | TraesCS4B01G213200 | SH3 domain containing protein, expressed | PF00018: SH3 domain | GO:0005488 MF: binding;GO:0005515 MF: protein binding |
|  |  |  |  |  | TraesCS4B01G213300 | NA | NA | NA |
|  |  |  |  |  | TraesCS4B01G213400 | RNA-binding family protein | PF00076: RNA recognition motif. (a.k.a. RRM, RBD, or RNP domain) | GO:0003676 MF: nucleic acid binding |
|  |  |  |  |  | TraesCS4B01G213500 | Coronatine-insensitive protein 1 | NA | NA |
|  |  |  |  |  | TraesCS4B01G213600 | 50S ribosomal protein L4 | PF00573: Ribosomal protein L4/L1 family | GO:0003735 MF: structural constituent of ribosome;GO:0005840 CC: ribosome; |
|  |  |  |  |  | TraesCS4B01G213700 | Mitochondrial carrier family | PF00153: Mitochondrial carrier protein | NA |
|  |  |  |  |  | TraesCS4B01G213800 | hydroxysteroid dehydrogenase 3 | PF00106: short chain dehydrogenase | NA |
|  |  |  |  |  | TraesCS4B01G213900 | Josephin, putative, expressed | PF02099: Josephin | GO:0004843 MF: thiol-dependent ubiquitin-specific protease activity;GO:0016579 BP: protein deubiquitination |
|  |  |  |  |  | TraesCS4B01G214000 | Glycogen synthase | PF13439: Glycosyltransferase Family 4; PF00534: Glycosyl transferases group 1 | NA |
|  |  |  |  |  | TraesCS4B01G214100 | Holliday junction ATP-dependent DNA helicase RuvB | PF00004: ATPase family associated with various cellular activities (AAA) | GO:0005524 MF: ATP binding |
|  |  |  |  |  | TraesCS4B01G214200 | BTB/POZ domain-containing protein | NA | GO:0005515 MF: protein binding |
|  |  |  |  |  | TraesCS4B01G214300 | NA | NA | NA |
|  |  |  |  |  | TraesCS4B01G214400 | C2H2-like zinc finger protein | NA | GO:0003676 MF: nucleic acid binding |
|  |  |  |  |  | TraesCS4B01G214500 | Anthranilate synthase | PF04715: Anthranilate synthase component I, N terminal region; PF00425: chorismate binding enzyme | GO:0000162 BP: tryptophan biosynthetic process;GO:0004049 MF: anthranilate synthase activity; |
|  |  |  |  |  | TraesCS4B01G214600 | Receptor-like kinase | PF00069: Protein kinase domain | GO:0004672 MF: protein kinase activity;GO:0005524 MF: ATP binding; |
|  |  |  |  |  | TraesCS4B01G214700 | Tetratricopeptide repeat (TPR)-like superfamily protein, putative isoform 1 | NA | NA |
|  |  |  |  |  | TraesCS4B01G214800 | LAG1 longevity assurance-like protein | PF03798: TLC domain | GO:0005783 CC: endoplasmic reticulum;GO:0016021 CC: integral component of membrane |
|  |  |  |  |  | TraesCS4B01G214900 | Endonuclease/exonuclease/phosphatase family protein | NA | NA |
|  |  |  |  |  | TraesCS4B01G215000 | Calcium-binding EF hand family protein, putative, expressed | PF13432: Tetratricopeptide repeat; PF14559: Tetratricopeptide repeat | GO:0005509 MF: calcium ion binding;GO:0005515 MF: protein binding |
|  |  |  |  |  | TraesCS4B01G215100 | S-ribonuclease binding protein | PF13920: Zinc finger, C3HC4 type (RING finger) | GO:0004842 MF: ubiquitin-protein transferase activity;GO:0005515 MF: protein binding; |
|  |  |  |  |  | TraesCS4B01G215200 | Selenoprotein K | PF10961: Selenoprotein SelK_SelG | NA |
|  |  |  |  |  | TraesCS4B01G215300 | Strictosidine synthase family protein | PF03088: Strictosidine synthase | GO:0009058 BP: biosynthetic process;GO:0016844 MF: strictosidine synthase activity |
|  |  |  |  |  | TraesCS4B01G215400 | F-box domain containing protein, expressed | NA | NA |
|  |  |  |  |  | TraesCS4B01G215500 | Splicing factor 3A subunit 2 | PF12874: Zinc-finger of C2H2 type; PF16835: Pre-mRNA-splicing factor SF3a complex subunit 2 (Prp11) | GO:0003676 MF: nucleic acid binding;GO:0005634 CC: nucleus; |
|  |  |  |  |  | TraesCS4B01G215600 | Mitochondrial carrier family | PF00153: Mitochondrial carrier protein | NA |
|  |  |  |  |  | TraesCS4B01G215700 | Nodulation-signaling pathway 2 protein | PF03514: GRAS domain family | NA |
|  |  |  |  |  | TraesCS4B01G215800 | ethylene-responsive transcription factor | PF00847: AP2 domain | GO:0003677 MF: DNA binding;GO:0003700 MF: transcription factor activity, sequence-specific DNA binding; |
|  |  |  |  |  | TraesCS4B01G215900 | Vacuolar protein sorting-associated protein 9A | PF02204: Vacuolar sorting protein 9 (VPS9) domain | NA |
|  |  |  |  |  | TraesCS4B01G216000 | Serine/threonine-protein kinase | PF01453: D-mannose binding lectin; PF00954: S-locus glycoprotein domain; PF08276: PAN-like domain; PF00069: Protein kinase domain | GO:0004672 MF: protein kinase activity;GO:0004674 MF: protein serine/threonine kinase activity; |
|  |  |  |  |  | TraesCS4B01G216100 | Late embryogenesis abundant (LEA) hydroxyproline-rich glycoprotein family | PF03168: Late embryogenesis abundant protein | NA |
|  |  |  |  |  | TraesCS4B01G216200 | HVA22-like protein | PF03134: TB2/DP1, HVA22 family | NA |
| Relative root shoot ratio | AX-158582575 |  | 4B | 509912494 | TraesCS4B01G245900.5 | Protein CRABS CLAW, putative | PF04690: YABBY protein | GO:0007275 BP: multicellular organism development |
|  |  |  |  |  | TraesCS4B01G246000 | GDP-mannose transporter | PF03151: Triose-phosphate Transporter family | NA |
|  |  |  |  |  | TraesCS4B01G246100 | Mitotic checkpoint protein bub3.1 | PF00400: WD domain, G-beta repeat | GO:0005515 MF: protein binding |
|  |  |  |  |  | TraesCS4B01G246200 | tRNA-2-methylthio-N(6)-dimethylallyladenosine synthase | NA | NA |
|  |  |  |  |  | TraesCS4B01G246400 | Expansin | PF03330: Lytic transglycolase; PF01357: Pollen allergen | GO:0005576 CC: extracellular region;GO:0009664 BP: plant-type cell wall organization |
|  |  |  |  |  | TraesCS4B01G246500 | Expansin | PF03330: Lytic transglycolase; PF01357: Pollen allergen | GO:0005576 CC: extracellular region;GO:0009664 BP: plant-type cell wall organization |
|  |  |  |  |  | TraesCS4B01G246600 | Expansin | PF03330: Lytic transglycolase; PF01357: Pollen allergen | GO:0005576 CC: extracellular region;GO:0009664 BP: plant-type cell wall organization |
|  |  |  |  |  | TraesCS4B01G246700 | Digalactosyldiacylglycerol synthase (Chloroplastic) | PF13692: Glycosyl transferases group 1 | NA |
|  |  |  |  |  | TraesCS4B01G246800 | Ninja-family protein | PF07897: Ethylene-responsive binding factor-associated repression; PF16135: TPL-binding domain in jasmonate signalling | NA |
| Relative shoot length | AX-158582483 |  | 4B | 661256962 | TraesCS4B01G377100 | Germin-like protein | PF00190: Cupin | GO:0030145 MF: manganese ion binding;GO:0045735 MF: nutrient reservoir activity |
|  |  |  |  |  | TraesCS4B01G377200 | Germin-like protein | PF00190: Cupin | GO:0030145 MF: manganese ion binding;GO:0045735 MF: nutrient reservoir activity |
|  |  |  |  |  | TraesCS4B01G377300 | Germin-like protein | PF00190: Cupin | GO:0030145 MF: manganese ion binding;GO:0045735 MF: nutrient reservoir activity |
|  |  |  |  |  | TraesCS4B01G377400 | Germin-like protein | PF00190: Cupin | GO:0030145 MF: manganese ion binding;GO:0045735 MF: nutrient reservoir activity |
|  |  |  |  |  | TraesCS4B01G377500 | Germin-like protein | PF00190: Cupin | GO:0030145 MF: manganese ion binding;GO:0045735 MF: nutrient reservoir activity |
|  |  |  |  |  | TraesCS4B01G377600 | Phospholipase D | PF00168: C2 domain; PF00614: Phospholipase D Active site motif; PF12357: Phospholipase D C terminal | GO:0003824 MF: catalytic activity;GO:0004630 MF: phospholipase D activity; |
|  |  |  |  |  | TraesCS4B01G377700 | Germin-like protein | PF00190: Cupin | GO:0030145 MF: manganese ion binding;GO:0045735 MF: nutrient reservoir activity |
|  |  |  |  |  | TraesCS4B01G377800 | Germin-like protein | PF00190: Cupin | GO:0030145 MF: manganese ion binding;GO:0045735 MF: nutrient reservoir activity |
|  |  |  |  |  | TraesCS4B01G377900 | Germin-like protein | PF00190: Cupin | GO:0030145 MF: manganese ion binding;GO:0045735 MF: nutrient reservoir activity |
|  |  |  |  |  | TraesCS4B01G378000 | Germin-like protein | PF00190: Cupin | GO:0030145 MF: manganese ion binding;GO:0045735 MF: nutrient reservoir activity |
|  |  |  |  |  | TraesCS4B01G378100 | Germin-like protein | PF00190: Cupin | GO:0030145 MF: manganese ion binding;GO:0045735 MF: nutrient reservoir activity |
|  |  |  |  |  | TraesCS4B01G378200 | Germin-like protein | PF00190: Cupin | GO:0030145 MF: manganese ion binding;GO:0045735 MF: nutrient reservoir activity |
|  |  |  |  |  | TraesCS4B01G378300 | Germin-like protein | PF00190: Cupin | GO:0030145 MF: manganese ion binding;GO:0045735 MF: nutrient reservoir activity |
|  |  |  |  |  | TraesCS4B01G378400 | Germin-like protein | PF00190: Cupin | GO:0030145 MF: manganese ion binding;GO:0045735 MF: nutrient reservoir activity |
|  |  |  |  |  | TraesCS4B01G378500 | tRNA/rRNA methyltransferase family protein | PF00588: SpoU rRNA Methylase family | GO:0003723 MF: RNA binding;GO:0005488 MF: binding; |
|  |  |  |  |  | TraesCS4B01G378600 | NA | NA | NA |
|  |  |  |  |  | TraesCS4B01G378700 | DNA repair endonuclease UVH1 | PF02732: ERCC4 domain | GO:0003677 MF: DNA binding;GO:0004518 MF: nuclease activity |
|  |  |  |  |  | TraesCS4B01G378800 | Peptidyl-prolyl cis-trans isomerase | PF14295: PAN domain; PF00160: Cyclophilin type peptidyl-prolyl cis-trans isomerase/CLD | GO:0000413 BP: protein peptidyl-prolyl isomerization;GO:0003755 MF: peptidyl-prolyl cis-trans isomerase activity; |
|  |  |  |  |  | TraesCS4B01G378900 | NA | NA | NA |
|  |  |  |  |  | TraesCS4B01G379000 | Alkyl transferase | PF01255: Putative undecaprenyl diphosphate synthase | GO:0016765 MF: transferase activity, transferring alkyl or aryl (other than methyl) groups |
|  |  |  |  |  | TraesCS4B01G379100 | Protein FLOWERING LOCUS T | PF01161: Phosphatidylethanolamine-binding protein | NA |
|  |  |  |  |  | TraesCS4B01G379200 | Calcineurin B-like protein | PF13499: EF-hand domain pair | GO:0005509 MF: calcium ion binding |
|  |  |  |  |  | TraesCS4B01G379300 | Casein kinase II subunit beta | PF01214: Casein kinase II regulatory subunit | GO:0005956 CC: protein kinase CK2 complex;GO:0019887 MF: protein kinase regulator activity |
|  |  |  |  |  | TraesCS4B01G379400 | NA | NA | NA |
|  |  |  |  |  | TraesCS4B01G379500 | Pterin-4-alpha-carbinolamine dehydratase, putative | PF01329: Pterin 4 alpha carbinolamine dehydratase | GO:0006729 BP: tetrahydrobiopterin biosynthetic process;GO:0008124 MF: 4-alpha-hydroxytetrahydrobiopterin dehydratase activity |
|  |  |  |  |  | TraesCS4B01G379600 | Invertase inhibitor | PF04043: Plant invertase/pectin methylesterase inhibitor | GO:0004857 MF: enzyme inhibitor activity |
|  |  |  |  |  | TraesCS4B01G379700 | Phosphatidylinositol N-acetylglucosaminyltransferase subunit P | PF08510: PIG-P | NA |
|  |  |  |  |  | TraesCS4B01G379800 | Man1-Src1p-carboxy-terminal domain protein | PF09402: Man1-Src1p-C-terminal domain | GO:0005639 CC: integral component of nuclear inner membrane |
|  |  |  |  |  | TraesCS4B01G379900 | Ribose-phosphate pyrophosphokinase | PF13793: N-terminal domain of ribose phosphate pyrophosphokinase; PF14572: Phosphoribosyl synthetase-associated domain | GO:0000287 MF: magnesium ion binding;GO:0004749 MF: ribose phosphate diphosphokinase activity; |
|  |  |  |  |  | TraesCS4B01G380000 | Protein NRT1/ PTR FAMILY 5.1 | PF00854: POT family | GO:0005215 MF: transporter activity;GO:0006810 BP: transport; |
|  |  |  |  |  | TraesCS4B01G380100 | C2 domain-containing family protein | PF00168: C2 domain | GO:0005515 MF: protein binding |
|  |  |  |  |  | TraesCS4B01G380200 | CAAX protease self-immunity protein | PF02517: CAAX protease self-immunity | GO:0016020 CC: membrane |
|  |  |  |  |  | TraesCS4B01G380300 | glycosyltransferase family exostosin protein | PF03016: Exostosin family | NA |
|  |  |  |  |  | TraesCS4B01G380400 | Arsenate reductase | PF00581: Rhodanese-like domain | NA |
|  |  |  |  |  | TraesCS4B01G380500 | Protein FLOWERING LOCUS T | NA | NA |
|  |  |  |  |  | TraesCS4B01G380600 | Protein FLOWERING LOCUS T | PF01161: Phosphatidylethanolamine-binding protein | NA |
|  |  |  |  |  | TraesCS4B01G380700 | Trichome birefringence-like protein | PF14416: PMR5 N terminal Domain; PF13839: GDSL/SGNH-like Acyl-Esterase family found in Pmr5 and Cas1p | NA |
|  |  |  |  |  | TraesCS4B01G380800 | ATP-dependent chaperone ClpB | PF02861: Clp amino terminal domain, pathogenicity island component; PF00004: ATPase family associated with various cellular activities (AAA); PF07724: AAA domain (Cdc48 subfamily); PF10431: C-terminal, D2-small domain, of ClpB protein | GO:0005524 MF: ATP binding;GO:0005737 CC: cytoplasm; |
|  |  |  |  |  | TraesCS4B01G380900 | RING/U-box superfamily protein | PF13445: RING-type zinc-finger | GO:0005515 MF: protein binding;GO:0008270 MF: zinc ion binding |
|  |  |  |  |  | TraesCS4B01G381000 | RING-finger ubiquitin ligase | PF13445: RING-type zinc-finger | GO:0005515 MF: protein binding;GO:0008270 MF: zinc ion binding |
|  |  |  |  |  | TraesCS4B01G381100 | RING/U-box superfamily protein | PF13639: Ring finger domain | GO:0005515 MF: protein binding;GO:0008270 MF: zinc ion binding |
|  |  |  |  |  | TraesCS4B01G381200 | Divalent metal cation transporter MntH | PF01566: Natural resistance-associated macrophage protein | GO:0005215 MF: transporter activity;GO:0006810 BP: transport; |
|  |  |  |  |  | TraesCS4B01G381300 | Carboxyl methyltransferase | PF03492: SAM dependent carboxyl methyltransferase | GO:0008168 MF: methyltransferase activity |
|  |  |  |  |  | TraesCS4B01G381400 | Cortactin-binding protein 2 | NA | NA |
|  |  |  |  |  | TraesCS4B01G381500 | Apyrase | PF01150: GDA1/CD39 (nucleoside phosphatase) family | GO:0016787 MF: hydrolase activity |
|  |  |  |  |  | TraesCS4B01G381600 | Apyrase | PF01150: GDA1/CD39 (nucleoside phosphatase) family | GO:0016787 MF: hydrolase activity |
|  |  |  |  |  | TraesCS4B01G381700 | Protein B602L | NA | NA |
|  |  |  |  |  | TraesCS4B01G381800 | Metacaspase-1 | PF00656: Caspase domain | NA |
|  |  |  |  |  | TraesCS4B01G381900 | Protein phosphatase 2c, putative | PF00481: Protein phosphatase 2C | GO:0003824 MF: catalytic activity |
|  |  |  |  |  | TraesCS4B01G382000 | RING/U-box superfamily protein | PF13639: Ring finger domain | GO:0005515 MF: protein binding;GO:0008270 MF: zinc ion binding |
|  |  |  |  |  | TraesCS4B01G382100 | Leucine-rich repeat receptor-like protein kinase family protein | PF08263: Leucine rich repeat N-terminal domain; PF13855: Leucine rich repeat | GO:0005515 MF: protein binding |
|  |  |  |  |  | TraesCS4B01G382200 | Non-specific lipid-transfer protein | PF00234: Protease inhibitor/seed storage/LTP family | GO:0006869 BP: lipid transport;GO:0008289 MF: lipid binding |
|  |  |  |  |  | TraesCS4B01G382300 | Cytochrome P450 | PF00067: Cytochrome P450 | GO:0005506 MF: iron ion binding;GO:0016705 MF: oxidoreductase activity, acting on paired donors, with incorporation or reduction of molecular oxygen; |
|  |  |  |  |  | TraesCS4B01G382400 | Phosphatase 2C family protein | PF00481: Protein phosphatase 2C | GO:0003824 MF: catalytic activity |
|  |  |  |  |  | TraesCS4B01G382500 | Protein phosphatase 2C | NA | GO:0003824 MF: catalytic activity |
|  |  |  |  |  | TraesCS4B01G382600 | Disease resistance protein (NBS-LRR class) family | PF00931: NB-ARC domain | GO:0043531 MF: ADP binding |
|  |  |  |  |  | TraesCS4B01G382700 | BAG family molecular chaperone regulator 6 | PF02179: BAG domain | GO:0005515 MF: protein binding;GO:0051087 MF: chaperone binding |
|  |  |  |  |  | TraesCS4B01G382800 | Dirigent protein | PF03018: Dirigent-like protein | NA |
| Relative_SFW | AX-108742709 |  | 5A | 578066076 | TraesCS5A01G381000 | WRKY transcription factor | PF10533: Plant zinc cluster domain; PF03106: WRKY DNA -binding domain | GO:0003700 MF: transcription factor activity, sequence-specific DNA binding;GO:0006355 BP: regulation of transcription, DNA-templated; |
|  |  |  |  |  | TraesCS5A01G381100 | Cytochrome P450 family protein, expressed | PF00067: Cytochrome P450 | GO:0005506 MF: iron ion binding;GO:0016705 MF: oxidoreductase activity, acting on paired donors, with incorporation or reduction of molecular oxygen; |
|  |  |  |  |  | TraesCS5A01G381200 | PRA1 family protein | PF03208: PRA1 family protein | NA |
| Relative shoot fresh weight | AX-108744896 |  | 5A | 577905618 | TraesCS5A01G379500 | Chalcone synthase | PF00195: Chalcone and stilbene synthases, N-terminal domain; PF02797: Chalcone and stilbene synthases, C-terminal domain | GO:0003824 MF: catalytic activity;GO:0008152 BP: metabolic process; |
|  |  |  |  |  | TraesCS5A01G379600 | Chalcone synthase | PF00195: Chalcone and stilbene synthases, N-terminal domain; PF02797: Chalcone and stilbene synthases, C-terminal domain | GO:0003824 MF: catalytic activity;GO:0008152 BP: metabolic process; |
|  |  |  |  |  | TraesCS5A01G379700 | Chalcone synthase | PF00195: Chalcone and stilbene synthases, N-terminal domain; PF02797: Chalcone and stilbene synthases, C-terminal domain | GO:0003824 MF: catalytic activity;GO:0008152 BP: metabolic process; |
|  |  |  |  |  | TraesCS5A01G379800 | Structural constituent of ribosome protein | PF03350: Uncharacterized protein family, UPF0114 | NA |
|  |  |  |  |  | TraesCS5A01G379900 | DUF4228 domain protein | PF14009: Domain of unknown function (DUF4228) | NA |
|  |  |  |  |  | TraesCS5A01G380000 | Dirigent protein | PF03018: Dirigent-like protein | NA |
|  |  |  |  |  | TraesCS5A01G380100 | Deoxyhypusine synthase, putative, expressed | PF01916: Deoxyhypusine synthase | GO:0008612 BP: peptidyl-lysine modification to peptidyl-hypusine |
| Relative shoot fresh weight | AX-110382510 |  | 5A | 577905566 | TraesCS5A01G379500 | Chalcone synthase | PF00195: Chalcone and stilbene synthases, N-terminal domain; PF02797: Chalcone and stilbene synthases, C-terminal domain | GO:0003824 MF: catalytic activity;GO:0008152 BP: metabolic process; |
|  |  |  |  |  | TraesCS5A01G379600 | Chalcone synthase | PF00195: Chalcone and stilbene synthases, N-terminal domain; PF02797: Chalcone and stilbene synthases, C-terminal domain | GO:0003824 MF: catalytic activity;GO:0008152 BP: metabolic process; |
|  |  |  |  |  | TraesCS5A01G379700 | Chalcone synthase | PF00195: Chalcone and stilbene synthases, N-terminal domain; PF02797: Chalcone and stilbene synthases, C-terminal domain | GO:0003824 MF: catalytic activity;GO:0008152 BP: metabolic process; |
|  |  |  |  |  | TraesCS5A01G379800 | Structural constituent of ribosome protein | PF03350: Uncharacterized protein family, UPF0114 | NA |
|  |  |  |  |  | TraesCS5A01G379900 | DUF4228 domain protein | PF14009: Domain of unknown function (DUF4228) | NA |
|  |  |  |  |  | TraesCS5A01G380000 | Dirigent protein | PF03018: Dirigent-like protein | NA |
|  |  |  |  |  | TraesCS5A01G380100 | Deoxyhypusine synthase, putative, expressed | PF01916: Deoxyhypusine synthase | GO:0008612 BP: peptidyl-lysine modification to peptidyl-hypusine |
|  |  |  |  |  | TraesCS5A01G380200 | F-box protein | PF00646: F-box domain | GO:0005515 MF: protein binding |
|  |  |  |  |  | TraesCS5A01G380300 | Deoxyhypusine synthase | PF01916: Deoxyhypusine synthase | GO:0008612 BP: peptidyl-lysine modification to peptidyl-hypusine |
|  |  |  |  |  | TraesCS5A01G380400 | CWF19-like protein 2 | PF04677: Protein similar to CwfJ C-terminus 1; PF04676: Protein similar to CwfJ C-terminus 2 | GO:0003824 MF: catalytic activity |
|  |  |  |  |  | TraesCS5A01G380500 | LEM3 (Ligand-effect modulator 3)-like | PF03381: LEM3 (ligand-effect modulator 3) family / CDC50 family | GO:0016020 CC: membrane |
|  |  |  |  |  | TraesCS5A01G380600 | Vacuolar sorting-associated protein 18-like protein | PF05131: Pep3/Vps18/deep orange family; PF00637: Region in Clathrin and VPS | GO:0005515 MF: protein binding;GO:0006886 BP: intracellular protein transport; |
|  |  |  |  |  | TraesCS5A01G380700 | Disease resistance protein (NBS-LRR class) family | PF00931: NB-ARC domain | GO:0043531 MF: ADP binding |
|  |  |  |  |  | TraesCS5A01G380800 | DNA repair protein XRCC4 | PF06632: DNA double-strand break repair and V(D)J recombination protein XRCC4 | GO:0003677 MF: DNA binding;GO:0005634 CC: nucleus; |
|  |  |  |  |  | TraesCS5A01G380900 | Basic helix-loop-helix transcription factor | NA | GO:0046983 MF: protein dimerization activity |
|  |  |  |  |  | TraesCS5A01G381000 | WRKY transcription factor | PF10533: Plant zinc cluster domain; PF03106: WRKY DNA -binding domain | GO:0003700 MF: transcription factor activity, sequence-specific DNA binding;GO:0006355 BP: regulation of transcription, DNA-templated; |
|  |  |  |  |  | TraesCS5A01G381100 | Cytochrome P450 family protein, expressed | PF00067: Cytochrome P450 | GO:0005506 MF: iron ion binding;GO:0016705 MF: oxidoreductase activity, acting on paired donors, with incorporation or reduction of molecular oxygen; |
| Relative shoot fresh weight | AX-89769139 |  | 5A | 578055910 | TraesCS5A01G380500 | LEM3 (Ligand-effect modulator 3)-like | PF03381: LEM3 (ligand-effect modulator 3) family / CDC50 family | GO:0016020 CC: membrane |
|  |  |  |  |  | TraesCS5A01G380600 | Vacuolar sorting-associated protein 18-like protein | PF05131: Pep3/Vps18/deep orange family; PF00637: Region in Clathrin and VPS | GO:0005515 MF: protein binding;GO:0006886 BP: intracellular protein transport; |
|  |  |  |  |  | TraesCS5A01G380700 | Disease resistance protein (NBS-LRR class) family | PF00931: NB-ARC domain | GO:0043531 MF: ADP binding |
|  |  |  |  |  | TraesCS5A01G380800 | DNA repair protein XRCC4 | PF06632: DNA double-strand break repair and V(D)J recombination protein XRCC4 | GO:0003677 MF: DNA binding;GO:0005634 CC: nucleus; |
| Relative shoot fresh weight | BS00074299_51 |  | 5A | 578061350 | TraesCS5A01G380800 | DNA repair protein XRCC4 | PF06632: DNA double-strand break repair and V(D)J recombination protein XRCC4 | GO:0003677 MF: DNA binding;GO:0005634 CC: nucleus; |
| Relative shoot fresh weight | BS00076246_51 |  | 5A | 578058139 | TraesCS5A01G380800 | DNA repair protein XRCC4 | PF06632: DNA double-strand break repair and V(D)J recombination protein XRCC4 | GO:0003677 MF: DNA binding;GO:0005634 CC: nucleus; |
| Relative shoot fresh weight | Ku_c19858_2078 |  | 5A | 577942755 | TraesCS5A01G380200 | F-box protein | PF00646: F-box domain | GO:0005515 MF: protein binding |
|  |  |  |  |  | TraesCS5A01G380300 | Deoxyhypusine synthase | PF01916: Deoxyhypusine synthase | GO:0008612 BP: peptidyl-lysine modification to peptidyl-hypusine |
|  |  |  |  |  | TraesCS5A01G380400 | CWF19-like protein 2 | PF04677: Protein similar to CwfJ C-terminus 1; PF04676: Protein similar to CwfJ C-terminus 2 | GO:0003824 MF: catalytic activity |
| Relative shoot fresh weight | Tdurum_contig86202_175 | 5A | 578063880 | TraesCS5A01G380700 |  | Disease resistance protein (NBS-LRR class) family | PF00931: NB-ARC domain | GO:0043531 MF: ADP binding |
|  |  |  |  | TraesCS5A01G380800 | DNA repair protein XRCC4 | PF06632: DNA double-strand break repair and V(D)J recombination protein XRCC4 | GO:0003677 MF: DNA binding;GO:0005634 CC: nucleus; |  |
| Relative_SL | AX-109884177 |  | 5A | 37843077 | TraesCS5A01G040900 | BTB/POZ domain containing protein, expressed | PF00651: BTB/POZ domain | GO:0005515 MF: protein binding |
|  |  |  |  |  | TraesCS5A01G041000 | BTB/POZ and MATH domain-containing protein 1 | PF00917: MATH domain; PF00651: BTB/POZ domain | GO:0005515 MF: protein binding |
|  |  |  |  |  | TraesCS5A01G041100 | Guanosine nucleotide diphosphate dissociation inhibitor | PF00996: GDP dissociation inhibitor | GO:0005092 MF: GDP-dissociation inhibitor activity;GO:0005093 MF: Rab GDP-dissociation inhibitor activity; |
|  |  |  |  |  | TraesCS5A01G041200 | F-box domain containing protein, expressed | NA | NA |
|  |  |  |  |  | TraesCS5A01G041300 | BTB/POZ and MATH domain-containing protein 2 | PF00917: MATH domain; PF00651: BTB/POZ domain | GO:0005515 MF: protein binding |
|  |  |  |  |  | TraesCS5A01G041400 | BTB/POZ and MATH domain-containing protein 2 | PF00917: MATH domain; PF00651: BTB/POZ domain | GO:0005515 MF: protein binding |
|  |  |  |  |  | TraesCS5A01G041500 | MYB-related transcription factor | PF00249: Myb-like DNA-binding domain; PF00538: linker histone H1 and H5 family | GO:0000786 CC: nucleosome;GO:0003677 MF: DNA binding; |
|  |  |  |  |  | TraesCS5A01G041600 | Pentatricopeptide repeat superfamily protein | PF01535: PPR repeat | GO:0005515 MF: protein binding |
|  |  |  |  |  | TraesCS5A01G041700 | FBD, F-box and Leucine Rich Repeat domains containing protein | PF07762: Protein of unknown function (DUF1618) | NA |
|  |  |  |  |  | TraesCS5A01G041800 | Vacuolar fusion protein CCZ1 | PF08217: Fungal domain of unknown function (DUF1712) | NA |
|  |  |  |  |  | TraesCS5A01G041900 | ternary complex factor MIP1 leucine-zipper protein (Protein of unknown function, DUF547) | NA | NA |
|  |  |  |  |  | TraesCS5A01G042000 | BTB/POZ domain containing protein | PF00917: MATH domain; PF00651: BTB/POZ domain | GO:0005515 MF: protein binding |
|  |  |  |  |  | TraesCS5A01G042100 | Embryogenesis transmembrane protein-like | PF13962: Domain of unknown function | NA |
|  |  |  |  |  | TraesCS5A01G042200 | Meiosis-specific protein PAIR3 | NA | NA |
|  |  |  |  |  | TraesCS5A01G042300 | RNA helicase | PF00270: DEAD/DEAH box helicase; PF00271: Helicase conserved C-terminal domain; PF13959: Domain of unknown function (DUF4217) | GO:0003676 MF: nucleic acid binding;GO:0005524 MF: ATP binding |
|  |  |  |  |  | TraesCS5A01G042400 | Eukaryotic translation initiation factor 3 subunit A | PF01399: PCI domain | GO:0005515 MF: protein binding |
|  |  |  |  |  | TraesCS5A01G042500 | Protein TRIGALACTOSYLDIACYLGLYCEROL 2, chloroplastic | PF02470: MlaD protein | NA |
|  |  |  |  |  | TraesCS5A01G042600 | BTB/POZ domain-containing protein | PF00651: BTB/POZ domain; PF03000: NPH3 family | GO:0005515 MF: protein binding |
|  |  |  |  |  | TraesCS5A01G042700 | Histone-lysine N-methyltransferase | PF00856: SET domain | GO:0005515 MF: protein binding |
|  |  |  |  |  | TraesCS5A01G042800 | Glutamate receptor | PF01094: Receptor family ligand binding region; PF00497: Bacterial extracellular solute-binding proteins, family 3; PF00060: Ligand-gated ion channel | GO:0004930 MF: G-protein coupled receptor activity;GO:0004970 MF: ionotropic glutamate receptor activity; |
|  |  |  |  |  | TraesCS5A01G042900 | Cationic amino acid transporter, putative | PF13520: Amino acid permease; PF13906: C-terminus of AA_permease | GO:0003333 BP: amino acid transmembrane transport;GO:0015171 MF: amino acid transmembrane transporter activity; |
|  |  |  |  |  | TraesCS5A01G043000 | Heavy metal transport/detoxification superfamily protein, putative | PF00403: Heavy-metal-associated domain | GO:0030001 BP: metal ion transport;GO:0046872 MF: metal ion binding |
|  |  |  |  |  | TraesCS5A01G043100 | WD40 repeat-like protein | PF00400: WD domain, G-beta repeat | GO:0005515 MF: protein binding |
| Relative root fresh weight | Tdurum_contig82473_67 | 5B | 620827424 | TraesCS5B01G447800 | Cytosine-specific methyltransferase | PF12047: Cytosine specific DNA methyltransferase replication foci domain; PF01426: BAH domain; PF00145: C-5 cytosine-specific DNA methylase | GO:0003682 MF: chromatin binding;GO:0008168 MF: methyltransferase activity | Relative_RFW |
|  |  |  |  |  | TraesCS5B01G447900 | PRA1 family protein | PF03208: PRA1 family protein | NA |
|  |  |  |  |  | TraesCS5B01G448000 | Expansin | PF03330: Lytic transglycolase; PF01357: Pollen allergen | GO:0005576 CC: extracellular region;GO:0009664 BP: plant-type cell wall organization |
|  |  |  |  |  | TraesCS5B01G448100 | 30S ribosomal protein S19 | PF00203: Ribosomal protein S19 | GO:0003723 MF: RNA binding;GO:0003735 MF: structural constituent of ribosome; |
|  |  |  |  |  | TraesCS5B01G448200 | Histone H1 | PF00538: linker histone H1 and H5 family | GO:0000786 CC: nucleosome;GO:0003677 MF: DNA binding; |
|  |  |  |  |  | TraesCS5B01G448300 | Kallikrein 1-related peptidase b21 | NA | NA |
|  |  |  |  |  | TraesCS5B01G448400 | Histone H1 | PF00538: linker histone H1 and H5 family | GO:0000786 CC: nucleosome;GO:0003677 MF: DNA binding; |
|  |  |  |  |  | TraesCS5B01G448500 | Histone H1 | PF00538: linker histone H1 and H5 family | GO:0000786 CC: nucleosome;GO:0003677 MF: DNA binding; |
|  |  |  |  |  | TraesCS5B01G448600 | Kinase-like | PF00069: Protein kinase domain | GO:0004672 MF: protein kinase activity;GO:0005524 MF: ATP binding; |
|  |  |  |  |  | TraesCS5B01G448700 | Mitochondrial transcription termination factor-like | PF02536: mTERF | GO:0003690 MF: double-stranded DNA binding;GO:0006355 BP: regulation of transcription, DNA-templated |
|  |  |  |  |  | TraesCS5B01G448800 | myosin-binding protein (Protein of unknown function, DUF593) | PF04576: Zein-binding | NA |
|  |  |  |  |  | TraesCS5B01G448900 | TPX2 (Targeting protein for Xklp2) family protein | PF06886: Targeting protein for Xklp2 (TPX2) | NA |
|  |  |  |  |  | TraesCS5B01G449000 | Histone H1 | PF00538: linker histone H1 and H5 family | GO:0000786 CC: nucleosome;GO:0003677 MF: DNA binding; |
|  |  |  |  |  | TraesCS5B01G449100 | Elongator complex protein 1 | NA | NA |
|  |  |  |  |  | TraesCS5B01G449200 | Receptor-like protein kinase | PF13947: Wall-associated receptor kinase galacturonan-binding; PF00069: Protein kinase domain | GO:0004672 MF: protein kinase activity;GO:0005509 MF: calcium ion binding; |
|  |  |  |  |  | TraesCS5B01G449300 | Receptor-like protein kinase | PF13947: Wall-associated receptor kinase galacturonan-binding; PF00069: Protein kinase domain | GO:0004672 MF: protein kinase activity;GO:0005509 MF: calcium ion binding; |
|  |  |  |  |  | TraesCS5B01G449400 | Divalent ion symporter | NA | NA |
| Relative root fresh weight | BS00065783_51 |  | 5D | 69456300 | TraesCS5D01G070700 | WRKY transcription factor | PF10533: Plant zinc cluster domain; PF03106: WRKY DNA -binding domain | GO:0003700 MF: transcription factor activity, sequence-specific DNA binding;GO:0006355 BP: regulation of transcription, DNA-templated;G |
|  |  |  |  |  | TraesCS5D01G070800 | Aminotransferase | PF00155: Aminotransferase class I and II | GO:0003824 MF: catalytic activity;GO:0009058 BP: biosynthetic process; |
|  |  |  |  |  | TraesCS5D01G070900 | GTP 3',8-cyclase | PF04055: Radical SAM superfamily; PF13353: 4Fe-4S single cluster domain; PF06463: Molybdenum Cofactor Synthesis C | GO:0003824 MF: catalytic activity;GO:0006777 BP: Mo-molybdopterin cofactor biosynthetic process; |
|  |  |  |  |  | TraesCS5D01G071000 | RNA-binding KH domain-containing protein | PF00013: KH domain | GO:0003676 MF: nucleic acid binding;GO:0003723 MF: RNA binding |
|  |  |  |  |  | TraesCS5D01G071100 | N-carbamyl-L-amino acid amidohydrolase | PF01546: Peptidase family M20/M25/M40 | GO:0008152 BP: metabolic process;GO:0016787 MF: hydrolase activity; |
|  |  |  |  |  | TraesCS5D01G071200 | Cinnamoyl-CoA reductase 4 | PF01370: NAD dependent epimerase/dehydratase family | GO:0003824 MF: catalytic activity;GO:0050662 MF: coenzyme binding |
|  |  |  |  |  | TraesCS5D01G071300 | FAD/NAD(P)-binding oxidoreductase family protein | PF13450: NAD(P)-binding Rossmann-like domain | GO:0016491 MF: oxidoreductase activity;GO:0055114 BP: oxidation-reduction process |
|  |  |  |  |  | TraesCS5D01G071400 | Gamma-tubulin complex component | PF04130: Spc97 / Spc98 family | GO:0000226 BP: microtubule cytoskeleton organization;GO:0000922 CC: spindle pole; |
|  |  |  |  |  | TraesCS5D01G071500 | WAT1-related protein | PF00892: EamA-like transporter family | GO:0016020 CC: membrane;GO:0016021 CC: integral component of membrane |
|  |  |  |  |  | TraesCS5D01G071600 | Splicing factor U2AF, large subunit | NA | GO:0003676 MF: nucleic acid binding |
|  |  |  |  |  | TraesCS5D01G071700 | Splicing factor U2AF, large subunit | NA | GO:0003676 MF: nucleic acid binding |
| Relative_RL | AX-158600273 |  | 6A | 520581021 | TraesCS6A01G287400 | Nuclear transcription factor Y subunit B | PF00808: Histone-like transcription factor (CBF/NF-Y) and archaeal histone | GO:0046982 MF: protein heterodimerization activity |
|  |  |  |  |  | TraesCS6A01G287500 | Nuclear transcription factor Y subunit B | NA | NA |
|  |  |  |  |  | TraesCS6A01G287600 | Fasciclin-like arabinogalactan protein | PF02469: Fasciclin domain | NA |
|  |  |  |  |  | TraesCS6A01G287700 | Dof zinc finger protein | PF02701: Dof domain, zinc finger | GO:0003677 MF: DNA binding;GO:0006355 BP: regulation of transcription, DNA-templated |
|  |  |  |  |  | TraesCS6A01G287800 | BSD domain containing protein | PF03909: BSD domain | NA |
|  |  |  |  |  | TraesCS6A01G287900 | O-fucosyltransferase family protein | PF10250: GDP-fucose protein O-fucosyltransferase | NA |
|  |  |  |  |  | TraesCS6A01G288000 | Acetolactate synthase | PF02776: Thiamine pyrophosphate enzyme, N-terminal TPP binding domain; PF00205: Thiamine pyrophosphate enzyme, central domain; PF02775: Thiamine pyrophosphate enzyme, C-terminal TPP binding domain | GO:0000287 MF: magnesium ion binding;GO:0003824 MF: catalytic activity; |
|  |  |  |  |  | TraesCS6A01G288100 | Basic helix-loop-helix transcription factor | PF00010: Helix-loop-helix DNA-binding domain | GO:0046983 MF: protein dimerization activity |
|  |  |  |  |  | TraesCS6A01G288200 | Amino acid transporter family protein | PF01490: Transmembrane amino acid transporter protein | NA |
| Relative_RL | AX-158600281 |  | 6A | 520712811 | TraesCS6A01G288300 | 2-oxoglutarate (2OG) and Fe(II)-dependent oxygenase superfamily protein | PF14226: non-haem dioxygenase in morphine synthesis N-terminal; PF03171: 2OG-Fe(II) oxygenase superfamily | GO:0016491 MF: oxidoreductase activity;GO:0055114 BP: oxidation-reduction process |
| Relative_RL |  |  | 3B | 115785002 | TraesCS6A01G288400 | 2-oxoglutarate (2OG) and Fe(II)-dependent oxygenase superfamily protein | PF14226: non-haem dioxygenase in morphine synthesis N-terminal; PF03171: 2OG-Fe(II) oxygenase superfamily | GO:0016491 MF: oxidoreductase activity;GO:0055114 BP: oxidation-reduction process |
| Relative_RFW | AX-158528874 | Rel_RFW_6B_Hap1 | 6B | 720513279 | TraesCS6B01G473000 | Mitochondrial intermediate peptidase | PF01432: Peptidase family M3 | GO:0004222 MF: metalloendopeptidase activity;GO:0006508 BP: proteolysis |
|  | BS00011795_51 | Rel_RFW_6B_Hap1 | 6B | 720560817 | TraesCS6B01G473100 | F-box protein family | PF08268: F-box associated domain | NA |
|  |  |  |  |  | TraesCS6B01G473200 | Pectinesterase inhibitor | PF04043: Plant invertase/pectin methylesterase inhibitor | GO:0004857 MF: enzyme inhibitor activity |
|  |  |  |  |  | TraesCS6B01G473300 | Plant invertase/pectin methylesterase inhibitor superfamily protein | NA | GO:0004857 MF: enzyme inhibitor activity |
|  |  |  |  |  | TraesCS6B01G473400 | heparan-alpha-glucosaminide N-acetyltransferase-like protein (DUF1624) | PF11267: Domain of unknown function (DUF3067) | NA |
|  |  |  |  |  | TraesCS6B01G473500 | Pentatricopeptide repeat-containing protein | PF01535: PPR repeat; PF13812: Pentatricopeptide repeat domain; PF12854: PPR repeat | GO:0005515 MF: protein binding |
|  |  |  |  |  | TraesCS6B01G473600 | multidrug resistance-associated protein 3 | NA | NA |
|  |  |  |  |  | TraesCS6B01G473700 | Methylthioribose-1-phosphate isomerase | NA | NA |
|  |  |  |  |  | TraesCS6B01G473800 | Thaumatin-like protein | PF00314: Thaumatin family | NA |
| Relative_RFW | Excalibur_c28759_914 |  | 6B | 716259047 | TraesCS6B01G462100 | Transcription factor, putative | PF03634: TCP family transcription factor | NA |
|  |  |  |  |  | TraesCS6B01G462200 | vesicle-associated protein 1-4 | NA | NA |
|  |  |  |  |  | TraesCS6B01G462300 | Peroxidase | PF00141: Peroxidase | GO:0004601 MF: peroxidase activity;GO:0006979 BP: response to oxidative stress; |
|  |  |  |  |  | TraesCS6B01G462400 | Gibberellin-regulated family protein | PF02704: Gibberellin regulated protein | NA |
|  |  |  |  |  | TraesCS6B01G462500 | 4-hydroxybenzoate octaprenyltransferase | PF01040: UbiA prenyltransferase family | GO:0004659 MF: prenyltransferase activity;GO:0006744 BP: ubiquinone biosynthetic process; |
|  |  |  |  |  | TraesCS6B01G462600 | Receptor-kinase, putative | PF08263: Leucine rich repeat N-terminal domain; PF00560: Leucine Rich Repeat; PF00069: Protein kinase domain | GO:0004672 MF: protein kinase activity;GO:0005515 MF: protein binding; |
|  |  |  |  |  | TraesCS6B01G462700 | Replication protein A 32 kDa subunit | PF08784: Replication protein A C terminal | GO:0003677 MF: DNA binding;GO:0005634 CC: nucleus; |
|  |  |  |  |  | TraesCS6B01G462800 | Ripening-related protein | NA | NA |
|  |  |  |  |  | TraesCS6B01G462900 | Tyrosine--tRNA ligase | PF00579: tRNA synthetases class I (W and Y) | GO:0000166 MF: nucleotide binding;GO:0004812 MF: aminoacyl-tRNA ligase activity; |
|  |  |  |  |  | TraesCS6B01G463000 | Calcium-dependent lipid-binding (CaLB domain) family protein | PF00168: C2 domain | GO:0005515 MF: protein binding |
|  |  |  |  |  | TraesCS6B01G463100 | UDP-N-acetylmuramyl-tripeptide synthetase | NA | NA |
|  |  |  |  |  | TraesCS6B01G463200 | F-box protein | PF00646: F-box domain | GO:0005515 MF: protein binding |
|  |  |  |  |  | TraesCS6B01G463300 | Glutamate--tRNA ligase | NA | NA |
|  |  |  |  |  | TraesCS6B01G463400 | Disease resistance protein RPM1 | NA | NA |
|  |  |  |  |  | TraesCS6B01G463500 | Disease resistance protein RPM1 | PF00931: NB-ARC domain | GO:0043531 MF: ADP binding |
|  |  |  |  |  | TraesCS6B01G463600 | Disease resistance protein RPM1 | PF00931: NB-ARC domain | GO:0043531 MF: ADP binding |
|  |  |  |  |  | TraesCS6B01G463700 | Disease resistance protein RPM1 | PF00931: NB-ARC domain | GO:0043531 MF: ADP binding |
|  |  |  |  |  | TraesCS6B01G463800 | Disease resistance protein (NBS-LRR class) family | PF00931: NB-ARC domain | GO:0043531 MF: ADP binding |
|  |  |  |  |  | TraesCS6B01G463900 | Disease resistance protein RPM1 | PF00931: NB-ARC domain | GO:0043531 MF: ADP binding |
|  |  |  |  |  | TraesCS6B01G464000 | Disease resistance protein RPM1 | PF00931: NB-ARC domain | GO:0043531 MF: ADP binding |
|  |  |  |  |  | TraesCS6B01G464100 | Disease resistance protein RPM1 | NA | NA |
|  |  |  |  |  | TraesCS6B01G464200 | Disease resistance protein RPM1 | PF00931: NB-ARC domain | GO:0043531 MF: ADP binding |
|  |  |  |  |  | TraesCS6B01G464300 | Disease resistance protein RPM1 | PF00931: NB-ARC domain | GO:0043531 MF: ADP binding |
|  |  |  |  |  | TraesCS6B01G464400 | Disease resistance protein RPM1 | PF00931: NB-ARC domain | GO:0043531 MF: ADP binding |
|  |  |  |  |  | TraesCS6B01G464500 | Disease resistance protein RPM1 | PF00931: NB-ARC domain | GO:0043531 MF: ADP binding |
|  |  |  |  |  | TraesCS6B01G464600 | NADH-ubiquinone oxidoreductase chain 6 | PF00499: NADH-ubiquinone/plastoquinone oxidoreductase chain 6 | GO:0008137 MF: NADH dehydrogenase (ubiquinone) activity;GO:0055114 BP: oxidation-reduction process |
|  |  |  |  |  | TraesCS6B01G464700 | Ephrin type-A receptor 1 | NA | NA |
|  |  |  |  |  | TraesCS6B01G464800 | 12-oxophytodienoate reductase-like protein | PF00724: NADH:flavin oxidoreductase / NADH oxidase family | GO:0003824 MF: catalytic activity;GO:0010181 MF: FMN binding; |
|  |  |  |  |  | TraesCS6B01G464900 | Disease resistance protein RPM1 | PF00931: NB-ARC domain | GO:0043531 MF: ADP binding |
|  |  |  |  |  | TraesCS6B01G465000 | Disease resistance protein (NBS-LRR class) family | PF00931: NB-ARC domain | GO:0043531 MF: ADP binding |
|  |  |  |  |  | TraesCS6B01G465100 | Disease resistance protein RPM1 | PF00931: NB-ARC domain | GO:0043531 MF: ADP binding |
|  |  |  |  |  | TraesCS6B01G465200 | Kinesin-like protein | NA | NA |
|  |  |  |  |  | TraesCS6B01G465300 | AP-5 complex subunit beta-1 | NA | NA |
|  |  |  |  |  | TraesCS6B01G465400 | transmembrane protein, putative (DUF594) | PF13968: Domain of unknown function (DUF4220) | NA |
|  |  |  |  |  | TraesCS6B01G465500 | transmembrane protein, putative (DUF594) | PF04578: Protein of unknown function, DUF594 | NA |
|  |  |  |  |  | TraesCS6B01G465600 | Protein kinase family protein | PF00069: Protein kinase domain | GO:0004672 MF: protein kinase activity;GO:0005515 MF: protein binding; |
|  |  |  |  |  | TraesCS6B01G465700 | receptor kinase 1 | PF00069: Protein kinase domain; PF14299: Phloem protein 2 | GO:0004672 MF: protein kinase activity;GO:0005524 MF: ATP binding; |
|  |  |  |  |  | TraesCS6B01G465800 | Disease resistance protein RPM1 | PF00931: NB-ARC domain | GO:0043531 MF: ADP binding |
|  |  |  |  |  | TraesCS6B01G465900 | BTB/POZ domain containing protein | PF00917: MATH domain; PF00651: BTB/POZ domain | GO:0005515 MF: protein binding |
|  |  |  |  |  | TraesCS6B01G466000 | receptor kinase 2 | PF00069: Protein kinase domain | GO:0004672 MF: protein kinase activity;GO:0005515 MF: protein binding; |
|  |  |  |  |  | TraesCS6B01G466100 | transmembrane protein, putative (DUF247) | PF03140: Plant protein of unknown function | NA |
|  |  |  |  |  | TraesCS6B01G466200 | Inhibitor protein | PF00280: Potato inhibitor I family | GO:0004867 MF: serine-type endopeptidase inhibitor activity;GO:0009611 BP: response to wounding |
|  |  |  |  |  | TraesCS6B01G466300 | Inhibitor protein | PF00280: Potato inhibitor I family | GO:0004867 MF: serine-type endopeptidase inhibitor activity;GO:0009611 BP: response to wounding |
|  |  |  |  |  | TraesCS6B01G466400 | SKIP interacting protein 16 | NA | NA |
|  |  |  |  |  | TraesCS6B01G466500 | Ankyrin repeat protein family-like protein | PF13962: Domain of unknown function | GO:0005515 MF: protein binding |
|  |  |  |  |  | TraesCS6B01G466600 | Sucrose synthase | PF00862: Sucrose synthase; PF00534: Glycosyl transferases group 1 | GO:0005985 BP: sucrose metabolic process;GO:0016157 MF: sucrose synthase activity |
|  |  |  |  |  | TraesCS6B01G466700 | Argonaute | PF16486: N-terminal domain of argonaute; PF08699: Argonaute linker 1 domain; PF02170: PAZ domain; PF16488: Argonaute linker 2 domain; PF16487: Mid domain of argonaute; PF02171: Piwi domain | GO:0003676 MF: nucleic acid binding;GO:0005515 MF: protein binding |
|  |  |  |  |  | TraesCS6B01G466800 | NBS-LRR-like resistance protein | PF00931: NB-ARC domain | GO:0043531 MF: ADP binding |
|  |  |  |  |  | TraesCS6B01G466900 | Auxin Efflux Carrier family protein | PF03547: Membrane transport protein | GO:0016021 CC: integral component of membrane;GO:0055085 BP: transmembrane transport |
|  |  |  |  |  | TraesCS6B01G467000 | Homoserine kinase | PF00288: GHMP kinases N terminal domain; PF08544: GHMP kinases C terminal | GO:0004413 MF: homoserine kinase activity;GO:0005524 MF: ATP binding; |
|  |  |  |  |  | TraesCS6B01G467100 | NA | NA | NA |
|  |  |  |  |  | TraesCS6B01G467200 | NA | NA | NA |
|  |  |  |  |  | TraesCS6B01G467300 | Calcium-dependent protein kinase | PF00069: Protein kinase domain; PF13499: EF-hand domain pair | GO:0004672 MF: protein kinase activity;GO:0005509 MF: calcium ion binding; |
|  |  |  |  |  | TraesCS6B01G467400 | Protein sawadee homeodomain-like 2 | PF16719: SAWADEE domain | GO:0003677 MF: DNA binding;GO:0003682 MF: chromatin binding |
| Relative_SFW | AX-158589824 |  | 6D | 3075685 | TraesCS6D01G004100 | F-box family protein | PF00646: F-box domain | GO:0005515 MF: protein binding |
|  |  |  |  |  | TraesCS6D01G004200 | FG-GAP repeat-containing protein | NA | NA |
|  |  |  |  |  | TraesCS6D01G004300 | Anthocyanidin synthase | PF14226: non-haem dioxygenase in morphine synthesis N-terminal; PF03171: 2OG-Fe(II) oxygenase superfamily | GO:0016491 MF: oxidoreductase activity;GO:0055114 BP: oxidation-reduction process |
|  |  |  |  |  | TraesCS6D01G004400 | carboxyl-terminal peptidase, putative (DUF239) | PF14365: Domain of unknown function (DUF4409); PF03080: Domain of unknown function (DUF239) | NA |
|  |  |  |  |  | TraesCS6D01G004500 | Cell surface glycoprotein 1 | NA | NA |
|  |  |  |  |  | TraesCS6D01G004600 | Protein disulfide-isomerase | PF00085: Thioredoxin | GO:0045454 BP: cell redox homeostasis |
|  |  |  |  |  | TraesCS6D01G004700 | Protein disulfide-isomerase | PF00085: Thioredoxin; PF13848: Thioredoxin-like domain | GO:0005783 CC: endoplasmic reticulum;GO:0016853 MF: isomerase activity; |
|  |  |  |  |  | TraesCS6D01G004800 | tetratricopeptide repeat (TPR)-containing protein | PF13181: Tetratricopeptide repeat | GO:0005515 MF: protein binding |
|  |  |  |  |  | TraesCS6D01G004900 | Enhancer of polycomb-like protein | PF10513: Enhancer of polycomb-like | NA |
|  |  |  |  |  | TraesCS6D01G005000 | Ferredoxin-thioredoxin reductase catalytic chain | PF02943: Ferredoxin thioredoxin reductase catalytic beta chain | GO:0016730 MF: oxidoreductase activity, acting on iron-sulfur proteins as donors;GO:0055114 BP: oxidation-reduction process |
|  |  |  |  |  | TraesCS6D01G005100 | RING/U-box superfamily protein | PF12906: RING-variant domain | GO:0008270 MF: zinc ion binding |
|  |  |  |  |  | TraesCS6D01G005200 | Protein disulfide-isomerase | PF00085: Thioredoxin; PF13848: Thioredoxin-like domain | GO:0005783 CC: endoplasmic reticulum;GO:0016853 MF: isomerase activity; |
|  |  |  |  |  | TraesCS6D01G005300 | NBS-LRR-like resistance protein | PF00931: NB-ARC domain | GO:0043531 MF: ADP binding |
|  |  |  |  |  | TraesCS6D01G005400 | transmembrane protein, putative (DUF594) | PF13968: Domain of unknown function (DUF4220); PF04578: Protein of unknown function, DUF594 | NA |
|  |  |  |  |  | TraesCS6D01G005500 | transmembrane protein, putative (DUF594) | PF13968: Domain of unknown function (DUF4220); PF04578: Protein of unknown function, DUF594 | NA |
|  |  |  |  |  | TraesCS6D01G005600 | E3 ubiquitin-protein ligase | PF00632: HECT-domain (ubiquitin-transferase) | GO:0004842 MF: ubiquitin-protein transferase activity;GO:0005488 MF: binding; |
|  |  |  |  |  | TraesCS6D01G005700 | casein kinase I | NA | NA |
|  |  |  |  |  | TraesCS6D01G005800 | Leucine-rich repeat receptor-like protein kinase family protein | PF08263: Leucine rich repeat N-terminal domain; PF13516: Leucine Rich repeat; PF00560: Leucine Rich Repeat; PF13855: Leucine rich repeat | GO:0005515 MF: protein binding |
|  |  |  |  |  | TraesCS6D01G005900 | Leucine-rich repeat receptor-like protein kinase family protein | PF08263: Leucine rich repeat N-terminal domain; PF00560: Leucine Rich Repeat | GO:0005515 MF: protein binding |
|  |  |  |  |  | TraesCS6D01G006000 | Leucine-rich repeat receptor-like protein kinase family protein | PF00560: Leucine Rich Repeat; PF13855: Leucine rich repeat | GO:0005515 MF: protein binding |
|  |  |  |  |  | TraesCS6D01G006100 | F-box protein | NA | GO:0005515 MF: protein binding |
|  |  |  |  |  | TraesCS6D01G006200 | Pollen Ole e 1 allergen/extensin | PF01190: Pollen proteins Ole e I like | NA |
|  |  |  |  |  | TraesCS6D01G006300 | Ubiquitin carboxyl-terminal hydrolase 2 | PF13968: Domain of unknown function (DUF4220); PF04578: Protein of unknown function, DUF594 | NA |
|  |  |  |  |  | TraesCS6D01G006400 | F-box protein | PF00646: F-box domain | GO:0005515 MF: protein binding |
|  |  |  |  |  | TraesCS6D01G006500 | Terpene cyclase/mutase family member | PF13249: Squalene-hopene cyclase N-terminal domain; PF13243: Squalene-hopene cyclase C-terminal domain | GO:0016866 MF: intramolecular transferase activity |
|  |  |  |  |  | TraesCS6D01G006600 | Ankyrin repeat family protein | PF13637: Ankyrin repeats (many copies) | GO:0005515 MF: protein binding |
|  |  |  |  |  | TraesCS6D01G006700 | transmembrane protein, putative (DUF594) | PF13968: Domain of unknown function (DUF4220); PF04578: Protein of unknown function, DUF594 | NA |
|  |  |  |  |  | TraesCS6D01G006900 | Ubiquitin carboxyl-terminal hydrolase 2 | PF13968: Domain of unknown function (DUF4220); PF04578: Protein of unknown function, DUF594 | NA |
|  |  |  |  |  | TraesCS6D01G007000 | Patatin | PF01734: Patatin-like phospholipase | GO:0006629 BP: lipid metabolic process;GO:0008152 BP: metabolic process |
|  |  |  |  |  | TraesCS6D01G007100 | DAG, chloroplastic | NA | NA |
|  |  |  |  |  | TraesCS6D01G007200 | Translocase of chloroplast | PF04548: AIG1 family | GO:0005525 MF: GTP binding |
|  |  |  |  |  | TraesCS6D01G007300 | Protein AIG1 | PF04548: AIG1 family | GO:0005525 MF: GTP binding |
|  |  |  |  |  | TraesCS6D01G007400 | Pathogen-related protein | NA | NA |
|  |  |  |  |  | TraesCS6D01G007500 | Glycosyltransferase | PF00201: UDP-glucoronosyl and UDP-glucosyl transferase | GO:0008152 BP: metabolic process;GO:0016758 MF: transferase activity, transferring hexosyl groups |
|  |  |  |  |  | TraesCS6D01G007600 | Protein ENHANCED DISEASE RESISTANCE 2-like | PF01852: START domain; PF07059: Protein of unknown function (DUF1336) | GO:0008289 MF: lipid binding |
|  |  |  |  |  | TraesCS6D01G007700 | Ubiquitin carboxyl-terminal hydrolase 2 | PF13968: Domain of unknown function (DUF4220); PF04578: Protein of unknown function, DUF594 | NA |
|  |  |  |  |  | TraesCS6D01G007800 | receptor kinase 1 | PF00069: Protein kinase domain | GO:0004672 MF: protein kinase activity;GO:0005524 MF: ATP binding; |
|  |  |  |  |  | TraesCS6D01G007900 | disease resistance protein (TIR-NBS-LRR class) | NA | NA |
|  |  |  |  |  | TraesCS6D01G008000 | T-complex protein 1 subunit theta | PF00118: TCP-1/cpn60 chaperonin family | GO:0005524 MF: ATP binding;GO:0006457 BP: protein folding; |
|  |  |  |  |  | TraesCS6D01G008100 | Flavonoid 3'-hydroxylase | PF00067: Cytochrome P450 | GO:0005506 MF: iron ion binding;GO:0016705 MF: oxidoreductase activity, acting on paired donors, with incorporation or reduction of molecular oxygen; |
|  |  |  |  |  | TraesCS6D01G008200 | O-methyltransferase | PF08100: Dimerisation domain; PF00891: O-methyltransferase | GO:0008168 MF: methyltransferase activity;GO:0008171 MF: O-methyltransferase activity; |
|  |  |  |  |  | TraesCS6D01G008300 | Senescence-associated protein DIN1 | PF00581: Rhodanese-like domain | NA |
|  |  |  |  |  | TraesCS6D01G008400 | Rhodanese-related sulfurtransferase | PF00581: Rhodanese-like domain | NA |
|  |  |  |  |  | TraesCS6D01G008500 | Rhodanese-related sulfurtransferase | PF00581: Rhodanese-like domain | NA |
|  |  |  |  |  | TraesCS6D01G008600 | Rhodanese-related sulfurtransferase | PF00581: Rhodanese-like domain | NA |
|  |  |  |  |  | TraesCS6D01G008700 | nuclear fusion defective 6 | NA | NA |
|  |  |  |  |  | TraesCS6D01G008800 | Red chlorophyll catabolite reductase | PF06405: Red chlorophyll catabolite reductase (RCC reductase) | GO:0051743 MF: red chlorophyll catabolite reductase activity |
|  |  |  |  |  | TraesCS6D01G008900 | GDSL esterase/lipase | PF00657: GDSL-like Lipase/Acylhydrolase | GO:0016788 MF: hydrolase activity, acting on ester bonds |
|  |  |  |  |  | TraesCS6D01G009000 | Red chlorophyll catabolite reductase | PF06405: Red chlorophyll catabolite reductase (RCC reductase) | GO:0051743 MF: red chlorophyll catabolite reductase activity |
|  |  |  |  |  | TraesCS6D01G009100 | 60 kDa chaperonin | PF00118: TCP-1/cpn60 chaperonin family | GO:0005524 MF: ATP binding;GO:0005737 CC: cytoplasm; |
|  |  |  |  |  | TraesCS6D01G009200 | Serine/threonine-protein kinase WNK1 | NA | NA |
|  |  |  |  |  | TraesCS6D01G009300 | Zinc finger (C3HC4-type RING finger) family protein | PF00092: von Willebrand factor type A domain; PF03168: Late embryogenesis abundant protein | NA |
|  |  |  |  |  | TraesCS6D01G009400 | Ankyrin repeat domain-containing protein CP77 | NA | NA |
|  |  |  |  |  | TraesCS6D01G009500 | Bidirectional sugar transporter SWEET | PF03083: Sugar efflux transporter for intercellular exchange | GO:0016021 CC: integral component of membrane |
|  |  |  |  |  | TraesCS6D01G009600 | Bidirectional sugar transporter SWEET | PF03083: Sugar efflux transporter for intercellular exchange | GO:0016021 CC: integral component of membrane |
|  |  |  |  |  | TraesCS6D01G009700 | Bidirectional sugar transporter SWEET | PF03083: Sugar efflux transporter for intercellular exchange | GO:0016021 CC: integral component of membrane |
|  |  |  |  |  | TraesCS6D01G009800 | WAT1-related protein | PF00892: EamA-like transporter family | GO:0016020 CC: membrane;GO:0016021 CC: integral component of membrane |
|  |  |  |  |  | TraesCS6D01G009900 | WAT1-related protein | PF00892: EamA-like transporter family | GO:0016020 CC: membrane;GO:0016021 CC: integral component of membrane |
|  |  |  |  |  | TraesCS6D01G010000 | F-box family protein | PF12937: F-box-like | GO:0005515 MF: protein binding |
|  |  |  |  |  | TraesCS6D01G010100 | Lipoxygenase | PF01477: PLAT/LH2 domain; PF00305: Lipoxygenase | GO:0005515 MF: protein binding;GO:0016491 MF: oxidoreductase activity; |
|  |  |  |  |  | TraesCS6D01G010200 | Urease subunit alpha | NA | NA |
|  |  |  |  |  | TraesCS6D01G010300 | Na(+)-translocating NADH-quinone reductase subunit A | NA | NA |
|  |  |  |  |  | TraesCS6D01G010400 | ROP uanine nucleotide exchange factor 10 | NA | NA |
|  |  |  |  |  | TraesCS6D01G010500 | Histone-lysine N-methyltransferase EZH2 | NA | NA |
|  |  |  |  |  | TraesCS6D01G010600 | F-box family protein | PF00646: F-box domain | GO:0005515 MF: protein binding |
|  |  |  |  |  | TraesCS6D01G010700 | F-box family protein | PF03478: Protein of unknown function (DUF295) | NA |
| Relative_root fresh weighr | AX-158626906 |  | 7B | 704629278 | TraesCS7B01G004400 | Apyrase | PF01150: GDA1/CD39 (nucleoside phosphatase) family | GO:0016787 MF: hydrolase activity |
|  |  |  |  |  | TraesCS7B01G004500 | F-box plant-like protein, putative | PF14476: Petal formation-expressed | NA |
|  |  |  |  |  | TraesCS7B01G004600 | F-box plant-like protein, putative | PF14476: Petal formation-expressed | NA |
|  |  |  |  |  | TraesCS7B01G004700 | Flavin-containing monooxygenase | PF13738: Pyridine nucleotide-disulphide oxidoreductase | GO:0004499 MF: N,N-dimethylaniline monooxygenase activity;GO:0016491 MF: oxidoreductase activity; |
|  |  |  |  |  | TraesCS7B01G004800 | HXXXD-type acyl-transferase-like protein | PF02458: Transferase family | GO:0016747 MF: transferase activity, transferring acyl groups other than amino-acyl groups |
|  |  |  |  |  | TraesCS7B01G004900 | NAC domain protein | PF02365: No apical meristem (NAM) protein | GO:0003677 MF: DNA binding;GO:0006355 BP: regulation of transcription, DNA-templated |
|  |  |  |  |  | TraesCS7B01G005000 | RNA-binding family protein | PF00076: RNA recognition motif. (a.k.a. RRM, RBD, or RNP domain) | GO:0003676 MF: nucleic acid binding |
|  |  |  |  |  | TraesCS7B01G005100 | Anthocyanin 5-aromatic acyltransferase | PF02458: Transferase family | GO:0016747 MF: transferase activity, transferring acyl groups other than amino-acyl groups |
|  |  |  |  |  | TraesCS7B01G005200 | Pleiotropic drug resistance ABC transporter | PF00005: ABC transporter; PF01061: ABC-2 type transporter; PF08370: Plant PDR ABC transporter associated | GO:0005524 MF: ATP binding;GO:0016020 CC: membrane; |
|  |  |  |  |  | TraesCS7B01G005400 | DNA repair radA-like protein | PF13481: AAA domain; PF13541: Subunit ChlI of Mg-chelatase | GO:0003677 MF: DNA binding;GO:0003684 MF: damaged DNA binding; |
|  |  |  |  |  | TraesCS7B01G005500 | ATP-dependent 6-phosphofructokinase | PF00365: Phosphofructokinase | GO:0003872 MF: 6-phosphofructokinase activity;GO:0005524 MF: ATP binding; |
|  |  |  |  |  | TraesCS7B01G005600 | B3 domain-containing protein family | PF02362: B3 DNA binding domain | GO:0003677 MF: DNA binding |
|  |  |  |  |  | TraesCS7B01G005700 | Ubiquitin-like modifier-activating enzyme 5 | PF00899: ThiF family | GO:0008641 MF: small protein activating enzyme activity |
|  |  |  |  |  | TraesCS7B01G005800 | 2,3,4,5-tetrahydropyridine-2,6-dicarboxylate N-acetyltransferase | PF00132: Bacterial transferase hexapeptide (six repeats) | NA |
|  |  |  |  |  | TraesCS7B01G005900 | NBS-LRR disease resistance protein-like | PF00931: NB-ARC domain | GO:0043531 MF: ADP binding |
|  |  |  |  |  | TraesCS7B01G006000 | Dual specificity protein phosphatase family protein | PF00782: Dual specificity phosphatase, catalytic domain | GO:0004725 MF: protein tyrosine phosphatase activity;GO:0006470 BP: protein dephosphorylation; |
|  |  |  |  |  | TraesCS7B01G006100 | 3-ketoacyl-CoA synthase | PF08392: FAE1/Type III polyketide synthase-like protein; PF08541: 3-Oxoacyl-[acyl-carrier-protein (ACP)] synthase III C terminal | GO:0003824 MF: catalytic activity;GO:0006633 BP: fatty acid biosynthetic process; |
|  |  |  |  |  | TraesCS7B01G006200 | 3-ketoacyl-CoA synthase | PF08392: FAE1/Type III polyketide synthase-like protein; PF08541: 3-Oxoacyl-[acyl-carrier-protein (ACP)] synthase III C terminal | GO:0003824 MF: catalytic activity;GO:0006633 BP: fatty acid biosynthetic process;G |
|  |  |  |  |  | TraesCS7B01G006300 | 3-ketoacyl-CoA synthase | PF08392: FAE1/Type III polyketide synthase-like protein; PF02797: Chalcone and stilbene synthases, C-terminal domain | GO:0003824 MF: catalytic activity;GO:0006633 BP: fatty acid biosynthetic process; |
|  |  |  |  |  | TraesCS7B01G006400 | Zinc finger, B-box | PF00643: B-box zinc finger | GO:0005622 CC: intracellular;GO:0008270 MF: zinc ion binding |
|  |  |  |  |  | TraesCS7B01G006500 | Serpin-like protein | PF00079: Serpin (serine protease inhibitor) | NA |
|  |  |  |  |  | TraesCS7B01G006600 | Photosynthetic NDH subcomplex B 3 | PF00111: 2Fe-2S iron-sulfur cluster binding domain | GO:0009055 MF: electron carrier activity;GO:0051536 MF: iron-sulfur cluster binding |
|  |  |  |  |  | TraesCS7B01G006700 | Ubiquinone biosynthesis O-methyltransferase | PF13489: Methyltransferase domain | GO:0006744 BP: ubiquinone biosynthetic process;GO:0008425 MF: 2-polyprenyl-6-methoxy-1,4-benzoquinone methyltransferase activity |
|  |  |  |  |  | TraesCS7B01G006800 | Transcription elongation factor 1 | PF05129: Transcription elongation factor Elf1 like | NA |
|  |  |  |  |  | TraesCS7B01G006900 | Pentatricopeptide repeat-containing protein | PF12854: PPR repeat; PF13041: PPR repeat family; PF01535: PPR repeat | NA |
|  |  |  |  |  | TraesCS7B01G007000 | GDSL esterase/lipase | PF00657: GDSL-like Lipase/Acylhydrolase | GO:0016788 MF: hydrolase activity, acting on ester bonds |
|  |  |  |  |  | TraesCS7B01G007100 | Ribosomal RNA apurinic site specific lyase | PF08387: FBD | NA |
|  |  |  |  |  | TraesCS7B01G007200 | epstein-barr nuclear antigen | NA | NA |
|  |  |  |  |  | TraesCS7B01G007300 | WAT1-related protein | PF00892: EamA-like transporter family | GO:0016020 CC: membrane;GO:0016021 CC: integral component of membrane |
| Relative root length | wsnp_Ku_c8497_14429303 |  | 7B | 64728963 | TraesCS7B01G061100 | Ring finger protein, putative | PF13639: Ring finger domain | GO:0005515 MF: protein binding;GO:0008270 MF: zinc ion binding |
|  |  |  |  |  | TraesCS7B01G061200 | Haloacid dehalogenase-like hydrolase superfamily protein | PF06941: 5' nucleotidase, deoxy (Pyrimidine), cytosolic type C protein (NT5C) | GO:0008253 MF: 5'-nucleotidase activity;GO:0009264 BP: deoxyribonucleotide catabolic process |
|  |  |  |  |  | TraesCS7B01G061300 | Histone H3 | PF00125: Core histone H2A/H2B/H3/H4 | GO:0000786 CC: nucleosome;GO:0003677 MF: DNA binding; |
|  |  |  |  |  | TraesCS7B01G061400 | Exocyst complex component, putative | PF03081: Exo70 exocyst complex subunit | GO:0000145 CC: exocyst;GO:0006887 BP: exocytosis |
|  |  |  |  |  | TraesCS7B01G061500 | Ubiquitin-conjugating enzyme, E2 | PF00179: Ubiquitin-conjugating enzyme | NA |
|  |  |  |  |  | TraesCS7B01G061600 | Pectinesterase | PF04043: Plant invertase/pectin methylesterase inhibitor; PF01095: Pectinesterase | GO:0004857 MF: enzyme inhibitor activity;GO:0005618 CC: cell wall; |
|  |  |  |  |  | TraesCS7B01G061700 | calcium-dependent lipid-binding family protein | NA | NA |
|  |  |  |  |  | TraesCS7B01G061800 | Beta-1,3-N-Acetylglucosaminyltransferase family protein, putative | NA | NA |
|  |  |  |  |  | TraesCS7B01G061900 | Receptor-like kinase | PF14380: Wall-associated receptor kinase C-terminal; PF00069: Protein kinase domain | GO:0004672 MF: protein kinase activity;GO:0005524 MF: ATP binding; |
|  |  |  |  |  | TraesCS7B01G062100 | Basic helix-loop-helix transcription factor | PF00010: Helix-loop-helix DNA-binding domain | GO:0046983 MF: protein dimerization activity |
|  |  |  |  |  | TraesCS7B01G062200 | Ethylene-responsive transcription factor | PF00847: AP2 domain | GO:0003677 MF: DNA binding;GO:0003700 MF: transcription factor activity, sequence-specific DNA binding; |
| Relative _SL | AX-158544315 |  | 7D | 87479166 | TraesCS7D01G135100 | Sulfiredoxin | NA | NA |
|  |  |  |  |  | TraesCS7D01G135200 | MYB-related transcription factor | PF00249: Myb-like DNA-binding domain | GO:0003677 MF: DNA binding |
|  |  |  |  |  | TraesCS7D01G135300 | MYB-related transcription factor | PF00249: Myb-like DNA-binding domain | GO:0003677 MF: DNA binding |
|  |  |  |  |  | TraesCS7D01G135400 | Sulfiredoxin | NA | NA |
|  |  |  |  |  | TraesCS7D01G135500 | MYB-related transcription factor | PF00249: Myb-like DNA-binding domain | GO:0003677 MF: DNA binding |
|  |  |  |  |  | TraesCS7D01G135600 | MYB-related transcription factor | PF00249: Myb-like DNA-binding domain | GO:0003677 MF: DNA binding |
|  |  |  |  |  | TraesCS7D01G135700 | MYB-related transcription factor | PF00249: Myb-like DNA-binding domain | GO:0003677 MF: DNA binding |
|  |  |  |  |  | TraesCS7D01G135800 | Sulfiredoxin | PF02195: ParB-like nuclease domain | NA |
|  |  |  |  |  | TraesCS7D01G135900 | DCD (Development and Cell Death) domain-like protein | PF10539: Development and cell death domain | NA |
|  |  |  |  |  | TraesCS7D01G136200 | DCD (Development and cell death) domain protein | PF10539: Development and cell death domain | NA |
|  |  |  |  |  | TraesCS7D01G136300 | Vesicle-associated membrane protein | PF13774: Regulated-SNARE-like domain; PF00957: Synaptobrevin | GO:0006810 BP: transport;GO:0016021 CC: integral component of membrane; |
|  |  |  |  |  | TraesCS7D01G136400 | Cytochrome P450 | PF00067: Cytochrome P450 | GO:0005506 MF: iron ion binding;GO:0016705 MF: oxidoreductase activity, acting on paired donors, with incorporation or reduction of molecular oxygen; |
|  |  |  |  |  | TraesCS7D01G136500 | COBRA-like protein | PF04833: COBRA-like protein | GO:0010215 BP: cellulose microfibril organization;GO:0016049 BP: cell growth; |
|  |  |  |  |  | TraesCS7D01G136600 | Cytochrome b6-f complex subunit 6 | PF05115: Cytochrome B6-F complex subunit VI (PetL) | GO:0009055 MF: electron carrier activity;GO:0009512 CC: cytochrome b6f complex |
|  |  |  |  |  | TraesCS7D01G136700 | Protein kinase family protein | PF13947: Wall-associated receptor kinase galacturonan-binding; PF07645: Calcium-binding EGF domain; PF00069: Protein kinase domain | GO:0004672 MF: protein kinase activity;GO:0005509 MF: calcium ion binding; |
|  |  |  |  |  | TraesCS7D01G136800 | Mitochondrial ATP synthase 6 kDa subunit | NA | NA |
|  |  |  |  |  | TraesCS7D01G137100 | 3'-N-debenzoyl-2'-deoxytaxol N-benzoyltransferase | PF02458: Transferase family | GO:0016747 MF: transferase activity, transferring acyl groups other than amino-acyl groups |
|  |  |  |  |  | TraesCS7D01G137200 | Pheophorbide a oxygenase, chloroplastic | PF00355: Rieske [2Fe-2S] domain; PF08417: Pheophorbide a oxygenase | GO:0010277 MF: chlorophyllide a oxygenase [overall] activity;GO:0016491 MF: oxidoreductase activity; |
|  |  |  |  |  | TraesCS7D01G137300 | Zinc knuckle family protein, expressed | NA | NA |
|  |  |  |  |  | TraesCS7D01G137500 | Cysteine-rich PDZ-binding protein | PF10235: Microtubule-associated protein CRIPT | NA |
|  |  |  |  |  | TraesCS7D01G137600 | NA | NA | NA |
|  |  |  |  |  | TraesCS7D01G137700 | RNA 3'-terminal phosphate cyclase | NA | NA |
|  |  |  |  |  | TraesCS7D01G137900 | Zinc knuckle family protein, expressed | NA | GO:0003676 MF: nucleic acid binding;GO:0008270 MF: zinc ion binding |

**Table S3: List of significant Haplotypes/SNPs for STI, and corresponding chromosomal position and their linked candidate genes in 1Mb span of up and down stream regions**

| **Trait** | **Marker** | **Haplotype** | **Chromosome** | **Position** | **Gene** | **Description** | **Protein family** | **Description** | **GO (Gene ontology)** |
| --- | --- | --- | --- | --- | --- | --- | --- | --- | --- |
| STI_SL | AX-158595571 | sti_SL_1A_Hap1 | 1A | 535318093 | TraesCS1A01G350400 | Heat shock transcription factor | PF00447: HSF-type DNA-binding | IPR000232: Heat shock factor (HSF)-type, DNA-binding; IPR011991: Winged helix-turn-helix DNA-binding domain | GO:0003700 MF: transcription factor activity, sequence-specific DNA binding;GO:0005634 CC: nucleus; |
|  |  |  |  |  | TraesCS1A01G350500 | Non-specific serine/threonine protein kinase | PF00069: Protein kinase domain; PF02149: Kinase associated domain 1 | IPR000719: Protein kinase domain; IPR001772: Kinase associated domain 1 (KA1); IPR008271: Serine/threonine-protein kinase, active site; IPR011009: Protein kinase-like domain; IPR015940: Ubiquitin-associated domain; IPR017441: Protein kinase, ATP binding site; IPR028375: KA1 domain/Ssp2, C-terminal | GO:0004672 MF: protein kinase activity;GO:0005524 MF: ATP binding; |
| STI_SFW | Excalibur_c54055_694 |  | 1D | 308455842 | TraesCS1D01G219800 | bZIP transcription factor (DUF630 and DUF632) | PF04783: Protein of unknown function (DUF630); PF04782: Protein of unknown function (DUF632) | IPR006867: Domain of unknown function DUF632; IPR006868: Domain of unknown function DUF630 | NA |
|  |  |  |  |  | TraesCS1D01G219900 | Protein BRANCHLESS TRICHOME | NA | NA | NA |
|  |  |  |  |  | TraesCS1D01G220000 | Zinc import ATP-binding protein ZnuC | PF04068: Possible Fer4-like domain in RNase L inhibitor, RLI; PF00037: 4Fe-4S binding domain; PF00005: ABC transporter | IPR003439: ABC transporter-like; IPR003593: AAA+ ATPase domain; IPR007209: RNase L inhibitor RLI, possible metal-binding domain; IPR013283: RLI1; IPR017871: ABC transporter, conserved site; IPR017896: 4Fe-4S ferredoxin-type, iron-sulphur binding domain; IPR017900: 4Fe-4S ferredoxin, iron-sulphur binding, conserved site; IPR027417: P-loop containing nucleoside triphosphate hydrolase | GO:0005524 MF: ATP binding;GO:0016887 MF: ATPase activity |
|  |  |  |  |  | TraesCS1D01G220100 | Kinase family protein | PF00069: Protein kinase domain | IPR000719: Protein kinase domain; IPR008271: Serine/threonine-protein kinase, active site; IPR011009: Protein kinase-like domain | GO:0004672 MF: protein kinase activity;GO:0005524 MF: ATP binding; |
|  |  |  |  |  | TraesCS1D01G220200 | Serine/threonine protein phosphatase 7 long form isogeny | PF10536: Plant mobile domain | IPR019557: Aminotransferase-like, plant mobile domain | NA |
|  |  |  |  |  | TraesCS1D01G220300 | Phospholipase a1-chloroplastic-like | PF01764: Lipase (class 3) | IPR002921: Fungal lipase-like domain; IPR029058: Alpha/Beta hydrolase fold | GO:0006629 BP: lipid metabolic process |
|  |  |  |  |  | TraesCS1D01G220400 | Serine hydroxymethyltransferase | PF00464: Serine hydroxymethyltransferase | IPR001085: Serine hydroxymethyltransferase; IPR015421: Pyridoxal phosphate-dependent transferase, major region, subdomain 1; IPR015422: Pyridoxal phosphate-dependent transferase, subdomain 2; IPR015424: Pyridoxal phosphate-dependent transferase; IPR019798: Serine hydroxymethyltransferase, pyridoxal phosphate binding site | GO:0003824 MF: catalytic activity;GO:0004372 MF: glycine hydroxymethyltransferase activity;binding |
|  |  |  |  |  | TraesCS1D01G220500 | MYB-related transcription factor | PF00249: Myb-like DNA-binding domain | IPR001005: SANT/Myb domain; IPR006447: Myb domain, plants; IPR009057: Homeobox domain-like; IPR017930: Myb domain | GO:0003677 MF: DNA binding |
|  |  |  |  |  | TraesCS1D01G220600 | Dual specificity protein phosphatase | PF00782: Dual specificity phosphatase, catalytic domain | IPR000340: Dual specificity phosphatase, catalytic domain; IPR000387: Tyrosine specific protein phosphatases domain; IPR016130: Protein-tyrosine phosphatase, active site; IPR020422: Dual specificity protein phosphatase domain; IPR029021: Protein-tyrosine phosphatase-like | GO:0004725 MF: protein tyrosine phosphatase activity;GO:0006470 BP: protein dephosphorylation; |
|  |  |  |  |  | TraesCS1D01G220700 | Cotton fiber protein | PF05553: Cotton fibre expressed protein | IPR008480: Protein of unknown function DUF761, plant | NA |
|  |  |  |  |  | TraesCS1D01G220800 | NA | NA | NA | NA |
|  |  |  |  |  | TraesCS1D01G220900 | Homeobox-leucine zipper family protein | PF04618: HD-ZIP protein N terminus; PF00046: Homeobox domain; PF02183: Homeobox associated leucine zipper | IPR000047: Helix-turn-helix motif; IPR001356: Homeobox domain; IPR003106: Leucine zipper, homeobox-associated; IPR006712: HD-ZIP protein, N-terminal; IPR009057: Homeobox domain-like; IPR017970: Homeobox, conserved site | GO:0003677 MF: DNA binding;GO:0003700 MF: transcription factor activity, sequence-specific DNA binding; |
|  |  |  |  |  | TraesCS1D01G221000 | Myb transcription factor | PF00249: Myb-like DNA-binding domain | IPR001005: SANT/Myb domain; IPR006447: Myb domain, plants; IPR009057: Homeobox domain-like; IPR017930: Myb domain | GO:0003677 MF: DNA binding |
|  |  |  |  |  | TraesCS1D01G221100 | Sugar transporter, putative | PF00083: Sugar (and other) transporter | IPR003663: Sugar/inositol transporter; IPR005828: Major facilitator, sugar transporter-like; IPR005829: Sugar transporter, conserved site; IPR020846: Major facilitator superfamily domain | GO:0005215 MF: transporter activity;GO:0016020 CC: membrane; |
|  |  |  |  |  | TraesCS1D01G221200 | Transcription elongation factor SPT5 | NA | NA | NA |
|  |  |  |  |  | TraesCS1D01G221300 | 8-amino-7-oxononanoate synthase | PF00155: Aminotransferase class I and II | IPR004839: Aminotransferase, class I/classII; IPR015421: Pyridoxal phosphate-dependent transferase, major region, subdomain 1; IPR015422: Pyridoxal phosphate-dependent transferase, subdomain 2; IPR015424: Pyridoxal phosphate-dependent transferase | GO:0003824 MF: catalytic activity;GO:0009058 BP: biosynthetic process; |
|  |  |  |  |  | TraesCS1D01G221400 | Leucine--tRNA ligase | PF00133: tRNA synthetases class I (I, L, M and V); PF08264: Anticodon-binding domain of tRNA | IPR001412: Aminoacyl-tRNA synthetase, class I, conserved site; IPR002300: Aminoacyl-tRNA synthetase, class Ia; IPR004493: Leucyl-tRNA synthetase, class Ia, archaeal/eukaryotic cytosolic; IPR009008: Valyl/Leucyl/Isoleucyl-tRNA synthetase, editing domain; IPR009080: Aminoacyl-tRNA synthetase, class Ia, anticodon-binding; IPR013155: Methionyl/Valyl/Leucyl/Isoleucyl-tRNA synthetase, anticodon-binding; IPR014729: Rossmann-like alpha/beta/alpha sandwich fold | GO:0000166 MF: nucleotide binding;GO:0002161 MF: aminoacyl-tRNA editing activity; |
|  |  |  |  |  | TraesCS1D01G221500 | ethylene-responsive transcription factor | PF00847: AP2 domain | IPR001471: AP2/ERF domain; IPR016177: DNA-binding domain | GO:0003677 MF: DNA binding;GO:0003700 MF: transcription factor activity, sequence-specific DNA binding; |
| STI_RL | AX-158596313 |  | 2A | 421064707 | TraesCS2A01G264800 | Two-component response regulator | PF00249: Myb-like DNA-binding domain | IPR001005: SANT/Myb domain; IPR006447: Myb domain, plants; IPR009057: Homeobox domain-like; IPR017930: Myb domain | GO:0003677 MF: DNA binding |
|  |  |  |  |  | TraesCS2A01G264900 | L-ascorbate oxidase-like protein | PF07732: Multicopper oxidase; PF00394: Multicopper oxidase; PF07731: Multicopper oxidase | IPR001117: Multicopper oxidase, type 1; IPR008972: Cupredoxin; IPR011706: Multicopper oxidase, type 2; IPR011707: Multicopper oxidase, type 3 | GO:0005507 MF: copper ion binding;GO:0016491 MF: oxidoreductase activity; |
|  |  |  |  |  | TraesCS2A01G265000 | N-succinylglutamate 5-semialdehyde dehydrogenase | NA | NA | NA |
|  |  |  |  |  | TraesCS2A01G265100 | Golgin | PF14695: Lines C-terminus | IPR029415: Protein Lines, C-terminal | NA |
|  |  |  |  |  | TraesCS2A01G265200 | B3 domain-containing protein | NA | IPR015300: DNA-binding pseudobarrel domain | NA |
|  |  |  |  |  | TraesCS2A01G265300 | F-box family protein-like protein | PF00646: F-box domain | IPR001810: F-box domain | GO:0005515 MF: protein binding |
|  |  |  |  |  | TraesCS2A01G265400 | F-box domain containing protein | NA | IPR011042: Six-bladed beta-propeller, TolB-like; IPR011047: Quinoprotein alcohol dehydrogenase-like superfamily | NA |
|  |  |  |  |  | TraesCS2A01G265500 | Disease resistance protein (TIR-NBS-LRR class) family | PF13456: Reverse transcriptase-like | IPR012337: Ribonuclease H-like domain | GO:0003676 MF: nucleic acid binding |
|  |  |  |  |  | TraesCS2A01G265600 | F-box family protein | PF00646: F-box domain | IPR001810: F-box domain; IPR011047: Quinoprotein alcohol dehydrogenase-like superfamily; IPR015943: WD40/YVTN repeat-like-containing domain | GO:0005515 MF: protein binding |
| STI_RL | AX-111016876 |  | 2A | 717417914 | TraesCS2A01G477000 | Pathogenesis-related protein 1 | PF00407: Pathogenesis-related protein Bet v I family | IPR000916: Bet v I/Major latex protein; IPR023393: START-like domain; IPR024949: Bet v I type allergen | GO:0006952 BP: defense response;GO:0009607 BP: response to biotic stimulus |
|  |  |  |  |  | TraesCS2A01G477100 | NA | NA | NA | NA |
|  |  |  |  |  | TraesCS2A01G477200 | NBS-LRR disease resistance protein | PF00931: NB-ARC domain | IPR002182: NB-ARC; IPR011991: Winged helix-turn-helix DNA-binding domain; IPR027417: P-loop containing nucleoside triphosphate hydrolase; IPR032675: Leucine-rich repeat domain, L domain-like | GO:0043531 MF: ADP binding |
|  |  |  |  |  | TraesCS2A01G477300 | NA | NA | NA | NA |
|  |  |  |  |  | TraesCS2A01G477400 | GDSL esterase/lipase | PF00657: GDSL-like Lipase/Acylhydrolase | IPR001087: GDSL lipase/esterase; IPR013830: SGNH hydrolase-type esterase domain | GO:0016788 MF: hydrolase activity, acting on ester bonds |
|  |  |  |  |  | TraesCS2A01G477500 | Pentatricopeptide repeat-containing protein | PF01535: PPR repeat; PF13041: PPR repeat family | IPR002885: Pentatricopeptide repeat; IPR011990: Tetratricopeptide-like helical domain | GO:0005515 MF: protein binding |
|  |  |  |  |  | TraesCS2A01G477600 | Non-specific lipid-transfer protein | PF00234: Protease inhibitor/seed storage/LTP family | IPR000528: Plant lipid transfer protein/Par allergen; IPR016140: Bifunctional inhibitor/plant lipid transfer protein/seed storage helical domain | GO:0006869 BP: lipid transport;GO:0008289 MF: lipid binding |
|  |  |  |  |  | TraesCS2A01G477700 | Non-specific lipid-transfer protein | PF00234: Protease inhibitor/seed storage/LTP family | IPR000528: Plant lipid transfer protein/Par allergen; IPR016140: Bifunctional inhibitor/plant lipid transfer protein/seed storage helical domain | GO:0006869 BP: lipid transport;GO:0008289 MF: lipid binding |
|  |  |  |  |  | TraesCS2A01G477800 | NA | NA | NA | NA |
|  |  |  |  |  | TraesCS2A01G477900 | Aldehyde dehydrogenase | PF00171: Aldehyde dehydrogenase family | IPR010061: Methylmalonate-semialdehyde dehydrogenase; IPR015590: Aldehyde dehydrogenase domain; IPR016160: Aldehyde dehydrogenase, cysteine active site; IPR016161: Aldehyde/histidinol dehydrogenase; IPR016162: Aldehyde dehydrogenase N-terminal domain | GO:0004491 MF: methylmalonate-semialdehyde dehydrogenase (acylating) activity;GO:0008152 BP: metabolic process; |
|  |  |  |  |  | TraesCS2A01G478000 | Histone-lysine N-methyltransferase | PF10440: Ubiquitin-binding WIYLD domain; PF05033: Pre-SET motif; PF00856: SET domain | IPR001214: SET domain; IPR007728: Pre-SET domain; IPR018848: WIYLD domain; IPR025776: Histone-lysine N-methyltransferase SUVR4/SUVR1/SUVR2 | GO:0005515 MF: protein binding;GO:0005634 CC: nucleus; |
|  |  |  |  |  | TraesCS2A01G478100 | Linalool synthase, chloroplastic | PF01397: Terpene synthase, N-terminal domain; PF03936: Terpene synthase family, metal binding domain | IPR001906: Terpene synthase, N-terminal domain; IPR005630: Terpene synthase, metal-binding domain; IPR008930: Terpenoid cyclases/protein prenyltransferase alpha-alpha toroid; IPR008949: Isoprenoid synthase domain | GO:0000287 MF: magnesium ion binding;GO:0008152 BP: metabolic process; |
|  |  |  |  |  | TraesCS2A01G478200 | NA | NA | NA | NA |
|  |  |  |  |  | TraesCS2A01G478300 | UHRF1-binding protein 1 | PF12624: N-terminal region of Chorein or VPS13 | IPR026854: Vacuolar protein sorting-associated protein 13, N-terminal domain | NA |
|  |  |  |  |  | TraesCS2A01G478400 | UDP-N-acetylmuramate--L-alanine ligase | NA | NA | NA |
|  |  |  |  |  | TraesCS2A01G478500 | Beta-glucosidase | PF00933: Glycosyl hydrolase family 3 N terminal domain; PF01915: Glycosyl hydrolase family 3 C-terminal domain | IPR001764: Glycoside hydrolase, family 3, N-terminal; IPR002772: Glycoside hydrolase family 3 C-terminal domain; IPR017853: Glycoside hydrolase superfamily; IPR019800: Glycoside hydrolase, family 3, active site | GO:0004553 MF: hydrolase activity, hydrolyzing O-glycosyl compounds;GO:0005975 BP: carbohydrate metabolic process |
|  |  |  |  |  | TraesCS2A01G478600 | Sugar transport protein 5 | NA | NA | NA |
|  |  |  |  |  | TraesCS2A01G478700 | Glycosyltransferase | PF00201: UDP-glucoronosyl and UDP-glucosyl transferase | IPR002213: UDP-glucuronosyl/UDP-glucosyltransferase | GO:0008152 BP: metabolic process;GO:0016758 MF: transferase activity, transferring hexosyl groups |
|  |  |  |  |  | TraesCS2A01G478800 | Histone-lysine N-methyltransferase | PF10440: Ubiquitin-binding WIYLD domain; PF05033: Pre-SET motif; PF00856: SET domain | IPR001214: SET domain; IPR007728: Pre-SET domain; IPR018848: WIYLD domain; IPR025776: Histone-lysine N-methyltransferase SUVR4/SUVR1/SUVR2 | GO:0005515 MF: protein binding;GO:0005634 CC: nucleus; |
|  |  |  |  |  | TraesCS2A01G478900 | Glycosyltransferase | PF00201: UDP-glucoronosyl and UDP-glucosyl transferase | IPR002213: UDP-glucuronosyl/UDP-glucosyltransferase | GO:0008152 BP: metabolic process;GO:0016758 MF: transferase activity, transferring hexosyl groups |
|  |  |  |  |  | TraesCS2A01G479000 | Phosphatidylinositol-4-phosphate 5-kinase, putative | PF02493: MORN repeat; PF01504: Phosphatidylinositol-4-phosphate 5-Kinase | IPR002498: Phosphatidylinositol-4-phosphate 5-kinase, core; IPR003409: MORN motif; IPR017163: Phosphatidylinositol-4-phosphate 5-kinase, plant; IPR027483: Phosphatidylinositol-4-phosphate 5-kinase, C-terminal; IPR027484: Phosphatidylinositol-4-phosphate 5-kinase, N-terminal domain | GO:0005524 MF: ATP binding;GO:0016307 MF: phosphatidylinositol phosphate kinase activity; |
|  |  |  |  |  | TraesCS2A01G479100 | Cyclin family protein | PF08613: Cyclin | IPR013763: Cyclin-like; IPR013922: Cyclin PHO80-like | GO:0000079 BP: regulation of cyclin-dependent protein serine/threonine kinase activity;GO:0019901 MF: protein kinase binding |
|  |  |  |  |  | TraesCS2A01G479200 | F-box family protein | NA | NA | NA |
|  |  |  |  |  | TraesCS2A01G479300 | ATP/DNA-binding protein | NA | IPR003594: Histidine kinase-like ATPase, C-terminal domain | NA |
|  |  |  |  |  | TraesCS2A01G479400 | Peroxisome biogenesis protein 22 | NA | NA | NA |
|  |  |  |  |  | TraesCS2A01G479500 | Histone-lysine N-methyltransferase | PF00856: SET domain; PF01753: MYND finger | IPR001214: SET domain; IPR002893: Zinc finger, MYND-type | GO:0005515 MF: protein binding |
|  |  |  |  |  | TraesCS2A01G479600 | CsAtPR5 | NA | NA | NA |
|  |  |  |  |  | TraesCS2A01G479700 | Invertase/pectin methylesterase inhibitor family protein | PF04043: Plant invertase/pectin methylesterase inhibitor | IPR006501: Pectinesterase inhibitor domain | GO:0004857 MF: enzyme inhibitor activity |
|  |  |  |  |  | TraesCS2A01G479800 | CsAtPR5, putative, expressed | NA | NA | NA |
|  |  |  |  |  | TraesCS2A01G479900 | SNF2 domain-containing protein / helicase domain-containing protein / zinc finger protein-like protein | PF00176: SNF2 family N-terminal domain; PF14634: zinc-RING finger domain; PF00271: Helicase conserved C-terminal domain | IPR000330: SNF2-related, N-terminal domain; IPR001650: Helicase, C-terminal; IPR001841: Zinc finger, RING-type; IPR013083: Zinc finger, RING/FYVE/PHD-type; IPR014001: Helicase superfamily 1/2, ATP-binding domain; IPR017907: Zinc finger, RING-type, conserved site; IPR027417: P-loop containing nucleoside triphosphate hydrolase | GO:0005515 MF: protein binding;GO:0005524 MF: ATP binding;GO:0008270 MF: zinc ion binding |
|  |  |  |  |  | TraesCS2A01G480000 | Phosphatidylinositol 3- and 4-kinase family protein | NA | NA | NA |
|  |  |  |  |  | TraesCS2A01G480100 | NBS-LRR disease resistance protein, putative, expressed | PF00931: NB-ARC domain | IPR002182: NB-ARC; IPR011991: Winged helix-turn-helix DNA-binding domain; IPR032675: Leucine-rich repeat domain, L domain-like | GO:0043531 MF: ADP binding |
|  |  |  |  |  | TraesCS2A01G480200 | Divalent-cation tolerance protein CutA | PF03091: CutA1 divalent ion tolerance protein | IPR004323: Divalent ion tolerance protein, CutA; IPR011322: Nitrogen regulatory PII-like, alpha/beta; IPR015867: Nitrogen regulatory protein PII/ATP phosphoribosyltransferase, C-terminal | GO:0010038 BP: response to metal ion |
|  |  |  |  |  | TraesCS2A01G480300 | Thioredoxin | PF00085: Thioredoxin | IPR012336: Thioredoxin-like fold; IPR013766: Thioredoxin domain | GO:0045454 BP: cell redox homeostasis |
|  |  |  |  |  | TraesCS2A01G480400 | NBS-LRR disease resistance protein | PF00931: NB-ARC domain | IPR002182: NB-ARC; IPR011991: Winged helix-turn-helix DNA-binding domain; IPR027417: P-loop containing nucleoside triphosphate hydrolase; IPR032675: Leucine-rich repeat domain, L domain-like | GO:0043531 MF: ADP binding |
|  |  |  |  |  | TraesCS2A01G480500 | Kinesin-like protein | PF00225: Kinesin motor domain | IPR001752: Kinesin motor domain; IPR019821: Kinesin motor domain, conserved site; IPR027417: P-loop containing nucleoside triphosphate hydrolase | GO:0003777 MF: microtubule motor activity;GO:0005524 MF: ATP binding;GO:0007018 BP: microtubule-based movement;GO:0008017 MF: microtubule binding |
|  |  |  |  |  | TraesCS2A01G480600 | Kinase-like | PF00069: Protein kinase domain | IPR000719: Protein kinase domain; IPR008271: Serine/threonine-protein kinase, active site; IPR011009: Protein kinase-like domain; IPR017441: Protein kinase, ATP binding site | GO:0004672 MF: protein kinase activity;GO:0005524 MF: ATP binding; |
|  |  |  |  |  | TraesCS2A01G480700 | Sodium- and chloride-dependent creatine transporter 1 | NA | NA | NA |
|  |  |  |  |  | TraesCS2A01G480800 | Serine/threonine-protein kinase | NA | NA | NA |
|  |  |  |  |  | TraesCS2A01G480900 | Glycosyltransferase | PF04577: Protein of unknown function (DUF563) | IPR007657: Glycosyltransferase 61 | GO:0016757 MF: transferase activity, transferring glycosyl groups |
| STI_RL | AX-108742509 |  | 2A | 718354502 | TraesCS2A01G480100 | NBS-LRR disease resistance protein, putative, expressed | PF00931: NB-ARC domain | IPR002182: NB-ARC; IPR011991: Winged helix-turn-helix DNA-binding domain; IPR032675: Leucine-rich repeat domain, L domain-like | GO:0043531 MF: ADP binding |
|  |  |  |  |  | TraesCS2A01G480200 | Divalent-cation tolerance protein CutA | PF03091: CutA1 divalent ion tolerance protein | IPR004323: Divalent ion tolerance protein, CutA; IPR011322: Nitrogen regulatory PII-like, alpha/beta; IPR015867: Nitrogen regulatory protein PII/ATP phosphoribosyltransferase, C-terminal | GO:0010038 BP: response to metal ion |
|  |  |  |  |  | TraesCS2A01G480300 | Thioredoxin | PF00085: Thioredoxin | IPR012336: Thioredoxin-like fold; IPR013766: Thioredoxin domain | GO:0045454 BP: cell redox homeostasis |
|  |  |  |  |  | TraesCS2A01G480400 | NBS-LRR disease resistance protein | PF00931: NB-ARC domain | IPR002182: NB-ARC; IPR011991: Winged helix-turn-helix DNA-binding domain; IPR027417: P-loop containing nucleoside triphosphate hydrolase; IPR032675: Leucine-rich repeat domain, L domain-like | GO:0043531 MF: ADP binding |
|  |  |  |  |  | TraesCS2A01G480500 | Kinesin-like protein | PF00225: Kinesin motor domain | IPR001752: Kinesin motor domain; IPR019821: Kinesin motor domain, conserved site; IPR027417: P-loop containing nucleoside triphosphate hydrolase | GO:0003777 MF: microtubule motor activity;GO:0005524 MF: ATP binding; |
|  |  |  |  |  | TraesCS2A01G480600 | Kinase-like | PF00069: Protein kinase domain | IPR000719: Protein kinase domain; IPR008271: Serine/threonine-protein kinase, active site; IPR011009: Protein kinase-like domain; IPR017441: Protein kinase, ATP binding site | GO:0004672 MF: protein kinase activity;GO:0005524 MF: ATP binding; |
|  |  |  |  |  | TraesCS2A01G480700 | Sodium- and chloride-dependent creatine transporter 1 | NA | NA | NA |
| STI_RSRatio | RAC875_c21906_247 |  | 2A | 691478630 | TraesCS2A01G439200 | Acylphosphatase | NA | NA | NA |
|  |  |  |  |  | TraesCS2A01G439300 | Protein kinase superfamily protein | NA | IPR011009: Protein kinase-like domain | NA |
|  |  |  |  |  | TraesCS2A01G439400 | Ubiquinol oxidase | PF01786: Alternative oxidase | IPR002680: Alternative oxidase | GO:0009916 MF: alternative oxidase activity;GO:0055114 BP: oxidation-reduction process |
|  |  |  |  |  | TraesCS2A01G439500 | Glucan endo-1,3-beta-glucosidase | PF00332: Glycosyl hydrolases family 17; PF07983: X8 domain | IPR000490: Glycoside hydrolase family 17; IPR012946: X8 domain; IPR017853: Glycoside hydrolase superfamily | GO:0004553 MF: hydrolase activity, hydrolyzing O-glycosyl compounds;GO:0005975 BP: carbohydrate metabolic process |
|  |  |  |  |  | TraesCS2A01G439600 | Pathogenesis-related protein 1 | PF00188: Cysteine-rich secretory protein family | IPR001283: Cysteine-rich secretory protein, allergen V5/Tpx-1-related; IPR014044: CAP domain | NA |
|  |  |  |  |  | TraesCS2A01G439700 | Pathogenesis-related protein 1 | PF00188: Cysteine-rich secretory protein family | IPR001283: Cysteine-rich secretory protein, allergen V5/Tpx-1-related; IPR014044: CAP domain | NA |
|  |  |  |  |  | TraesCS2A01G439800 | Cytochrome P450 | PF00067: Cytochrome P450 | IPR001128: Cytochrome P450; IPR002401: Cytochrome P450, E-class, group I; IPR017972: Cytochrome P450, conserved site | GO:0005506 MF: iron ion binding;GO:0016705 MF: oxidoreductase activity, acting on paired donors, with incorporation or reduction of molecular oxygen; |
|  |  |  |  |  | TraesCS2A01G439900 | CobW-domain-containing protein | PF02492: CobW/HypB/UreG, nucleotide-binding domain; PF07683: Cobalamin synthesis protein cobW C-terminal domain | IPR003495: CobW/HypB/UreG, nucleotide-binding domain; IPR011629: Cobalamin (vitamin B12) biosynthesis CobW-like, C-terminal; IPR027417: P-loop containing nucleoside triphosphate hydrolase | NA |
|  |  |  |  |  | TraesCS2A01G440000 | WD-repeat cell cycle regulatory protein | PF12894: Anaphase-promoting complex subunit 4 WD40 domain; PF00400: WD domain, G-beta repeat | IPR001680: WD40 repeat; IPR015943: WD40/YVTN repeat-like-containing domain; IPR017986: WD40-repeat-containing domain; IPR019775: WD40 repeat, conserved site; IPR024977: Anaphase-promoting complex subunit 4, WD40 domain | GO:0005515 MF: protein binding |
|  |  |  |  |  | TraesCS2A01G440100 | Clathrin interactor EPSIN 1 | PF01417: ENTH domain | IPR008942: ENTH/VHS; IPR013809: ENTH domain | NA |
|  |  |  |  |  | TraesCS2A01G440200 | NA | NA | NA | NA |
|  |  |  |  |  | TraesCS2A01G440300 | Bifunctional inhibitor/lipid-transfer protein/seed storage 2S albumin-like protein | PF14368: Probable lipid transfer | IPR016140: Bifunctional inhibitor/plant lipid transfer protein/seed storage helical domain | NA |
|  |  |  |  |  | TraesCS2A01G440400 | Lipid transfer protein | PF14368: Probable lipid transfer | IPR016140: Bifunctional inhibitor/plant lipid transfer protein/seed storage helical domain | NA |
|  |  |  |  |  | TraesCS2A01G440500 | Protease inhibitor/seed storage/lipid transfer protein family protein | PF14368: Probable lipid transfer | IPR016140: Bifunctional inhibitor/plant lipid transfer protein/seed storage helical domain | NA |
|  |  |  |  |  | TraesCS2A01G440600 | Protease inhibitor/seed storage/lipid transfer protein family protein | PF14368: Probable lipid transfer | IPR016140: Bifunctional inhibitor/plant lipid transfer protein/seed storage helical domain | NA |
|  |  |  |  |  | TraesCS2A01G440700 | histone-lysine N-methyltransferase | NA | NA | NA |
|  |  |  |  |  | TraesCS2A01G440800 | Hypoxia-responsive family protein-like | PF04588: Hypoxia induced protein conserved region | IPR007667: Hypoxia induced protein, domain | NA |
|  |  |  |  |  | TraesCS2A01G440900 | O-methyltransferase family protein, expressed | PF08100: Dimerisation domain; PF00891: O-methyltransferase | IPR001077: O-methyltransferase, family 2; IPR011991: Winged helix-turn-helix DNA-binding domain; IPR012967: Plant methyltransferase dimerisation; IPR016461: O-methyltransferase COMT-type; IPR029063: S-adenosyl-L-methionine-dependent methyltransferase | GO:0008168 MF: methyltransferase activity;GO:0008171 MF: O-methyltransferase activity; |
|  |  |  |  |  | TraesCS2A01G441000 | NA | NA | NA | NA |
|  |  |  |  |  | TraesCS2A01G441100 | O-methyltransferase-like protein | PF08100: Dimerisation domain; PF00891: O-methyltransferase | IPR001077: O-methyltransferase, family 2; IPR011991: Winged helix-turn-helix DNA-binding domain; IPR012967: Plant methyltransferase dimerisation; IPR016461: O-methyltransferase COMT-type; IPR029063: S-adenosyl-L-methionine-dependent methyltransferase | GO:0008168 MF: methyltransferase activity;GO:0008171 MF: O-methyltransferase activity;G |
|  |  |  |  |  | TraesCS2A01G441200 | Nucleolar pre-ribosomal-associated protein 1 | PF11707: Ribosome 60S biogenesis N-terminal; PF16201: Nucleolar pre-ribosomal-associated protein 1 | IPR021714: Nucleolar pre-ribosomal-associated protein 1, N-terminal; IPR032436: Nucleolar pre-ribosomal-associated protein 1, C-terminal domain | NA |
|  |  |  |  |  | TraesCS2A01G441300 | Myb family transcription factor family protein | PF00249: Myb-like DNA-binding domain; PF14379: MYB-CC type transfactor, LHEQLE motif | IPR001005: SANT/Myb domain; IPR006447: Myb domain, plants; IPR009057: Homeobox domain-like; IPR017930: Myb domain; IPR025756: MYB-CC type transcription factor, LHEQLE-containing domain | GO:0003677 MF: DNA binding |
|  |  |  |  |  | TraesCS2A01G441400 | Pathogenesis-related 1 protein | PF00188: Cysteine-rich secretory protein family | IPR001283: Cysteine-rich secretory protein, allergen V5/Tpx-1-related; IPR014044: CAP domain | NA |
|  |  |  |  |  | TraesCS2A01G441500 | Elongator complex protein 1 | NA | NA | NA |
|  |  |  |  |  | TraesCS2A01G441600 | Wound-induced basic | PF08186: Wound-inducible basic protein family | IPR012643: Wound-inducible basic | NA |
|  |  |  |  |  | TraesCS2A01G441700 | Ubiquitin-conjugating enzyme E2 | PF00179: Ubiquitin-conjugating enzyme | IPR000608: Ubiquitin-conjugating enzyme E2; IPR016135: Ubiquitin-conjugating enzyme/RWD-like | NA |
|  |  |  |  |  | TraesCS2A01G441800 | Phosphatidylinositol N-acetyglucosaminlytransferase subunit P-like protein | NA | NA | NA |
|  |  |  |  |  | TraesCS2A01G441900 | Lupus brain antigen 1-like protein | NA | NA | NA |
|  |  |  |  |  | TraesCS2A01G442000 | WD-40 repeat family protein-2 | NA | NA | NA |
|  |  |  |  |  | TraesCS2A01G442100 | Proline--tRNA ligase | NA | NA | NA |
|  |  |  |  |  | TraesCS2A01G442200 | HXXXD-type acyl-transferase family protein | PF02458: Transferase family | IPR003480: Transferase | GO:0016747 MF: transferase activity, transferring acyl groups other than amino-acyl groups |
| STI_RSRatio | AX-158555653 |  | 2A | 691479513 | TraesCS2A01G441600 | Wound-induced basic | PF08186: Wound-inducible basic protein family | IPR012643: Wound-inducible basic | NA |
| STI_RSRatio | AX-158555652 |  | 2A | 691845720 | TraesCS2A01G439800 | Cytochrome P450 | PF00067: Cytochrome P450 | IPR001128: Cytochrome P450; IPR002401: Cytochrome P450, E-class, group I; IPR017972: Cytochrome P450, conserved site | GO:0005506 MF: iron ion binding;GO:0016705 MF: oxidoreductase activity, acting on paired donors, with incorporation or reduction of molecular oxygen; |
|  |  |  |  |  | TraesCS2A01G439900 | CobW-domain-containing protein | PF02492: CobW/HypB/UreG, nucleotide-binding domain; PF07683: Cobalamin synthesis protein cobW C-terminal domain | IPR003495: CobW/HypB/UreG, nucleotide-binding domain; IPR011629: Cobalamin (vitamin B12) biosynthesis CobW-like, C-terminal; IPR027417: P-loop containing nucleoside triphosphate hydrolase | NA |
|  |  |  |  |  | TraesCS2A01G440000 | WD-repeat cell cycle regulatory protein | PF12894: Anaphase-promoting complex subunit 4 WD40 domain; PF00400: WD domain, G-beta repeat | IPR001680: WD40 repeat; IPR015943: WD40/YVTN repeat-like-containing domain; IPR017986: WD40-repeat-containing domain; IPR019775: WD40 repeat, conserved site; IPR024977: Anaphase-promoting complex subunit 4, WD40 domain | GO:0005515 MF: protein binding |
|  |  |  |  |  | TraesCS2A01G440100 | Clathrin interactor EPSIN 1 | PF01417: ENTH domain | IPR008942: ENTH/VHS; IPR013809: ENTH domain | NA |
|  |  |  |  |  | TraesCS2A01G440200 | NA | NA | NA | NA |
|  |  |  |  |  | TraesCS2A01G440300 | Bifunctional inhibitor/lipid-transfer protein/seed storage 2S albumin-like protein | PF14368: Probable lipid transfer | IPR016140: Bifunctional inhibitor/plant lipid transfer protein/seed storage helical domain | NA |
|  |  |  |  |  | TraesCS2A01G440400 | Lipid transfer protein | PF14368: Probable lipid transfer | IPR016140: Bifunctional inhibitor/plant lipid transfer protein/seed storage helical domain | NA |
|  |  |  |  |  | TraesCS2A01G440500 | Protease inhibitor/seed storage/lipid transfer protein family protein | PF14368: Probable lipid transfer | IPR016140: Bifunctional inhibitor/plant lipid transfer protein/seed storage helical domain | NA |
|  |  |  |  |  | TraesCS2A01G440600 | Protease inhibitor/seed storage/lipid transfer protein family protein | PF14368: Probable lipid transfer | IPR016140: Bifunctional inhibitor/plant lipid transfer protein/seed storage helical domain | NA |
|  |  |  |  |  | TraesCS2A01G440700 | histone-lysine N-methyltransferase | NA | NA | NA |
|  |  |  |  |  | TraesCS2A01G440800 | Hypoxia-responsive family protein-like | PF04588: Hypoxia induced protein conserved region | IPR007667: Hypoxia induced protein, domain | NA |
|  |  |  |  |  | TraesCS2A01G440900 | O-methyltransferase family protein, expressed | PF08100: Dimerisation domain; PF00891: O-methyltransferase | IPR001077: O-methyltransferase, family 2; IPR011991: Winged helix-turn-helix DNA-binding domain; IPR012967: Plant methyltransferase dimerisation; IPR016461: O-methyltransferase COMT-type; IPR029063: S-adenosyl-L-methionine-dependent methyltransferase | GO:0008168 MF: methyltransferase activity;GO:0008171 MF: O-methyltransferase activity; |
|  |  |  |  |  | TraesCS2A01G441100 | O-methyltransferase-like protein | PF08100: Dimerisation domain; PF00891: O-methyltransferase | IPR001077: O-methyltransferase, family 2; IPR011991: Winged helix-turn-helix DNA-binding domain; IPR012967: Plant methyltransferase dimerisation; IPR016461: O-methyltransferase COMT-type; IPR029063: S-adenosyl-L-methionine-dependent methyltransferase | GO:0008168 MF: methyltransferase activity;GO:0008171 MF: O-methyltransferase activity; |
|  |  |  |  |  | TraesCS2A01G441200 | Nucleolar pre-ribosomal-associated protein 1 | PF11707: Ribosome 60S biogenesis N-terminal; PF16201: Nucleolar pre-ribosomal-associated protein 1 | IPR021714: Nucleolar pre-ribosomal-associated protein 1, N-terminal; IPR032436: Nucleolar pre-ribosomal-associated protein 1, C-terminal domain | NA |
|  |  |  |  |  | TraesCS2A01G441300 | Myb family transcription factor family protein | PF00249: Myb-like DNA-binding domain; PF14379: MYB-CC type transfactor, LHEQLE motif | IPR001005: SANT/Myb domain; IPR006447: Myb domain, plants; IPR009057: Homeobox domain-like; IPR017930: Myb domain; IPR025756: MYB-CC type transcription factor, LHEQLE-containing domain | GO:0003677 MF: DNA binding |
|  |  |  |  |  | TraesCS2A01G441400 | Pathogenesis-related 1 protein | PF00188: Cysteine-rich secretory protein family | IPR001283: Cysteine-rich secretory protein, allergen V5/Tpx-1-related; IPR014044: CAP domain | NA |
|  |  |  |  |  | TraesCS2A01G441500 | Elongator complex protein 1 | NA | NA | NA |
|  |  |  |  |  | TraesCS2A01G441600 | Wound-induced basic | PF08186: Wound-inducible basic protein family | IPR012643: Wound-inducible basic | NA |
|  |  |  |  |  | TraesCS2A01G441700 | Ubiquitin-conjugating enzyme E2 | PF00179: Ubiquitin-conjugating enzyme | IPR000608: Ubiquitin-conjugating enzyme E2; IPR016135: Ubiquitin-conjugating enzyme/RWD-like | NA |
|  |  |  |  |  | TraesCS2A01G441800 | Phosphatidylinositol N-acetyglucosaminlytransferase subunit P-like protein | NA | NA | NA |
|  |  |  |  |  | TraesCS2A01G441900 | Lupus brain antigen 1-like protein | NA | NA | NA |
|  |  |  |  |  | TraesCS2A01G442000 | WD-40 repeat family protein-2 | NA | NA | NA |
|  |  |  |  |  | TraesCS2A01G442100 | Proline--tRNA ligase | NA | NA | NA |
|  |  |  |  |  | TraesCS2A01G442200 | HXXXD-type acyl-transferase family protein | PF02458: Transferase family | IPR003480: Transferase | GO:0016747 MF: transferase activity, transferring acyl groups other than amino-acyl groups |
|  |  |  |  |  | TraesCS2A01G442300 | p-loop containing nucleoside triphosphate hydrolases superfamily protein, putative | PF13086: AAA domain; PF13087: AAA domain; PF00580: UvrD/REP helicase N-terminal domain | IPR011990: Tetratricopeptide-like helical domain; IPR027417: P-loop containing nucleoside triphosphate hydrolase; IPR034739: UvrD/AddA helicase, N-terminal | GO:0005515 MF: protein binding;GO:0005524 MF: ATP binding |
|  |  |  |  |  | TraesCS2A01G442400 | Phospholipid scramblase | PF03803: Scramblase | IPR005552: Scramblase; IPR025659: Tubby C-terminal-like domain | NA |
|  |  |  |  |  | TraesCS2A01G442500 | Basic helix loop helix (BHLH) family transcription factor | PF00010: Helix-loop-helix DNA-binding domain | IPR011598: Myc-type, basic helix-loop-helix (bHLH) domain | GO:0046983 MF: protein dimerization activity |
|  |  |  |  |  | TraesCS2A01G442600 | Glycosyltransferase | PF04577: Protein of unknown function (DUF563) | IPR007657: Glycosyltransferase 61 | GO:0016757 MF: transferase activity, transferring glycosyl groups |
|  |  |  |  |  | TraesCS2A01G442700 | Basic helix-loop-helix (BHLH) Transcription Factor | PF00010: Helix-loop-helix DNA-binding domain | IPR011598: Myc-type, basic helix-loop-helix (bHLH) domain | GO:0046983 MF: protein dimerization activity |
|  |  |  |  |  | TraesCS2A01G442800 | Tudor/PWWP/MBT superfamily protein | PF00855: PWWP domain | IPR000313: PWWP domain | NA |
| STI_RSRatio | RAC875_c52458_454 |  | 2A | 692755001 | TraesCS2A01G442900 | Receptor-like protein kinase | PF13947: Wall-associated receptor kinase galacturonan-binding; PF08488: Wall-associated kinase; PF07714: Protein tyrosine kinase | IPR000152: EGF-type aspartate/asparagine hydroxylation site; IPR000719: Protein kinase domain; IPR000742: EGF-like domain; IPR001245: Serine-threonine/tyrosine-protein kinase, catalytic domain; IPR001881: EGF-like calcium-binding domain; IPR008271: Serine/threonine-protein kinase, active site; IPR011009: Protein kinase-like domain; IPR013320: Concanavalin A-like lectin/glucanase domain; IPR013695: Wall-associated receptor kinase; IPR017441: Protein kinase, ATP binding site; IPR018097: EGF-like calcium-binding, conserved site; IPR025287: Wall-associated receptor kinase, galacturonan-binding domain | GO:0004672 MF: protein kinase activity;GO:0004674 MF: protein serine/threonine kinase activity; |
|  |  |  |  |  | TraesCS2A01G443000 | Kinase, putative | PF00069: Protein kinase domain | IPR000719: Protein kinase domain; IPR008271: Serine/threonine-protein kinase, active site; IPR011009: Protein kinase-like domain; IPR017441: Protein kinase, ATP binding site | GO:0004672 MF: protein kinase activity;GO:0005524 MF: ATP binding; |
| STI_RSRatio | BobWhite_c17403_635 |  | 2A | 693241897 | TraesCS2A01G443200 | 50S ribosomal protein L11 | PF03946: Ribosomal protein L11, N-terminal domain; PF00298: Ribosomal protein L11, RNA binding domain | IPR000911: Ribosomal protein L11/L12; IPR020783: Ribosomal protein L11, C-terminal; IPR020784: Ribosomal protein L11, N-terminal; IPR020785: Ribosomal protein L11, conserved site | GO:0003735 MF: structural constituent of ribosome;GO:0005840 CC: ribosome; |
| STI_RSRatio | AX-158572604 |  | 2A | 706575588 | TraesCS2A01G457000 | RING/U-box superfamily protein | PF12274: Protein of unknown function (DUF3615) | IPR022059: Protein of unknown function DUF3615 | NA |
|  |  |  |  |  | TraesCS2A01G457100 | Protein SET DOMAIN GROUP 40 | PF00856: SET domain; PF09273: Rubisco LSMT substrate-binding | IPR001214: SET domain; IPR015353: Rubisco LSMT, substrate-binding domain | GO:0005515 MF: protein binding |
|  |  |  |  |  | TraesCS2A01G457200 | Phosphate acyltransferase | NA | NA | NA |
|  |  |  |  |  | TraesCS2A01G457300 | Prefoldin subunit 2 | PF01920: Prefoldin subunit | IPR002777: Prefoldin beta-like | GO:0006457 BP: protein folding;GO:0016272 CC: prefoldin complex;GO:0051082 MF: unfolded protein binding |
|  |  |  |  |  | TraesCS2A01G457400 | ABC transporter G family member 25 | NA | NA | NA |
|  |  |  |  |  | TraesCS2A01G457500 | Structural maintenance of chromosomes protein | PF02463: RecF/RecN/SMC N terminal domain; PF06470: SMC proteins Flexible Hinge Domain | IPR003395: RecF/RecN/SMC, N-terminal; IPR010935: SMCs flexible hinge; IPR024704: Structural maintenance of chromosomes protein; IPR027417: P-loop containing nucleoside triphosphate hydrolase | GO:0005515 MF: protein binding;GO:0005524 MF: ATP binding; |
|  |  |  |  |  | TraesCS2A01G457600 | ATP-dependent zinc metalloprotease FtsH | PF00004: ATPase family associated with various cellular activities (AAA) | IPR003593: AAA+ ATPase domain; IPR003959: ATPase, AAA-type, core; IPR003960: ATPase, AAA-type, conserved site; IPR027417: P-loop containing nucleoside triphosphate hydrolase | GO:0005524 MF: ATP binding |
|  |  |  |  |  | TraesCS2A01G457700 | Calcium-dependent lipid-binding (CaLB domain) family protein | NA | IPR000008: C2 domain | GO:0005515 MF: protein binding |
|  |  |  |  |  | TraesCS2A01G457800 | tRNA (Cytosine(34)-C(5))-methyltransferase | NA | IPR012337: Ribonuclease H-like domain | GO:0003676 MF: nucleic acid binding |
|  |  |  |  |  | TraesCS2A01G457900 | D-2-hydroxyglutarate dehydrogenase | PF01565: FAD binding domain; PF02913: FAD linked oxidases, C-terminal domain | IPR004113: FAD-linked oxidase, C-terminal; IPR006094: FAD linked oxidase, N-terminal; IPR016164: FAD-linked oxidase-like, C-terminal; IPR016166: FAD-binding, type 2; IPR016167: FAD-binding, type 2, subdomain 1; IPR016169: CO dehydrogenase flavoprotein-like, FAD-binding, subdomain 2; IPR016171: Vanillyl-alcohol oxidase, C-terminal subdomain 2 | GO:0003824 MF: catalytic activity;GO:0016491 MF: oxidoreductase activity; |
|  |  |  |  |  | TraesCS2A01G458000 | UBiQuitin family member | PF00240: Ubiquitin family; PF01020: Ribosomal L40e family | IPR000626: Ubiquitin domain; IPR001975: Ribosomal protein L40e; IPR011332: Zinc-binding ribosomal protein; IPR019954: Ubiquitin conserved site; IPR019956: Ubiquitin; IPR029071: Ubiquitin-related domain | GO:0003735 MF: structural constituent of ribosome;GO:0005515 MF: protein binding; |
|  |  |  |  |  | TraesCS2A01G458100 | UBiQuitin family member | PF00240: Ubiquitin family; PF01020: Ribosomal L40e family | IPR000626: Ubiquitin domain; IPR001975: Ribosomal protein L40e; IPR011332: Zinc-binding ribosomal protein; IPR019954: Ubiquitin conserved site; IPR019956: Ubiquitin; IPR029071: Ubiquitin-related domain | GO:0003735 MF: structural constituent of ribosome;GO:0005515 MF: protein binding; |
|  |  |  |  |  | TraesCS2A01G458200 | GRF zinc finger family protein, expressed | NA | NA | NA |
|  |  |  |  |  | TraesCS2A01G458500 | BTB/POZ domain containing protein, expressed | PF00651: BTB/POZ domain | IPR000210: BTB/POZ domain; IPR011333: SKP1/BTB/POZ domain | GO:0005515 MF: protein binding |
|  |  |  |  |  | TraesCS2A01G458600 | Ubiquitin | PF00240: Ubiquitin family; PF01020: Ribosomal L40e family | IPR000626: Ubiquitin domain; IPR001975: Ribosomal protein L40e; IPR011332: Zinc-binding ribosomal protein; IPR019954: Ubiquitin conserved site; IPR019956: Ubiquitin; IPR029071: Ubiquitin-related domain | GO:0003735 MF: structural constituent of ribosome;GO:0005515 MF: protein binding; |
|  |  |  |  |  | TraesCS2A01G458700 | BTB/POZ domain containing protein, expressed | PF00651: BTB/POZ domain | IPR000210: BTB/POZ domain; IPR011333: SKP1/BTB/POZ domain | GO:0005515 MF: protein binding |
|  |  |  |  |  | TraesCS2A01G458800 | Ubiquitin | PF00240: Ubiquitin family; PF01020: Ribosomal L40e family | IPR000626: Ubiquitin domain; IPR001975: Ribosomal protein L40e; IPR011332: Zinc-binding ribosomal protein; IPR019956: Ubiquitin; IPR029071: Ubiquitin-related domain | GO:0003735 MF: structural constituent of ribosome;GO:0005515 MF: protein binding; |
|  |  |  |  |  | TraesCS2A01G458900 | Expansin protein | PF03330: Lytic transglycolase; PF01357: Pollen allergen | IPR005795: Major pollen allergen Lol pI; IPR007112: Expansin/pollen allergen, DPBB domain; IPR007117: Expansin, cellulose-binding-like domain; IPR007118: Expansin/Lol pI; IPR009009: RlpA-like protein, double-psi beta-barrel domain | GO:0005576 CC: extracellular region;GO:0019953 BP: sexual reproduction |
|  |  |  |  |  | TraesCS2A01G459000 | 30S ribosomal protein S11 | PF00411: Ribosomal protein S11 | IPR001971: Ribosomal protein S11 | GO:0003735 MF: structural constituent of ribosome;GO:0005840 CC: ribosome; |
|  |  |  |  |  | TraesCS2A01G459100 | NBS-LRR disease resistance protein | PF00931: NB-ARC domain | IPR002182: NB-ARC; IPR011991: Winged helix-turn-helix DNA-binding domain; IPR027417: P-loop containing nucleoside triphosphate hydrolase; IPR032675: Leucine-rich repeat domain, L domain-like | GO:0043531 MF: ADP binding |
|  |  |  |  |  | TraesCS2A01G459200 | MADS-box transcription factor family protein | PF00319: SRF-type transcription factor (DNA-binding and dimerisation domain) | IPR002100: Transcription factor, MADS-box | GO:0003677 MF: DNA binding;GO:0046983 MF: protein dimerization activity |
|  |  |  |  |  | TraesCS2A01G459300 | filamentous hemagglutinin transporter | NA | NA | NA |
|  |  |  |  |  | TraesCS2A01G459400 | filamentous hemagglutinin transporter | NA | NA | NA |
|  |  |  |  |  | TraesCS2A01G459500 | filamentous hemagglutinin transporter | NA | NA | NA |
|  |  |  |  |  | TraesCS2A01G459600 | Imidazoleglycerol-phosphate dehydratase | PF00475: Imidazoleglycerol-phosphate dehydratase | IPR000807: Imidazoleglycerol-phosphate dehydratase; IPR020565: Imidazoleglycerol-phosphate dehydratase, conserved site; IPR020568: Ribosomal protein S5 domain 2-type fold | GO:0000105 BP: histidine biosynthetic process;GO:0004424 MF: imidazoleglycerol-phosphate dehydratase activity |
|  |  |  |  |  | TraesCS2A01G459700 | NAC domain protein | PF02365: No apical meristem (NAM) protein | IPR003441: NAC domain | GO:0003677 MF: DNA binding;GO:0006355 BP: regulation of transcription, DNA-templated |
|  |  |  |  |  | TraesCS2A01G459800 | Germin-like protein | PF00190: Cupin | IPR001929: Germin; IPR006045: Cupin 1; IPR011051: RmlC-like cupin domain; IPR014710: RmlC-like jelly roll fold; IPR019780: Germin, manganese binding site | GO:0030145 MF: manganese ion binding;GO:0045735 MF: nutrient reservoir activity |
|  |  |  |  |  | TraesCS2A01G459900 | Germin-like protein | PF00190: Cupin | IPR001929: Germin; IPR006045: Cupin 1; IPR011051: RmlC-like cupin domain; IPR014710: RmlC-like jelly roll fold; IPR019780: Germin, manganese binding site | GO:0030145 MF: manganese ion binding;GO:0045735 MF: nutrient reservoir activity |
|  |  |  |  |  | TraesCS2A01G460000 | GPI mannosyltransferase 2 | PF04188: Mannosyltransferase (PIG-V) | IPR007315: GPI mannosyltransferase 2 | GO:0004584 MF: dolichyl-phosphate-mannose-glycolipid alpha-mannosyltransferase activity;GO:0006506 BP: GPI anchor biosynthetic process |
|  |  |  |  |  | TraesCS2A01G460100 | Pentatricopeptide repeat-containing protein | PF13812: Pentatricopeptide repeat domain; PF13041: PPR repeat family; PF01535: PPR repeat | IPR002885: Pentatricopeptide repeat | NA |
|  |  |  |  |  | TraesCS2A01G460200 | Nodulation signaling protein 1 | PF03514: GRAS domain family | IPR005202: Transcription factor GRAS | NA |
|  |  |  |  |  | TraesCS2A01G460300 | B3 domain-containing protein family | NA | IPR003340: B3 DNA binding domain; IPR015300: DNA-binding pseudobarrel domain | GO:0003677 MF: DNA binding |
|  |  |  |  |  | TraesCS2A01G460400 | UDP-glucose 4-epimerase, putative | PF16363: GDP-mannose 4,6 dehydratase | IPR005886: UDP-glucose 4-epimerase; IPR016040: NAD(P)-binding domain | GO:0003978 MF: UDP-glucose 4-epimerase activity;GO:0006012 BP: galactose metabolic process |
|  |  |  |  |  | TraesCS2A01G460500 | NT-3 growth factor receptor | NA | NA | NA |
|  |  |  |  |  | TraesCS2A01G460600 | Leguminosin group485 secreted peptide | NA | NA | NA |
|  |  |  |  |  | TraesCS2A01G460700 | B3 domain-containing protein | PF02362: B3 DNA binding domain | IPR003340: B3 DNA binding domain; IPR015300: DNA-binding pseudobarrel domain | GO:0003677 MF: DNA binding |
|  |  |  |  |  | TraesCS2A01G460800 | PAP/OAS1 substrate-binding domain superfamily | NA | NA | NA |
|  |  |  |  |  | TraesCS2A01G460900 | BLT14.1 protein | NA | NA | NA |
|  |  |  |  |  | TraesCS2A01G461000 | Homeodomain-like superfamily protein | NA | NA | NA |
|  |  |  |  |  | TraesCS2A01G461100 | BLT14.2 protein | NA | NA | NA |
|  |  |  |  |  | TraesCS2A01G461200 | BLT14.1 protein | NA | NA | NA |
|  |  |  |  |  | TraesCS2A01G461300 | Collagen alpha-1(IV) chain | NA | NA | NA |
|  |  |  |  |  | TraesCS2A01G461400 | NA | NA | NA | NA |
|  |  |  |  |  | TraesCS2A01G461500 | Collagen alpha-1(IV) chain | NA | NA | NA |
|  |  |  |  |  | TraesCS2A01G461600 | Gamma-soluble NSF attachment protein | PF14938: Soluble NSF attachment protein, SNAP | IPR011990: Tetratricopeptide-like helical domain | GO:0005515 MF: protein binding |
|  |  |  |  |  | TraesCS2A01G461700 | Basic helix-loop-helix transcription factor | PF00010: Helix-loop-helix DNA-binding domain | IPR011598: Myc-type, basic helix-loop-helix (bHLH) domain | GO:0046983 MF: protein dimerization activity |
| STI_RSRatio | AX-158572603 |  | 2A | 706757509 | TraesCS2A01G461800 | Receptor-like kinase | PF08263: Leucine rich repeat N-terminal domain; PF00560: Leucine Rich Repeat; PF13855: Leucine rich repeat; PF13516: Leucine Rich repeat; PF00069: Protein kinase domain | IPR000719: Protein kinase domain; IPR001611: Leucine-rich repeat; IPR003591: Leucine-rich repeat, typical subtype; IPR008271: Serine/threonine-protein kinase, active site; IPR011009: Protein kinase-like domain; IPR013210: Leucine-rich repeat-containing N-terminal, plant-type; IPR032675: Leucine-rich repeat domain, L domain-like | GO:0004672 MF: protein kinase activity;GO:0005515 MF: protein binding; |
|  |  |  |  |  | TraesCS2A01G461900 | Leucine-rich repeat receptor-like protein kinase family protein | PF08263: Leucine rich repeat N-terminal domain; PF00560: Leucine Rich Repeat; PF13855: Leucine rich repeat | IPR001611: Leucine-rich repeat; IPR003591: Leucine-rich repeat, typical subtype; IPR013210: Leucine-rich repeat-containing N-terminal, plant-type; IPR032675: Leucine-rich repeat domain, L domain-like | GO:0005515 MF: protein binding |
|  |  |  |  |  | TraesCS2A01G462000 | Glutathione S-transferase T3 | NA | NA | NA |
|  |  |  |  |  | TraesCS2A01G462100 | DTW domain containing protein, expressed | PF03942: DTW domain | IPR005636: DTW | NA |
| STI_RSRatio | AX-111016876 |  | 2A | 717417914 | TraesCS2A01G477000 | Pathogenesis-related protein 1 | PF00407: Pathogenesis-related protein Bet v I family | IPR000916: Bet v I/Major latex protein; IPR023393: START-like domain; IPR024949: Bet v I type allergen | GO:0006952 BP: defense response;GO:0009607 BP: response to biotic stimulus |
|  |  |  |  |  | TraesCS2A01G477100 | NA | NA | NA | NA |
|  |  |  |  |  | TraesCS2A01G477200 | NBS-LRR disease resistance protein | PF00931: NB-ARC domain | IPR002182: NB-ARC; IPR011991: Winged helix-turn-helix DNA-binding domain; IPR027417: P-loop containing nucleoside triphosphate hydrolase; IPR032675: Leucine-rich repeat domain, L domain-like | GO:0043531 MF: ADP binding |
|  |  |  |  |  | TraesCS2A01G477300 | NA | NA | NA | NA |
|  |  |  |  |  | TraesCS2A01G477400 | GDSL esterase/lipase | PF00657: GDSL-like Lipase/Acylhydrolase | IPR001087: GDSL lipase/esterase; IPR013830: SGNH hydrolase-type esterase domain | GO:0016788 MF: hydrolase activity, acting on ester bonds |
|  |  |  |  |  | TraesCS2A01G477500 | Pentatricopeptide repeat-containing protein | PF01535: PPR repeat; PF13041: PPR repeat family | IPR002885: Pentatricopeptide repeat; IPR011990: Tetratricopeptide-like helical domain | GO:0005515 MF: protein binding |
|  |  |  |  |  | TraesCS2A01G477600 | Non-specific lipid-transfer protein | PF00234: Protease inhibitor/seed storage/LTP family | IPR000528: Plant lipid transfer protein/Par allergen; IPR016140: Bifunctional inhibitor/plant lipid transfer protein/seed storage helical domain | GO:0006869 BP: lipid transport;GO:0008289 MF: lipid binding |
|  |  |  |  |  | TraesCS2A01G477700 | Non-specific lipid-transfer protein | PF00234: Protease inhibitor/seed storage/LTP family | IPR000528: Plant lipid transfer protein/Par allergen; IPR016140: Bifunctional inhibitor/plant lipid transfer protein/seed storage helical domain | GO:0006869 BP: lipid transport;GO:0008289 MF: lipid binding |
|  |  |  |  |  | TraesCS2A01G477800 | NA | NA | NA | NA |
|  |  |  |  |  | TraesCS2A01G477900 | Aldehyde dehydrogenase | PF00171: Aldehyde dehydrogenase family | IPR010061: Methylmalonate-semialdehyde dehydrogenase; IPR015590: Aldehyde dehydrogenase domain; IPR016160: Aldehyde dehydrogenase, cysteine active site; IPR016161: Aldehyde/histidinol dehydrogenase; IPR016162: Aldehyde dehydrogenase N-terminal domain | GO:0004491 MF: methylmalonate-semialdehyde dehydrogenase (acylating) activity;GO:0008152 BP: metabolic process; |
|  |  |  |  |  | TraesCS2A01G478000 | Histone-lysine N-methyltransferase | PF10440: Ubiquitin-binding WIYLD domain; PF05033: Pre-SET motif; PF00856: SET domain | IPR001214: SET domain; IPR007728: Pre-SET domain; IPR018848: WIYLD domain; IPR025776: Histone-lysine N-methyltransferase SUVR4/SUVR1/SUVR2 | GO:0005515 MF: protein binding;GO:0005634 CC: nucleus; |
|  |  |  |  |  | TraesCS2A01G478100 | Linalool synthase, chloroplastic | PF01397: Terpene synthase, N-terminal domain; PF03936: Terpene synthase family, metal binding domain | IPR001906: Terpene synthase, N-terminal domain; IPR005630: Terpene synthase, metal-binding domain; IPR008930: Terpenoid cyclases/protein prenyltransferase alpha-alpha toroid; IPR008949: Isoprenoid synthase domain | GO:0000287 MF: magnesium ion binding;GO:0008152 BP: metabolic process; |
|  |  |  |  |  | TraesCS2A01G478200 | NA | NA | NA | NA |
|  |  |  |  |  | TraesCS2A01G478300 | UHRF1-binding protein 1 | PF12624: N-terminal region of Chorein or VPS13 | IPR026854: Vacuolar protein sorting-associated protein 13, N-terminal domain | NA |
|  |  |  |  |  | TraesCS2A01G478400 | UDP-N-acetylmuramate--L-alanine ligase | NA | NA | NA |
|  |  |  |  |  | TraesCS2A01G478500 | Beta-glucosidase | PF00933: Glycosyl hydrolase family 3 N terminal domain; PF01915: Glycosyl hydrolase family 3 C-terminal domain | IPR001764: Glycoside hydrolase, family 3, N-terminal; IPR002772: Glycoside hydrolase family 3 C-terminal domain; IPR017853: Glycoside hydrolase superfamily; IPR019800: Glycoside hydrolase, family 3, active site | GO:0004553 MF: hydrolase activity, hydrolyzing O-glycosyl compounds;GO:0005975 BP: carbohydrate metabolic process |
|  |  |  |  |  | TraesCS2A01G478600 | Sugar transport protein 5 | NA | NA | NA |
|  |  |  |  |  | TraesCS2A01G478700 | Glycosyltransferase | PF00201: UDP-glucoronosyl and UDP-glucosyl transferase | IPR002213: UDP-glucuronosyl/UDP-glucosyltransferase | GO:0008152 BP: metabolic process;GO:0016758 MF: transferase activity, transferring hexosyl groups |
|  |  |  |  |  | TraesCS2A01G478800 | Histone-lysine N-methyltransferase | PF10440: Ubiquitin-binding WIYLD domain; PF05033: Pre-SET motif; PF00856: SET domain | IPR001214: SET domain; IPR007728: Pre-SET domain; IPR018848: WIYLD domain; IPR025776: Histone-lysine N-methyltransferase SUVR4/SUVR1/SUVR2 | GO:0005515 MF: protein binding;GO:0005634 CC: nucleus;GO:0008270 MF: zinc ion binding;GO:0018024 MF: histone-lysine N-methyltransferase activity;GO:0034968 BP: histone lysine methylation |
|  |  |  |  |  | TraesCS2A01G478900 | Glycosyltransferase | PF00201: UDP-glucoronosyl and UDP-glucosyl transferase | IPR002213: UDP-glucuronosyl/UDP-glucosyltransferase | GO:0008152 BP: metabolic process;GO:0016758 MF: transferase activity, transferring hexosyl groups |
|  |  |  |  |  | TraesCS2A01G479000 | Phosphatidylinositol-4-phosphate 5-kinase, putative | PF02493: MORN repeat; PF01504: Phosphatidylinositol-4-phosphate 5-Kinase | IPR002498: Phosphatidylinositol-4-phosphate 5-kinase, core; IPR003409: MORN motif; IPR017163: Phosphatidylinositol-4-phosphate 5-kinase, plant; IPR027483: Phosphatidylinositol-4-phosphate 5-kinase, C-terminal; IPR027484: Phosphatidylinositol-4-phosphate 5-kinase, N-terminal domain | GO:0005524 MF: ATP binding;GO:0016307 MF: phosphatidylinositol phosphate kinase activity; |
|  |  |  |  |  | TraesCS2A01G479100 | Cyclin family protein | PF08613: Cyclin | IPR013763: Cyclin-like; IPR013922: Cyclin PHO80-like | GO:0000079 BP: regulation of cyclin-dependent protein serine/threonine kinase activity;GO:0019901 MF: protein kinase binding |
|  |  |  |  |  | TraesCS2A01G479200 | F-box family protein | NA | NA | NA |
|  |  |  |  |  | TraesCS2A01G479300 | ATP/DNA-binding protein | NA | IPR003594: Histidine kinase-like ATPase, C-terminal domain | NA |
|  |  |  |  |  | TraesCS2A01G479400 | Peroxisome biogenesis protein 22 | NA | NA | NA |
|  |  |  |  |  | TraesCS2A01G479500 | Histone-lysine N-methyltransferase | PF00856: SET domain; PF01753: MYND finger | IPR001214: SET domain; IPR002893: Zinc finger, MYND-type | GO:0005515 MF: protein binding |
|  |  |  |  |  | TraesCS2A01G479600 | CsAtPR5 | NA | NA | NA |
|  |  |  |  |  | TraesCS2A01G479700 | Invertase/pectin methylesterase inhibitor family protein | PF04043: Plant invertase/pectin methylesterase inhibitor | IPR006501: Pectinesterase inhibitor domain | GO:0004857 MF: enzyme inhibitor activity |
|  |  |  |  |  | TraesCS2A01G479800 | CsAtPR5, putative, expressed | NA | NA | NA |
|  |  |  |  |  | TraesCS2A01G479900 | SNF2 domain-containing protein / helicase domain-containing protein / zinc finger protein-like protein | PF00176: SNF2 family N-terminal domain; PF14634: zinc-RING finger domain; PF00271: Helicase conserved C-terminal domain | IPR000330: SNF2-related, N-terminal domain; IPR001650: Helicase, C-terminal; IPR001841: Zinc finger, RING-type; IPR013083: Zinc finger, RING/FYVE/PHD-type; IPR014001: Helicase superfamily 1/2, ATP-binding domain; IPR017907: Zinc finger, RING-type, conserved site; IPR027417: P-loop containing nucleoside triphosphate hydrolase | GO:0005515 MF: protein binding;GO:0005524 MF: ATP binding; |
|  |  |  |  |  | TraesCS2A01G480000 | Phosphatidylinositol 3- and 4-kinase family protein | NA | NA | NA |
|  |  |  |  |  | TraesCS2A01G480100 | NBS-LRR disease resistance protein, putative, expressed | PF00931: NB-ARC domain | IPR002182: NB-ARC; IPR011991: Winged helix-turn-helix DNA-binding domain; IPR032675: Leucine-rich repeat domain, L domain-like | GO:0043531 MF: ADP binding |
|  |  |  |  |  | TraesCS2A01G480200 | Divalent-cation tolerance protein CutA | PF03091: CutA1 divalent ion tolerance protein | IPR004323: Divalent ion tolerance protein, CutA; IPR011322: Nitrogen regulatory PII-like, alpha/beta; IPR015867: Nitrogen regulatory protein PII/ATP phosphoribosyltransferase, C-terminal | GO:0010038 BP: response to metal ion |
|  |  |  |  |  | TraesCS2A01G480300 | Thioredoxin | PF00085: Thioredoxin | IPR012336: Thioredoxin-like fold; IPR013766: Thioredoxin domain | GO:0045454 BP: cell redox homeostasis |
|  |  |  |  |  | TraesCS2A01G480400 | NBS-LRR disease resistance protein | PF00931: NB-ARC domain | IPR002182: NB-ARC; IPR011991: Winged helix-turn-helix DNA-binding domain; IPR027417: P-loop containing nucleoside triphosphate hydrolase; IPR032675: Leucine-rich repeat domain, L domain-like | GO:0043531 MF: ADP binding |
|  |  |  |  |  | TraesCS2A01G480500 | Kinesin-like protein | PF00225: Kinesin motor domain | IPR001752: Kinesin motor domain; IPR019821: Kinesin motor domain, conserved site; IPR027417: P-loop containing nucleoside triphosphate hydrolase | GO:0003777 MF: microtubule motor activity;GO:0005524 MF: ATP binding; |
|  |  |  |  |  | TraesCS2A01G480600 | Kinase-like | PF00069: Protein kinase domain | IPR000719: Protein kinase domain; IPR008271: Serine/threonine-protein kinase, active site; IPR011009: Protein kinase-like domain; IPR017441: Protein kinase, ATP binding site | GO:0004672 MF: protein kinase activity;GO:0005524 MF: ATP binding; |
|  |  |  |  |  | TraesCS2A01G480700 | Sodium- and chloride-dependent creatine transporter 1 | NA | NA | NA |
|  |  |  |  |  | TraesCS2A01G480800 | Serine/threonine-protein kinase | NA | NA | NA |
|  |  |  |  |  | TraesCS2A01G480900 | Glycosyltransferase | PF04577: Protein of unknown function (DUF563) | IPR007657: Glycosyltransferase 61 | GO:0016757 MF: transferase activity, transferring glycosyl groups |
| STI_RSRatio | AX-158557238 |  | 2A | 717418634 | TraesCS2A01G479100 | Cyclin family protein | PF08613: Cyclin | IPR013763: Cyclin-like; IPR013922: Cyclin PHO80-like | GO:0000079 BP: regulation of cyclin-dependent protein serine/threonine kinase activity;GO:0019901 MF: protein kinase binding |
|  |  |  |  |  | TraesCS2A01G479200 | F-box family protein | NA | NA | NA |
|  |  |  |  |  | TraesCS2A01G479300 | ATP/DNA-binding protein | NA | IPR003594: Histidine kinase-like ATPase, C-terminal domain | NA |
|  |  |  |  |  | TraesCS2A01G479400 | Peroxisome biogenesis protein 22 | NA | NA | NA |
|  |  |  |  |  | TraesCS2A01G479500 | Histone-lysine N-methyltransferase | PF00856: SET domain; PF01753: MYND finger | IPR001214: SET domain; IPR002893: Zinc finger, MYND-type | GO:0005515 MF: protein binding |
|  |  |  |  |  | TraesCS2A01G479600 | CsAtPR5 | NA | NA | NA |
|  |  |  |  |  | TraesCS2A01G479700 | Invertase/pectin methylesterase inhibitor family protein | PF04043: Plant invertase/pectin methylesterase inhibitor | IPR006501: Pectinesterase inhibitor domain | GO:0004857 MF: enzyme inhibitor activity |
|  |  |  |  |  | TraesCS2A01G479800 | CsAtPR5, putative, expressed | NA | NA | NA |
|  |  |  |  |  | TraesCS2A01G479900 | SNF2 domain-containing protein / helicase domain-containing protein / zinc finger protein-like protein | PF00176: SNF2 family N-terminal domain; PF14634: zinc-RING finger domain; PF00271: Helicase conserved C-terminal domain | IPR000330: SNF2-related, N-terminal domain; IPR001650: Helicase, C-terminal; IPR001841: Zinc finger, RING-type; IPR013083: Zinc finger, RING/FYVE/PHD-type; IPR014001: Helicase superfamily 1/2, ATP-binding domain; IPR017907: Zinc finger, RING-type, conserved site; IPR027417: P-loop containing nucleoside triphosphate hydrolase | GO:0005515 MF: protein binding;GO:0005524 MF: ATP binding; |
|  |  |  |  |  | TraesCS2A01G480000 | Phosphatidylinositol 3- and 4-kinase family protein | NA | NA | NA |
|  |  |  |  |  | TraesCS2A01G480100 | NBS-LRR disease resistance protein, putative, expressed | PF00931: NB-ARC domain | IPR002182: NB-ARC; IPR011991: Winged helix-turn-helix DNA-binding domain; IPR032675: Leucine-rich repeat domain, L domain-like | GO:0043531 MF: ADP binding |
|  |  |  |  |  | TraesCS2A01G480200 | Divalent-cation tolerance protein CutA | PF03091: CutA1 divalent ion tolerance protein | IPR004323: Divalent ion tolerance protein, CutA; IPR011322: Nitrogen regulatory PII-like, alpha/beta; IPR015867: Nitrogen regulatory protein PII/ATP phosphoribosyltransferase, C-terminal | GO:0010038 BP: response to metal ion |
|  |  |  |  |  | TraesCS2A01G480300 | Thioredoxin | PF00085: Thioredoxin | IPR012336: Thioredoxin-like fold; IPR013766: Thioredoxin domain | GO:0045454 BP: cell redox homeostasis |
|  |  |  |  |  | TraesCS2A01G480400 | NBS-LRR disease resistance protein | PF00931: NB-ARC domain | IPR002182: NB-ARC; IPR011991: Winged helix-turn-helix DNA-binding domain; IPR027417: P-loop containing nucleoside triphosphate hydrolase; IPR032675: Leucine-rich repeat domain, L domain-like | GO:0043531 MF: ADP binding |
|  |  |  |  |  | TraesCS2A01G480500 | Kinesin-like protein | PF00225: Kinesin motor domain | IPR001752: Kinesin motor domain; IPR019821: Kinesin motor domain, conserved site; IPR027417: P-loop containing nucleoside triphosphate hydrolase | GO:0003777 MF: microtubule motor activity;GO:0005524 MF: ATP binding; |
|  |  |  |  |  | TraesCS2A01G480600 | Kinase-like | PF00069: Protein kinase domain | IPR000719: Protein kinase domain; IPR008271: Serine/threonine-protein kinase, active site; IPR011009: Protein kinase-like domain; IPR017441: Protein kinase, ATP binding site | GO:0004672 MF: protein kinase activity;GO:0005524 MF: ATP binding; |
|  |  |  |  |  | TraesCS2A01G480700 | Sodium- and chloride-dependent creatine transporter 1 | NA | NA | NA |
|  |  |  |  |  | TraesCS2A01G480800 | Serine/threonine-protein kinase | NA | NA | NA |
|  |  |  |  |  | TraesCS2A01G480900 | Glycosyltransferase | PF04577: Protein of unknown function (DUF563) | IPR007657: Glycosyltransferase 61 | GO:0016757 MF: transferase activity, transferring glycosyl groups |
| STI_RSRatio | AX-109854150 |  | 2A | 717857707 | TraesCS2A01G477800 | NA | NA | NA | NA |
|  |  |  |  |  | TraesCS2A01G477900 | Aldehyde dehydrogenase | PF00171: Aldehyde dehydrogenase family | IPR010061: Methylmalonate-semialdehyde dehydrogenase; IPR015590: Aldehyde dehydrogenase domain; IPR016160: Aldehyde dehydrogenase, cysteine active site; IPR016161: Aldehyde/histidinol dehydrogenase; IPR016162: Aldehyde dehydrogenase N-terminal domain | GO:0004491 MF: methylmalonate-semialdehyde dehydrogenase (acylating) activity;GO:0008152 BP: metabolic process; |
|  |  |  |  |  | TraesCS2A01G478000 | Histone-lysine N-methyltransferase | PF10440: Ubiquitin-binding WIYLD domain; PF05033: Pre-SET motif; PF00856: SET domain | IPR001214: SET domain; IPR007728: Pre-SET domain; IPR018848: WIYLD domain; IPR025776: Histone-lysine N-methyltransferase SUVR4/SUVR1/SUVR2 | GO:0005515 MF: protein binding;GO:0005634 CC: nucleus;GO:0008270 MF: zinc ion binding; |
|  |  |  |  |  | TraesCS2A01G478100 | Linalool synthase, chloroplastic | PF01397: Terpene synthase, N-terminal domain; PF03936: Terpene synthase family, metal binding domain | IPR001906: Terpene synthase, N-terminal domain; IPR005630: Terpene synthase, metal-binding domain; IPR008930: Terpenoid cyclases/protein prenyltransferase alpha-alpha toroid; IPR008949: Isoprenoid synthase domain | GO:0000287 MF: magnesium ion binding;GO:0008152 BP: metabolic process; |
|  |  |  |  |  | TraesCS2A01G478200 | NA | NA | NA | NA |
|  |  |  |  |  | TraesCS2A01G478300 | UHRF1-binding protein 1 | PF12624: N-terminal region of Chorein or VPS13 | IPR026854: Vacuolar protein sorting-associated protein 13, N-terminal domain | NA |
|  |  |  |  |  | TraesCS2A01G478400 | UDP-N-acetylmuramate--L-alanine ligase | NA | NA | NA |
|  |  |  |  |  | TraesCS2A01G478500 | Beta-glucosidase | PF00933: Glycosyl hydrolase family 3 N terminal domain; PF01915: Glycosyl hydrolase family 3 C-terminal domain | IPR001764: Glycoside hydrolase, family 3, N-terminal; IPR002772: Glycoside hydrolase family 3 C-terminal domain; IPR017853: Glycoside hydrolase superfamily; IPR019800: Glycoside hydrolase, family 3, active site | GO:0004553 MF: hydrolase activity, hydrolyzing O-glycosyl compounds;GO:0005975 BP: carbohydrate metabolic process |
|  |  |  |  |  | TraesCS2A01G478600 | Sugar transport protein 5 | NA | NA | NA |
|  |  |  |  |  | TraesCS2A01G478700 | Glycosyltransferase | PF00201: UDP-glucoronosyl and UDP-glucosyl transferase | IPR002213: UDP-glucuronosyl/UDP-glucosyltransferase | GO:0008152 BP: metabolic process;GO:0016758 MF: transferase activity, transferring hexosyl groups |
|  |  |  |  |  | TraesCS2A01G478800 | Histone-lysine N-methyltransferase | PF10440: Ubiquitin-binding WIYLD domain; PF05033: Pre-SET motif; PF00856: SET domain | IPR001214: SET domain; IPR007728: Pre-SET domain; IPR018848: WIYLD domain; IPR025776: Histone-lysine N-methyltransferase SUVR4/SUVR1/SUVR2 | GO:0005515 MF: protein binding;GO:0005634 CC: nucleus; |
|  |  |  |  |  | TraesCS2A01G478900 | Glycosyltransferase | PF00201: UDP-glucoronosyl and UDP-glucosyl transferase | IPR002213: UDP-glucuronosyl/UDP-glucosyltransferase | GO:0008152 BP: metabolic process;GO:0016758 MF: transferase activity, transferring hexosyl groups |
|  |  |  |  |  | TraesCS2A01G479000 | Phosphatidylinositol-4-phosphate 5-kinase, putative | PF02493: MORN repeat; PF01504: Phosphatidylinositol-4-phosphate 5-Kinase | IPR002498: Phosphatidylinositol-4-phosphate 5-kinase, core; IPR003409: MORN motif; IPR017163: Phosphatidylinositol-4-phosphate 5-kinase, plant; IPR027483: Phosphatidylinositol-4-phosphate 5-kinase, C-terminal; IPR027484: Phosphatidylinositol-4-phosphate 5-kinase, N-terminal domain | GO:0005524 MF: ATP binding;GO:0016307 MF: phosphatidylinositol phosphate kinase activity; |
|  |  |  |  |  | TraesCS2A01G479100 | Cyclin family protein | PF08613: Cyclin | IPR013763: Cyclin-like; IPR013922: Cyclin PHO80-like | GO:0000079 BP: regulation of cyclin-dependent protein serine/threonine kinase activity;GO:0019901 MF: protein kinase binding |
|  |  |  |  |  | TraesCS2A01G479200 | F-box family protein | NA | NA | NA |
|  |  |  |  |  | TraesCS2A01G479300 | ATP/DNA-binding protein | NA | IPR003594: Histidine kinase-like ATPase, C-terminal domain | NA |
|  |  |  |  |  | TraesCS2A01G479400 | Peroxisome biogenesis protein 22 | NA | NA | NA |
|  |  |  |  |  | TraesCS2A01G479400 | NA | NA | NA | NA |
|  |  |  |  |  | TraesCS2A01G479500 | Histone-lysine N-methyltransferase | PF00856: SET domain; PF01753: MYND finger | IPR001214: SET domain; IPR002893: Zinc finger, MYND-type | GO:0005515 MF: protein binding |
|  |  |  |  |  | TraesCS2A01G479600 | CsAtPR5 | NA | NA | NA |
|  |  |  |  |  | TraesCS2A01G479700 | Invertase/pectin methylesterase inhibitor family protein | PF04043: Plant invertase/pectin methylesterase inhibitor | IPR006501: Pectinesterase inhibitor domain | GO:0004857 MF: enzyme inhibitor activity |
|  |  |  |  |  | TraesCS2A01G479800 | CsAtPR5, putative, expressed | NA | NA | NA |
|  |  |  |  |  | TraesCS2A01G479900 | SNF2 domain-containing protein / helicase domain-containing protein / zinc finger protein-like protein | PF00176: SNF2 family N-terminal domain; PF14634: zinc-RING finger domain; PF00271: Helicase conserved C-terminal domain | IPR000330: SNF2-related, N-terminal domain; IPR001650: Helicase, C-terminal; IPR001841: Zinc finger, RING-type; IPR013083: Zinc finger, RING/FYVE/PHD-type; IPR014001: Helicase superfamily 1/2, ATP-binding domain; IPR017907: Zinc finger, RING-type, conserved site; IPR027417: P-loop containing nucleoside triphosphate hydrolase | GO:0005515 MF: protein binding;GO:0005524 MF: ATP binding; |
|  |  |  |  |  | TraesCS2A01G480000 | Phosphatidylinositol 3- and 4-kinase family protein | NA | NA | NA |
| STI_RSRatio | AX-108742509 |  | 2A | 718354502 | TraesCS2A01G480100 | NBS-LRR disease resistance protein, putative, expressed | PF00931: NB-ARC domain | IPR002182: NB-ARC; IPR011991: Winged helix-turn-helix DNA-binding domain; IPR032675: Leucine-rich repeat domain, L domain-like | GO:0043531 MF: ADP binding |
|  |  |  |  |  | TraesCS2A01G480200 | Divalent-cation tolerance protein CutA | PF03091: CutA1 divalent ion tolerance protein | IPR004323: Divalent ion tolerance protein, CutA; IPR011322: Nitrogen regulatory PII-like, alpha/beta; IPR015867: Nitrogen regulatory protein PII/ATP phosphoribosyltransferase, C-terminal | GO:0010038 BP: response to metal ion |
|  |  |  |  |  | TraesCS2A01G480300 | Thioredoxin | PF00085: Thioredoxin | IPR012336: Thioredoxin-like fold; IPR013766: Thioredoxin domain | GO:0045454 BP: cell redox homeostasis |
|  |  |  |  |  | TraesCS2A01G480400 | NBS-LRR disease resistance protein | PF00931: NB-ARC domain | IPR002182: NB-ARC; IPR011991: Winged helix-turn-helix DNA-binding domain; IPR027417: P-loop containing nucleoside triphosphate hydrolase; IPR032675: Leucine-rich repeat domain, L domain-like | GO:0043531 MF: ADP binding |
|  |  |  |  |  | TraesCS2A01G480500 | Kinesin-like protein | PF00225: Kinesin motor domain | IPR001752: Kinesin motor domain; IPR019821: Kinesin motor domain, conserved site; IPR027417: P-loop containing nucleoside triphosphate hydrolase | GO:0003777 MF: microtubule motor activity;GO:0005524 MF: ATP binding; |
|  |  |  |  |  | TraesCS2A01G480600 | Kinase-like | PF00069: Protein kinase domain | IPR000719: Protein kinase domain; IPR008271: Serine/threonine-protein kinase, active site; IPR011009: Protein kinase-like domain; IPR017441: Protein kinase, ATP binding site | GO:0004672 MF: protein kinase activity;GO:0005524 MF: ATP binding; |
|  |  |  |  |  | TraesCS2A01G480700 | Sodium- and chloride-dependent creatine transporter 1 | NA | NA | NA |
| STI_RSRatio | AX-158608713 |  | 2A | 718358893 | TraesCS2A01G480800 | Serine/threonine-protein kinase | NA | NA | NA |
|  |  |  |  |  | TraesCS2A01G480900 | Glycosyltransferase | PF04577: Protein of unknown function (DUF563) | IPR007657: Glycosyltransferase 61 | GO:0016757 MF: transferase activity, transferring glycosyl groups |
|  |  |  |  |  | TraesCS2A01G481000 | Protein SDA1-like protein | PF08158: NUC130/3NT domain; PF05285: SDA1 | IPR007949: SDA1 domain; IPR011989: Armadillo-like helical; IPR012977: Uncharacterised domain NUC130/133, N-terminal; IPR016024: Armadillo-type fold | GO:0005488 MF: binding |
| STI_RSRatio | BS00093201_51 |  | 2A | 718571577 | TraesCS2A01G481100 | At4g28290 | NA | NA | NA |
|  |  |  |  |  | TraesCS2A01G481200 | Anthocyanidin reductase | PF01370: NAD dependent epimerase/dehydratase family | IPR001509: NAD-dependent epimerase/dehydratase; IPR016040: NAD(P)-binding domain | GO:0003824 MF: catalytic activity;GO:0050662 MF: coenzyme binding |
|  |  |  |  |  | TraesCS2A01G481300 | Anthocyanidin reductase | PF01370: NAD dependent epimerase/dehydratase family | IPR001509: NAD-dependent epimerase/dehydratase; IPR016040: NAD(P)-binding domain | GO:0003824 MF: catalytic activity;GO:0050662 MF: coenzyme binding |
| STI_RSRatio | AX-110966497 |  | 2A | 718721301 | TraesCS2A01G481400 | Anthocyanidin reductase | PF01370: NAD dependent epimerase/dehydratase family | IPR001509: NAD-dependent epimerase/dehydratase; IPR016040: NAD(P)-binding domain | GO:0003824 MF: catalytic activity;GO:0050662 MF: coenzyme binding |
| STI_RSRatio | BS00081506_51 |  | 2A | 718730480 | TraesCS2A01G481500 | Anthocyanidin reductase | PF01370: NAD dependent epimerase/dehydratase family | IPR001509: NAD-dependent epimerase/dehydratase; IPR016040: NAD(P)-binding domain | GO:0003824 MF: catalytic activity;GO:0050662 MF: coenzyme binding |
|  |  |  |  |  | TraesCS2A01G481600 | NA | NA | NA | NA |
|  |  |  |  |  | TraesCS2A01G481700 | Anthocyanidin reductase | PF01370: NAD dependent epimerase/dehydratase family | IPR001509: NAD-dependent epimerase/dehydratase; IPR016040: NAD(P)-binding domain | GO:0003824 MF: catalytic activity;GO:0050662 MF: coenzyme binding |
| STI_RSRatio | AX-109340451 |  | 2A | 718934808 | TraesCS2A01G481800 | Anthocyanidin reductase | PF01370: NAD dependent epimerase/dehydratase family | IPR001509: NAD-dependent epimerase/dehydratase; IPR016040: NAD(P)-binding domain | GO:0003824 MF: catalytic activity;GO:0050662 MF: coenzyme binding |
|  |  |  |  |  | TraesCS2A01G481900 | Anthocyanidin reductase | PF01370: NAD dependent epimerase/dehydratase family | IPR001509: NAD-dependent epimerase/dehydratase; IPR016040: NAD(P)-binding domain | GO:0003824 MF: catalytic activity;GO:0050662 MF: coenzyme binding |
|  |  |  |  |  | TraesCS2A01G482000 | CASP-like protein | PF04535: Domain of unknown function (DUF588) | IPR006702: Domain of unknown function DUF588 | NA |
|  |  |  |  |  | TraesCS2A01G482100 | Anthocyanidin reductase | PF01370: NAD dependent epimerase/dehydratase family | IPR001509: NAD-dependent epimerase/dehydratase; IPR016040: NAD(P)-binding domain | GO:0003824 MF: catalytic activity;GO:0050662 MF: coenzyme binding |
|  |  |  |  |  | TraesCS2A01G482200 | Anthocyanidin reductase | PF01370: NAD dependent epimerase/dehydratase family | IPR001509: NAD-dependent epimerase/dehydratase; IPR016040: NAD(P)-binding domain | GO:0003824 MF: catalytic activity;GO:0050662 MF: coenzyme binding |
|  |  |  |  |  | TraesCS2A01G482300 | Anthocyanidin reductase | PF05368: NmrA-like family | IPR008030: NmrA-like domain; IPR016040: NAD(P)-binding domain | NA |
|  |  |  |  |  | TraesCS2A01G482400 | Mediator of RNA polymerase II transcription subunit 15a | PF16987: KIX domain | IPR003101: Coactivator CBP, KIX domain | GO:0003712 MF: transcription cofactor activity;GO:0006355 BP: regulation of transcription, DNA-templated |
|  |  |  |  |  | TraesCS2A01G482500 | NA | NA | NA | NA |
|  |  |  |  |  | TraesCS2A01G482600 | Late embryogenesis abundant protein | PF03168: Late embryogenesis abundant protein | IPR004864: Late embryogenesis abundant protein, LEA-14 | NA |
|  |  |  |  |  | TraesCS2A01G482700 | tRNA (Guanine(10)-N2)-methyltransferase-like protein | PF01170: Putative RNA methylase family UPF0020 | IPR000241: Putative RNA methylase domain; IPR002052: DNA methylase, N-6 adenine-specific, conserved site; IPR016691: tRNA guanosine-2'-O-methyltransferase, TRM11; IPR029063: S-adenosyl-L-methionine-dependent methyltransferase | GO:0003676 MF: nucleic acid binding;GO:0008168 MF: methyltransferase activity;GO:0032259 BP: methylation |
|  |  |  |  |  | TraesCS2A01G482800 | Harpin-induced protein | PF03168: Late embryogenesis abundant protein | IPR004864: Late embryogenesis abundant protein, LEA-14 | NA |
|  |  |  |  |  | TraesCS2A01G482900 | alpha-1,3-mannosyl-glycoprotein beta-1,2-N-acetylglucosaminyltransferase | NA | NA | NA |
|  |  |  |  |  | TraesCS2A01G483000 | Glycosyltransferase | PF00201: UDP-glucoronosyl and UDP-glucosyl transferase | IPR002213: UDP-glucuronosyl/UDP-glucosyltransferase | GO:0008152 BP: metabolic process;GO:0016758 MF: transferase activity, transferring hexosyl groups |
|  |  |  |  |  | TraesCS2A01G483100 | Anthocyanidin reductase | PF01370: NAD dependent epimerase/dehydratase family | IPR001509: NAD-dependent epimerase/dehydratase; IPR016040: NAD(P)-binding domain | GO:0003824 MF: catalytic activity;GO:0050662 MF: coenzyme binding |
|  |  |  |  |  | TraesCS2A01G483200 | DNA topoisomerase 3-alpha | NA | NA | NA |
|  |  |  |  |  | TraesCS2A01G483300 | Pleiotropic drug resistance ABC transporter | PF14510: ABC-transporter extracellular N-terminal; PF00005: ABC transporter; PF01061: ABC-2 type transporter; PF08370: Plant PDR ABC transporter associated | IPR003439: ABC transporter-like; IPR003593: AAA+ ATPase domain; IPR013525: ABC-2 type transporter; IPR013581: Plant PDR ABC transporter associated; IPR027417: P-loop containing nucleoside triphosphate hydrolase; IPR029481: ABC-transporter extracellular N-terminal domain | GO:0005524 MF: ATP binding;GO:0016020 CC: membrane; |
|  |  |  |  |  | TraesCS2A01G483400 | Organic cation transporter-like protein | PF00083: Sugar (and other) transporter | IPR005828: Major facilitator, sugar transporter-like; IPR005829: Sugar transporter, conserved site; IPR020846: Major facilitator superfamily domain | GO:0005215 MF: transporter activity;GO:0016021 CC: integral component of membrane; |
|  |  |  |  |  | TraesCS2A01G483500 | Organic cation transporter protein | PF00083: Sugar (and other) transporter | IPR005828: Major facilitator, sugar transporter-like; IPR005829: Sugar transporter, conserved site; IPR020846: Major facilitator superfamily domain | GO:0005215 MF: transporter activity;GO:0016021 CC: integral component of membrane; |
|  |  |  |  |  | TraesCS2A01G483600 | Elongation factor 1-alpha | NA | NA | NA |
|  |  |  |  |  | TraesCS2A01G483700 | Organic cation transporter protein | PF00083: Sugar (and other) transporter | IPR005828: Major facilitator, sugar transporter-like; IPR005829: Sugar transporter, conserved site; IPR020846: Major facilitator superfamily domain | GO:0005215 MF: transporter activity;GO:0016021 CC: integral component of membrane; |
|  |  |  |  |  | TraesCS2A01G483800 | NA | NA | NA | NA |
|  |  |  |  |  | TraesCS2A01G483900 | Organic cation transporter protein | PF00083: Sugar (and other) transporter | IPR005828: Major facilitator, sugar transporter-like; IPR005829: Sugar transporter, conserved site; IPR020846: Major facilitator superfamily domain | GO:0005215 MF: transporter activity;GO:0016021 CC: integral component of membrane; |
|  |  |  |  |  | TraesCS2A01G484000 | Organic cation transporter protein | PF00083: Sugar (and other) transporter | IPR005828: Major facilitator, sugar transporter-like; IPR005829: Sugar transporter, conserved site; IPR020846: Major facilitator superfamily domain | GO:0005215 MF: transporter activity;GO:0016021 CC: integral component of membrane; |
|  |  |  |  |  | TraesCS2A01G484100 | Xyloglucan endotransglucosylase/hydrolase | PF00722: Glycosyl hydrolases family 16 | IPR000757: Glycoside hydrolase family 16; IPR013320: Concanavalin A-like lectin/glucanase domain | GO:0004553 MF: hydrolase activity, hydrolyzing O-glycosyl compounds;GO:0005975 BP: carbohydrate metabolic process |
|  |  |  |  |  | TraesCS2A01G484200 | Yellow stripe-like transporter 12 | PF03169: OPT oligopeptide transporter protein | IPR004813: Oligopeptide transporter, OPT superfamily | GO:0055085 BP: transmembrane transport |
|  |  |  |  |  | TraesCS2A01G484300 | UDP-N-acetylglucosamine 1-carboxyvinyltransferase | NA | NA | NA |
|  |  |  |  |  | TraesCS2A01G484400 | cytochrome P450, family 71, subfamily A, polypeptide 14 | NA | NA | NA |
|  |  |  |  |  | TraesCS2A01G484500 | Pollen Ole e 1 allergen and extensin family protein, putative | NA | NA | NA |
|  |  |  |  |  | TraesCS2A01G484600 | Pollen Ole e 1 allergen and extensin family protein, putative | NA | NA | NA |
|  |  |  |  |  | TraesCS2A01G484700 | basic helix-loop-helix (bHLH) DNA-binding superfamily protein | NA | IPR011598: Myc-type, basic helix-loop-helix (bHLH) domain | GO:0046983 MF: protein dimerization activity |
|  |  |  |  |  | TraesCS2A01G484800 | Serine/threonine-protein kinase | PF01453: D-mannose binding lectin; PF00954: S-locus glycoprotein domain; PF08276: PAN-like domain; PF07714: Protein tyrosine kinase | IPR000719: Protein kinase domain; IPR000858: S-locus glycoprotein domain; IPR001245: Serine-threonine/tyrosine-protein kinase, catalytic domain; IPR001480: Bulb-type lectin domain; IPR003609: PAN/Apple domain; IPR011009: Protein kinase-like domain; IPR017441: Protein kinase, ATP binding site | GO:0004672 MF: protein kinase activity;GO:0005524 MF: ATP binding; |
|  |  |  |  |  | TraesCS2A01G484900 | Serine/threonine-protein kinase | PF07714: Protein tyrosine kinase | IPR000719: Protein kinase domain; IPR001245: Serine-threonine/tyrosine-protein kinase, catalytic domain; IPR008271: Serine/threonine-protein kinase, active site; IPR011009: Protein kinase-like domain | GO:0004672 MF: protein kinase activity;GO:0005524 MF: ATP binding; |
| STI_RL | wsnp_JD_c52_87219 | sti_RL_2B_Hap1 | 2B | 640688855 | TraesCS2B01G447700 | alpha/beta-Hydrolases superfamily protein | NA | NA | NA |
|  | Excalibur_c11392_1193 | sti_RL_2B_Hap1 | 2B | 641943134 | TraesCS2B01G447800 | Late embryogenesis abundant protein family protein | NA | NA | NA |
|  |  |  |  |  | TraesCS2B01G447900 | Yellow stripe-like transporter 17 | PF03169: OPT oligopeptide transporter protein | IPR004813: Oligopeptide transporter, OPT superfamily | GO:0055085 BP: transmembrane transport |
|  |  |  |  |  | TraesCS2B01G448000 | Methionine aminopeptidase | PF00557: Metallopeptidase family M24 | IPR000994: Peptidase M24; IPR001714: Peptidase M24, methionine aminopeptidase; IPR002467: Peptidase M24A, methionine aminopeptidase, subfamily 1 | GO:0004177 MF: aminopeptidase activity;GO:0006508 BP: proteolysis; |
|  |  |  |  |  | TraesCS2B01G448100 | Ethylene-responsive transcription factor | PF00847: AP2 domain | IPR001471: AP2/ERF domain; IPR016177: DNA-binding domain | GO:0003677 MF: DNA binding;GO:0003700 MF: transcription factor activity, sequence-specific DNA binding;GO:0006355 BP: regulation of transcription, DNA-templated |
|  |  |  |  |  | TraesCS2B01G448300 | Receptor-like kinase | PF00069: Protein kinase domain | IPR000719: Protein kinase domain; IPR008271: Serine/threonine-protein kinase, active site; IPR011009: Protein kinase-like domain | GO:0004672 MF: protein kinase activity;GO:0005524 MF: ATP binding; |
|  |  |  |  |  | TraesCS2B01G448400 | Heat-inducible transcription repressor HrcA | PF03004: Plant transposase (Ptta/En/Spm family) | IPR004252: Probable transposase, Ptta/En/Spm, plant | NA |
|  |  |  |  |  | TraesCS2B01G448500 | Protein strawberry notch-like protein 1 | PF03004: Plant transposase (Ptta/En/Spm family) | IPR004252: Probable transposase, Ptta/En/Spm, plant | NA |
|  |  |  |  |  | TraesCS2B01G448600 | Protein strawberry notch-like protein 1 | NA | NA | NA |
|  |  |  |  |  | TraesCS2B01G448700 | Cytochrome P450 | PF00067: Cytochrome P450 | IPR001128: Cytochrome P450; IPR002401: Cytochrome P450, E-class, group I; IPR017972: Cytochrome P450, conserved site | GO:0005506 MF: iron ion binding;GO:0016705 MF: oxidoreductase activity, acting on paired donors, with incorporation or reduction of molecular oxygen; |
|  |  |  |  |  | TraesCS2B01G448800 | Glycosyl transferase family 2 protein | NA | NA | NA |
|  |  |  |  |  | TraesCS2B01G448900 | piezo-type mechanosensitive ion channel component | NA | NA | NA |
|  |  |  |  |  | TraesCS2B01G449000 | 30S ribosomal protein S8 | PF00410: Ribosomal protein S8 | IPR000630: Ribosomal protein S8 | GO:0003735 MF: structural constituent of ribosome;GO:0005840 CC: ribosome; |
|  |  |  |  |  | TraesCS2B01G449100 | Transporter-related family protein | PF00083: Sugar (and other) transporter | IPR005828: Major facilitator, sugar transporter-like; IPR005829: Sugar transporter, conserved site; IPR020846: Major facilitator superfamily domain | GO:0005215 MF: transporter activity;GO:0016021 CC: integral component of membrane; |
|  |  |  |  |  | TraesCS2B01G449200 | Protein phosphatase 2c, putative | PF00481: Protein phosphatase 2C | IPR001932: PPM-type phosphatase domain | GO:0003824 MF: catalytic activity |
|  |  |  |  |  | TraesCS2B01G449300 | Pumilio-like protein | PF00806: Pumilio-family RNA binding repeat | IPR001313: Pumilio RNA-binding repeat; IPR011989: Armadillo-like helical; IPR016024: Armadillo-type fold; IPR033133: Pumilio homology domain | GO:0003723 MF: RNA binding;GO:0005488 MF: binding |
|  |  |  |  |  | TraesCS2B01G449400 | Pumilio-like protein | PF00806: Pumilio-family RNA binding repeat | IPR001313: Pumilio RNA-binding repeat; IPR011989: Armadillo-like helical; IPR016024: Armadillo-type fold; IPR033133: Pumilio homology domain | GO:0003723 MF: RNA binding;GO:0005488 MF: binding |
|  |  |  |  |  | TraesCS2B01G449500 | HXXXD-type acyl-transferase family protein, putative | PF02458: Transferase family | IPR003480: Transferase | GO:0016747 MF: transferase activity, transferring acyl groups other than amino-acyl groups |
|  |  |  |  |  | TraesCS2B01G449600 | HXXXD-type acyl-transferase family protein | PF02458: Transferase family | IPR003480: Transferase | GO:0016747 MF: transferase activity, transferring acyl groups other than amino-acyl groups |
|  |  |  |  |  | TraesCS2B01G449700 | HXXXD-type acyl-transferase family protein, putative | PF02458: Transferase family | IPR003480: Transferase | GO:0016747 MF: transferase activity, transferring acyl groups other than amino-acyl groups |
|  |  |  |  |  | TraesCS2B01G449800 | B3 domain-containing protein family | PF02362: B3 DNA binding domain | IPR003340: B3 DNA binding domain; IPR015300: DNA-binding pseudobarrel domain | GO:0003677 MF: DNA binding |
|  |  |  |  |  | TraesCS2B01G449900 | Serine/threonine-protein kinase | PF01453: D-mannose binding lectin; PF00069: Protein kinase domain | IPR000719: Protein kinase domain; IPR001480: Bulb-type lectin domain; IPR008271: Serine/threonine-protein kinase, active site; IPR011009: Protein kinase-like domain; IPR024171: S-receptor-like serine/threonine-protein kinase | GO:0004672 MF: protein kinase activity;GO:0004674 MF: protein serine/threonine kinase activity; |
|  |  |  |  |  | TraesCS2B01G450000 | Kaurene synthase | PF01397: Terpene synthase, N-terminal domain; PF03936: Terpene synthase family, metal binding domain | IPR001906: Terpene synthase, N-terminal domain; IPR005630: Terpene synthase, metal-binding domain; IPR008930: Terpenoid cyclases/protein prenyltransferase alpha-alpha toroid; IPR008949: Isoprenoid synthase domain | GO:0000287 MF: magnesium ion binding;GO:0008152 BP: metabolic process; |
|  |  |  |  |  | TraesCS2B01G450100 | Cytochrome P450 | PF00067: Cytochrome P450 | IPR001128: Cytochrome P450; IPR002401: Cytochrome P450, E-class, group I; IPR017972: Cytochrome P450, conserved site | GO:0005506 MF: iron ion binding;GO:0016705 MF: oxidoreductase activity, acting on paired donors, with incorporation or reduction of molecular oxygen;GO:0020037 MF: heme binding;GO:0055114 BP: oxidation-reduction process |
|  |  |  |  |  | TraesCS2B01G450200 | Cytochrome P450 | PF00067: Cytochrome P450 | IPR001128: Cytochrome P450; IPR002401: Cytochrome P450, E-class, group I; IPR017972: Cytochrome P450, conserved site | GO:0005506 MF: iron ion binding;GO:0016705 MF: oxidoreductase activity, acting on paired donors, with incorporation or reduction of molecular oxygen; |
|  |  |  |  |  | TraesCS2B01G450300 | NA | NA | NA | NA |
|  |  |  |  |  | TraesCS2B01G450400 | NA | NA | NA | NA |
|  |  |  |  |  | TraesCS2B01G450500 | Transcriptional factor B3 family protein | NA | IPR015300: DNA-binding pseudobarrel domain | NA |
|  |  |  |  |  | TraesCS2B01G450600 | Transcriptional factor B3 family protein | NA | IPR015300: DNA-binding pseudobarrel domain | NA |
|  |  |  |  |  | TraesCS2B01G450700 | Pre-mRNA-splicing factor CWC21 | PF08312: cwf21 domain | IPR013170: mRNA splicing factor Cwf21 domain | NA |
|  |  |  |  |  | TraesCS2B01G450800 | 26S proteasome non-ATPase regulatory subunit 1 | PF01851: Proteasome/cyclosome repeat; PF13646: HEAT repeats | IPR002015: Proteasome/cyclosome repeat; IPR011989: Armadillo-like helical; IPR016024: Armadillo-type fold; IPR016642: 26S proteasome regulatory complex, non-ATPase subcomplex, Rpn2/Psmd1 subunit | GO:0000502 CC: proteasome complex;GO:0005488 MF: binding; |
| STI_RSRatio | Kukri_c51247_322 |  | 3A | 140043493 | TraesCS3A01G151100 | Peptidyl-prolyl cis-trans isomerase | PF00160: Cyclophilin type peptidyl-prolyl cis-trans isomerase/CLD | IPR002130: Cyclophilin-type peptidyl-prolyl cis-trans isomerase domain; IPR020892: Cyclophilin-type peptidyl-prolyl cis-trans isomerase, conserved site; IPR029000: Cyclophilin-like domain | GO:0000413 BP: protein peptidyl-prolyl isomerization;GO:0003755 MF: peptidyl-prolyl cis-trans isomerase activity; |
|  |  |  |  |  | TraesCS3A01G151200 | Reticulon-like protein | PF02453: Reticulon | IPR003388: Reticulon | NA |
|  |  |  |  |  | TraesCS3A01G151300 | ATP-dependent zinc metalloprotease FtsH 1 | PF00004: ATPase family associated with various cellular activities (AAA) | IPR003593: AAA+ ATPase domain; IPR003959: ATPase, AAA-type, core; IPR003960: ATPase, AAA-type, conserved site; IPR027417: P-loop containing nucleoside triphosphate hydrolase | GO:0005524 MF: ATP binding |
|  |  |  |  |  | TraesCS3A01G151400 | S-type anion channel | PF03595: Voltage-dependent anion channel | IPR004695: Voltage-dependent anion channel | GO:0016021 CC: integral component of membrane;GO:0055085 BP: transmembrane transport |
|  |  |  |  |  | TraesCS3A01G151500 | F-box family protein | NA | IPR001810: F-box domain | GO:0005515 MF: protein binding |
|  |  |  |  |  | TraesCS3A01G151600 | 60S ribosomal protein L51, mitochondrial | PF05047: Mitochondrial ribosomal protein L51 / S25 / CI-B8 domain | IPR007741: Ribosomal protein/NADH dehydrogenase domain; IPR012336: Thioredoxin-like fold | NA |
| STI_SL | AX-108852904 |  | 3B | 822891332 | TraesCS3B01G601500 | 11S globulin seed storage protein 2 | PF00190: Cupin | IPR006044: 11-S seed storage protein, plant; IPR006045: Cupin 1; IPR011051: RmlC-like cupin domain; IPR014710: RmlC-like jelly roll fold | GO:0045735 MF: nutrient reservoir activity |
|  |  |  |  |  | TraesCS3B01G601600 | Metallothionein | PF01439: Metallothionein | IPR000347: Metallothionein, family 15, plant | GO:0046872 MF: metal ion binding |
|  |  |  |  |  | TraesCS3B01G601700 | Metallothionein | PF01439: Metallothionein | IPR000347: Metallothionein, family 15, plant | GO:0046872 MF: metal ion binding |
|  |  |  |  |  | TraesCS3B01G601800 | Metallothionein | PF01439: Metallothionein | IPR000347: Metallothionein, family 15, plant | GO:0046872 MF: metal ion binding |
|  |  |  |  |  | TraesCS3B01G601900 | Plant invertase/pectin methylesterase inhibitor superfamily protein | NA | NA | NA |
|  |  |  |  |  | TraesCS3B01G602000 | 30S ribosomal protein S17 | PF00366: Ribosomal protein S17 | IPR000266: Ribosomal protein S17/S11; IPR012340: Nucleic acid-binding, OB-fold; IPR019984: 30S ribosomal protein S17 | GO:0003735 MF: structural constituent of ribosome;GO:0005622 CC: intracellular; |
|  |  |  |  |  | TraesCS3B01G602100 | 30S ribosomal protein S19 | PF00203: Ribosomal protein S19 | IPR002222: Ribosomal protein S19/S15; IPR005732: Ribosomal protein S19, bacterial-type; IPR020934: Ribosomal protein S19 conserved site; IPR023575: Ribosomal protein S19, superfamily | GO:0003723 MF: RNA binding;GO:0003735 MF: structural constituent of ribosome;GO:0005840 CC: ribosome; |
|  |  |  |  |  | TraesCS3B01G602200 | SANT domain-containing protein 2 | NA | NA | NA |
|  |  |  |  |  | TraesCS3B01G602300 | Mitochondrial ATP synthase 6 kDa subunit | NA | NA | NA |
|  |  |  |  |  | TraesCS3B01G602400 | PR5-like receptor kinase | PF00069: Protein kinase domain | IPR000719: Protein kinase domain; IPR001680: WD40 repeat; IPR008271: Serine/threonine-protein kinase, active site; IPR008962: PapD-like; IPR011009: Protein kinase-like domain; IPR015943: WD40/YVTN repeat-like-containing domain; IPR017441: Protein kinase, ATP binding site; IPR017986: WD40-repeat-containing domain; IPR019775: WD40 repeat, conserved site | GO:0004672 MF: protein kinase activity;GO:0005515 MF: protein binding; |
|  |  |  |  |  | TraesCS3B01G602500 | Lipoxygenase | PF01477: PLAT/LH2 domain; PF00305: Lipoxygenase | IPR001024: PLAT/LH2 domain; IPR001246: Lipoxygenase, plant; IPR013819: Lipoxygenase, C-terminal; IPR020833: Lipoxygenase, iron binding site; IPR020834: Lipoxygenase, conserved site; IPR027433: Lipoxygenase, domain 3 | GO:0005515 MF: protein binding;GO:0016491 MF: oxidoreductase activity; |
|  |  |  |  |  | TraesCS3B01G602600 | Extra-large guanine nucleotide binding family protein | NA | IPR013083: Zinc finger, RING/FYVE/PHD-type | NA |
|  |  |  |  |  | TraesCS3B01G602700 | Protein kinase-like protein | PF08263: Leucine rich repeat N-terminal domain; PF13855: Leucine rich repeat; PF00069: Protein kinase domain | IPR000719: Protein kinase domain; IPR001611: Leucine-rich repeat; IPR003591: Leucine-rich repeat, typical subtype; IPR008271: Serine/threonine-protein kinase, active site; IPR011009: Protein kinase-like domain; IPR013210: Leucine-rich repeat-containing N-terminal, plant-type; IPR017441: Protein kinase, ATP binding site; IPR032675: Leucine-rich repeat domain, L domain-like | GO:0004672 MF: protein kinase activity;GO:0005515 MF: protein binding; |
|  |  |  |  |  | TraesCS3B01G602800 | Plant cadmium resistance protein | PF11204: Protein of unknown function (DUF2985); PF04749: PLAC8 family | IPR006461: PLAC8 motif-containing protein; IPR015943: WD40/YVTN repeat-like-containing domain; IPR021369: Protein of unknown function DUF2985 | GO:0005515 MF: protein binding |
|  |  |  |  |  | TraesCS3B01G602900 | Dirigent protein | PF03018: Dirigent-like protein | IPR004265: Plant disease resistance response protein | NA |
|  |  |  |  |  | TraesCS3B01G603000 | Gibberellin 2-beta-dioxygenase | PF14226: non-haem dioxygenase in morphine synthesis N-terminal; PF03171: 2OG-Fe(II) oxygenase superfamily | IPR005123: Oxoglutarate/iron-dependent dioxygenase; IPR026992: Non-haem dioxygenase N-terminal domain; IPR027443: Isopenicillin N synthase-like | GO:0016491 MF: oxidoreductase activity;GO:0055114 BP: oxidation-reduction process |
|  |  |  |  |  | TraesCS3B01G603100 | Chaperone protein dnaJ, putative | PF13432: Tetratricopeptide repeat; PF13181: Tetratricopeptide repeat; PF00226: DnaJ domain | IPR001623: DnaJ domain; IPR011990: Tetratricopeptide-like helical domain; IPR013026: Tetratricopeptide repeat-containing domain; IPR018253: DnaJ domain, conserved site; IPR019734: Tetratricopeptide repeat | GO:0005515 MF: protein binding |
|  |  |  |  |  | TraesCS3B01G603200 | SKP1-like protein | PF01466: Skp1 family, dimerisation domain | IPR001232: S-phase kinase-associated protein 1-like; IPR011333: SKP1/BTB/POZ domain; IPR016072: SKP1 component, dimerisation | GO:0006511 BP: ubiquitin-dependent protein catabolic process |
|  |  |  |  |  | TraesCS3B01G603300 | Kinase family protein | PF07714: Protein tyrosine kinase | IPR000719: Protein kinase domain; IPR001245: Serine-threonine/tyrosine-protein kinase, catalytic domain; IPR008271: Serine/threonine-protein kinase, active site; IPR011009: Protein kinase-like domain; IPR017441: Protein kinase, ATP binding site | GO:0004672 MF: protein kinase activity;GO:0005524 MF: ATP binding; |
|  |  |  |  |  | TraesCS3B01G603400 | E3 SUMO-protein ligase SIZ1 | PF02891: MIZ/SP-RING zinc finger | IPR001965: Zinc finger, PHD-type; IPR003034: SAP domain; IPR004181: Zinc finger, MIZ-type; IPR011011: Zinc finger, FYVE/PHD-type; IPR013083: Zinc finger, RING/FYVE/PHD-type; IPR019786: Zinc finger, PHD-type, conserved site | GO:0005515 MF: protein binding;GO:0008270 MF: zinc ion binding |
|  |  |  |  |  | TraesCS3B01G603500 | Protein IQ-DOMAIN 1 | PF00612: IQ calmodulin-binding motif; PF13178: Protein of unknown function (DUF4005) | IPR000048: IQ motif, EF-hand binding site; IPR025064: Domain of unknown function DUF4005 | GO:0005515 MF: protein binding |
|  |  |  |  |  | TraesCS3B01G603600 | Serine/threonine-protein kinase ATM | PF00855: PWWP domain | IPR000313: PWWP domain | NA |
|  |  |  |  |  | TraesCS3B01G603700 | Serine/threonine-protein kinase ATM | NA | IPR000504: RNA recognition motif domain | GO:0003676 MF: nucleic acid binding |
|  |  |  |  |  | TraesCS3B01G603800 | Kinase-like protein | PF12819: Carbohydrate-binding protein of the ER; PF07714: Protein tyrosine kinase | IPR000719: Protein kinase domain; IPR001245: Serine-threonine/tyrosine-protein kinase, catalytic domain; IPR008271: Serine/threonine-protein kinase, active site; IPR011009: Protein kinase-like domain; IPR017441: Protein kinase, ATP binding site; IPR024788: Malectin-like carbohydrate-binding domain; IPR032675: Leucine-rich repeat domain, L domain-like | GO:0004672 MF: protein kinase activity;GO:0005524 MF: ATP binding;GO:0006468 BP: protein phosphorylation |
|  |  |  |  |  | TraesCS3B01G603900 | GATA transcription factor, putative | PF00320: GATA zinc finger | IPR000679: Zinc finger, GATA-type; IPR013088: Zinc finger, NHR/GATA-type | GO:0003700 MF: transcription factor activity, sequence-specific DNA binding;GO:0006355 BP: regulation of transcription, DNA-templated;GO:0008270 MF: zinc ion binding; |
|  |  |  |  |  | TraesCS3B01G604000 | Protein phosphatase 2C family protein | PF00481: Protein phosphatase 2C | IPR000222: PPM-type phosphatase, divalent cation binding; IPR001932: PPM-type phosphatase domain | GO:0003824 MF: catalytic activity;GO:0043169 MF: cation binding |
|  |  |  |  |  | TraesCS3B01G604100 | NBS-LRR disease resistance protein | PF00931: NB-ARC domain | IPR002182: NB-ARC; IPR011991: Winged helix-turn-helix DNA-binding domain; IPR027417: P-loop containing nucleoside triphosphate hydrolase; IPR032675: Leucine-rich repeat domain, L domain-like | GO:0043531 MF: ADP binding |
|  |  |  |  |  | TraesCS3B01G604200 | Ubiquitin carboxyl-terminal hydrolase 2 | PF13968: Domain of unknown function (DUF4220) | IPR025315: Domain of unknown function DUF4220 | NA |
|  |  |  |  |  | TraesCS3B01G604300 | rRNA N-glycosidase | PF00161: Ribosome inactivating protein | IPR001574: Ribosome-inactivating protein; IPR016138: Ribosome-inactivating protein, subdomain 1 | GO:0017148 BP: negative regulation of translation;GO:0030598 MF: rRNA N-glycosylase activity |
|  |  |  |  |  | TraesCS3B01G604400 | rRNA N-glycosidase | NA | NA | NA |
|  |  |  |  |  | TraesCS3B01G604500 | NBS-LRR resistance-like protein | PF00931: NB-ARC domain | IPR002182: NB-ARC; IPR011991: Winged helix-turn-helix DNA-binding domain; IPR027417: P-loop containing nucleoside triphosphate hydrolase; IPR032675: Leucine-rich repeat domain, L domain-like | GO:0043531 MF: ADP binding |
|  |  |  |  |  | TraesCS3B01G604600 | Disease resistance protein (NBS-LRR class) family | PF00931: NB-ARC domain | IPR002182: NB-ARC; IPR027417: P-loop containing nucleoside triphosphate hydrolase | GO:0043531 MF: ADP binding |
| STI_SL | BS00071183_51 |  | 3B | 823762843 | TraesCS3B01G604700 | External alternative NAD(P)H-ubiquinone oxidoreductase B2, mitochondrial | PF07992: Pyridine nucleotide-disulphide oxidoreductase | IPR023753: FAD/NAD(P)-binding domain | GO:0016491 MF: oxidoreductase activity;GO:0055114 BP: oxidation-reduction process |
|  |  |  |  |  | TraesCS3B01G604800 | NBS-LRR-like resistance protein | PF00931: NB-ARC domain | IPR002182: NB-ARC; IPR011991: Winged helix-turn-helix DNA-binding domain; IPR027417: P-loop containing nucleoside triphosphate hydrolase; IPR032675: Leucine-rich repeat domain, L domain-like | GO:0043531 MF: ADP binding |
|  |  |  |  |  | TraesCS3B01G604900 | Disease resistance protein (NBS-LRR class) family | PF00931: NB-ARC domain; PF00161: Ribosome inactivating protein | IPR001574: Ribosome-inactivating protein; IPR002182: NB-ARC; IPR011991: Winged helix-turn-helix DNA-binding domain; IPR016138: Ribosome-inactivating protein, subdomain 1; IPR027417: P-loop containing nucleoside triphosphate hydrolase; IPR032675: Leucine-rich repeat domain, L domain-like | GO:0017148 BP: negative regulation of translation;GO:0030598 MF: rRNA N-glycosylase activity;GO:0043531 MF: ADP binding |
|  |  |  |  |  | TraesCS3B01G605000 | transmembrane protein, putative (DUF594) | PF13968: Domain of unknown function (DUF4220); PF04578: Protein of unknown function, DUF594 | IPR007658: Protein of unknown function DUF594; IPR025315: Domain of unknown function DUF4220 | NA |
|  |  |  |  |  | TraesCS3B01G605100 | Kinase interacting (KIP1-like) family protein | PF00856: SET domain; PF09273: Rubisco LSMT substrate-binding; PF07765: KIP1-like protein | IPR001214: SET domain; IPR011684: Protein Networked (NET), actin-binding (NAB) domain; IPR015353: Rubisco LSMT, substrate-binding domain | GO:0003779 MF: actin binding;GO:0005515 MF: protein binding |
|  |  |  |  |  | TraesCS3B01G605200 | Transmembrane protein, putative | PF06749: Protein of unknown function (DUF1218) | IPR009606: Protein of unknown function DUF1218 | NA |
|  |  |  |  |  | TraesCS3B01G605300 | ubiquitin carboxyl-terminal hydrolase-like protein, putative (DUF627 and DUF629) | NA | NA | NA |
|  |  |  |  |  | TraesCS3B01G605400 | Divalent ion symporter | NA | NA | NA |
|  |  |  |  |  | TraesCS3B01G605500 | Cortactin-binding protein 2 | NA | NA | NA |
|  |  |  |  |  | TraesCS3B01G605600 | AGAP002737-PA | NA | NA | NA |
|  |  |  |  |  | TraesCS3B01G605700 | Lipoxygenase | PF01477: PLAT/LH2 domain; PF00305: Lipoxygenase | IPR001024: PLAT/LH2 domain; IPR001246: Lipoxygenase, plant; IPR013819: Lipoxygenase, C-terminal; IPR020833: Lipoxygenase, iron binding site; IPR020834: Lipoxygenase, conserved site; IPR027433: Lipoxygenase, domain 3 | GO:0005515 MF: protein binding;GO:0016491 MF: oxidoreductase activity;G |
|  |  |  |  |  | TraesCS3B01G605800 | plant/protein (Protein of unknown function, DUF538) | PF04398: Protein of unknown function, DUF538 | IPR007493: Protein of unknown function DUF538 | NA |
|  |  |  |  |  | TraesCS3B01G605900 | F-box protein | NA | NA | NA |
| STI_SL | IAAV8659 |  | 3B | 826081626 | TraesCS3B01G606000 | F-box protein | PF03478: Protein of unknown function (DUF295) | IPR005174: Domain unknown function DUF295 | NA |
|  |  |  |  |  | TraesCS3B01G606100 | AGAP002737-PA | NA | NA | NA |
|  |  |  |  |  | TraesCS3B01G606200 | AGAP002737-PA | NA | NA | NA |
|  |  |  |  |  | TraesCS3B01G606300 | CAP-gly domain linker | NA | NA | NA |
|  |  |  |  |  | TraesCS3B01G606400 | Aspartic proteinase nepenthesin-1 | PF14543: Xylanase inhibitor N-terminal; PF14541: Xylanase inhibitor C-terminal | IPR001461: Aspartic peptidase A1 family; IPR021109: Aspartic peptidase domain; IPR032799: Xylanase inhibitor, C-terminal; IPR032861: Xylanase inhibitor, N-terminal; IPR033121: Peptidase family A1 domain | GO:0004190 MF: aspartic-type endopeptidase activity;GO:0006508 BP: proteolysis |
|  |  |  |  |  | TraesCS3B01G606500 | plant/protein (Protein of unknown function, DUF538) | PF04398: Protein of unknown function, DUF538 | IPR007493: Protein of unknown function DUF538 | NA |
|  |  |  |  |  | TraesCS3B01G606600 | NA | NA | NA | NA |
|  |  |  |  |  | TraesCS3B01G606700 | Disease resistance protein (TIR-NBS-LRR class) family | PF00931: NB-ARC domain | IPR002182: NB-ARC; IPR011991: Winged helix-turn-helix DNA-binding domain; IPR027417: P-loop containing nucleoside triphosphate hydrolase; IPR032675: Leucine-rich repeat domain, L domain-like | GO:0043531 MF: ADP binding |
|  |  |  |  |  | TraesCS3B01G606800 | rRNA N-glycosidase | PF00161: Ribosome inactivating protein | IPR001574: Ribosome-inactivating protein; IPR016138: Ribosome-inactivating protein, subdomain 1 | GO:0017148 BP: negative regulation of translation;GO:0030598 MF: rRNA N-glycosylase activity |
|  |  |  |  |  | TraesCS3B01G606900 | Epoxide hydrolase 2 | PF12697: Alpha/beta hydrolase family | IPR000073: Alpha/beta hydrolase fold-1; IPR000639: Epoxide hydrolase-like; IPR029058: Alpha/Beta hydrolase fold | GO:0003824 MF: catalytic activity |
|  |  |  |  |  | TraesCS3B01G607000 | Aspartic proteinase nepenthesin-1 | PF14543: Xylanase inhibitor N-terminal; PF14541: Xylanase inhibitor C-terminal | IPR001461: Aspartic peptidase A1 family; IPR001969: Aspartic peptidase, active site; IPR021109: Aspartic peptidase domain; IPR032799: Xylanase inhibitor, C-terminal; IPR032861: Xylanase inhibitor, N-terminal; IPR033121: Peptidase family A1 domain | GO:0004190 MF: aspartic-type endopeptidase activity;GO:0006508 BP: proteolysis |
|  |  |  |  |  | TraesCS3B01G607100 | Aspartic proteinase nepenthesin-1 | PF14543: Xylanase inhibitor N-terminal; PF14541: Xylanase inhibitor C-terminal | IPR001461: Aspartic peptidase A1 family; IPR001969: Aspartic peptidase, active site; IPR021109: Aspartic peptidase domain; IPR032799: Xylanase inhibitor, C-terminal; IPR032861: Xylanase inhibitor, N-terminal; IPR033121: Peptidase family A1 domain | GO:0004190 MF: aspartic-type endopeptidase activity;GO:0006508 BP: proteolysis |
|  |  |  |  |  | TraesCS3B01G607200 | SNARE-interacting protein KEULE | PF00995: Sec1 family | IPR001619: Sec1-like protein | GO:0006904 BP: vesicle docking involved in exocytosis;GO:0016192 BP: vesicle-mediated transport |
|  |  |  |  |  | TraesCS3B01G607300 | Pectinesterase inhibitor | PF04043: Plant invertase/pectin methylesterase inhibitor | IPR006501: Pectinesterase inhibitor domain | GO:0004857 MF: enzyme inhibitor activity |
|  |  |  |  |  | TraesCS3B01G607400 | Plant/T31B5-30 protein | PF11443: Domain of unknown function (DUF2828) | IPR000878: Tetrapyrrole methylase; IPR024553: Domain of unknown function DUF2828 | GO:0008152 BP: metabolic process;GO:0008168 MF: methyltransferase activity |
| STI_SL | wsnp_Ra_rep_c75740_73183118 |  | 3B | 826081626 | TraesCS3B01G607500 | Chaperone protein dnaJ | PF00226: DnaJ domain; PF11926: Domain of unknown function (DUF3444) | IPR001623: DnaJ domain; IPR024593: Domain of unknown function DUF3444 | NA |
| STI_SL | AX-158598301 |  | 3B | 826091387 | TraesCS3B01G607500 | Chaperone protein dnaJ | PF00226: DnaJ domain; PF11926: Domain of unknown function (DUF3444) | IPR001623: DnaJ domain; IPR024593: Domain of unknown function DUF3444 | NA |
|  |  |  |  |  | TraesCS3B01G607600 | ABC transporter B family protein | PF00664: ABC transporter transmembrane region; PF00005: ABC transporter | IPR003439: ABC transporter-like; IPR003593: AAA+ ATPase domain; IPR011527: ABC transporter type 1, transmembrane domain; IPR027417: P-loop containing nucleoside triphosphate hydrolase | GO:0005524 MF: ATP binding;GO:0006810 BP: transport; |
|  |  |  |  |  | TraesCS3B01G607700 | Disease resistance protein RPP13 | PF00931: NB-ARC domain | IPR002182: NB-ARC; IPR011991: Winged helix-turn-helix DNA-binding domain; IPR027417: P-loop containing nucleoside triphosphate hydrolase; IPR032675: Leucine-rich repeat domain, L domain-like | GO:0043531 MF: ADP binding |
|  |  |  |  |  | TraesCS3B01G607800 | ubiquinone biosynthesis protein (Protein of unknown function, DUF547) | NA | NA | NA |
|  |  |  |  |  | TraesCS3B01G607900 | Dirigent protein | PF03018: Dirigent-like protein | IPR000772: Ricin B, lectin domain; IPR004265: Plant disease resistance response protein | NA |
|  |  |  |  |  | TraesCS3B01G608000 | Pro-apoptotic serine protease nma111 | NA | NA | NA |
|  |  |  |  |  | TraesCS3B01G608100 | Leucine-rich repeat (LRR) family protein | NA | NA | NA |
|  |  |  |  |  | TraesCS3B01G608200 | Vacuolar fusion protein MON1 | PF03164: Trafficking protein Mon1 | IPR004353: Vacuolar fusion protein Mon1 | NA |
|  |  |  |  |  | TraesCS3B01G608300 | Monopolar spindle protein 2 | PF07795: Protein of unknown function (DUF1635) | IPR012862: Protein of unknown function DUF1635 | NA |
|  |  |  |  |  | TraesCS3B01G608400 | GDSL esterase/lipase | PF00657: GDSL-like Lipase/Acylhydrolase | IPR001087: GDSL lipase/esterase; IPR013830: SGNH hydrolase-type esterase domain | GO:0016788 MF: hydrolase activity, acting on ester bonds |
|  |  |  |  |  | TraesCS3B01G608500 | Aquaporin | PF00230: Major intrinsic protein | IPR000425: Major intrinsic protein; IPR022357: Major intrinsic protein, conserved site; IPR023271: Aquaporin-like | GO:0005215 MF: transporter activity;GO:0006810 BP: transport; |
|  |  |  |  |  | TraesCS3B01G608600 | MADS-box transcription factor | PF00319: SRF-type transcription factor (DNA-binding and dimerisation domain) | IPR002100: Transcription factor, MADS-box | GO:0003677 MF: DNA binding;GO:0046983 MF: protein dimerization activity |
| STI_SL | BS00073411_51 |  | 3B | 829197896 | TraesCS3B01G608900 | Dof zinc finger protein | PF02701: Dof domain, zinc finger | IPR003851: Zinc finger, Dof-type | GO:0003677 MF: DNA binding;GO:0006355 BP: regulation of transcription, DNA-templated |
|  |  |  |  |  | TraesCS3B01G609000 | Dof zinc finger protein | PF02701: Dof domain, zinc finger | IPR003851: Zinc finger, Dof-type | GO:0003677 MF: DNA binding;GO:0006355 BP: regulation of transcription, DNA-templated |
|  |  |  |  |  | TraesCS3B01G609100 | Dof zinc finger protein | PF02701: Dof domain, zinc finger | IPR003851: Zinc finger, Dof-type | GO:0003677 MF: DNA binding;GO:0006355 BP: regulation of transcription, DNA-templated |
|  |  |  |  |  | TraesCS3B01G609200 | transmembrane protein | PF14990: Domain of unknown function (DUF4516) | IPR027858: Protein of unknown function DUF4516 | NA |
|  |  |  |  |  | TraesCS3B01G609300 | F-box protein-like protein | PF12937: F-box-like | IPR001810: F-box domain; IPR032675: Leucine-rich repeat domain, L domain-like | GO:0005515 MF: protein binding |
|  |  |  |  |  | TraesCS3B01G609400 | Cytochrome P450 | PF00067: Cytochrome P450 | IPR001128: Cytochrome P450; IPR002401: Cytochrome P450, E-class, group I; IPR017972: Cytochrome P450, conserved site | GO:0005506 MF: iron ion binding;GO:0016705 MF: oxidoreductase activity, acting on paired donors, with incorporation or reduction of molecular oxygen; |
|  |  |  |  |  | TraesCS3B01G609500 | Ankyrin repeat family protein | PF12796: Ankyrin repeats (3 copies); PF00023: Ankyrin repeat | IPR001841: Zinc finger, RING-type; IPR002110: Ankyrin repeat; IPR013083: Zinc finger, RING/FYVE/PHD-type; IPR020683: Ankyrin repeat-containing domain | GO:0005515 MF: protein binding;GO:0008270 MF: zinc ion binding |
|  |  |  |  |  | TraesCS3B01G609600 | Cytochrome P450 | PF00067: Cytochrome P450 | IPR001128: Cytochrome P450; IPR002401: Cytochrome P450, E-class, group I; IPR017972: Cytochrome P450, conserved site | GO:0005506 MF: iron ion binding;GO:0016705 MF: oxidoreductase activity, acting on paired donors, with incorporation or reduction of molecular oxygen;GO:0020037 MF: heme binding; |
|  |  |  |  |  | TraesCS3B01G609700 | Nodulin MtN21 /EamA-like transporter family protein | NA | NA | NA |
|  |  |  |  |  | TraesCS3B01G609800 | 3-oxo-5-alpha-steroid 4-dehydrogenase family protein | PF14990: Domain of unknown function (DUF4516) | IPR027858: Protein of unknown function DUF4516 | NA |
|  |  |  |  |  | TraesCS3B01G609900 | Pre-mRNA-splicing factor ISY1-like protein | PF06246: Isy1-like splicing family | IPR009360: Pre-mRNA-splicing factor Isy1 | GO:0000350 BP: generation of catalytic spliceosome for second transesterification step |
|  |  |  |  |  | TraesCS3B01G610000 | Protein CHUP1, chloroplastic | NA | NA | NA |
|  |  |  |  |  | TraesCS3B01G610100 | Pectin acetylesterase | PF03283: Pectinacetylesterase | IPR004963: Pectinacetylesterase/NOTUM; IPR029058: Alpha/Beta hydrolase fold | GO:0016787 MF: hydrolase activity |
|  |  |  |  |  | TraesCS3B01G610200 | Pectin acetylesterase | PF03283: Pectinacetylesterase | IPR004963: Pectinacetylesterase/NOTUM; IPR029058: Alpha/Beta hydrolase fold | GO:0016787 MF: hydrolase activity |
|  |  |  |  |  | TraesCS3B01G610300 | Pectin acetylesterase | PF03283: Pectinacetylesterase | IPR004963: Pectinacetylesterase/NOTUM; IPR029058: Alpha/Beta hydrolase fold | GO:0016787 MF: hydrolase activity |
|  |  |  |  |  | TraesCS3B01G610400 | Pectin acetylesterase | PF03283: Pectinacetylesterase | IPR004963: Pectinacetylesterase/NOTUM | GO:0016787 MF: hydrolase activity |
|  |  |  |  |  | TraesCS3B01G610500 | 91A protein | NA | NA | NA |
|  |  |  |  |  | TraesCS3B01G610600 | Pectin acetylesterase | PF03283: Pectinacetylesterase | IPR004963: Pectinacetylesterase/NOTUM; IPR029058: Alpha/Beta hydrolase fold | GO:0016787 MF: hydrolase activity |
|  |  |  |  |  | TraesCS3B01G610700 | B3 domain-containing protein | PF02362: B3 DNA binding domain | IPR003340: B3 DNA binding domain; IPR015300: DNA-binding pseudobarrel domain | GO:0003677 MF: DNA binding |
| STI_SL | AX-111015220 |  | 3B | 829203418 | TraesCS3B01G610800 | Histone H2A | PF00125: Core histone H2A/H2B/H3/H4; PF16211: C-terminus of histone H2A | IPR002119: Histone H2A; IPR007125: Histone H2A/H2B/H3; IPR009072: Histone-fold; IPR032454: Histone H2A, C-terminal domain; IPR032458: Histone H2A conserved site | GO:0000786 CC: nucleosome;GO:0003677 MF: DNA binding; |
|  |  |  |  |  | TraesCS3B01G610900 | RNA-binding protein | PF00076: RNA recognition motif. (a.k.a. RRM, RBD, or RNP domain) | IPR000504: RNA recognition motif domain | GO:0003676 MF: nucleic acid binding |
|  |  |  |  |  | TraesCS3B01G611100 | Receptor-like protein kinase | PF00069: Protein kinase domain; PF00635: MSP (Major sperm protein) domain; PF00931: NB-ARC domain | IPR000535: Major sperm protein (MSP) domain; IPR000719: Protein kinase domain; IPR002182: NB-ARC; IPR008271: Serine/threonine-protein kinase, active site; IPR008962: PapD-like; IPR011009: Protein kinase-like domain; IPR013783: Immunoglobulin-like fold; IPR027417: P-loop containing nucleoside triphosphate hydrolase | GO:0004672 MF: protein kinase activity;GO:0005524 MF: ATP binding; |
|  |  |  |  |  | TraesCS3B01G611200 | DNA topoisomerase 3 | PF13968: Domain of unknown function (DUF4220); PF04578: Protein of unknown function, DUF594 | IPR007658: Protein of unknown function DUF594; IPR025315: Domain of unknown function DUF4220 | NA |
|  |  |  |  |  | TraesCS3B01G611300 | Histone H2A | PF00125: Core histone H2A/H2B/H3/H4; PF16211: C-terminus of histone H2A | IPR002119: Histone H2A; IPR007125: Histone H2A/H2B/H3; IPR009072: Histone-fold; IPR032454: Histone H2A, C-terminal domain; IPR032458: Histone H2A conserved site | GO:0000786 CC: nucleosome;GO:0003677 MF: DNA binding; |
|  |  |  |  |  | TraesCS3B01G611400 | Outer-membrane lipoprotein LolB | NA | NA | NA |
|  |  |  |  |  | TraesCS3B01G611500 | transmembrane protein, putative (DUF594) | PF13968: Domain of unknown function (DUF4220); PF04578: Protein of unknown function, DUF594 | IPR007658: Protein of unknown function DUF594; IPR025315: Domain of unknown function DUF4220 | NA |
|  |  |  |  |  | TraesCS3B01G611600 | Soluble inorganic pyrophosphatase | PF00719: Inorganic pyrophosphatase | IPR008162: Inorganic pyrophosphatase | GO:0000287 MF: magnesium ion binding;GO:0004427 MF: inorganic diphosphatase activity; |
|  |  |  |  |  | TraesCS3B01G611700 | Kelch repeat-containing F-box family protein | PF00646: F-box domain; PF01344: Kelch motif | IPR001810: F-box domain; IPR006652: Kelch repeat type 1; IPR015915: Kelch-type beta propeller | GO:0005515 MF: protein binding |
|  |  |  |  |  | TraesCS3B01G611800 | Soluble inorganic pyrophosphatase | PF00719: Inorganic pyrophosphatase | IPR008162: Inorganic pyrophosphatase | GO:0000287 MF: magnesium ion binding;GO:0004427 MF: inorganic diphosphatase activity; |
|  |  |  |  |  | TraesCS3B01G611900 | Ubiquitin family protein | PF00240: Ubiquitin family | IPR000626: Ubiquitin domain; IPR029071: Ubiquitin-related domain | GO:0005515 MF: protein binding |
|  |  |  |  |  | TraesCS3B01G612000 | O-methyltransferase | PF08100: Dimerisation domain; PF00891: O-methyltransferase | IPR001077: O-methyltransferase, family 2; IPR011991: Winged helix-turn-helix DNA-binding domain; IPR012967: Plant methyltransferase dimerisation; IPR016461: O-methyltransferase COMT-type; IPR029063: S-adenosyl-L-methionine-dependent methyltransferase | GO:0008168 MF: methyltransferase activity;GO:0008171 MF: O-methyltransferase activity;GO:0046983 MF: protein dimerization activity |
|  |  |  |  |  | TraesCS3B01G612100 | Protein upstream of flc | PF06136: Domain of unknown function (DUF966) | IPR010369: Protein of unknown function DUF966; IPR021182: Uncharacterised conserved protein UCP031043 | NA |
|  |  |  |  |  | TraesCS3B01G612200 | MYB transcription factor | PF00249: Myb-like DNA-binding domain | IPR001005: SANT/Myb domain; IPR009057: Homeobox domain-like; IPR017930: Myb domain | GO:0003677 MF: DNA binding |
|  |  |  |  |  | TraesCS3B01G612300 | Transcription factor, MADS-box | PF00319: SRF-type transcription factor (DNA-binding and dimerisation domain) | IPR002100: Transcription factor, MADS-box | GO:0003677 MF: DNA binding;GO:0046983 MF: protein dimerization activity |
|  |  |  |  |  | TraesCS3B01G612400 | Transcription factor, MADS-box | PF00319: SRF-type transcription factor (DNA-binding and dimerisation domain) | IPR002100: Transcription factor, MADS-box | GO:0003677 MF: DNA binding;GO:0046983 MF: protein dimerization activity |
|  |  |  |  |  | TraesCS3B01G612500 | Transcription factor, MADS-box | PF00319: SRF-type transcription factor (DNA-binding and dimerisation domain) | IPR002100: Transcription factor, MADS-box | GO:0003677 MF: DNA binding;GO:0046983 MF: protein dimerization activity |
|  |  |  |  |  | TraesCS3B01G612600 | Transcription factor, MADS-box | PF00319: SRF-type transcription factor (DNA-binding and dimerisation domain) | IPR002100: Transcription factor, MADS-box | GO:0003677 MF: DNA binding;GO:0046983 MF: protein dimerization activity |
|  |  |  |  |  | TraesCS3B01G612700 | Transcription factor, MADS-box | PF00319: SRF-type transcription factor (DNA-binding and dimerisation domain) | IPR002100: Transcription factor, MADS-box | GO:0003677 MF: DNA binding;GO:0046983 MF: protein dimerization activity |
| STI_SL | AX-158578652 |  | 3B | 829293411 | TraesCS3B01G609100 | Dof zinc finger protein | PF02701: Dof domain, zinc finger | IPR003851: Zinc finger, Dof-type | GO:0003677 MF: DNA binding;GO:0006355 BP: regulation of transcription, DNA-templated |
|  |  |  |  |  | TraesCS3B01G609200 | transmembrane protein | PF14990: Domain of unknown function (DUF4516) | IPR027858: Protein of unknown function DUF4516 | NA |
|  |  |  |  |  | TraesCS3B01G609300 | F-box protein-like protein | PF12937: F-box-like | IPR001810: F-box domain; IPR032675: Leucine-rich repeat domain, L domain-like | GO:0005515 MF: protein binding |
|  |  |  |  |  | TraesCS3B01G609400 | Cytochrome P450 | PF00067: Cytochrome P450 | IPR001128: Cytochrome P450; IPR002401: Cytochrome P450, E-class, group I; IPR017972: Cytochrome P450, conserved site | GO:0005506 MF: iron ion binding;GO:0016705 MF: oxidoreductase activity, acting on paired donors, with incorporation or reduction of molecular oxygen; |
|  |  |  |  |  | TraesCS3B01G609500 | Ankyrin repeat family protein | PF12796: Ankyrin repeats (3 copies); PF00023: Ankyrin repeat | IPR001841: Zinc finger, RING-type; IPR002110: Ankyrin repeat; IPR013083: Zinc finger, RING/FYVE/PHD-type; IPR020683: Ankyrin repeat-containing domain | GO:0005515 MF: protein binding;GO:0008270 MF: zinc ion binding |
|  |  |  |  |  | TraesCS3B01G609600 | Cytochrome P450 | PF00067: Cytochrome P450 | IPR001128: Cytochrome P450; IPR002401: Cytochrome P450, E-class, group I; IPR017972: Cytochrome P450, conserved site | GO:0005506 MF: iron ion binding;GO:0016705 MF: oxidoreductase activity, acting on paired donors, with incorporation or reduction of molecular oxygen;GO:0020037 MF: heme binding;GO:0055114 BP: oxidation-reduction process |
|  |  |  |  |  | TraesCS3B01G609700 | Nodulin MtN21 /EamA-like transporter family protein | NA | NA | NA |
|  |  |  |  |  | TraesCS3B01G609800 | 3-oxo-5-alpha-steroid 4-dehydrogenase family protein | PF14990: Domain of unknown function (DUF4516) | IPR027858: Protein of unknown function DUF4516 | NA |
|  |  |  |  |  | TraesCS3B01G609900 | Pre-mRNA-splicing factor ISY1-like protein | PF06246: Isy1-like splicing family | IPR009360: Pre-mRNA-splicing factor Isy1 | GO:0000350 BP: generation of catalytic spliceosome for second transesterification step |
|  |  |  |  |  | TraesCS3B01G610000 | Protein CHUP1, chloroplastic | NA | NA | NA |
|  |  |  |  |  | TraesCS3B01G610100 | Pectin acetylesterase | PF03283: Pectinacetylesterase | IPR004963: Pectinacetylesterase/NOTUM; IPR029058: Alpha/Beta hydrolase fold | GO:0016787 MF: hydrolase activity |
|  |  |  |  |  | TraesCS3B01G610200 | Pectin acetylesterase | PF03283: Pectinacetylesterase | IPR004963: Pectinacetylesterase/NOTUM; IPR029058: Alpha/Beta hydrolase fold | GO:0016787 MF: hydrolase activity |
|  |  |  |  |  | TraesCS3B01G610300 | Pectin acetylesterase | PF03283: Pectinacetylesterase | IPR004963: Pectinacetylesterase/NOTUM; IPR029058: Alpha/Beta hydrolase fold | GO:0016787 MF: hydrolase activity |
|  |  |  |  |  | TraesCS3B01G610400 | Pectin acetylesterase | PF03283: Pectinacetylesterase | IPR004963: Pectinacetylesterase/NOTUM | GO:0016787 MF: hydrolase activity |
|  |  |  |  |  | TraesCS3B01G610500 | 91A protein | NA | NA | NA |
|  |  |  |  |  | TraesCS3B01G610600 | Pectin acetylesterase | PF03283: Pectinacetylesterase | IPR004963: Pectinacetylesterase/NOTUM; IPR029058: Alpha/Beta hydrolase fold | GO:0016787 MF: hydrolase activity |
|  |  |  |  |  | TraesCS3B01G610700 | B3 domain-containing protein | PF02362: B3 DNA binding domain | IPR003340: B3 DNA binding domain; IPR015300: DNA-binding pseudobarrel domain | GO:0003677 MF: DNA binding |
|  |  |  |  |  | TraesCS3B01G610800 | Histone H2A | PF00125: Core histone H2A/H2B/H3/H4; PF16211: C-terminus of histone H2A | IPR002119: Histone H2A; IPR007125: Histone H2A/H2B/H3; IPR009072: Histone-fold; IPR032454: Histone H2A, C-terminal domain; IPR032458: Histone H2A conserved site | GO:0000786 CC: nucleosome;GO:0003677 MF: DNA binding; |
|  |  |  |  |  | TraesCS3B01G610900 | RNA-binding protein | PF00076: RNA recognition motif. (a.k.a. RRM, RBD, or RNP domain) | IPR000504: RNA recognition motif domain | GO:0003676 MF: nucleic acid binding |
|  |  |  |  |  | TraesCS3B01G611100 | Receptor-like protein kinase | PF00069: Protein kinase domain; PF00635: MSP (Major sperm protein) domain; PF00931: NB-ARC domain | IPR000535: Major sperm protein (MSP) domain; IPR000719: Protein kinase domain; IPR002182: NB-ARC; IPR008271: Serine/threonine-protein kinase, active site; IPR008962: PapD-like; IPR011009: Protein kinase-like domain; IPR013783: Immunoglobulin-like fold; IPR027417: P-loop containing nucleoside triphosphate hydrolase | GO:0004672 MF: protein kinase activity;GO:0005524 MF: ATP binding; |
|  |  |  |  |  | TraesCS3B01G611200 | DNA topoisomerase 3 | PF13968: Domain of unknown function (DUF4220); PF04578: Protein of unknown function, DUF594 | IPR007658: Protein of unknown function DUF594; IPR025315: Domain of unknown function DUF4220 | NA |
|  |  |  |  |  | TraesCS3B01G611300 | Histone H2A | PF00125: Core histone H2A/H2B/H3/H4; PF16211: C-terminus of histone H2A | IPR002119: Histone H2A; IPR007125: Histone H2A/H2B/H3; IPR009072: Histone-fold; IPR032454: Histone H2A, C-terminal domain; IPR032458: Histone H2A conserved site | GO:0000786 CC: nucleosome;GO:0003677 MF: DNA binding; |
|  |  |  |  |  | TraesCS3B01G611400 | Outer-membrane lipoprotein LolB | NA | NA | NA |
|  |  |  |  |  | TraesCS3B01G611500 | transmembrane protein, putative (DUF594) | PF13968: Domain of unknown function (DUF4220); PF04578: Protein of unknown function, DUF594 | IPR007658: Protein of unknown function DUF594; IPR025315: Domain of unknown function DUF4220 | NA |
|  |  |  |  |  | TraesCS3B01G611600 | Soluble inorganic pyrophosphatase | PF00719: Inorganic pyrophosphatase | IPR008162: Inorganic pyrophosphatase | GO:0000287 MF: magnesium ion binding;GO:0004427 MF: inorganic diphosphatase activity; |
|  |  |  |  |  | TraesCS3B01G611700 | Kelch repeat-containing F-box family protein | PF00646: F-box domain; PF01344: Kelch motif | IPR001810: F-box domain; IPR006652: Kelch repeat type 1; IPR015915: Kelch-type beta propeller | GO:0005515 MF: protein binding |
|  |  |  |  |  | TraesCS3B01G611800 | Soluble inorganic pyrophosphatase | PF00719: Inorganic pyrophosphatase | IPR008162: Inorganic pyrophosphatase | GO:0000287 MF: magnesium ion binding;GO:0004427 MF: inorganic diphosphatase activity; |
|  |  |  |  |  | TraesCS3B01G611900 | Ubiquitin family protein | PF00240: Ubiquitin family | IPR000626: Ubiquitin domain; IPR029071: Ubiquitin-related domain | GO:0005515 MF: protein binding |
|  |  |  |  |  | TraesCS3B01G612000 | O-methyltransferase | PF08100: Dimerisation domain; PF00891: O-methyltransferase | IPR001077: O-methyltransferase, family 2; IPR011991: Winged helix-turn-helix DNA-binding domain; IPR012967: Plant methyltransferase dimerisation; IPR016461: O-methyltransferase COMT-type; IPR029063: S-adenosyl-L-methionine-dependent methyltransferase | GO:0008168 MF: methyltransferase activity;GO:0008171 MF: O-methyltransferase activity; |
|  |  |  |  |  | TraesCS3B01G612100 | Protein upstream of flc | PF06136: Domain of unknown function (DUF966) | IPR010369: Protein of unknown function DUF966; IPR021182: Uncharacterised conserved protein UCP031043 | NA |
|  |  |  |  |  | TraesCS3B01G612200 | NA | NA | NA | NA |
|  |  |  |  |  | TraesCS3B01G612200 | MYB transcription factor | PF00249: Myb-like DNA-binding domain | IPR001005: SANT/Myb domain; IPR009057: Homeobox domain-like; IPR017930: Myb domain | GO:0003677 MF: DNA binding |
|  |  |  |  |  | TraesCS3B01G612300 | Transcription factor, MADS-box | PF00319: SRF-type transcription factor (DNA-binding and dimerisation domain) | IPR002100: Transcription factor, MADS-box | GO:0003677 MF: DNA binding;GO:0046983 MF: protein dimerization activity |
|  |  |  |  |  | TraesCS3B01G612400 | Transcription factor, MADS-box | PF00319: SRF-type transcription factor (DNA-binding and dimerisation domain) | IPR002100: Transcription factor, MADS-box | GO:0003677 MF: DNA binding;GO:0046983 MF: protein dimerization activity |
|  |  |  |  |  | TraesCS3B01G612500 | Transcription factor, MADS-box | PF00319: SRF-type transcription factor (DNA-binding and dimerisation domain) | IPR002100: Transcription factor, MADS-box | GO:0003677 MF: DNA binding;GO:0046983 MF: protein dimerization activity |
|  |  |  |  |  | TraesCS3B01G612600 | Transcription factor, MADS-box | PF00319: SRF-type transcription factor (DNA-binding and dimerisation domain) | IPR002100: Transcription factor, MADS-box | GO:0003677 MF: DNA binding;GO:0046983 MF: protein dimerization activity |
|  |  |  |  |  | TraesCS3B01G612700 | Transcription factor, MADS-box | PF00319: SRF-type transcription factor (DNA-binding and dimerisation domain) | IPR002100: Transcription factor, MADS-box | GO:0003677 MF: DNA binding;GO:0046983 MF: protein dimerization activity |
| STI_RSRatio | AX-108852904 |  | 3B | 822891332 | TraesCS3B01G601500 | 11S globulin seed storage protein 2 | PF00190: Cupin | IPR006044: 11-S seed storage protein, plant; IPR006045: Cupin 1; IPR011051: RmlC-like cupin domain; IPR014710: RmlC-like jelly roll fold | GO:0045735 MF: nutrient reservoir activity |
|  |  |  |  |  | TraesCS3B01G601600 | Metallothionein | PF01439: Metallothionein | IPR000347: Metallothionein, family 15, plant | GO:0046872 MF: metal ion binding |
|  |  |  |  |  | TraesCS3B01G601700 | Metallothionein | PF01439: Metallothionein | IPR000347: Metallothionein, family 15, plant | GO:0046872 MF: metal ion binding |
|  |  |  |  |  | TraesCS3B01G601800 | Metallothionein | PF01439: Metallothionein | IPR000347: Metallothionein, family 15, plant | GO:0046872 MF: metal ion binding |
|  |  |  |  |  | TraesCS3B01G601900 | Plant invertase/pectin methylesterase inhibitor superfamily protein | NA | NA | NA |
|  |  |  |  |  | TraesCS3B01G602000 | 30S ribosomal protein S17 | PF00366: Ribosomal protein S17 | IPR000266: Ribosomal protein S17/S11; IPR012340: Nucleic acid-binding, OB-fold; IPR019984: 30S ribosomal protein S17 | GO:0003735 MF: structural constituent of ribosome;GO:0005622 CC: intracellular; |
|  |  |  |  |  | TraesCS3B01G602100 | 30S ribosomal protein S19 | PF00203: Ribosomal protein S19 | IPR002222: Ribosomal protein S19/S15; IPR005732: Ribosomal protein S19, bacterial-type; IPR020934: Ribosomal protein S19 conserved site; IPR023575: Ribosomal protein S19, superfamily | GO:0003723 MF: RNA binding;GO:0003735 MF: structural constituent of ribosome; |
|  |  |  |  |  | TraesCS3B01G602200 | SANT domain-containing protein 2 | NA | NA | NA |
|  |  |  |  |  | TraesCS3B01G602300 | Mitochondrial ATP synthase 6 kDa subunit | NA | NA | NA |
|  |  |  |  |  | TraesCS3B01G602400 | PR5-like receptor kinase | PF00069: Protein kinase domain | IPR000719: Protein kinase domain; IPR001680: WD40 repeat; IPR008271: Serine/threonine-protein kinase, active site; IPR008962: PapD-like; IPR011009: Protein kinase-like domain; IPR015943: WD40/YVTN repeat-like-containing domain; IPR017441: Protein kinase, ATP binding site; IPR017986: WD40-repeat-containing domain; IPR019775: WD40 repeat, conserved site | GO:0004672 MF: protein kinase activity;GO:0005515 MF: protein binding; |
|  |  |  |  |  | TraesCS3B01G602500 | Lipoxygenase | PF01477: PLAT/LH2 domain; PF00305: Lipoxygenase | IPR001024: PLAT/LH2 domain; IPR001246: Lipoxygenase, plant; IPR013819: Lipoxygenase, C-terminal; IPR020833: Lipoxygenase, iron binding site; IPR020834: Lipoxygenase, conserved site; IPR027433: Lipoxygenase, domain 3 | GO:0005515 MF: protein binding;GO:0016491 MF: oxidoreductase activity; |
|  |  |  |  |  | TraesCS3B01G602600 | Extra-large guanine nucleotide binding family protein | NA | IPR013083: Zinc finger, RING/FYVE/PHD-type | NA |
|  |  |  |  |  | TraesCS3B01G602700 | Protein kinase-like protein | PF08263: Leucine rich repeat N-terminal domain; PF13855: Leucine rich repeat; PF00069: Protein kinase domain | IPR000719: Protein kinase domain; IPR001611: Leucine-rich repeat; IPR003591: Leucine-rich repeat, typical subtype; IPR008271: Serine/threonine-protein kinase, active site; IPR011009: Protein kinase-like domain; IPR013210: Leucine-rich repeat-containing N-terminal, plant-type; IPR017441: Protein kinase, ATP binding site; IPR032675: Leucine-rich repeat domain, L domain-like | GO:0004672 MF: protein kinase activity;GO:0005515 MF: protein binding; |
|  |  |  |  |  | TraesCS3B01G602800 | Plant cadmium resistance protein | PF11204: Protein of unknown function (DUF2985); PF04749: PLAC8 family | IPR006461: PLAC8 motif-containing protein; IPR015943: WD40/YVTN repeat-like-containing domain; IPR021369: Protein of unknown function DUF2985 | GO:0005515 MF: protein binding |
|  |  |  |  |  | TraesCS3B01G602900 | Dirigent protein | PF03018: Dirigent-like protein | IPR004265: Plant disease resistance response protein | NA |
|  |  |  |  |  | TraesCS3B01G603000 | Gibberellin 2-beta-dioxygenase | PF14226: non-haem dioxygenase in morphine synthesis N-terminal; PF03171: 2OG-Fe(II) oxygenase superfamily | IPR005123: Oxoglutarate/iron-dependent dioxygenase; IPR026992: Non-haem dioxygenase N-terminal domain; IPR027443: Isopenicillin N synthase-like | GO:0016491 MF: oxidoreductase activity;GO:0055114 BP: oxidation-reduction process |
|  |  |  |  |  | TraesCS3B01G603100 | Chaperone protein dnaJ, putative | PF13432: Tetratricopeptide repeat; PF13181: Tetratricopeptide repeat; PF00226: DnaJ domain | IPR001623: DnaJ domain; IPR011990: Tetratricopeptide-like helical domain; IPR013026: Tetratricopeptide repeat-containing domain; IPR018253: DnaJ domain, conserved site; IPR019734: Tetratricopeptide repeat | GO:0005515 MF: protein binding |
|  |  |  |  |  | TraesCS3B01G603200 | SKP1-like protein | PF01466: Skp1 family, dimerisation domain | IPR001232: S-phase kinase-associated protein 1-like; IPR011333: SKP1/BTB/POZ domain; IPR016072: SKP1 component, dimerisation | GO:0006511 BP: ubiquitin-dependent protein catabolic process |
|  |  |  |  |  | TraesCS3B01G603300 | Kinase family protein | PF07714: Protein tyrosine kinase | IPR000719: Protein kinase domain; IPR001245: Serine-threonine/tyrosine-protein kinase, catalytic domain; IPR008271: Serine/threonine-protein kinase, active site; IPR011009: Protein kinase-like domain; IPR017441: Protein kinase, ATP binding site | GO:0004672 MF: protein kinase activity;GO:0005524 MF: ATP binding;GO:0006468 BP: protein phosphorylation |
|  |  |  |  |  | TraesCS3B01G603400 | E3 SUMO-protein ligase SIZ1 | PF02891: MIZ/SP-RING zinc finger | IPR001965: Zinc finger, PHD-type; IPR003034: SAP domain; IPR004181: Zinc finger, MIZ-type; IPR011011: Zinc finger, FYVE/PHD-type; IPR013083: Zinc finger, RING/FYVE/PHD-type; IPR019786: Zinc finger, PHD-type, conserved site | GO:0005515 MF: protein binding;GO:0008270 MF: zinc ion binding |
|  |  |  |  |  | TraesCS3B01G603500 | Protein IQ-DOMAIN 1 | PF00612: IQ calmodulin-binding motif; PF13178: Protein of unknown function (DUF4005) | IPR000048: IQ motif, EF-hand binding site; IPR025064: Domain of unknown function DUF4005 | GO:0005515 MF: protein binding |
|  |  |  |  |  | TraesCS3B01G603600 | Serine/threonine-protein kinase ATM | PF00855: PWWP domain | IPR000313: PWWP domain | NA |
|  |  |  |  |  | TraesCS3B01G603700 | Serine/threonine-protein kinase ATM | NA | IPR000504: RNA recognition motif domain | GO:0003676 MF: nucleic acid binding |
|  |  |  |  |  | TraesCS3B01G603800 | Kinase-like protein | PF12819: Carbohydrate-binding protein of the ER; PF07714: Protein tyrosine kinase | IPR000719: Protein kinase domain; IPR001245: Serine-threonine/tyrosine-protein kinase, catalytic domain; IPR008271: Serine/threonine-protein kinase, active site; IPR011009: Protein kinase-like domain; IPR017441: Protein kinase, ATP binding site; IPR024788: Malectin-like carbohydrate-binding domain; IPR032675: Leucine-rich repeat domain, L domain-like | GO:0004672 MF: protein kinase activity;GO:0005524 MF: ATP binding;GO:0006468 BP: protein phosphorylation |
|  |  |  |  |  | TraesCS3B01G603900 | GATA transcription factor, putative | PF00320: GATA zinc finger | IPR000679: Zinc finger, GATA-type; IPR013088: Zinc finger, NHR/GATA-type | GO:0003700 MF: transcription factor activity, sequence-specific DNA binding;GO:0006355 BP: regulation of transcription, DNA-templated; |
|  |  |  |  |  | TraesCS3B01G604000 | Protein phosphatase 2C family protein | PF00481: Protein phosphatase 2C | IPR000222: PPM-type phosphatase, divalent cation binding; IPR001932: PPM-type phosphatase domain | GO:0003824 MF: catalytic activity;GO:0043169 MF: cation binding |
|  |  |  |  |  | TraesCS3B01G604100 | NBS-LRR disease resistance protein | PF00931: NB-ARC domain | IPR002182: NB-ARC; IPR011991: Winged helix-turn-helix DNA-binding domain; IPR027417: P-loop containing nucleoside triphosphate hydrolase; IPR032675: Leucine-rich repeat domain, L domain-like | GO:0043531 MF: ADP binding |
|  |  |  |  |  | TraesCS3B01G604200 | Ubiquitin carboxyl-terminal hydrolase 2 | PF13968: Domain of unknown function (DUF4220) | IPR025315: Domain of unknown function DUF4220 | NA |
|  |  |  |  |  | TraesCS3B01G604300 | rRNA N-glycosidase | PF00161: Ribosome inactivating protein | IPR001574: Ribosome-inactivating protein; IPR016138: Ribosome-inactivating protein, subdomain 1 | GO:0017148 BP: negative regulation of translation;GO:0030598 MF: rRNA N-glycosylase activity |
|  |  |  |  |  | TraesCS3B01G604400 | rRNA N-glycosidase | NA | NA | NA |
|  |  |  |  |  | TraesCS3B01G604500 | NBS-LRR resistance-like protein | PF00931: NB-ARC domain | IPR002182: NB-ARC; IPR011991: Winged helix-turn-helix DNA-binding domain; IPR027417: P-loop containing nucleoside triphosphate hydrolase; IPR032675: Leucine-rich repeat domain, L domain-like | GO:0043531 MF: ADP binding |
|  |  |  |  |  | TraesCS3B01G604600 | Disease resistance protein (NBS-LRR class) family | PF00931: NB-ARC domain | IPR002182: NB-ARC; IPR027417: P-loop containing nucleoside triphosphate hydrolase | GO:0043531 MF: ADP binding |
| STI_RSRatio | BS00071183_51 |  | 3B | 823762843 | TraesCS3B01G604700 | External alternative NAD(P)H-ubiquinone oxidoreductase B2, mitochondrial | PF07992: Pyridine nucleotide-disulphide oxidoreductase | IPR023753: FAD/NAD(P)-binding domain | GO:0016491 MF: oxidoreductase activity;GO:0055114 BP: oxidation-reduction process |
|  |  |  |  |  | TraesCS3B01G604800 | NBS-LRR-like resistance protein | PF00931: NB-ARC domain | IPR002182: NB-ARC; IPR011991: Winged helix-turn-helix DNA-binding domain; IPR027417: P-loop containing nucleoside triphosphate hydrolase; IPR032675: Leucine-rich repeat domain, L domain-like | GO:0043531 MF: ADP binding |
|  |  |  |  |  | TraesCS3B01G604900 | Disease resistance protein (NBS-LRR class) family | PF00931: NB-ARC domain; PF00161: Ribosome inactivating protein | IPR001574: Ribosome-inactivating protein; IPR002182: NB-ARC; IPR011991: Winged helix-turn-helix DNA-binding domain; IPR016138: Ribosome-inactivating protein, subdomain 1; IPR027417: P-loop containing nucleoside triphosphate hydrolase; IPR032675: Leucine-rich repeat domain, L domain-like | GO:0017148 BP: negative regulation of translation;GO:0030598 MF: rRNA N-glycosylase activity; |
|  |  |  |  |  | TraesCS3B01G605000 | transmembrane protein, putative (DUF594) | PF13968: Domain of unknown function (DUF4220); PF04578: Protein of unknown function, DUF594 | IPR007658: Protein of unknown function DUF594; IPR025315: Domain of unknown function DUF4220 | NA |
|  |  |  |  |  | TraesCS3B01G605100 | Kinase interacting (KIP1-like) family protein | PF00856: SET domain; PF09273: Rubisco LSMT substrate-binding; PF07765: KIP1-like protein | IPR001214: SET domain; IPR011684: Protein Networked (NET), actin-binding (NAB) domain; IPR015353: Rubisco LSMT, substrate-binding domain | GO:0003779 MF: actin binding;GO:0005515 MF: protein binding |
|  |  |  |  |  | TraesCS3B01G605200 | Transmembrane protein, putative | PF06749: Protein of unknown function (DUF1218) | IPR009606: Protein of unknown function DUF1218 | NA |
|  |  |  |  |  | TraesCS3B01G605300 | ubiquitin carboxyl-terminal hydrolase-like protein, putative (DUF627 and DUF629) | NA | NA | NA |
|  |  |  |  |  | TraesCS3B01G605400 | Divalent ion symporter | NA | NA | NA |
|  |  |  |  |  | TraesCS3B01G605500 | Cortactin-binding protein 2 | NA | NA | NA |
|  |  |  |  |  | TraesCS3B01G605600 | AGAP002737-PA | NA | NA | NA |
|  |  |  |  |  | TraesCS3B01G605700 | Lipoxygenase | PF01477: PLAT/LH2 domain; PF00305: Lipoxygenase | IPR001024: PLAT/LH2 domain; IPR001246: Lipoxygenase, plant; IPR013819: Lipoxygenase, C-terminal; IPR020833: Lipoxygenase, iron binding site; IPR020834: Lipoxygenase, conserved site; IPR027433: Lipoxygenase, domain 3 | GO:0005515 MF: protein binding;GO:0016491 MF: oxidoreductase activity; |
|  |  |  |  |  | TraesCS3B01G605800 | plant/protein (Protein of unknown function, DUF538) | PF04398: Protein of unknown function, DUF538 | IPR007493: Protein of unknown function DUF538 | NA |
|  |  |  |  |  | TraesCS3B01G605900 | F-box protein | NA | NA | NA |
| STI_RSRatio | AX-158598301 |  | 3B | 826091387 | TraesCS3B01G607500 | Chaperone protein dnaJ | PF00226: DnaJ domain; PF11926: Domain of unknown function (DUF3444) | IPR001623: DnaJ domain; IPR024593: Domain of unknown function DUF3444 | NA |
|  |  |  |  |  | TraesCS3B01G607600 | ABC transporter B family protein | PF00664: ABC transporter transmembrane region; PF00005: ABC transporter | IPR003439: ABC transporter-like; IPR003593: AAA+ ATPase domain; IPR011527: ABC transporter type 1, transmembrane domain; IPR027417: P-loop containing nucleoside triphosphate hydrolase | GO:0005524 MF: ATP binding;GO:0006810 BP: transport; |
|  |  |  |  |  | TraesCS3B01G607700 | Disease resistance protein RPP13 | PF00931: NB-ARC domain | IPR002182: NB-ARC; IPR011991: Winged helix-turn-helix DNA-binding domain; IPR027417: P-loop containing nucleoside triphosphate hydrolase; IPR032675: Leucine-rich repeat domain, L domain-like | GO:0043531 MF: ADP binding |
|  |  |  |  |  | TraesCS3B01G607800 | ubiquinone biosynthesis protein (Protein of unknown function, DUF547) | NA | NA | NA |
|  |  |  |  |  | TraesCS3B01G607900 | Dirigent protein | PF03018: Dirigent-like protein | IPR000772: Ricin B, lectin domain; IPR004265: Plant disease resistance response protein | NA |
|  |  |  |  |  | TraesCS3B01G608000 | Pro-apoptotic serine protease nma111 | NA | NA | NA |
|  |  |  |  |  | TraesCS3B01G608100 | Leucine-rich repeat (LRR) family protein | NA | NA | NA |
|  |  |  |  |  | TraesCS3B01G608200 | Vacuolar fusion protein MON1 | PF03164: Trafficking protein Mon1 | IPR004353: Vacuolar fusion protein Mon1 | NA |
|  |  |  |  |  | TraesCS3B01G608300 | Monopolar spindle protein 2 | PF07795: Protein of unknown function (DUF1635) | IPR012862: Protein of unknown function DUF1635 | NA |
|  |  |  |  |  | TraesCS3B01G608400 | GDSL esterase/lipase | PF00657: GDSL-like Lipase/Acylhydrolase | IPR001087: GDSL lipase/esterase; IPR013830: SGNH hydrolase-type esterase domain | GO:0016788 MF: hydrolase activity, acting on ester bonds |
|  |  |  |  |  | TraesCS3B01G608500 | Aquaporin | PF00230: Major intrinsic protein | IPR000425: Major intrinsic protein; IPR022357: Major intrinsic protein, conserved site; IPR023271: Aquaporin-like | GO:0005215 MF: transporter activity;GO:0006810 BP: transport; |
|  |  |  |  |  | TraesCS3B01G608600 | MADS-box transcription factor | PF00319: SRF-type transcription factor (DNA-binding and dimerisation domain) | IPR002100: Transcription factor, MADS-box | GO:0003677 MF: DNA binding;GO:0046983 MF: protein dimerization activity |
| STI_RSRatio | BS00073411_51 |  | 3B | 829197896 | TraesCS3B01G608900 | Dof zinc finger protein | PF02701: Dof domain, zinc finger | IPR003851: Zinc finger, Dof-type | GO:0003677 MF: DNA binding;GO:0006355 BP: regulation of transcription, DNA-templated |
|  |  |  |  |  | TraesCS3B01G609000 | Dof zinc finger protein | PF02701: Dof domain, zinc finger | IPR003851: Zinc finger, Dof-type | GO:0003677 MF: DNA binding;GO:0006355 BP: regulation of transcription, DNA-templated |
|  |  |  |  |  | TraesCS3B01G609100 | Dof zinc finger protein | PF02701: Dof domain, zinc finger | IPR003851: Zinc finger, Dof-type | GO:0003677 MF: DNA binding;GO:0006355 BP: regulation of transcription, DNA-templated |
|  |  |  |  |  | TraesCS3B01G609200 | transmembrane protein | PF14990: Domain of unknown function (DUF4516) | IPR027858: Protein of unknown function DUF4516 | NA |
|  |  |  |  |  | TraesCS3B01G609300 | F-box protein-like protein | PF12937: F-box-like | IPR001810: F-box domain; IPR032675: Leucine-rich repeat domain, L domain-like | GO:0005515 MF: protein binding |
|  |  |  |  |  | TraesCS3B01G609400 | Cytochrome P450 | PF00067: Cytochrome P450 | IPR001128: Cytochrome P450; IPR002401: Cytochrome P450, E-class, group I; IPR017972: Cytochrome P450, conserved site | GO:0005506 MF: iron ion binding;GO:0016705 MF: oxidoreductase activity, acting on paired donors, with incorporation or reduction of molecular oxygen; |
|  |  |  |  |  | TraesCS3B01G609500 | Ankyrin repeat family protein | PF12796: Ankyrin repeats (3 copies); PF00023: Ankyrin repeat | IPR001841: Zinc finger, RING-type; IPR002110: Ankyrin repeat; IPR013083: Zinc finger, RING/FYVE/PHD-type; IPR020683: Ankyrin repeat-containing domain | GO:0005515 MF: protein binding;GO:0008270 MF: zinc ion binding |
|  |  |  |  |  | TraesCS3B01G609600 | Cytochrome P450 | PF00067: Cytochrome P450 | IPR001128: Cytochrome P450; IPR002401: Cytochrome P450, E-class, group I; IPR017972: Cytochrome P450, conserved site | GO:0005506 MF: iron ion binding;GO:0016705 MF: oxidoreductase activity, acting on paired donors, with incorporation or reduction of molecular oxygen; |
|  |  |  |  |  | TraesCS3B01G609700 | Nodulin MtN21 /EamA-like transporter family protein | NA | NA | NA |
|  |  |  |  |  | TraesCS3B01G609800 | 3-oxo-5-alpha-steroid 4-dehydrogenase family protein | PF14990: Domain of unknown function (DUF4516) | IPR027858: Protein of unknown function DUF4516 | NA |
|  |  |  |  |  | TraesCS3B01G609900 | Pre-mRNA-splicing factor ISY1-like protein | PF06246: Isy1-like splicing family | IPR009360: Pre-mRNA-splicing factor Isy1 | GO:0000350 BP: generation of catalytic spliceosome for second transesterification step |
|  |  |  |  |  | TraesCS3B01G610000 | Protein CHUP1, chloroplastic | NA | NA | NA |
|  |  |  |  |  | TraesCS3B01G610100 | Pectin acetylesterase | PF03283: Pectinacetylesterase | IPR004963: Pectinacetylesterase/NOTUM; IPR029058: Alpha/Beta hydrolase fold | GO:0016787 MF: hydrolase activity |
|  |  |  |  |  | TraesCS3B01G610200 | Pectin acetylesterase | PF03283: Pectinacetylesterase | IPR004963: Pectinacetylesterase/NOTUM; IPR029058: Alpha/Beta hydrolase fold | GO:0016787 MF: hydrolase activity |
|  |  |  |  |  | TraesCS3B01G610300 | Pectin acetylesterase | PF03283: Pectinacetylesterase | IPR004963: Pectinacetylesterase/NOTUM; IPR029058: Alpha/Beta hydrolase fold | GO:0016787 MF: hydrolase activity |
|  |  |  |  |  | TraesCS3B01G610400 | Pectin acetylesterase | PF03283: Pectinacetylesterase | IPR004963: Pectinacetylesterase/NOTUM | GO:0016787 MF: hydrolase activity |
|  |  |  |  |  | TraesCS3B01G610500 | 91A protein | NA | NA | NA |
|  |  |  |  |  | TraesCS3B01G610600 | Pectin acetylesterase | PF03283: Pectinacetylesterase | IPR004963: Pectinacetylesterase/NOTUM; IPR029058: Alpha/Beta hydrolase fold | GO:0016787 MF: hydrolase activity |
|  |  |  |  |  | TraesCS3B01G610700 | B3 domain-containing protein | PF02362: B3 DNA binding domain | IPR003340: B3 DNA binding domain; IPR015300: DNA-binding pseudobarrel domain | GO:0003677 MF: DNA binding |
| STI_RSRatio | AX-111015220 |  | 3B | 829203418 | TraesCS3B01G610800 | Histone H2A | PF00125: Core histone H2A/H2B/H3/H4; PF16211: C-terminus of histone H2A | IPR002119: Histone H2A; IPR007125: Histone H2A/H2B/H3; IPR009072: Histone-fold; IPR032454: Histone H2A, C-terminal domain; IPR032458: Histone H2A conserved site | GO:0000786 CC: nucleosome;GO:0003677 MF: DNA binding;GO:0005634 CC: nucleus; |
|  |  |  |  |  | TraesCS3B01G610900 | RNA-binding protein | PF00076: RNA recognition motif. (a.k.a. RRM, RBD, or RNP domain) | IPR000504: RNA recognition motif domain | GO:0003676 MF: nucleic acid binding |
|  |  |  |  |  | TraesCS3B01G611100 | Receptor-like protein kinase | PF00069: Protein kinase domain; PF00635: MSP (Major sperm protein) domain; PF00931: NB-ARC domain | IPR000535: Major sperm protein (MSP) domain; IPR000719: Protein kinase domain; IPR002182: NB-ARC; IPR008271: Serine/threonine-protein kinase, active site; IPR008962: PapD-like; IPR011009: Protein kinase-like domain; IPR013783: Immunoglobulin-like fold; IPR027417: P-loop containing nucleoside triphosphate hydrolase | GO:0004672 MF: protein kinase activity;GO:0005524 MF: ATP binding; |
|  |  |  |  |  | TraesCS3B01G611200 | DNA topoisomerase 3 | PF13968: Domain of unknown function (DUF4220); PF04578: Protein of unknown function, DUF594 | IPR007658: Protein of unknown function DUF594; IPR025315: Domain of unknown function DUF4220 | NA |
|  |  |  |  |  | TraesCS3B01G611300 | Histone H2A | PF00125: Core histone H2A/H2B/H3/H4; PF16211: C-terminus of histone H2A | IPR002119: Histone H2A; IPR007125: Histone H2A/H2B/H3; IPR009072: Histone-fold; IPR032454: Histone H2A, C-terminal domain; IPR032458: Histone H2A conserved site | GO:0000786 CC: nucleosome;GO:0003677 MF: DNA binding; |
|  |  |  |  |  | TraesCS3B01G611400 | Outer-membrane lipoprotein LolB | NA | NA | NA |
|  |  |  |  |  | TraesCS3B01G611500 | transmembrane protein, putative (DUF594) | PF13968: Domain of unknown function (DUF4220); PF04578: Protein of unknown function, DUF594 | IPR007658: Protein of unknown function DUF594; IPR025315: Domain of unknown function DUF4220 | NA |
|  |  |  |  |  | TraesCS3B01G611600 | Soluble inorganic pyrophosphatase | PF00719: Inorganic pyrophosphatase | IPR008162: Inorganic pyrophosphatase | GO:0000287 MF: magnesium ion binding;GO:0004427 MF: inorganic diphosphatase activity; |
|  |  |  |  |  | TraesCS3B01G611700 | Kelch repeat-containing F-box family protein | PF00646: F-box domain; PF01344: Kelch motif | IPR001810: F-box domain; IPR006652: Kelch repeat type 1; IPR015915: Kelch-type beta propeller | GO:0005515 MF: protein binding |
|  |  |  |  |  | TraesCS3B01G611800 | Soluble inorganic pyrophosphatase | PF00719: Inorganic pyrophosphatase | IPR008162: Inorganic pyrophosphatase | GO:0000287 MF: magnesium ion binding;GO:0004427 MF: inorganic diphosphatase activity; |
|  |  |  |  |  | TraesCS3B01G611900 | Ubiquitin family protein | PF00240: Ubiquitin family | IPR000626: Ubiquitin domain; IPR029071: Ubiquitin-related domain | GO:0005515 MF: protein binding |
|  |  |  |  |  | TraesCS3B01G612000 | O-methyltransferase | PF08100: Dimerisation domain; PF00891: O-methyltransferase | IPR001077: O-methyltransferase, family 2; IPR011991: Winged helix-turn-helix DNA-binding domain; IPR012967: Plant methyltransferase dimerisation; IPR016461: O-methyltransferase COMT-type; IPR029063: S-adenosyl-L-methionine-dependent methyltransferase | GO:0008168 MF: methyltransferase activity;GO:0008171 MF: O-methyltransferase activity; |
|  |  |  |  |  | TraesCS3B01G612100 | Protein upstream of flc | PF06136: Domain of unknown function (DUF966) | IPR010369: Protein of unknown function DUF966; IPR021182: Uncharacterised conserved protein UCP031043 | NA |
|  |  |  |  |  | TraesCS3B01G612200 | MYB transcription factor | PF00249: Myb-like DNA-binding domain | IPR001005: SANT/Myb domain; IPR009057: Homeobox domain-like; IPR017930: Myb domain | GO:0003677 MF: DNA binding |
|  |  |  |  |  | TraesCS3B01G612300 | Transcription factor, MADS-box | PF00319: SRF-type transcription factor (DNA-binding and dimerisation domain) | IPR002100: Transcription factor, MADS-box | GO:0003677 MF: DNA binding;GO:0046983 MF: protein dimerization activity |
|  |  |  |  |  | TraesCS3B01G612400 | Transcription factor, MADS-box | PF00319: SRF-type transcription factor (DNA-binding and dimerisation domain) | IPR002100: Transcription factor, MADS-box | GO:0003677 MF: DNA binding;GO:0046983 MF: protein dimerization activity |
|  |  |  |  |  | TraesCS3B01G612500 | Transcription factor, MADS-box | PF00319: SRF-type transcription factor (DNA-binding and dimerisation domain) | IPR002100: Transcription factor, MADS-box | GO:0003677 MF: DNA binding;GO:0046983 MF: protein dimerization activity |
|  |  |  |  |  | TraesCS3B01G612600 | Transcription factor, MADS-box | PF00319: SRF-type transcription factor (DNA-binding and dimerisation domain) | IPR002100: Transcription factor, MADS-box | GO:0003677 MF: DNA binding;GO:0046983 MF: protein dimerization activity |
|  |  |  |  |  | TraesCS3B01G612700 | Transcription factor, MADS-box | PF00319: SRF-type transcription factor (DNA-binding and dimerisation domain) | IPR002100: Transcription factor, MADS-box | GO:0003677 MF: DNA binding;GO:0046983 MF: protein dimerization activity |
| STI_RSRatio | AX-158578652 |  | 3B | 829293411 | TraesCS3B01G609100 | Dof zinc finger protein | PF02701: Dof domain, zinc finger | IPR003851: Zinc finger, Dof-type | GO:0003677 MF: DNA binding;GO:0006355 BP: regulation of transcription, DNA-templated |
|  |  |  |  |  | TraesCS3B01G609200 | transmembrane protein | PF14990: Domain of unknown function (DUF4516) | IPR027858: Protein of unknown function DUF4516 | NA |
|  |  |  |  |  | TraesCS3B01G609300 | F-box protein-like protein | PF12937: F-box-like | IPR001810: F-box domain; IPR032675: Leucine-rich repeat domain, L domain-like | GO:0005515 MF: protein binding |
|  |  |  |  |  | TraesCS3B01G609400 | Cytochrome P450 | PF00067: Cytochrome P450 | IPR001128: Cytochrome P450; IPR002401: Cytochrome P450, E-class, group I; IPR017972: Cytochrome P450, conserved site | GO:0005506 MF: iron ion binding;GO:0016705 MF: oxidoreductase activity, acting on paired donors, with incorporation or reduction of molecular oxygen;GO:0020037 MF: heme binding;GO:0055114 BP: oxidation-reduction process |
|  |  |  |  |  | TraesCS3B01G609500 | Ankyrin repeat family protein | PF12796: Ankyrin repeats (3 copies); PF00023: Ankyrin repeat | IPR001841: Zinc finger, RING-type; IPR002110: Ankyrin repeat; IPR013083: Zinc finger, RING/FYVE/PHD-type; IPR020683: Ankyrin repeat-containing domain | GO:0005515 MF: protein binding;GO:0008270 MF: zinc ion binding |
|  |  |  |  |  | TraesCS3B01G609600 | Cytochrome P450 | PF00067: Cytochrome P450 | IPR001128: Cytochrome P450; IPR002401: Cytochrome P450, E-class, group I; IPR017972: Cytochrome P450, conserved site | GO:0005506 MF: iron ion binding;GO:0016705 MF: oxidoreductase activity, acting on paired donors, with incorporation or reduction of molecular oxygen;GO:0020037 MF: heme binding;GO:0055114 BP: oxidation-reduction process |
|  |  |  |  |  | TraesCS3B01G609700 | Nodulin MtN21 /EamA-like transporter family protein | NA | NA | NA |
|  |  |  |  |  | TraesCS3B01G609800 | 3-oxo-5-alpha-steroid 4-dehydrogenase family protein | PF14990: Domain of unknown function (DUF4516) | IPR027858: Protein of unknown function DUF4516 | NA |
|  |  |  |  |  | TraesCS3B01G609900 | Pre-mRNA-splicing factor ISY1-like protein | PF06246: Isy1-like splicing family | IPR009360: Pre-mRNA-splicing factor Isy1 | GO:0000350 BP: generation of catalytic spliceosome for second transesterification step |
|  |  |  |  |  | TraesCS3B01G610000 | Protein CHUP1, chloroplastic | NA | NA | NA |
|  |  |  |  |  | TraesCS3B01G610100 | Pectin acetylesterase | PF03283: Pectinacetylesterase | IPR004963: Pectinacetylesterase/NOTUM; IPR029058: Alpha/Beta hydrolase fold | GO:0016787 MF: hydrolase activity |
|  |  |  |  |  | TraesCS3B01G610200 | Pectin acetylesterase | PF03283: Pectinacetylesterase | IPR004963: Pectinacetylesterase/NOTUM; IPR029058: Alpha/Beta hydrolase fold | GO:0016787 MF: hydrolase activity |
|  |  |  |  |  | TraesCS3B01G610300 | Pectin acetylesterase | PF03283: Pectinacetylesterase | IPR004963: Pectinacetylesterase/NOTUM; IPR029058: Alpha/Beta hydrolase fold | GO:0016787 MF: hydrolase activity |
|  |  |  |  |  | TraesCS3B01G610400 | Pectin acetylesterase | PF03283: Pectinacetylesterase | IPR004963: Pectinacetylesterase/NOTUM | GO:0016787 MF: hydrolase activity |
|  |  |  |  |  | TraesCS3B01G610500 | 91A protein | NA | NA | NA |
|  |  |  |  |  | TraesCS3B01G610600 | Pectin acetylesterase | PF03283: Pectinacetylesterase | IPR004963: Pectinacetylesterase/NOTUM; IPR029058: Alpha/Beta hydrolase fold | GO:0016787 MF: hydrolase activity |
|  |  |  |  |  | TraesCS3B01G610700 | B3 domain-containing protein | PF02362: B3 DNA binding domain | IPR003340: B3 DNA binding domain; IPR015300: DNA-binding pseudobarrel domain | GO:0003677 MF: DNA binding |
|  |  |  |  |  | TraesCS3B01G610800 | Histone H2A | PF00125: Core histone H2A/H2B/H3/H4; PF16211: C-terminus of histone H2A | IPR002119: Histone H2A; IPR007125: Histone H2A/H2B/H3; IPR009072: Histone-fold; IPR032454: Histone H2A, C-terminal domain; IPR032458: Histone H2A conserved site | GO:0000786 CC: nucleosome;GO:0003677 MF: DNA binding; |
|  |  |  |  |  | TraesCS3B01G610900 | RNA-binding protein | PF00076: RNA recognition motif. (a.k.a. RRM, RBD, or RNP domain) | IPR000504: RNA recognition motif domain | GO:0003676 MF: nucleic acid binding |
|  |  |  |  |  | TraesCS3B01G611100 | Receptor-like protein kinase | PF00069: Protein kinase domain; PF00635: MSP (Major sperm protein) domain; PF00931: NB-ARC domain | IPR000535: Major sperm protein (MSP) domain; IPR000719: Protein kinase domain; IPR002182: NB-ARC; IPR008271: Serine/threonine-protein kinase, active site; IPR008962: PapD-like; IPR011009: Protein kinase-like domain; IPR013783: Immunoglobulin-like fold; IPR027417: P-loop containing nucleoside triphosphate hydrolase | GO:0004672 MF: protein kinase activity;GO:0005524 MF: ATP binding; |
|  |  |  |  |  | TraesCS3B01G611200 | DNA topoisomerase 3 | PF13968: Domain of unknown function (DUF4220); PF04578: Protein of unknown function, DUF594 | IPR007658: Protein of unknown function DUF594; IPR025315: Domain of unknown function DUF4220 | NA |
|  |  |  |  |  | TraesCS3B01G611300 | Histone H2A | PF00125: Core histone H2A/H2B/H3/H4; PF16211: C-terminus of histone H2A | IPR002119: Histone H2A; IPR007125: Histone H2A/H2B/H3; IPR009072: Histone-fold; IPR032454: Histone H2A, C-terminal domain; IPR032458: Histone H2A conserved site | GO:0000786 CC: nucleosome;GO:0003677 MF: DNA binding; |
|  |  |  |  |  | TraesCS3B01G611400 | Outer-membrane lipoprotein LolB | NA | NA | NA |
|  |  |  |  |  | TraesCS3B01G611500 | transmembrane protein, putative (DUF594) | PF13968: Domain of unknown function (DUF4220); PF04578: Protein of unknown function, DUF594 | IPR007658: Protein of unknown function DUF594; IPR025315: Domain of unknown function DUF4220 | NA |
|  |  |  |  |  | TraesCS3B01G611600 | Soluble inorganic pyrophosphatase | PF00719: Inorganic pyrophosphatase | IPR008162: Inorganic pyrophosphatase | GO:0000287 MF: magnesium ion binding;GO:0004427 MF: inorganic diphosphatase activity; |
|  |  |  |  |  | TraesCS3B01G611700 | Kelch repeat-containing F-box family protein | PF00646: F-box domain; PF01344: Kelch motif | IPR001810: F-box domain; IPR006652: Kelch repeat type 1; IPR015915: Kelch-type beta propeller | GO:0005515 MF: protein binding |
|  |  |  |  |  | TraesCS3B01G611800 | Soluble inorganic pyrophosphatase | PF00719: Inorganic pyrophosphatase | IPR008162: Inorganic pyrophosphatase | GO:0000287 MF: magnesium ion binding;GO:0004427 MF: inorganic diphosphatase activity; |
|  |  |  |  |  | TraesCS3B01G611900 | Ubiquitin family protein | PF00240: Ubiquitin family | IPR000626: Ubiquitin domain; IPR029071: Ubiquitin-related domain | GO:0005515 MF: protein binding |
|  |  |  |  |  | TraesCS3B01G612000 | O-methyltransferase | PF08100: Dimerisation domain; PF00891: O-methyltransferase | IPR001077: O-methyltransferase, family 2; IPR011991: Winged helix-turn-helix DNA-binding domain; IPR012967: Plant methyltransferase dimerisation; IPR016461: O-methyltransferase COMT-type; IPR029063: S-adenosyl-L-methionine-dependent methyltransferase | GO:0008168 MF: methyltransferase activity;GO:0008171 MF: O-methyltransferase activity;G |
|  |  |  |  |  | TraesCS3B01G612100 | Protein upstream of flc | PF06136: Domain of unknown function (DUF966) | IPR010369: Protein of unknown function DUF966; IPR021182: Uncharacterised conserved protein UCP031043 | NA |
|  |  |  |  |  | TraesCS3B01G612200 | NA | NA | NA | NA |
|  |  |  |  |  | TraesCS3B01G612200 | MYB transcription factor | PF00249: Myb-like DNA-binding domain | IPR001005: SANT/Myb domain; IPR009057: Homeobox domain-like; IPR017930: Myb domain | GO:0003677 MF: DNA binding |
|  |  |  |  |  | TraesCS3B01G612300 | Transcription factor, MADS-box | PF00319: SRF-type transcription factor (DNA-binding and dimerisation domain) | IPR002100: Transcription factor, MADS-box | GO:0003677 MF: DNA binding;GO:0046983 MF: protein dimerization activity |
|  |  |  |  |  | TraesCS3B01G612400 | Transcription factor, MADS-box | PF00319: SRF-type transcription factor (DNA-binding and dimerisation domain) | IPR002100: Transcription factor, MADS-box | GO:0003677 MF: DNA binding;GO:0046983 MF: protein dimerization activity |
|  |  |  |  |  | TraesCS3B01G612500 | Transcription factor, MADS-box | PF00319: SRF-type transcription factor (DNA-binding and dimerisation domain) | IPR002100: Transcription factor, MADS-box | GO:0003677 MF: DNA binding;GO:0046983 MF: protein dimerization activity |
|  |  |  |  |  | TraesCS3B01G612600 | Transcription factor, MADS-box | PF00319: SRF-type transcription factor (DNA-binding and dimerisation domain) | IPR002100: Transcription factor, MADS-box | GO:0003677 MF: DNA binding;GO:0046983 MF: protein dimerization activity |
|  |  |  |  |  | TraesCS3B01G612700 | Transcription factor, MADS-box | PF00319: SRF-type transcription factor (DNA-binding and dimerisation domain) | IPR002100: Transcription factor, MADS-box | GO:0003677 MF: DNA binding;GO:0046983 MF: protein dimerization activity |
| STI_SL and | BS00065603_51 |  | 3D | 611497215 | TraesCS3D01G537400 | Dof zinc finger protein | PF02701: Dof domain, zinc finger | IPR003851: Zinc finger, Dof-type | GO:0003677 MF: DNA binding;GO:0006355 BP: regulation of transcription, DNA-templated |
| STI_RSRatio |  |  |  |  | TraesCS3D01G537500 | Dof zinc finger protein | PF02701: Dof domain, zinc finger | IPR003851: Zinc finger, Dof-type | GO:0003677 MF: DNA binding;GO:0006355 BP: regulation of transcription, DNA-templated |
|  |  |  |  |  | TraesCS3D01G537600 | 3-oxo-5-alpha-steroid 4-dehydrogenase family protein | PF14990: Domain of unknown function (DUF4516) | IPR027858: Protein of unknown function DUF4516 | NA |
|  |  |  |  |  | TraesCS3D01G537700 | F-box protein-like protein | PF12937: F-box-like | IPR001810: F-box domain; IPR032675: Leucine-rich repeat domain, L domain-like | GO:0005515 MF: protein binding |
|  |  |  |  |  | TraesCS3D01G537800 | Cytochrome P450 | PF00067: Cytochrome P450 | IPR001128: Cytochrome P450; IPR002401: Cytochrome P450, E-class, group I; IPR017972: Cytochrome P450, conserved site | GO:0005506 MF: iron ion binding;GO:0016705 MF: oxidoreductase activity, acting on paired donors, with incorporation or reduction of molecular oxygen; |
|  |  |  |  |  | TraesCS3D01G537900 | Ankyrin repeat family protein | PF12796: Ankyrin repeats (3 copies); PF00023: Ankyrin repeat | IPR001841: Zinc finger, RING-type; IPR002110: Ankyrin repeat; IPR013083: Zinc finger, RING/FYVE/PHD-type; IPR020683: Ankyrin repeat-containing domain | GO:0005515 MF: protein binding;GO:0008270 MF: zinc ion binding |
|  |  |  |  |  | TraesCS3D01G538000 | Protein CHUP1, chloroplastic | NA | NA | NA |
|  |  |  |  |  | TraesCS3D01G538100 | P-glycoprotein 6 | NA | NA | NA |
|  |  |  |  |  | TraesCS3D01G538200 | Myb/SANT-like DNA-binding domain protein | NA | NA | NA |
|  |  |  |  |  | TraesCS3D01G538300 | Protein kinase family protein | PF13947: Wall-associated receptor kinase galacturonan-binding; PF07645: Calcium-binding EGF domain; PF00069: Protein kinase domain | IPR000152: EGF-type aspartate/asparagine hydroxylation site; IPR000719: Protein kinase domain; IPR000742: EGF-like domain; IPR001881: EGF-like calcium-binding domain; IPR008271: Serine/threonine-protein kinase, active site; IPR009030: Growth factor receptor cysteine-rich domain; IPR011009: Protein kinase-like domain; IPR017441: Protein kinase, ATP binding site; IPR018097: EGF-like calcium-binding, conserved site; IPR025287: Wall-associated receptor kinase, galacturonan-binding domain | GO:0004672 MF: protein kinase activity;GO:0005509 MF: calcium ion binding; |
|  |  |  |  |  | TraesCS3D01G538400 | Pectin acetylesterase | PF03283: Pectinacetylesterase | IPR004963: Pectinacetylesterase/NOTUM | GO:0016787 MF: hydrolase activity |
|  |  |  |  |  | TraesCS3D01G538500 | Pectin acetylesterase | PF03283: Pectinacetylesterase | IPR004963: Pectinacetylesterase/NOTUM; IPR029058: Alpha/Beta hydrolase fold | GO:0016787 MF: hydrolase activity |
|  |  |  |  |  | TraesCS3D01G538600 | Pectin acetylesterase | PF03283: Pectinacetylesterase | IPR004963: Pectinacetylesterase/NOTUM; IPR029058: Alpha/Beta hydrolase fold | GO:0016787 MF: hydrolase activity |
|  |  |  |  |  | TraesCS3D01G538700 | Pectin acetylesterase | PF03283: Pectinacetylesterase | IPR004963: Pectinacetylesterase/NOTUM; IPR029058: Alpha/Beta hydrolase fold | GO:0016787 MF: hydrolase activity |
|  |  |  |  |  | TraesCS3D01G538800 | Pectin acetylesterase | PF03283: Pectinacetylesterase | IPR004963: Pectinacetylesterase/NOTUM; IPR029058: Alpha/Beta hydrolase fold | GO:0016787 MF: hydrolase activity |
|  |  |  |  |  | TraesCS3D01G538900 | Pectin acetylesterase | PF03283: Pectinacetylesterase | IPR004963: Pectinacetylesterase/NOTUM | GO:0016787 MF: hydrolase activity |
|  |  |  |  |  | TraesCS3D01G539000 | 91A protein | NA | NA | NA |
|  |  |  |  |  | TraesCS3D01G539100 | Pectin acetylesterase | PF03283: Pectinacetylesterase | IPR004963: Pectinacetylesterase/NOTUM | GO:0016787 MF: hydrolase activity |
|  |  |  |  |  | TraesCS3D01G539200 | B3 domain-containing protein | PF02362: B3 DNA binding domain | IPR003340: B3 DNA binding domain; IPR015300: DNA-binding pseudobarrel domain | GO:0003677 MF: DNA binding |
|  |  |  |  |  | TraesCS3D01G539300 | Histone H2A | PF00125: Core histone H2A/H2B/H3/H4; PF16211: C-terminus of histone H2A | IPR002119: Histone H2A; IPR007125: Histone H2A/H2B/H3; IPR009072: Histone-fold; IPR032454: Histone H2A, C-terminal domain; IPR032458: Histone H2A conserved site | GO:0000786 CC: nucleosome;GO:0003677 MF: DNA binding; |
|  |  |  |  |  | TraesCS3D01G539400 | RNA-binding protein | PF00076: RNA recognition motif. (a.k.a. RRM, RBD, or RNP domain) | IPR000504: RNA recognition motif domain | GO:0003676 MF: nucleic acid binding |
|  |  |  |  |  | TraesCS3D01G539500 | Histone H2A | PF00125: Core histone H2A/H2B/H3/H4; PF16211: C-terminus of histone H2A | IPR002119: Histone H2A; IPR007125: Histone H2A/H2B/H3; IPR009072: Histone-fold; IPR032454: Histone H2A, C-terminal domain | GO:0000786 CC: nucleosome;GO:0003677 MF: DNA binding; |
|  |  |  |  |  | TraesCS3D01G539600 | transmembrane protein, putative (DUF594) | PF13968: Domain of unknown function (DUF4220); PF04578: Protein of unknown function, DUF594 | IPR007658: Protein of unknown function DUF594; IPR025315: Domain of unknown function DUF4220 | NA |
|  |  |  |  |  | TraesCS3D01G539700 | Histone H2A | PF00125: Core histone H2A/H2B/H3/H4; PF16211: C-terminus of histone H2A | IPR002119: Histone H2A; IPR007125: Histone H2A/H2B/H3; IPR009072: Histone-fold; IPR032454: Histone H2A, C-terminal domain; IPR032458: Histone H2A conserved site | GO:0000786 CC: nucleosome;GO:0003677 MF: DNA binding; |
|  |  |  |  |  | TraesCS3D01G539800 | Hexose transporter HXT14 | NA | NA | NA |
|  |  |  |  |  | TraesCS3D01G539900 | Soluble inorganic pyrophosphatase | PF00719: Inorganic pyrophosphatase | IPR008162: Inorganic pyrophosphatase | GO:0000287 MF: magnesium ion binding;GO:0004427 MF: inorganic diphosphatase activity; |
|  |  |  |  |  | TraesCS3D01G540000 | Kelch repeat-containing F-box family protein | PF00646: F-box domain; PF01344: Kelch motif | IPR001810: F-box domain; IPR006652: Kelch repeat type 1; IPR015915: Kelch-type beta propeller | GO:0005515 MF: protein binding |
|  |  |  |  |  | TraesCS3D01G540100 | Soluble inorganic pyrophosphatase | PF00719: Inorganic pyrophosphatase | IPR008162: Inorganic pyrophosphatase | GO:0000287 MF: magnesium ion binding;GO:0004427 MF: inorganic diphosphatase activity; |
|  |  |  |  |  | TraesCS3D01G540200 | O-methyltransferase | PF08100: Dimerisation domain; PF00891: O-methyltransferase | IPR001077: O-methyltransferase, family 2; IPR011991: Winged helix-turn-helix DNA-binding domain; IPR012967: Plant methyltransferase dimerisation; IPR016461: O-methyltransferase COMT-type; IPR029063: S-adenosyl-L-methionine-dependent methyltransferase | GO:0008168 MF: methyltransferase activity;GO:0008171 MF: O-methyltransferase activity |
|  |  |  |  |  | TraesCS3D01G540300 | Eukaryotic translation initiation factor 3 subunit D | NA | NA | NA |
|  |  |  |  |  | TraesCS3D01G540400 | Ankyrin repeat family protein | PF13962: Domain of unknown function | IPR026961: PGG domain | NA |
|  |  |  |  |  | TraesCS3D01G540500 | Protein upstream of flc | PF06136: Domain of unknown function (DUF966) | IPR010369: Protein of unknown function DUF966; IPR021182: Uncharacterised conserved protein UCP031043 | NA |
|  |  |  |  |  | TraesCS3D01G540600 | MYB transcription factor | PF00249: Myb-like DNA-binding domain | IPR001005: SANT/Myb domain; IPR009057: Homeobox domain-like; IPR017930: Myb domain | GO:0003677 MF: DNA binding |
|  |  |  |  |  | TraesCS3D01G540700 | MADS-box transcription factor | PF00319: SRF-type transcription factor (DNA-binding and dimerisation domain) | IPR002100: Transcription factor, MADS-box | GO:0003677 MF: DNA binding;GO:0046983 MF: protein dimerization activity |
|  |  |  |  |  | TraesCS3D01G540800 | DnaJ homolog subfamily C member 14 | NA | NA | NA |
|  |  |  |  |  | TraesCS3D01G540900 | Aquaporin | PF00230: Major intrinsic protein | IPR000425: Major intrinsic protein; IPR022357: Major intrinsic protein, conserved site; IPR023271: Aquaporin-like | GO:0005215 MF: transporter activity;GO:0006810 BP: transport;GO:0016020 CC: membrane |
|  |  |  |  |  | TraesCS3D01G541000 | GDSL esterase/lipase | PF00657: GDSL-like Lipase/Acylhydrolase | IPR001087: GDSL lipase/esterase; IPR013830: SGNH hydrolase-type esterase domain | GO:0016788 MF: hydrolase activity, acting on ester bonds |
|  |  |  |  |  | TraesCS3D01G541100 | TOX high mobility group box protein, putative (DUF1635) | PF07795: Protein of unknown function (DUF1635) | IPR012862: Protein of unknown function DUF1635 | NA |
|  |  |  |  |  | TraesCS3D01G541200 | Vacuolar fusion protein MON1 | PF03164: Trafficking protein Mon1 | IPR004353: Vacuolar fusion protein Mon1 | NA |
|  |  |  |  |  | TraesCS3D01G541300 | Enoyl-[acyl-carrier-protein] reductase [NADH] | NA | NA | NA |
| STI_SL and | BS00068415_51 |  | 3D | 612903461 | TraesCS3D01G541400 | Regulator of chromosome condensation (RCC1) family with FYVE zinc finger domain-containing protein | NA | NA | NA |
| STI_RSRatio |  |  |  |  | TraesCS3D01G541500 | Disease resistance protein RPP13 | NA | NA | NA |
|  |  |  |  |  | TraesCS3D01G541600 | Disease resistance protein (NBS-LRR class) family | PF00931: NB-ARC domain | IPR002182: NB-ARC; IPR027417: P-loop containing nucleoside triphosphate hydrolase | GO:0043531 MF: ADP binding |
|  |  |  |  |  | TraesCS3D01G541700 | ABC transporter B family protein | PF00664: ABC transporter transmembrane region; PF00005: ABC transporter | IPR003439: ABC transporter-like; IPR003593: AAA+ ATPase domain; IPR011527: ABC transporter type 1, transmembrane domain; IPR027417: P-loop containing nucleoside triphosphate hydrolase | GO:0005524 MF: ATP binding;GO:0006810 BP: transport; |
|  |  |  |  |  | TraesCS3D01G541800 | Chaperone protein dnaJ | PF00226: DnaJ domain; PF11926: Domain of unknown function (DUF3444) | IPR001623: DnaJ domain; IPR024593: Domain of unknown function DUF3444 | NA |
|  |  |  |  |  | TraesCS3D01G541900 | Plant/T31B5-30 protein | PF11443: Domain of unknown function (DUF2828) | IPR011205: Uncharacterised conserved protein UCP015417, vWA; IPR024553: Domain of unknown function DUF2828 | NA |
|  |  |  |  |  | TraesCS3D01G542000 | Pectinesterase inhibitor | PF04043: Plant invertase/pectin methylesterase inhibitor | IPR006501: Pectinesterase inhibitor domain | GO:0004857 MF: enzyme inhibitor activity |
|  |  |  |  |  | TraesCS3D01G542100 | SNARE-interacting protein KEULE | PF00995: Sec1 family | IPR001619: Sec1-like protein | GO:0006904 BP: vesicle docking involved in exocytosis;GO:0016192 BP: vesicle-mediated transport |
|  |  |  |  |  | TraesCS3D01G542200 | Extra-large guanine nucleotide binding family protein | NA | IPR013083: Zinc finger, RING/FYVE/PHD-type | NA |
|  |  |  |  |  | TraesCS3D01G542300 | Protein kinase-like protein | PF08263: Leucine rich repeat N-terminal domain; PF13855: Leucine rich repeat; PF00069: Protein kinase domain | IPR000719: Protein kinase domain; IPR001611: Leucine-rich repeat; IPR003591: Leucine-rich repeat, typical subtype; IPR008271: Serine/threonine-protein kinase, active site; IPR011009: Protein kinase-like domain; IPR013210: Leucine-rich repeat-containing N-terminal, plant-type; IPR017441: Protein kinase, ATP binding site; IPR032675: Leucine-rich repeat domain, L domain-like | GO:0004672 MF: protein kinase activity;GO:0005515 MF: protein binding; |
|  |  |  |  |  | TraesCS3D01G542400 | lectin-receptor kinase | PF00069: Protein kinase domain | IPR000719: Protein kinase domain; IPR008271: Serine/threonine-protein kinase, active site; IPR011009: Protein kinase-like domain; IPR017441: Protein kinase, ATP binding site | GO:0004672 MF: protein kinase activity;GO:0005524 MF: ATP binding; |
|  |  |  |  |  | TraesCS3D01G542500 | Disease resistance protein (TIR-NBS-LRR class) family | NA | IPR032675: Leucine-rich repeat domain, L domain-like | NA |
|  |  |  |  |  | TraesCS3D01G542600 | Plant cadmium resistance protein | PF11204: Protein of unknown function (DUF2985); PF04749: PLAC8 family | IPR006461: PLAC8 motif-containing protein; IPR021369: Protein of unknown function DUF2985 | NA |
|  |  |  |  |  | TraesCS3D01G542700 | Dirigent protein | PF03018: Dirigent-like protein | IPR004265: Plant disease resistance response protein | NA |
|  |  |  |  |  | TraesCS3D01G542800 | Gibberellin 2-beta-dioxygenase | PF14226: non-haem dioxygenase in morphine synthesis N-terminal; PF03171: 2OG-Fe(II) oxygenase superfamily | IPR005123: Oxoglutarate/iron-dependent dioxygenase; IPR026992: Non-haem dioxygenase N-terminal domain; IPR027443: Isopenicillin N synthase-like | GO:0016491 MF: oxidoreductase activity;GO:0055114 BP: oxidation-reduction process |
|  |  |  |  |  | TraesCS3D01G542900 | Dirigent protein | PF03018: Dirigent-like protein | IPR004265: Plant disease resistance response protein | NA |
|  |  |  |  |  | TraesCS3D01G543000 | transmembrane protein, putative (DUF594) | PF13968: Domain of unknown function (DUF4220); PF04578: Protein of unknown function, DUF594 | IPR007658: Protein of unknown function DUF594; IPR025315: Domain of unknown function DUF4220 | NA |
|  |  |  |  |  | TraesCS3D01G543100 | Chaperone protein dnaJ, putative | PF13432: Tetratricopeptide repeat; PF13181: Tetratricopeptide repeat; PF00226: DnaJ domain | IPR001623: DnaJ domain; IPR011990: Tetratricopeptide-like helical domain; IPR013026: Tetratricopeptide repeat-containing domain; IPR018253: DnaJ domain, conserved site; IPR019734: Tetratricopeptide repeat | GO:0005515 MF: protein binding |
|  |  |  |  |  | TraesCS3D01G543200 | SKP1-like protein | NA | IPR001232: S-phase kinase-associated protein 1-like; IPR011333: SKP1/BTB/POZ domain; IPR016072: SKP1 component, dimerisation | GO:0006511 BP: ubiquitin-dependent protein catabolic process |
|  |  |  |  |  | TraesCS3D01G543300 | Kinase family protein | PF00069: Protein kinase domain | IPR000719: Protein kinase domain; IPR008271: Serine/threonine-protein kinase, active site; IPR011009: Protein kinase-like domain; IPR017441: Protein kinase, ATP binding site | GO:0004672 MF: protein kinase activity;GO:0005524 MF: ATP binding;GO:0006468 BP: protein phosphorylation |
|  |  |  |  |  | TraesCS3D01G543400 | E3 SUMO-protein ligase SIZ1 | PF02891: MIZ/SP-RING zinc finger | IPR001965: Zinc finger, PHD-type; IPR003034: SAP domain; IPR004181: Zinc finger, MIZ-type; IPR011011: Zinc finger, FYVE/PHD-type; IPR013083: Zinc finger, RING/FYVE/PHD-type; IPR019786: Zinc finger, PHD-type, conserved site | GO:0005515 MF: protein binding;GO:0008270 MF: zinc ion binding |
|  |  |  |  |  | TraesCS3D01G543500 | Protein IQ-DOMAIN 1 | PF00612: IQ calmodulin-binding motif; PF13178: Protein of unknown function (DUF4005) | IPR000048: IQ motif, EF-hand binding site; IPR025064: Domain of unknown function DUF4005 | GO:0005515 MF: protein binding |
|  |  |  |  |  | TraesCS3D01G543600 | SKP1-like protein | PF03931: Skp1 family, tetramerisation domain; PF01466: Skp1 family, dimerisation domain | IPR001232: S-phase kinase-associated protein 1-like; IPR011333: SKP1/BTB/POZ domain; IPR016072: SKP1 component, dimerisation; IPR016073: SKP1 component, POZ domain | GO:0006511 BP: ubiquitin-dependent protein catabolic process |
|  |  |  |  |  | TraesCS3D01G543700 | Bifunctional lycopene cyclase/phytoene synthase | NA | NA | NA |
|  |  |  |  |  | TraesCS3D01G543800 | Serine/threonine-protein kinase ATM | PF00855: PWWP domain | IPR000313: PWWP domain; IPR000504: RNA recognition motif domain | GO:0003676 MF: nucleic acid binding |
|  |  |  |  |  | TraesCS3D01G543900 | Kinase-like protein | PF12819: Carbohydrate-binding protein of the ER; PF07714: Protein tyrosine kinase | IPR000719: Protein kinase domain; IPR001245: Serine-threonine/tyrosine-protein kinase, catalytic domain; IPR008271: Serine/threonine-protein kinase, active site; IPR011009: Protein kinase-like domain; IPR017441: Protein kinase, ATP binding site; IPR024788: Malectin-like carbohydrate-binding domain; IPR032675: Leucine-rich repeat domain, L domain-like | GO:0004672 MF: protein kinase activity;GO:0005524 MF: ATP binding;GO:0006468 BP: protein phosphorylation |
|  |  |  |  |  | TraesCS3D01G544000 | GATA transcription factor, putative | PF00320: GATA zinc finger | IPR000679: Zinc finger, GATA-type; IPR013088: Zinc finger, NHR/GATA-type | GO:0003700 MF: transcription factor activity, sequence-specific DNA binding;GO:0006355 BP: regulation of transcription, DNA-templated;GO:0008270 MF: zinc ion binding; |
|  |  |  |  |  | TraesCS3D01G544100 | Phosphatase 2C family protein | PF00481: Protein phosphatase 2C | IPR000222: PPM-type phosphatase, divalent cation binding; IPR001932: PPM-type phosphatase domain | GO:0003824 MF: catalytic activity;GO:0043169 MF: cation binding |
|  |  |  |  |  | TraesCS3D01G544200 | non-photochemical quenching 1 | NA | NA | NA |
|  |  |  |  |  | TraesCS3D01G544300 | transmembrane protein, putative (DUF594) | PF13968: Domain of unknown function (DUF4220); PF04578: Protein of unknown function, DUF594 | IPR007658: Protein of unknown function DUF594; IPR025315: Domain of unknown function DUF4220 | NA |
| STI_RL | AX-158581925 |  | 4A | 43031174 | TraesCS4A01G051300 | Cyclin family protein | PF00134: Cyclin, N-terminal domain; PF02984: Cyclin, C-terminal domain | IPR004367: Cyclin, C-terminal domain; IPR006671: Cyclin, N-terminal; IPR013763: Cyclin-like | GO:0005634 CC: nucleus |
|  |  |  |  |  | TraesCS4A01G051400 | Mannose-1-phosphate guanyltransferase | PF00483: Nucleotidyl transferase | IPR005835: Nucleotidyl transferase domain; IPR029044: Nucleotide-diphospho-sugar transferases | GO:0009058 BP: biosynthetic process;GO:0016779 MF: nucleotidyltransferase activity |
|  |  |  |  |  | TraesCS4A01G051500 | RAN GTPase-activating protein 2 | PF13943: WPP domain | IPR025265: WPP domain | NA |
|  |  |  |  |  | TraesCS4A01G051600 | Protein transport protein GOT1 | PF04178: Got1/Sft2-like family | IPR007305: Vesicle transport protein, Got1/SFT2-like | GO:0016192 BP: vesicle-mediated transport |
|  |  |  |  |  | TraesCS4A01G051700 | S-acyltransferase | PF01529: DHHC palmitoyltransferase | IPR001594: Palmitoyltransferase, DHHC domain | NA |
|  |  |  |  |  | TraesCS4A01G051800 | Myb/SANT-like DNA-binding domain protein | NA | NA | NA |
|  |  |  |  |  | TraesCS4A01G051900 | Methionine--tRNA ligase | PF00133: tRNA synthetases class I (I, L, M and V); PF09334: tRNA synthetases class I (M) | IPR002300: Aminoacyl-tRNA synthetase, class Ia; IPR009080: Aminoacyl-tRNA synthetase, class Ia, anticodon-binding; IPR014729: Rossmann-like alpha/beta/alpha sandwich fold; IPR014758: Methionyl-tRNA synthetase; IPR015413: Methionyl/Leucyl tRNA synthetase; IPR023457: Methionine-tRNA synthetase, type 2; IPR033911: Methioninyl-tRNA synthetase core domain | GO:0000166 MF: nucleotide binding;GO:0004812 MF: aminoacyl-tRNA ligase activity; |
|  |  |  |  |  | TraesCS4A01G052000 | Cysteine proteinase inhibitor | PF16845: Aspartic acid proteinase inhibitor | IPR000010: Cystatin domain; IPR018073: Proteinase inhibitor I25, cystatin, conserved site | GO:0004869 MF: cysteine-type endopeptidase inhibitor activity |
|  |  |  |  |  | TraesCS4A01G052100 | Cysteine proteinase inhibitor | PF16845: Aspartic acid proteinase inhibitor | IPR000010: Cystatin domain | GO:0004869 MF: cysteine-type endopeptidase inhibitor activity |
|  |  |  |  |  | TraesCS4A01G052200 | Beta-xylosidase, putative | PF00933: Glycosyl hydrolase family 3 N terminal domain; PF01915: Glycosyl hydrolase family 3 C-terminal domain; PF14310: Fibronectin type III-like domain | IPR001764: Glycoside hydrolase, family 3, N-terminal; IPR002772: Glycoside hydrolase family 3 C-terminal domain; IPR013783: Immunoglobulin-like fold; IPR017853: Glycoside hydrolase superfamily; IPR026891: Fibronectin type III-like domain | GO:0004553 MF: hydrolase activity, hydrolyzing O-glycosyl compounds;GO:0005975 BP: carbohydrate metabolic process |
|  |  |  |  |  | TraesCS4A01G052300 | Protein weak chloroplast movement under blue light 1 | NA | NA | NA |
|  |  |  |  |  | TraesCS4A01G052400 | DUF538 family protein (Protein of unknown function, DUF538) | PF04398: Protein of unknown function, DUF538 | IPR007493: Protein of unknown function DUF538 | NA |
|  |  |  |  |  | TraesCS4A01G052500 | Nodulin-related protein 1, putative | NA | NA | NA |
|  |  |  |  |  | TraesCS4A01G052600 | Acetyl-CoA decarbonylase/synthase complex subunit alpha 1 | NA | NA | NA |
|  |  |  |  |  | TraesCS4A01G052700 | Poly(A) RNA polymerase cid14 | NA | NA | NA |
| STI_RSRatio | AX-158617434 |  | 4A | 18119033 | TraesCS4A01G024900 | CASP-like protein | PF04535: Domain of unknown function (DUF588) | IPR006702: Domain of unknown function DUF588 | NA |
|  |  |  |  |  | TraesCS4A01G025000 | Fimbria adhesin EcpD | NA | NA | NA |
|  |  |  |  |  | TraesCS4A01G025100 | Exocyst complex component SEC15A | PF04091: Exocyst complex subunit Sec15-like | IPR007225: Exocyst complex component EXOC6/Sec15 | GO:0000145 CC: exocyst;GO:0006904 BP: vesicle docking involved in exocytosis |
|  |  |  |  |  | TraesCS4A01G025200 | Glutathione reductase | PF07992: Pyridine nucleotide-disulphide oxidoreductase; PF02852: Pyridine nucleotide-disulphide oxidoreductase, dimerisation domain | IPR004099: Pyridine nucleotide-disulphide oxidoreductase, dimerisation domain; IPR006324: Glutathione-disulphide reductase; IPR012999: Pyridine nucleotide-disulphide oxidoreductase, class I, active site; IPR016156: FAD/NAD-linked reductase, dimerisation domain; IPR023753: FAD/NAD(P)-binding domain | GO:0004362 MF: glutathione-disulfide reductase activity;GO:0006749 BP: glutathione metabolic process; |
|  |  |  |  |  | TraesCS4A01G025300 | 3-ketoacyl-CoA synthase | PF08392: FAE1/Type III polyketide synthase-like protein; PF08541: 3-Oxoacyl-[acyl-carrier-protein (ACP)] synthase III C terminal | IPR012392: Very-long-chain 3-ketoacyl-CoA synthase; IPR013601: FAE1/Type III polyketide synthase-like protein; IPR013747: 3-Oxoacyl-[acyl-carrier-protein (ACP)] synthase III, C-terminal; IPR016039: Thiolase-like | GO:0003824 MF: catalytic activity;GO:0006633 BP: fatty acid biosynthetic process; |
|  |  |  |  |  | TraesCS4A01G025400 | Zinc transporter protein | PF02535: ZIP Zinc transporter | IPR003689: Zinc/iron permease; IPR004698: Zinc/iron permease, fungal/plant | GO:0005385 MF: zinc ion transmembrane transporter activity;GO:0016020 CC: membrane; |
|  |  |  |  |  | TraesCS4A01G025500 | DUF506 family protein | PF04720: PDDEXK-like family of unknown function | IPR006502: Protein of unknown function PDDEXK-like | NA |
|  |  |  |  |  | TraesCS4A01G025700 | Serine protease HTRA1 | PF13365: Trypsin-like peptidase domain | IPR009003: Peptidase S1, PA clan | NA |
|  |  |  |  |  | TraesCS4A01G025800 | NA | NA | NA | NA |
|  |  |  |  |  | TraesCS4A01G025900 | Serine protease HtrA-like | PF00595: PDZ domain (Also known as DHR or GLGF) | IPR001478: PDZ domain | GO:0005515 MF: protein binding |
|  |  |  |  |  | TraesCS4A01G026000 | Werner Syndrome-like exonuclease | PF01612: 3'-5' exonuclease | IPR002562: 3'-5' exonuclease domain; IPR012337: Ribonuclease H-like domain | GO:0003676 MF: nucleic acid binding;GO:0006139 BP: nucleobase-containing compound metabolic process;G |
|  |  |  |  |  | TraesCS4A01G026100 | receptor kinase 1 | PF00069: Protein kinase domain | IPR000719: Protein kinase domain; IPR008271: Serine/threonine-protein kinase, active site; IPR011009: Protein kinase-like domain; IPR017441: Protein kinase, ATP binding site | GO:0004672 MF: protein kinase activity;GO:0005524 MF: ATP binding; |
|  |  |  |  |  | TraesCS4A01G026200 | Serine/threonine-protein kinase | PF01453: D-mannose binding lectin; PF00069: Protein kinase domain | IPR000719: Protein kinase domain; IPR001480: Bulb-type lectin domain; IPR003609: PAN/Apple domain; IPR008271: Serine/threonine-protein kinase, active site; IPR011009: Protein kinase-like domain; IPR017441: Protein kinase, ATP binding site; IPR024171: S-receptor-like serine/threonine-protein kinase | GO:0004672 MF: protein kinase activity;GO:0004674 MF: protein serine/threonine kinase activity; |
|  |  |  |  |  | TraesCS4A01G026300 | receptor kinase 1 | PF00069: Protein kinase domain; PF00931: NB-ARC domain | IPR000719: Protein kinase domain; IPR002182: NB-ARC; IPR008271: Serine/threonine-protein kinase, active site; IPR011009: Protein kinase-like domain; IPR011991: Winged helix-turn-helix DNA-binding domain; IPR017441: Protein kinase, ATP binding site; IPR027417: P-loop containing nucleoside triphosphate hydrolase; IPR032675: Leucine-rich repeat domain, L domain-like | GO:0004672 MF: protein kinase activity;GO:0005524 MF: ATP binding; |
|  |  |  |  |  | TraesCS4A01G026400 | DNA topoisomerase 3-alpha | NA | NA | NA |
|  |  |  |  |  | TraesCS4A01G026500 | cleavage and polyadenylation specificity factor 160 | NA | NA | NA |
|  |  |  |  |  | TraesCS4A01G026600 | receptor kinase 1 | PF00069: Protein kinase domain; PF00635: MSP (Major sperm protein) domain | IPR000535: Major sperm protein (MSP) domain; IPR000719: Protein kinase domain; IPR008271: Serine/threonine-protein kinase, active site; IPR008962: PapD-like; IPR011009: Protein kinase-like domain; IPR013783: Immunoglobulin-like fold; IPR017441: Protein kinase, ATP binding site | GO:0004672 MF: protein kinase activity;GO:0005524 MF: ATP binding; |
|  |  |  |  |  | TraesCS4A01G026700 | Protein kinase family protein | PF00069: Protein kinase domain | IPR000719: Protein kinase domain; IPR011009: Protein kinase-like domain; IPR017441: Protein kinase, ATP binding site | GO:0004672 MF: protein kinase activity;GO:0005524 MF: ATP binding; |
|  |  |  |  |  | TraesCS4A01G026800 | transmembrane protein, putative (DUF247) | PF03140: Plant protein of unknown function | IPR004158: Protein of unknown function DUF247, plant | NA |
|  |  |  |  |  | TraesCS4A01G026900 | Serine protease HtrA-like | PF13180: PDZ domain | IPR001478: PDZ domain; IPR009003: Peptidase S1, PA clan | GO:0005515 MF: protein binding |
|  |  |  |  |  | TraesCS4A01G027000 | Serine protease HtrA-like | PF13180: PDZ domain | IPR001478: PDZ domain; IPR009003: Peptidase S1, PA clan | GO:0005515 MF: protein binding |
|  |  |  |  |  | TraesCS4A01G027100 | core-2/I-branching beta-1,6-n-acetylglucosaminyltransferase family protein | NA | NA | NA |
|  |  |  |  |  | TraesCS4A01G027200 | Core-2/I-branching beta-1,6-N-acetylglucosaminyltransferase family protein | NA | NA | NA |
|  |  |  |  |  | TraesCS4A01G027300 | NA | NA | NA | NA |
| STI_RSRatio | Kukri_rep_c68594_530 |  | 4D | 12773159 | TraesCS4D01G026700 | 5'-AMP-activated protein kinase subunit beta-1 | PF16561: Glycogen recognition site of AMP-activated protein kinase | IPR013783: Immunoglobulin-like fold; IPR014756: Immunoglobulin E-set; IPR032640: AMP-activated protein kinase, glycogen-binding domain | NA |
|  |  |  |  |  | TraesCS4D01G026800 | NRT1/PTR family protein 2.2 | PF00854: POT family | IPR000109: Proton-dependent oligopeptide transporter family; IPR020846: Major facilitator superfamily domain | GO:0005215 MF: transporter activity;GO:0006810 BP: transport; |
|  |  |  |  |  | TraesCS4D01G026900 | Hexosyltransferase | PF01501: Glycosyl transferase family 8 | IPR002495: Glycosyl transferase, family 8; IPR029044: Nucleotide-diphospho-sugar transferases | GO:0016757 MF: transferase activity, transferring glycosyl groups |
|  |  |  |  |  | TraesCS4D01G027000 | ESX-5 secretion system ATPase EccB5 | NA | NA | NA |
|  |  |  |  |  | TraesCS4D01G027100 | Glycine-rich family protein | NA | IPR015943: WD40/YVTN repeat-like-containing domain | GO:0005515 MF: protein binding |
|  |  |  |  |  | TraesCS4D01G027200 | Heat shock 70 kDa protein 17 | NA | NA | NA |
|  |  |  |  |  | TraesCS4D01G027300 | Methionyl-tRNA formyltransferase | NA | NA | NA |
|  |  |  |  |  | TraesCS4D01G027400 | rRNA N-glycosidase | PF00161: Ribosome inactivating protein | IPR001574: Ribosome-inactivating protein; IPR016138: Ribosome-inactivating protein, subdomain 1 | GO:0017148 BP: negative regulation of translation;GO:0030598 MF: rRNA N-glycosylase activity |
|  |  |  |  |  | TraesCS4D01G027500 | Heat stress transcription factor A-9 | PF07762: Protein of unknown function (DUF1618) | IPR011676: Domain of unknown function DUF1618 | NA |
|  |  |  |  |  | TraesCS4D01G027600 | Calcium-dependent protein kinase | PF00069: Protein kinase domain; PF13499: EF-hand domain pair | IPR000719: Protein kinase domain; IPR002048: EF-hand domain; IPR008271: Serine/threonine-protein kinase, active site; IPR011009: Protein kinase-like domain; IPR011992: EF-hand domain pair; IPR017441: Protein kinase, ATP binding site; IPR018247: EF-Hand 1, calcium-binding site | GO:0004672 MF: protein kinase activity;GO:0005509 MF: calcium ion binding; |
|  |  |  |  |  | TraesCS4D01G027700 | B3 domain-containing protein | PF02362: B3 DNA binding domain | IPR003340: B3 DNA binding domain; IPR015300: DNA-binding pseudobarrel domain | GO:0003677 MF: DNA binding |
|  |  |  |  |  | TraesCS4D01G027800 | Inositol hexakisphosphate and diphosphoinositol-pentakisphosphate kinase | PF00328: Histidine phosphatase superfamily (branch 2) | IPR000560: Histidine phosphatase superfamily, clade-2; IPR029033: Histidine phosphatase superfamily; IPR033379: Histidine acid phosphatase active site | GO:0003993 MF: acid phosphatase activity |
|  |  |  |  |  | TraesCS4D01G027900 | Plasma membrane ATPase | PF00690: Cation transporter/ATPase, N-terminus; PF00122: E1-E2 ATPase; PF00702: haloacid dehalogenase-like hydrolase | IPR001757: P-type ATPase; IPR004014: Cation-transporting P-type ATPase, N-terminal; IPR006534: P-type ATPase, subfamily IIIA; IPR008250: P-type ATPase, A domain; IPR018303: P-type ATPase, phosphorylation site; IPR023214: HAD-like domain; IPR023298: P-type ATPase, transmembrane domain; IPR023299: P-type ATPase, cytoplasmic domain N | GO:0000166 MF: nucleotide binding;GO:0006754 BP: ATP biosynthetic process; |
|  |  |  |  |  | TraesCS4D01G028000 | Tetratricopeptide repeat protein 38 | NA | IPR011990: Tetratricopeptide-like helical domain | GO:0005515 MF: protein binding |
|  |  |  |  |  | TraesCS4D01G028100 | Costars family protein | PF14705: Costars | IPR027817: Costars domain | NA |
|  |  |  |  |  | TraesCS4D01G028200 | FBD-associated F-box protein | NA | IPR001810: F-box domain; IPR032675: Leucine-rich repeat domain, L domain-like | GO:0005515 MF: protein binding |
|  |  |  |  |  | TraesCS4D01G028300 | TBC1 domain family member | PF00566: Rab-GTPase-TBC domain | IPR000195: Rab-GTPase-TBC domain | NA |
|  |  |  |  |  | TraesCS4D01G028400 | RNA-binding family protein isoform 1 | NA | IPR000504: RNA recognition motif domain | GO:0003676 MF: nucleic acid binding |
|  |  |  |  |  | TraesCS4D01G028500 | EBNA-1-like protein | NA | NA | NA |
|  |  |  |  |  | TraesCS4D01G028600 | Serine/threonine-protein kinase | PF01453: D-mannose binding lectin; PF00954: S-locus glycoprotein domain; PF08276: PAN-like domain; PF07714: Protein tyrosine kinase | IPR000719: Protein kinase domain; IPR000858: S-locus glycoprotein domain; IPR001245: Serine-threonine/tyrosine-protein kinase, catalytic domain; IPR001480: Bulb-type lectin domain; IPR003609: PAN/Apple domain; IPR008271: Serine/threonine-protein kinase, active site; IPR011009: Protein kinase-like domain; IPR017441: Protein kinase, ATP binding site | GO:0004672 MF: protein kinase activity;GO:0005524 MF: ATP binding; |
|  |  |  |  |  | TraesCS4D01G028700 | Serine/threonine-protein kinase | PF01453: D-mannose binding lectin; PF00954: S-locus glycoprotein domain; PF08276: PAN-like domain; PF07714: Protein tyrosine kinase | IPR000719: Protein kinase domain; IPR000858: S-locus glycoprotein domain; IPR001245: Serine-threonine/tyrosine-protein kinase, catalytic domain; IPR001480: Bulb-type lectin domain; IPR003609: PAN/Apple domain; IPR008271: Serine/threonine-protein kinase, active site; IPR011009: Protein kinase-like domain; IPR017441: Protein kinase, ATP binding site; IPR024171: S-receptor-like serine/threonine-protein kinase | GO:0004672 MF: protein kinase activity;GO:0004674 MF: protein serine/threonine kinase activity; |
|  |  |  |  |  | TraesCS4D01G028800 | Thioesterase family protein | PF03061: Thioesterase superfamily | IPR003736: Phenylacetic acid degradation-related domain; IPR006683: Thioesterase domain; IPR029069: HotDog domain | NA |
|  |  |  |  |  | TraesCS4D01G028900 | BHLH family protein, putative, expressed | NA | NA | NA |
|  |  |  |  |  | TraesCS4D01G029000 | Thioesterase family protein | PF03061: Thioesterase superfamily | IPR003736: Phenylacetic acid degradation-related domain; IPR006683: Thioesterase domain; IPR029069: HotDog domain | NA |
|  |  |  |  |  | TraesCS4D01G029100 | Octicosapeptide/Phox/Bem1p domain-containing protein kinase | PF00564: PB1 domain | IPR000270: PB1 domain | GO:0005515 MF: protein binding |
|  |  |  |  |  | TraesCS4D01G029200 | Nuclease domain-containing protein 1 | PF00565: Staphylococcal nuclease homologue; PF00567: Tudor domain | IPR002999: Tudor domain; IPR016071: Staphylococcal nuclease (SNase-like), OB-fold; IPR016685: RNA-induced silencing complex, nuclease component Tudor-SN | GO:0016442 CC: RISC complex;GO:0031047 BP: gene silencing by RNA |
|  |  |  |  |  | TraesCS4D01G029300 | Phosphatidylinositol N-acetyglucosaminlytransferase subunit P-like protein | PF14383: DUF761-associated sequence motif; PF14309: Domain of unknown function (DUF4378) | IPR025486: Domain of unknown function DUF4378; IPR032795: DUF3741-associated sequence motif | NA |
|  |  |  |  |  | TraesCS4D01G029400 | Core-2/I-branching beta-1,6-N-acetylglucosaminyltransferase family protein | PF02485: Core-2/I-Branching enzyme | IPR003406: Glycosyl transferase, family 14 | GO:0008375 MF: acetylglucosaminyltransferase activity;GO:0016020 CC: membrane |
|  |  |  |  |  | TraesCS4D01G029500 | Hedgehog-interacting-like protein | PF07995: Glucose / Sorbosone dehydrogenase | IPR011041: Soluble quinoprotein glucose/sorbosone dehydrogenase; IPR011042: Six-bladed beta-propeller, TolB-like; IPR012938: Glucose/Sorbosone dehydrogenase | GO:0003824 MF: catalytic activity;GO:0005975 BP: carbohydrate metabolic process;GO:0016901 MF: oxidoreductase activity, acting on the CH-OH group of donors, quinone or similar compound as acceptor;GO:0048038 MF: quinone binding |
|  |  |  |  |  | TraesCS4D01G029600 | CLAVATA3/ESR (CLE)-related protein 25 | NA | NA | NA |
|  |  |  |  |  | TraesCS4D01G029700 | Protein UPSTREAM OF FLC | PF06136: Domain of unknown function (DUF966) | IPR010369: Protein of unknown function DUF966; IPR021182: Uncharacterised conserved protein UCP031043 | NA |
|  |  |  |  |  | TraesCS4D01G029800 | Beta-1,3-galactosyltransferase-like protein | PF00337: Galactoside-binding lectin; PF01762: Galactosyltransferase | IPR001079: Galectin, carbohydrate recognition domain; IPR002659: Glycosyl transferase, family 31; IPR013320: Concanavalin A-like lectin/glucanase domain | GO:0006486 BP: protein glycosylation;GO:0008378 MF: galactosyltransferase activity; |
|  |  |  |  |  | TraesCS4D01G029900 | DUF538 family protein, putative (Protein of unknown function, DUF538) | PF04398: Protein of unknown function, DUF538 | IPR007493: Protein of unknown function DUF538 | NA |
|  |  |  |  |  | TraesCS4D01G030000 | DUF538 family protein, putative (Protein of unknown function, DUF538) | PF04398: Protein of unknown function, DUF538 | IPR007493: Protein of unknown function DUF538 | NA |
|  |  |  |  |  | TraesCS4D01G030100 | glycine-rich protein | PF07762: Protein of unknown function (DUF1618) | IPR011676: Domain of unknown function DUF1618 | NA |
|  |  |  |  |  | TraesCS4D01G030200 | Protein ENHANCED DISEASE RESISTANCE 2 | PF07059: Protein of unknown function (DUF1336) | IPR009769: Protein ENHANCED DISEASE RESISTANCE 2, C-terminal | NA |
|  |  |  |  |  | TraesCS4D01G030300 | Thiosulfate sulfurtransferase/rhodanese-like domain-containing protein 2 | PF07762: Protein of unknown function (DUF1618) | IPR011676: Domain of unknown function DUF1618 | NA |
| STI_RL | AX-110016919 |  | 5B | 591062022 | TraesCS5B01G415900 | N-acetylglucosaminyl-phosphatidylinositol biosynthetic protein gpi1 | PF05024: N-acetylglucosaminyl transferase component (Gpi1) | IPR007720: N-acetylglucosaminyl transferase component | GO:0006506 BP: GPI anchor biosynthetic process;GO:0016021 CC: integral component of membrane; |
|  |  |  |  |  | TraesCS5B01G416000 | transmembrane protein, putative (DUF1068) | PF06364: Protein of unknown function (DUF1068) | IPR010471: Protein of unknown function DUF1068 | NA |
|  |  |  |  |  | TraesCS5B01G416100 | 60 kDa chaperonin 2 | PF06364: Protein of unknown function (DUF1068) | IPR010471: Protein of unknown function DUF1068 | NA |
|  |  |  |  |  | TraesCS5B01G416200 | Ubiquitin carboxyl-terminal hydrolase 2 | PF13968: Domain of unknown function (DUF4220); PF04578: Protein of unknown function, DUF594 | IPR007658: Protein of unknown function DUF594; IPR025315: Domain of unknown function DUF4220 | NA |
|  |  |  |  |  | TraesCS5B01G416300 | transmembrane protein, putative (DUF1068) | PF06364: Protein of unknown function (DUF1068) | IPR010471: Protein of unknown function DUF1068 | NA |
|  |  |  |  |  | TraesCS5B01G416400 | Hexosyltransferase | PF01501: Glycosyl transferase family 8 | IPR002495: Glycosyl transferase, family 8; IPR029044: Nucleotide-diphospho-sugar transferases | GO:0016757 MF: transferase activity, transferring glycosyl groups |
|  |  |  |  |  | TraesCS5B01G416500 | TOM1-like protein 2 | PF00790: VHS domain; PF03127: GAT domain | IPR002014: VHS domain; IPR004152: GAT domain; IPR008942: ENTH/VHS | GO:0005622 CC: intracellular;GO:0006886 BP: intracellular protein transport |
|  |  |  |  |  | TraesCS5B01G416700 | Citrate synthase | PF00285: Citrate synthase, C-terminal domain | IPR002020: Citrate synthase; IPR016142: Citrate synthase-like, large alpha subdomain; IPR019810: Citrate synthase active site | GO:0006099 BP: tricarboxylic acid cycle;GO:0046912 MF: transferase activity, transferring acyl groups, acyl groups converted into alkyl on transfer |
|  |  |  |  |  | TraesCS5B01G416800 | NBS-LRR disease resistance protein, putative, expressed | PF00931: NB-ARC domain | IPR002182: NB-ARC; IPR011991: Winged helix-turn-helix DNA-binding domain; IPR027417: P-loop containing nucleoside triphosphate hydrolase; IPR032675: Leucine-rich repeat domain, L domain-like | GO:0043531 MF: ADP binding |
|  |  |  |  |  | TraesCS5B01G416900 | Calmodulin-binding protein, putative, expressed | PF07887: Calmodulin binding protein-like | IPR012416: CALMODULIN-BINDING PROTEIN60 | GO:0005516 MF: calmodulin binding;GO:0006950 BP: response to stress |
|  |  |  |  |  | TraesCS5B01G417000 | receptor kinase 1 | PF00069: Protein kinase domain; PF07887: Calmodulin binding protein-like; PF03514: GRAS domain family | IPR000719: Protein kinase domain; IPR005202: Transcription factor GRAS; IPR008271: Serine/threonine-protein kinase, active site; IPR011009: Protein kinase-like domain; IPR012416: CALMODULIN-BINDING PROTEIN60; IPR017441: Protein kinase, ATP binding site | GO:0004672 MF: protein kinase activity;GO:0005516 MF: calmodulin binding; |
|  |  |  |  |  | TraesCS5B01G417200 | Calmodulin-binding protein, putative, expressed | PF07887: Calmodulin binding protein-like | IPR012416: CALMODULIN-BINDING PROTEIN60 | GO:0005516 MF: calmodulin binding;GO:0006950 BP: response to stress |
|  |  |  |  |  | TraesCS5B01G417300 | Calmodulin-binding protein, putative, expressed | PF07887: Calmodulin binding protein-like | IPR012416: CALMODULIN-BINDING PROTEIN60 | GO:0005516 MF: calmodulin binding;GO:0006950 BP: response to stress |
|  |  |  |  |  | TraesCS5B01G417400 | Photosystem I reaction center subunit III | PF02507: Photosystem I reaction centre subunit III | IPR003666: Photosystem I PsaF, reaction centre subunit III | GO:0009522 CC: photosystem I;GO:0009538 CC: photosystem I reaction center;GO:0015979 BP: photosynthesis |
| STI_SL | Excalibur_c5329_1335 | sti_SL_5B_Hap1 | 5B | 580686254 | TraesCS5B01G403500 | Gibberellin-regulated protein 1 | PF02704: Gibberellin regulated protein | IPR003854: Gibberellin regulated protein | NA |
|  |  |  |  |  | TraesCS5B01G403600 | Gibberellin-regulated protein 1 | PF02704: Gibberellin regulated protein | IPR003854: Gibberellin regulated protein | NA |
|  |  |  |  |  | TraesCS5B01G403700 | Chitinase | PF00182: Chitinase class I | IPR000726: Glycoside hydrolase, family 19, catalytic; IPR016283: Glycoside hydrolase, family 19; IPR023346: Lysozyme-like domain | GO:0004568 MF: chitinase activity;GO:0005975 BP: carbohydrate metabolic process; |
|  |  |  |  |  | TraesCS5B01G403800 | GDSL esterase/lipase | PF00657: GDSL-like Lipase/Acylhydrolase | IPR001087: GDSL lipase/esterase | GO:0016788 MF: hydrolase activity, acting on ester bonds |
|  |  |  |  |  | TraesCS5B01G403900 | Upstream activation factor subunit spp27 | PF02201: SWIB/MDM2 domain | IPR003121: SWIB/MDM2 domain; IPR019835: SWIB domain | GO:0005515 MF: protein binding |
|  |  |  |  |  | TraesCS5B01G404000 | Phosphatase 2C family protein | PF00481: Protein phosphatase 2C | IPR000222: PPM-type phosphatase, divalent cation binding; IPR001932: PPM-type phosphatase domain | GO:0003824 MF: catalytic activity;GO:0043169 MF: cation binding |
|  |  |  |  |  | TraesCS5B01G404100 | Ankyrin repeat family protein, putative, expressed | PF13637: Ankyrin repeats (many copies) | IPR002110: Ankyrin repeat; IPR020683: Ankyrin repeat-containing domain; IPR026480: Arginine N-methyltransferase 2-like domain; IPR029063: S-adenosyl-L-methionine-dependent methyltransferase | GO:0005515 MF: protein binding |
|  |  |  |  |  | TraesCS5B01G404200 | Peroxidase | PF00141: Peroxidase | IPR000823: Plant peroxidase; IPR002016: Haem peroxidase, plant/fungal/bacterial; IPR010255: Haem peroxidase; IPR019793: Peroxidases heam-ligand binding site; IPR019794: Peroxidase, active site | GO:0004601 MF: peroxidase activity;GO:0006979 BP: response to oxidative stress;GO:0020037 MF: heme binding;GO:0055114 BP: oxidation-reduction process |
|  |  |  |  |  | TraesCS5B01G404300 | Peroxidase | PF00141: Peroxidase | IPR000823: Plant peroxidase; IPR002016: Haem peroxidase, plant/fungal/bacterial; IPR010255: Haem peroxidase; IPR019793: Peroxidases heam-ligand binding site; IPR019794: Peroxidase, active site | GO:0004601 MF: peroxidase activity;GO:0006979 BP: response to oxidative stress;GO:0020037 MF: heme binding;GO:0055114 BP: oxidation-reduction process |
|  |  |  |  |  | TraesCS5B01G404400 | Pentatricopeptide repeat protein | PF01535: PPR repeat; PF13041: PPR repeat family; PF12854: PPR repeat | IPR002885: Pentatricopeptide repeat | NA |
|  |  |  |  |  | TraesCS5B01G404500 | NA | NA | NA | NA |
|  |  |  |  |  | TraesCS5B01G404600 | Subtilisin-like protease | PF05922: Peptidase inhibitor I9; PF00082: Subtilase family; PF02225: PA domain | IPR000209: Peptidase S8/S53 domain; IPR003137: PA domain; IPR010259: Peptidase S8 propeptide/proteinase inhibitor I9; IPR015500: Peptidase S8, subtilisin-related; IPR023828: Peptidase S8, subtilisin, Ser-active site | GO:0004252 MF: serine-type endopeptidase activity;GO:0006508 BP: proteolysis |
|  |  |  |  |  | TraesCS5B01G404700 | Chaperone protein DnaJ | PF00226: DnaJ domain | IPR001623: DnaJ domain | NA |
|  |  |  |  |  | TraesCS5B01G404800 | Prolyl 4-hydroxylase alpha-like protein | PF13640: 2OG-Fe(II) oxygenase superfamily | IPR005123: Oxoglutarate/iron-dependent dioxygenase; IPR006620: Prolyl 4-hydroxylase, alpha subunit | GO:0005506 MF: iron ion binding;GO:0016491 MF: oxidoreductase activity; |
| STI_SFW | AX-158586104 | sti_SFW_5B_Hap1 | 5B | 560396077 | TraesCS5B01G382000 | NA | NA | NA | NA |
|  |  |  |  |  | TraesCS5B01G382000 | F-box/LRR-repeat protein 17 | NA | IPR001810: F-box domain; IPR032675: Leucine-rich repeat domain, L domain-like | GO:0005515 MF: protein binding |
|  |  |  |  |  | TraesCS5B01G382000 | NA | NA | NA | NA |
| STI_SFW | AX-158600273 |  | 6A | 520581021 | TraesCS6A01G287400 | Nuclear transcription factor Y subunit B | PF00808: Histone-like transcription factor (CBF/NF-Y) and archaeal histone | IPR003958: Transcription factor CBF/NF-Y/archaeal histone domain; IPR009072: Histone-fold | GO:0046982 MF: protein heterodimerization activity |
|  |  |  |  |  | TraesCS6A01G287500 | Nuclear transcription factor Y subunit B | NA | NA | NA |
|  |  |  |  |  | TraesCS6A01G287600 | Fasciclin-like arabinogalactan protein | PF02469: Fasciclin domain | IPR000782: FAS1 domain | NA |
|  |  |  |  |  | TraesCS6A01G287700 | Dof zinc finger protein | PF02701: Dof domain, zinc finger | IPR003851: Zinc finger, Dof-type | GO:0003677 MF: DNA binding;GO:0006355 BP: regulation of transcription, DNA-templated |
|  |  |  |  |  | TraesCS6A01G287800 | BSD domain containing protein | PF03909: BSD domain | IPR005607: BSD domain | NA |
|  |  |  |  |  | TraesCS6A01G287900 | O-fucosyltransferase family protein | PF10250: GDP-fucose protein O-fucosyltransferase | IPR019378: GDP-fucose protein O-fucosyltransferase; IPR024709: O-fucosyltransferase, plant | NA |
|  |  |  |  |  | TraesCS6A01G288000 | Acetolactate synthase | PF02776: Thiamine pyrophosphate enzyme, N-terminal TPP binding domain; PF00205: Thiamine pyrophosphate enzyme, central domain; PF02775: Thiamine pyrophosphate enzyme, C-terminal TPP binding domain | IPR011766: Thiamine pyrophosphate enzyme, C-terminal TPP-binding; IPR012000: Thiamine pyrophosphate enzyme, central domain; IPR012001: Thiamine pyrophosphate enzyme, N-terminal TPP-binding domain; IPR012846: Acetolactate synthase, large subunit, biosynthetic; IPR029035: DHS-like NAD/FAD-binding domain; IPR029061: Thiamin diphosphate-binding fold | GO:0000287 MF: magnesium ion binding;GO:0003824 MF: catalytic activity;G |
|  |  |  |  |  | TraesCS6A01G288100 | Basic helix-loop-helix transcription factor | PF00010: Helix-loop-helix DNA-binding domain | IPR011598: Myc-type, basic helix-loop-helix (bHLH) domain | GO:0046983 MF: protein dimerization activity |
|  |  |  |  |  | TraesCS6A01G288200 | Amino acid transporter family protein | PF01490: Transmembrane amino acid transporter protein | IPR013057: Amino acid transporter, transmembrane domain | NA |
| STI_SFW | AX-158600281 |  | 6A | 520712811 | TraesCS6A01G288300 | 2-oxoglutarate (2OG) and Fe(II)-dependent oxygenase superfamily protein | PF14226: non-haem dioxygenase in morphine synthesis N-terminal; PF03171: 2OG-Fe(II) oxygenase superfamily | IPR005123: Oxoglutarate/iron-dependent dioxygenase; IPR026992: Non-haem dioxygenase N-terminal domain; IPR027443: Isopenicillin N synthase-like | GO:0016491 MF: oxidoreductase activity;GO:0055114 BP: oxidation-reduction process |
|  |  |  |  |  | TraesCS6A01G288400 | 2-oxoglutarate (2OG) and Fe(II)-dependent oxygenase superfamily protein | PF14226: non-haem dioxygenase in morphine synthesis N-terminal; PF03171: 2OG-Fe(II) oxygenase superfamily | IPR005123: Oxoglutarate/iron-dependent dioxygenase; IPR026992: Non-haem dioxygenase N-terminal domain; IPR027443: Isopenicillin N synthase-like | GO:0016491 MF: oxidoreductase activity;GO:0055114 BP: oxidation-reduction process |
| STI_SFW | wsnp_Ku_c4296_7807837 |  | 6A | 520717537 | TraesCS6A01G288600 | NBS-LRR-like resistance protein | NA | IPR027417: P-loop containing nucleoside triphosphate hydrolase | NA |
|  |  |  |  |  | TraesCS6A01G288700 | NBS-LRR-like resistance protein | NA | IPR027417: P-loop containing nucleoside triphosphate hydrolase | NA |
|  |  |  |  |  | TraesCS6A01G288800 | RING/U-box superfamily protein | PF13639: Ring finger domain | IPR001841: Zinc finger, RING-type; IPR013083: Zinc finger, RING/FYVE/PHD-type | GO:0005515 MF: protein binding;GO:0008270 MF: zinc ion binding |
|  |  |  |  |  | TraesCS6A01G288900 | BZIP transcription factor | PF00170: bZIP transcription factor | IPR004827: Basic-leucine zipper domain | GO:0003700 MF: transcription factor activity, sequence-specific DNA binding;GO:0006355 BP: regulation of transcription, DNA-templated;GO:0043565 MF: sequence-specific DNA binding |
|  |  |  |  |  | TraesCS6A01G289000 | Eukaryotic translation initiation factor | PF01873: Domain found in IF2B/IF5; PF02020: eIF4-gamma/eIF5/eIF2-epsilon | IPR002735: Translation initiation factor IF2/IF5; IPR003307: W2 domain; IPR016024: Armadillo-type fold; IPR016189: Translation initiation factor IF2/IF5, N-terminal; IPR016190: Translation initiation factor IF2/IF5, zinc-binding | GO:0003743 MF: translation initiation factor activity;GO:0005488 MF: binding; |
|  |  |  |  |  | TraesCS6A01G289100 | RmlC-like jelly roll fold protein | PF06172: Cupin superfamily (DUF985) | IPR009327: Cupin domain of unknown function DUF985; IPR011051: RmlC-like cupin domain; IPR014710: RmlC-like jelly roll fold | NA |
|  |  |  |  |  | TraesCS6A01G289200 | Signal peptide protease | PF01343: Peptidase family S49 | IPR002142: Peptidase S49; IPR004634: Peptidase S49, protease IV; IPR004635: Peptidase S49, SppA; IPR029045: ClpP/crotonase-like domain | GO:0006465 BP: signal peptide processing;GO:0006508 BP: proteolysis; |
|  |  |  |  |  | TraesCS6A01G289300 | LAG1 longevity assurance-like protein | PF03798: TLC domain | IPR006634: TRAM/LAG1/CLN8 homology domain; IPR016439: Ceramide synthase component Lag1/Lac1 | GO:0005783 CC: endoplasmic reticulum;GO:0016021 CC: integral component of membrane |
|  |  |  |  |  | TraesCS6A01G289400 | CONSTANS-like 1 protein | PF00643: B-box zinc finger; PF06203: CCT motif | IPR000315: B-box-type zinc finger; IPR010402: CCT domain | GO:0005515 MF: protein binding;GO:0005622 CC: intracellular;GO:0008270 MF: zinc ion binding |
|  |  |  |  |  | TraesCS6A01G289500 | Receptor-like kinase | PF08263: Leucine rich repeat N-terminal domain; PF13855: Leucine rich repeat; PF07714: Protein tyrosine kinase | IPR000719: Protein kinase domain; IPR001245: Serine-threonine/tyrosine-protein kinase, catalytic domain; IPR001611: Leucine-rich repeat; IPR008271: Serine/threonine-protein kinase, active site; IPR011009: Protein kinase-like domain; IPR013210: Leucine-rich repeat-containing N-terminal, plant-type; IPR017441: Protein kinase, ATP binding site; IPR032675: Leucine-rich repeat domain, L domain-like | GO:0004672 MF: protein kinase activity;GO:0005515 MF: protein binding; |
|  |  |  |  |  | TraesCS6A01G289600 | H/ACA ribonucleoprotein complex subunit 2-like protein | PF01248: Ribosomal protein L7Ae/L30e/S12e/Gadd45 family | IPR002415: H/ACA ribonucleoprotein complex, subunit Nhp2, eukaryote; IPR004038: Ribosomal protein L7Ae/L30e/S12e/Gadd45; IPR018492: Ribosomal protein L7Ae/L8/Nhp2 family; IPR029064: 50S ribosomal protein L30e-like | GO:0003723 MF: RNA binding;GO:0005730 CC: nucleolus |
|  |  |  |  |  | TraesCS6A01G289700 | ribosomal RNA-processing 7 protein | PF12923: Ribosomal RNA-processing protein 7 (RRP7) | IPR024326: Ribosomal RNA-processing protein 7 | NA |
|  |  |  |  |  | TraesCS6A01G289800 | Tetraspanin family protein | PF00335: Tetraspanin family | IPR018499: Tetraspanin/Peripherin | GO:0016021 CC: integral component of membrane |
| STI_SFW | AX-158535753 |  | 6B | 51225226 | TraesCS6B01G073900 | Ubiquitin thioesterase | PF10275: Peptidase C65 Otubain | IPR003323: OTU domain; IPR019400: Peptidase C65, otubain | NA |
|  |  |  |  |  | TraesCS6B01G074000 | Histone H4 | PF15511: Centromere kinetochore component CENP-T histone fold | IPR001951: Histone H4; IPR009072: Histone-fold; IPR019809: Histone H4, conserved site | GO:0000786 CC: nucleosome;GO:0003677 MF: DNA binding; |
|  |  |  |  |  | TraesCS6B01G074100 | Cytochrome P450 family protein | PF00067: Cytochrome P450 | IPR001128: Cytochrome P450; IPR002401: Cytochrome P450, E-class, group I; IPR017972: Cytochrome P450, conserved site | GO:0005506 MF: iron ion binding;GO:0016705 MF: oxidoreductase activity, acting on paired donors, with incorporation or reduction of molecular oxygen; |
|  |  |  |  |  | TraesCS6B01G074200 | Laccase | PF07732: Multicopper oxidase; PF00394: Multicopper oxidase; PF07731: Multicopper oxidase | IPR001117: Multicopper oxidase, type 1; IPR002355: Multicopper oxidase, copper-binding site; IPR008972: Cupredoxin; IPR011706: Multicopper oxidase, type 2; IPR011707: Multicopper oxidase, type 3; IPR033138: Multicopper oxidases, conserved site | GO:0005507 MF: copper ion binding;GO:0016491 MF: oxidoreductase activity; |
|  |  |  |  |  | TraesCS6B01G074300 | Copine-1 | PF00168: C2 domain; PF07002: Copine | IPR000008: C2 domain; IPR002035: von Willebrand factor, type A; IPR010734: Copine | GO:0005515 MF: protein binding |
|  |  |  |  |  | TraesCS6B01G074400 | F-box family protein | PF00646: F-box domain | IPR001810: F-box domain | GO:0005515 MF: protein binding |
|  |  |  |  |  | TraesCS6B01G074500 | F-box family protein | PF00646: F-box domain | IPR001810: F-box domain | GO:0005515 MF: protein binding |
|  |  |  |  |  | TraesCS6B01G074600 | F-box domain containing protein, expressed | NA | NA | NA |
|  |  |  |  |  | TraesCS6B01G074700 | F-box family protein | PF12937: F-box-like | IPR001810: F-box domain | GO:0005515 MF: protein binding |
|  |  |  |  |  | TraesCS6B01G074800 | RNA-binding KH domain-containing protein | PF00013: KH domain | IPR004087: K Homology domain; IPR004088: K Homology domain, type 1 | GO:0003676 MF: nucleic acid binding;GO:0003723 MF: RNA binding |
|  |  |  |  |  | TraesCS6B01G074900 | F-box family protein | PF00646: F-box domain | IPR001810: F-box domain | GO:0005515 MF: protein binding |
|  |  |  |  |  | TraesCS6B01G075000 | F-box family protein | NA | IPR001810: F-box domain; IPR011043: Galactose oxidase/kelch, beta-propeller | GO:0005515 MF: protein binding |
|  |  |  |  |  | TraesCS6B01G075100 | F-box family protein | PF12937: F-box-like | IPR001810: F-box domain | GO:0005515 MF: protein binding |
|  |  |  |  |  | TraesCS6B01G075200 | NAC domain protein | PF02365: No apical meristem (NAM) protein | IPR003441: NAC domain | GO:0003677 MF: DNA binding;GO:0006355 BP: regulation of transcription, DNA-templated |
|  |  |  |  |  | TraesCS6B01G075300 | F-box and associated interaction domains-containing protein | PF07734: F-box associated | IPR006527: F-box associated domain, type 1 | NA |
|  |  |  |  |  | TraesCS6B01G075400 | F-box family protein | PF12937: F-box-like | IPR001810: F-box domain; IPR011043: Galactose oxidase/kelch, beta-propeller | GO:0005515 MF: protein binding |
|  |  |  |  |  | TraesCS6B01G075500 | F-box family protein | PF12937: F-box-like | IPR001810: F-box domain; IPR011042: Six-bladed beta-propeller, TolB-like | GO:0005515 MF: protein binding |
|  |  |  |  |  | TraesCS6B01G075600 | NBS-LRR disease resistance protein-like protein | PF00931: NB-ARC domain | IPR002182: NB-ARC; IPR003593: AAA+ ATPase domain; IPR011991: Winged helix-turn-helix DNA-binding domain; IPR027417: P-loop containing nucleoside triphosphate hydrolase; IPR032675: Leucine-rich repeat domain, L domain-like | GO:0043531 MF: ADP binding |
|  |  |  |  |  | TraesCS6B01G075700 | NBS-LRR disease resistance protein-like protein | PF00931: NB-ARC domain | IPR002182: NB-ARC; IPR003593: AAA+ ATPase domain; IPR011991: Winged helix-turn-helix DNA-binding domain; IPR027417: P-loop containing nucleoside triphosphate hydrolase; IPR032675: Leucine-rich repeat domain, L domain-like | GO:0043531 MF: ADP binding |
|  |  |  |  |  | TraesCS6B01G075800 | SAUR-like auxin-responsive protein family, putative | PF02519: Auxin responsive protein | IPR003676: Small auxin-up RNA | GO:0009733 BP: response to auxin |
|  |  |  |  |  | TraesCS6B01G075900 | SAUR-like auxin-responsive protein family, putative | PF02519: Auxin responsive protein | IPR003676: Small auxin-up RNA | GO:0009733 BP: response to auxin |
|  |  |  |  |  | TraesCS6B01G076100 | Peptide transporter | PF00854: POT family | IPR000109: Proton-dependent oligopeptide transporter family; IPR020846: Major facilitator superfamily domain | GO:0005215 MF: transporter activity;GO:0006810 BP: transport;GO:0016020 CC: membrane |
|  |  |  |  |  | TraesCS6B01G076200 | F-box family protein | PF00646: F-box domain | IPR001810: F-box domain; IPR017451: F-box associated interaction domain | GO:0005515 MF: protein binding |
| STI_RL | D_contig78519_72 |  | 7D | 10724734 | TraesCS7D01G021200 | Protein kinase family protein | PF00069: Protein kinase domain; PF00931: NB-ARC domain | IPR000719: Protein kinase domain; IPR002182: NB-ARC; IPR008271: Serine/threonine-protein kinase, active site; IPR011009: Protein kinase-like domain; IPR017441: Protein kinase, ATP binding site; IPR027417: P-loop containing nucleoside triphosphate hydrolase | GO:0004672 MF: protein kinase activity;GO:0005524 MF: ATP binding; |
|  |  |  |  |  | TraesCS7D01G021300 | Disease resistance protein (NBS-LRR class) family | NA | IPR032675: Leucine-rich repeat domain, L domain-like | NA |
|  |  |  |  |  | TraesCS7D01G021400 | NA | NA | NA | NA |
|  |  |  |  |  | TraesCS7D01G021500 | Lectin-like protein kinase | PF00139: Legume lectin domain | IPR001220: Legume lectin domain; IPR013320: Concanavalin A-like lectin/glucanase domain; IPR019825: Legume lectin, beta chain, Mn/Ca-binding site | GO:0030246 MF: carbohydrate binding |
|  |  |  |  |  | TraesCS7D01G021600 | F-box protein | PF00646: F-box domain | IPR001810: F-box domain; IPR011043: Galactose oxidase/kelch, beta-propeller; IPR015915: Kelch-type beta propeller; IPR017451: F-box associated interaction domain | GO:0005515 MF: protein binding |
|  |  |  |  |  | TraesCS7D01G021700 | F-box protein | PF00646: F-box domain | IPR001810: F-box domain; IPR015915: Kelch-type beta propeller | GO:0005515 MF: protein binding |
|  |  |  |  |  | TraesCS7D01G021800 | F-box protein | PF00646: F-box domain | IPR001810: F-box domain | GO:0005515 MF: protein binding |
|  |  |  |  |  | TraesCS7D01G021900 | disease resistance family protein / LRR family protein | NA | IPR011991: Winged helix-turn-helix DNA-binding domain; IPR032675: Leucine-rich repeat domain, L domain-like | NA |
|  |  |  |  |  | TraesCS7D01G022000 | receptor-like protein kinase 1 | PF00069: Protein kinase domain | IPR000719: Protein kinase domain; IPR008271: Serine/threonine-protein kinase, active site; IPR011009: Protein kinase-like domain; IPR017441: Protein kinase, ATP binding site | GO:0004672 MF: protein kinase activity;GO:0005524 MF: ATP binding; |
|  |  |  |  |  | TraesCS7D01G022100 | Disease resistance protein (NBS-LRR class) family | PF00931: NB-ARC domain | IPR002182: NB-ARC; IPR011991: Winged helix-turn-helix DNA-binding domain; IPR027417: P-loop containing nucleoside triphosphate hydrolase; IPR032675: Leucine-rich repeat domain, L domain-like | GO:0043531 MF: ADP binding |
|  |  |  |  |  | TraesCS7D01G022200 | Protein kinase family protein | PF00069: Protein kinase domain | IPR000719: Protein kinase domain; IPR008271: Serine/threonine-protein kinase, active site; IPR011009: Protein kinase-like domain | GO:0004672 MF: protein kinase activity;GO:0005524 MF: ATP binding; |
|  |  |  |  |  | TraesCS7D01G022300 | Disease resistance protein (NBS-LRR class) family | PF00931: NB-ARC domain | IPR001611: Leucine-rich repeat; IPR002182: NB-ARC; IPR011991: Winged helix-turn-helix DNA-binding domain; IPR027417: P-loop containing nucleoside triphosphate hydrolase; IPR032675: Leucine-rich repeat domain, L domain-like | GO:0005515 MF: protein binding;GO:0043531 MF: ADP binding |
|  |  |  |  |  | TraesCS7D01G022400 | Disease resistance protein (NBS-LRR class) family | PF00931: NB-ARC domain; PF02362: B3 DNA binding domain | IPR002182: NB-ARC; IPR003340: B3 DNA binding domain; IPR011991: Winged helix-turn-helix DNA-binding domain; IPR015300: DNA-binding pseudobarrel domain; IPR027417: P-loop containing nucleoside triphosphate hydrolase; IPR032675: Leucine-rich repeat domain, L domain-like | GO:0003677 MF: DNA binding;GO:0043531 MF: ADP binding |
|  |  |  |  |  | TraesCS7D01G022500 | Protein FRA10AC1 | PF09725: Folate-sensitive fragile site protein Fra10Ac1 | IPR019129: Folate-sensitive fragile site protein Fra10Ac1 | NA |
|  |  |  |  |  | TraesCS7D01G022600 | RING finger protein | PF13639: Ring finger domain | IPR001841: Zinc finger, RING-type; IPR013083: Zinc finger, RING/FYVE/PHD-type | GO:0005515 MF: protein binding;GO:0008270 MF: zinc ion binding |
|  |  |  |  |  | TraesCS7D01G022700 | WAT1-related protein | PF00892: EamA-like transporter family | IPR000620: EamA domain | GO:0016020 CC: membrane;GO:0016021 CC: integral component of membrane |
|  |  |  |  |  | TraesCS7D01G022800 | WAT1-related protein | PF00892: EamA-like transporter family | IPR000620: EamA domain | GO:0016020 CC: membrane;GO:0016021 CC: integral component of membrane |
|  |  |  |  |  | TraesCS7D01G022900 | Invertase inhibitor | PF04043: Plant invertase/pectin methylesterase inhibitor | IPR006501: Pectinesterase inhibitor domain | GO:0004857 MF: enzyme inhibitor activity |
|  |  |  |  |  | TraesCS7D01G023000 | RING finger protein | PF13639: Ring finger domain | IPR001841: Zinc finger, RING-type; IPR011016: Zinc finger, RING-CH-type; IPR013083: Zinc finger, RING/FYVE/PHD-type | GO:0005515 MF: protein binding;GO:0008270 MF: zinc ion binding |
|  |  |  |  |  | TraesCS7D01G023100 | RING finger family protein | PF13639: Ring finger domain | IPR001841: Zinc finger, RING-type; IPR013083: Zinc finger, RING/FYVE/PHD-type | GO:0005515 MF: protein binding;GO:0008270 MF: zinc ion binding |
|  |  |  |  |  | TraesCS7D01G023200 | NA | NA | NA | NA |
|  |  |  |  |  | TraesCS7D01G023300 | Cytochrome P450 | PF00067: Cytochrome P450 | IPR001128: Cytochrome P450; IPR002401: Cytochrome P450, E-class, group I; IPR017972: Cytochrome P450, conserved site | GO:0005506 MF: iron ion binding;GO:0016705 MF: oxidoreductase activity, acting on paired donors, with incorporation or reduction of molecular oxygen; |
|  |  |  |  |  | TraesCS7D01G023400 | F-box protein | PF00646: F-box domain | IPR001810: F-box domain; IPR011043: Galactose oxidase/kelch, beta-propeller; IPR015915: Kelch-type beta propeller | GO:0005515 MF: protein binding |
|  |  |  |  |  | TraesCS7D01G023500 | F-box/kelch-repeat protein At1g74510 | NA | IPR015915: Kelch-type beta propeller | GO:0005515 MF: protein binding |
|  |  |  |  |  | TraesCS7D01G023600 | ATP-dependent zinc metalloprotease FtsH | PF00004: ATPase family associated with various cellular activities (AAA) | IPR003593: AAA+ ATPase domain; IPR003959: ATPase, AAA-type, core; IPR003960: ATPase, AAA-type, conserved site; IPR027417: P-loop containing nucleoside triphosphate hydrolase | GO:0005524 MF: ATP binding |
|  |  |  |  |  | TraesCS7D01G023700 | F-box protein | PF00646: F-box domain | IPR001810: F-box domain; IPR015915: Kelch-type beta propeller | GO:0005515 MF: protein binding |
|  |  |  |  |  | TraesCS7D01G023800 | ATP-dependent RNA helicase | NA | NA | NA |
|  |  |  |  |  | TraesCS7D01G023900 | DEAD-box ATP-dependent RNA helicase 21 | NA | NA | NA |
|  |  |  |  |  | TraesCS7D01G024000 | BTB/POZ/MATH-domain protein | PF00651: BTB/POZ domain | IPR000210: BTB/POZ domain; IPR002083: MATH/TRAF domain; IPR008974: TRAF-like; IPR011333: SKP1/BTB/POZ domain | GO:0005515 MF: protein binding |
|  |  |  |  |  | TraesCS7D01G024100 | NBS-LRR resistance-like protein | NA | IPR032675: Leucine-rich repeat domain, L domain-like | NA |
|  |  |  |  |  | TraesCS7D01G024200 | Haloacid dehalogenase-like hydrolase family protein | PF13419: Haloacid dehalogenase-like hydrolase | IPR006439: HAD hydrolase, subfamily IA; IPR023198: Phosphoglycolate phosphatase, domain 2; IPR023214: HAD-like domain | GO:0008152 BP: metabolic process;GO:0016787 MF: hydrolase activity |
|  |  |  |  |  | TraesCS7D01G024300 | Pentatricopeptide repeat-containing protein | PF01535: PPR repeat; PF13041: PPR repeat family | IPR002885: Pentatricopeptide repeat; IPR011990: Tetratricopeptide-like helical domain | GO:0005515 MF: protein binding |
|  |  |  |  |  | TraesCS7D01G024400 | Ubiquitin carboxyl-terminal hydrolase family protein | PF02148: Zn-finger in ubiquitin-hydrolases and other protein; PF00443: Ubiquitin carboxyl-terminal hydrolase | IPR001394: Peptidase C19, ubiquitin carboxyl-terminal hydrolase; IPR001607: Zinc finger, UBP-type; IPR013083: Zinc finger, RING/FYVE/PHD-type; IPR028889: Ubiquitin specific protease domain | GO:0008270 MF: zinc ion binding;GO:0016579 BP: protein deubiquitination; |
|  |  |  |  |  | TraesCS7D01G024500 | Cytochrome P450 family protein, expressed | PF00067: Cytochrome P450 | IPR001128: Cytochrome P450; IPR002401: Cytochrome P450, E-class, group I; IPR017972: Cytochrome P450, conserved site | GO:0005506 MF: iron ion binding;GO:0016705 MF: oxidoreductase activity, acting on paired donors, with incorporation or reduction of molecular oxygen; |
|  |  |  |  |  | TraesCS7D01G024600 | Polyubiquitin | PF00240: Ubiquitin family | IPR000626: Ubiquitin domain; IPR019956: Ubiquitin; IPR029071: Ubiquitin-related domain | GO:0005515 MF: protein binding |
|  |  |  |  |  | TraesCS7D01G024700 | NBS-LRR resistance-like protein | PF00931: NB-ARC domain | IPR002182: NB-ARC; IPR011991: Winged helix-turn-helix DNA-binding domain; IPR027417: P-loop containing nucleoside triphosphate hydrolase; IPR032675: Leucine-rich repeat domain, L domain-like | GO:0043531 MF: ADP binding |
|  |  |  |  |  | TraesCS7D01G024800 | tonoplast monosaccharide transporter3 | NA | NA | NA |
|  |  |  |  |  | TraesCS7D01G024900 | Disease resistance protein (NBS-LRR class) family | PF00931: NB-ARC domain | IPR002182: NB-ARC; IPR003593: AAA+ ATPase domain; IPR011991: Winged helix-turn-helix DNA-binding domain; IPR027417: P-loop containing nucleoside triphosphate hydrolase; IPR032675: Leucine-rich repeat domain, L domain-like | GO:0043531 MF: ADP binding |
|  |  |  |  |  | TraesCS7D01G025000 | Ribosomal protein L5 | NA | IPR022803: Ribosomal protein L5 domain | NA |
|  |  |  |  |  | TraesCS7D01G025100 | F-box family protein | PF12937: F-box-like | IPR001810: F-box domain | GO:0005515 MF: protein binding |

**Table S4. Pearson’s correlation coefficients among root and shoot traits under H_2_O_2_ treatment in the evaluated wheat association panel**

| **Traits** | **RLH** | **SLH** | **RSRatio_H** | **TLH** | **SFWH** | **SDWH** | **RFWH** |
| --- | --- | --- | --- | --- | --- | --- | --- |
| **RLH** |  |  |  |  |  |  |  |
| **SLH** | 0.58**** |  |  |  |  |  |  |
| **RSRatioH** | 0.49**** | -0.40**** |  |  |  |  |  |
| **TLH** | 0.89**** | 0.89**** | 0.05 |  |  |  |  |
| **SFWH** | 0.54**** | 0.86**** | -0.35*** | 0.78**** |  |  |  |
| **SDWH** | 0.27** | 0.38**** | -0.07 | 0.36**** | 0.37**** |  |  |
| **RFWH** | 0.30*** | 0.30*** | 0.01 | 0.34**** | 0.44**** | 0.11 |  |
| **RDWH** | -0.23** | -0.19* | 0.07 | -0.24** | -0.13 | -0.14 | 0.16 |

Abbreviations: *p* <0.0001 '****'; *p* <0.001 '***'; *p* < 0.01 '**'; *p* <0.05 '*', RLH, Root length under H_2_O_2_ stress; SLH, Shoot length under H_2_O_2_ stress, RSRatioH, Root-shoot ratio under H_2_O_2_ stress; TLH, Total length under H_2_O_2_ stress; SFWH, Shoot fresh weight under H_2_O_2_ stress; SDWH, Shoot dry weight under H_2_O_2_ stress; RFWH, Root fresh weight under H_2_O_2_ stress; RDWC, Root dry weight under H_2_O_2_ stress

**Table S5a: Relative root length of modern and traditional cultivar groups carrying the favorable allele (GTGAGCC) of Rel_SL_1A_Hap1**

| **Cultivar Group** | **Name** | **Year of Release** | **Relative Shoot length** |
| --- | --- | --- | --- |
| Modern | Lucius | 2006 | 104.29 |
|  | Oakley | 2008 | 103.01 |
|  | Memory | 2013 | 102.58 |
|  | Hermann | 2004 | 102.22 |
|  | Kalahari | 2010 | 101.94 |
|  | **Average** | | **102.81** |
| Traditional | Claire | 1999 | 100.14 |
|  | Ivanka | 1998 | 86.41 |
|  | Apache | 1997 | 84.48 |
|  | Flair | 1996 | 99.03 |
|  | Isengrain | 1996 | 97.36 |
|  | Renesansa | 1995 | 80.82 |
|  | Batis | 1994 | 96.17 |
|  | Transit | 1994 | 93.52 |
|  | Tambor | 1993 | 90.16 |
|  | Gaucho | 1993 | 86.52 |
|  | Aron | 1992 | 92.23 |
|  | Tarso | 1992 | 90.83 |
|  | Ibis | 1991 | 88.80 |
|  | Konsul | 1990 | 99.60 |
|  | Contra | 1990 | 95.08 |
|  | Kontrast | 1990 | 94.92 |
|  | Toronto | 1990 | 81.37 |
|  | Greif | 1989 | 100.38 |
|  | Astron | 1989 | 88.21 |
|  | Orestis | 1988 | 101.76 |
|  | Obelisk | 1987 | 92.50 |
|  | Soissons | 1987 | 80.42 |
|  | Sorbas | 1985 | 95.38 |
|  | Knirps | 1985 | 91.41 |
|  | Sperber | 1982 | 96.18 |
|  | Kraka | 1982 | 88.45 |
|  | Phoenix | 1981 | 98.34 |
|  | Camp Remy | 1980 | 105.13 |
|  | Basalt | 1980 | 97.34 |
|  | Rektor | 1980 | 95.53 |
|  | Oberst | 1980 | 94.05 |
|  | Granada | 1980 | 89.85 |
|  | Benni multifloret | 1980 | 89.72 |
|  | TJB 990-15 | 1980 | 88.69 |
|  | Lambriego Inia | 1980 | 83.56 |
|  | Avalon | 1980 | 79.52 |
|  | Brigand | 1979 | 97.36 |
|  | Aquila | 1979 | 89.24 |
|  | Götz | 1978 | 90.91 |
|  | Vuka | 1975 | 97.83 |
|  | Cardos | 1975 | 96.59 |
|  | Carisuper | 1975 | 95.37 |
|  | Disponent | 1975 | 93.83 |
|  | Nimbus | 1975 | 92.22 |
|  | Benno | 1973 | 95.30 |
|  | Cajeme 71 | 1971 | 100.67 |
|  | Caribo | 1968 | 92.47 |
|  | Highbury | 1968 | 87.51 |
|  | Sonalika | 1967 | 82.92 |
|  | Aristos | 1966 | 93.21 |
|  | Siete Cerros 66 | 1966 | 89.33 |
|  | Mironovska 808 | 1963 | 89.82 |
|  | **Average** | | **92.27** |

**Table S5 b. Stress tolerance index (sti) of modern and traditional cultivar groups carrying the favourable allele (CGGT) of sti_SL_1A_Hap1**

| **Cultivar Group** | **Name** | **Year of Release** | **stress tolerance index** |
| --- | --- | --- | --- |
| Modern | Skater | 2000 | 1.00 |
|  | Caphorn | 2000 | 0.68 |
|  | Sokrates | 2001 | 0.98 |
|  | Cubus | 2002 | 0.79 |
|  | SW Topper | 2002 | 0.88 |
|  | Enorm | 2002 | 0.73 |
|  | Winnetou | 2002 | 0.99 |
|  | Cordiale | 2003 | 0.62 |
|  | Einstein | 2004 | 0.75 |
|  | Robigus | 2004 | 0.75 |
|  | Kerubino | 2004 | 1.05 |
|  | Akratos | 2004 | 1.08 |
|  | Impression | 2005 | 0.87 |
|  | Aszita | 2005 | 1.03 |
|  | Alixan | 2005 | 0.77 |
|  | Carenius | 2006 | 0.78 |
|  | Mulan | 2006 | 0.86 |
|  | Manager | 2006 | 0.77 |
|  | Lucius | 2006 | 0.78 |
|  | Arlequin | 2007 | 0.81 |
|  | Oakley | 2008 | 0.72 |
|  | Jafet | 2008 | 0.77 |
|  | Tabasco | 2008 | 0.80 |
|  | Zappa | 2009 | 0.76 |
|  | Global | 2009 | 0.98 |
|  | Meister | 2010 | 0.83 |
|  | KWS Pius | 2010 | 0.77 |
|  | Linus | 2010 | 0.75 |
|  | Kalahari | 2010 | 0.81 |
|  | Orcas | 2010 | 0.77 |
|  | KWS Santiago | 2011 | 0.75 |
|  | Nelson | 2011 | 0.76 |
|  | Glaucus | 2011 | 1.00 |
|  | Mentor | 2012 | 0.80 |
|  | Estivus | 2012 | 1.10 |
|  | Capone | 2012 | 0.79 |
|  | Forum | 2012 | 0.79 |
|  | Atomic | 2012 | 0.83 |
|  | Bombus | 2012 | 0.81 |
|  | WW 4180 | 2012 | 0.72 |
|  | Rebell | 2013 | 0.77 |
|  | Kurt | 2013 | 0.67 |
|  | Anapolis | 2013 | 0.81 |
|  | Edward | 2013 | 0.86 |
|  | Gourmet | 2013 | 0.75 |
|  | Average | | 0.93 |
| Traditional | Cappelle Desprez | 1946 | 0.97 |
|  | Mironovska 808 | 1963 | 1.04 |
|  | Aristos | 1966 | 0.95 |
|  | Sonalika | 1967 | 0.76 |
|  | Caribo | 1968 | 0.91 |
|  | Centurk | 1971 | 0.98 |
|  | Joss | 1972 | 1.04 |
|  | Benno | 1973 | 0.95 |
|  | Maris Huntsman | 1975 | 1.07 |
|  | Vuka | 1975 | 1.00 |
|  | Kobold | 1978 | 0.99 |
|  | Brigand | 1979 | 0.77 |
|  | Aquila | 1979 | 1.06 |
|  | TJB 990-15 | 1980 | 0.71 |
|  | NaturaSt | 1980 | 1.27 |
|  | Rektor | 1980 | 0.95 |
|  | Oberst | 1980 | 0.94 |
|  | Basalt | 1980 | 1.06 |
|  | Benni multifloret | 1980 | 0.84 |
|  | Camp Remy | 1980 | 0.79 |
|  | Avalon | 1980 | 0.87 |
|  | Kraka | 1982 | 1.03 |
|  | Obelisk | 1987 | 1.01 |
|  | Alidos | 1987 | 0.92 |
|  | Greif | 1989 | 0.82 |
|  | Astron | 1989 | 0.99 |
|  | Toronto | 1990 | 1.02 |
|  | Contra | 1990 | 0.72 |
|  | Konsul | 1990 | 0.94 |
|  | Kontrast | 1990 | 0.95 |
|  | Aron | 1992 | 0.90 |
|  | Gaucho | 1993 | 0.97 |
|  | Tambor | 1993 | 0.88 |
|  | Transit | 1994 | 0.96 |
|  | Batis | 1994 | 0.97 |
|  | Renesansa | 1995 | 0.89 |
|  | Flair | 1996 | 0.82 |
|  | Isengrain | 1996 | 0.68 |
|  | Apache | 1997 | 0.85 |
|  | Claire | 1999 | 0.93 |
|  | Average | | 0.83 |

**Table S6. The haplotype blocks associated with different traits and their chromosomal positions and alleles**

| **Trait** | **Haplotype block** | **Significant marker (s)** | **NMHB** | **Chr** | **No. of Genes** | **Favorable allele** |
| --- | --- | --- | --- | --- | --- | --- |
| STI_SL | sti_SL_1A_Hap1 | AX-158595571 | 4 | 1A | 2 | CGGT |
| STI_RL | sti_RL_2B_Hap1 | wsnp_JD_c52_87219, Excalibur_c11392_1193 | 6 | 2B | 31 | CACGAC |
| STI_SL | sti_SL_5B_Hap1 | Excalibur_c5329_1335 | **7** | 5B | 15 | AGCCCGA |
| STI_SFW | sti_SFW_5B_Hap1 | AX-158586104 | 5 | 5B | 3 | GACGG |
| Relative_RFW | Rel_RFW_1B_Hap1 | Excalibur_c7954_672 | 4 | 1B | 1 | GCGG |
| Relative_RFW | Rel_RFW_1B_Hap2 | RAC875_c28894_526,  wsnp_Ex_c11976_19193550 | 4 | 1B | 29 | AGGT |
| Relative_SL | Rel_SL_1B_Hap1 | AX-158540096, AX-158560878, Tdurum_contig94450_255 | 7 | 1B | 3 | GTGAGCC |
| Relative_SFW | Rel_SFW_3A_Hap1 | AX-158532834 | 19 | 3A | 26 | GGGGCTGCCGGAACCATTTA |
| Relative_RL | Rel_RL_3B_Hap1 | BobWhite_c10402_140,  wsnp_JD_c30422_23944042 | 4 | 3B | 1 | CCCC |
| Relative_RFW | Rel_RFW_6B_Hap1 | AX-158528874,  BS00011795_51 | 4 | 6B | 9 | CAGT |
| Relative_RL and Relative_RSRatio | Com_Hap1 | AX-158582574 | 5 | 4B | 157 | TCGGG |

Abbreviations: NMHB, number of markers in haplotype block; Chr, Chromosome
